# Actinide Pnictinidene Chemistry: A Terminal Thorium Parent‐Arsinidene Complex Stabilised by a Super‐Bulky Triamidoamine Ligand

**DOI:** 10.1002/anie.202211627

**Published:** 2022-11-16

**Authors:** Jingzhen Du, Gábor Balázs, John A. Seed, Jonathan D. Cryer, Ashley J. Wooles, Manfred Scheer, Stephen T. Liddle

**Affiliations:** ^1^ Department of Chemistry The University of Manchester Oxford Road Manchester M13 9PL UK; ^2^ Institute of Inorganic Chemistry University of Regensburg Universitätsstr. 31 93053 Regensburg Germany

**Keywords:** Actinides, Arsinidene, Density Functional Theory, Multiple Bonds, Phosphinidene

## Abstract

We report the direct synthesis of the terminal pnictidenes [An(Tren^TCHS^)(PnH)][M(2,2,2‐cryptand)] (Tren^TCHS^={N(CH_2_CH_2_NSiCy_3_)_3_}^3−^; An/Pn/M=Th/P/Na **5**, Th/As/K **6**, U/P/Na **7**, U/As/K **8**) and their conversion to the pnictides [An(Tren^TCHS^)(PnH_2_)] (An/Pn=Th/P **9**, Th/As **10**, U/P **11**, U/As **12**). Use of the super‐bulky Tren^TCHS^ ligand was essential to accessing complete families, and **6** is an unprecedented example of a terminal thorium‐arsinidene complex and only the second structurally authenticated parent terminal arsinidene complex of any metal. Comparison of the terminal Th=AsH unit of **6** to the bridging ThAs(H)K linkage in structurally analogous [Th(Tren^TIPS^){μ‐As(H)K(15‐crown‐5)}] (Tren^TIPS^={N(CH_2_CH_2_NSiPr^i^_3_)_3_}^3−^) reveals a stronger Th−As bond in the former compared to the latter, and a large response overall to the nature of the Th=AsH bonding upon removal of the electrostatically‐bound K‐ion; the σ‐bond changes little but the π‐bond is significantly perturbed.

## Graphical abstract

Introducing a new super‐bulky triamidoamine ligand to actinide chemistry has enabled the previously elusive terminal parent thorium‐arsinidene linkage, that is the first terminal thorium‐arsinidene, to be synthesised and isolated. Quantum chemical calculations reveal a surprisingly large change to the Th=AsH linkage compared to an electrostatically‐bound potassium‐capped analogue.

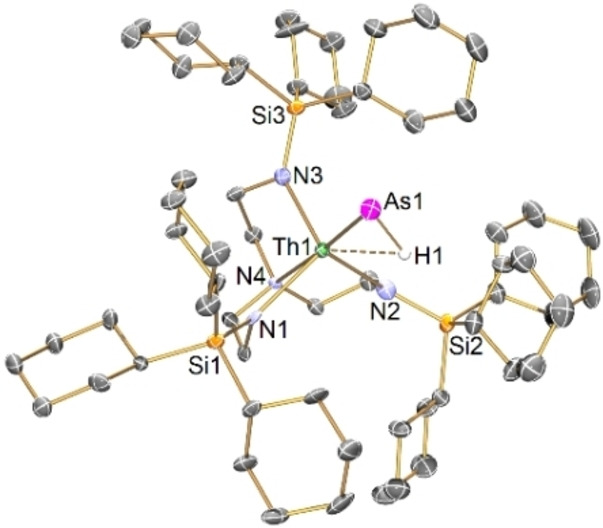

## Introduction

There is burgeoning interest in the covalency of actinide‐ligand (An‐L) bonding^[1]^ involving soft P‐ and S‐donor ligands.[^2^, ^3^] However, in contrast to An−N and −O multiple bond chemistry, An‐molecules containing heavier pnictinidenes (RPn^2−^) (Pn=P, As, Sb, Bi), and indeed An−Pn Zintl clusters, are less well‐developed,^[4]^ despite being fundamental carbene analogues, and metal‐pnictinidenes (M=PnR) are key agents in pnictinidene group‐transfer reactions.^[5]^ Amongst the M=PnR family, terminal parent M=PnH linkages, lacking a sterically demanding R group to protect the M=Pn bond, are uniquely analogous to the M=NH unit that is a key intermediate in N_2_ reduction to NH_3_.^[6]^ However, due to the reactive triplet ground state, unsaturated valence shell, and relatively large size of heavier Pn ions, previous studies were mainly restricted to spectroscopic species,^[7]^ or a small number of bridging PnH stabilised by transition metals.^[8]^

Structurally authenticated molecules with terminal (i.e. the PnR group is bound to only one metal) PnH groups are sparse across the whole Periodic Table, with a handful of terminal parent phosphinidene (M=PH) compounds known for p‐,^[9]^ d‐,^[10]^ and f‐blocks.^[11]^ Terminal arsinidene (M=AsR) congeners have emerged for p‐^[12]^ and f‐block^[13]^ derivatives recently, but M=AsH remains unknown for the d‐block; indeed, only five M=AsR d‐block complexes have been crystallographically characterised in the past 28 years.[^14^, ^15^, ^16^, ^17^] Stabilisation of the parent arsinidene functional group AsH at hard, highly Lewis acidic actinide centres such as thorium and uranium, amongst the largest ions in the Periodic Table, is therefore inherently challenging.

**Figure 1:**
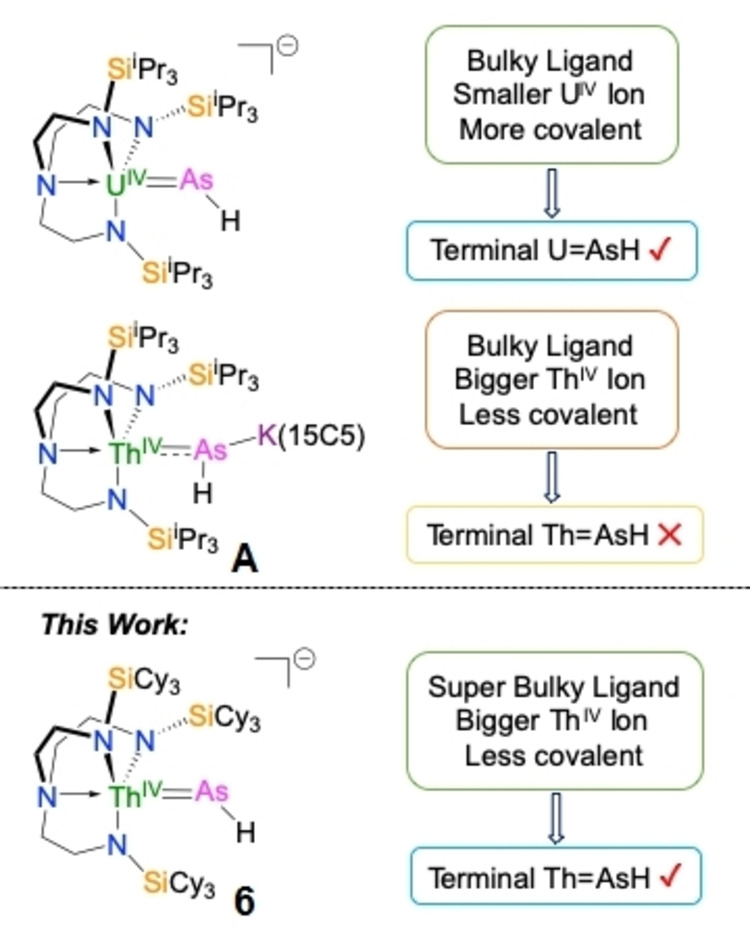
Previous and the current work.

Previously, we reported An−PH_2_, An=PH, An−P(H)−An, and AnPAn species (An=Th, U) using the sterically demanding Tren^TIPS^ ligand ({N(CH_2_CH_2_NSi^i^Pr_3_)_3_}^3−^).[^11^, ^18^] This chemistry could be extended to include U−AsH_2_, U≡AsK_2_, and UAsU, and U=AsH linkages, the latter of which was the first terminal parent metal‐arsinidene, Figure 1.^[12]^ In contrast, all attempts to prepare a terminal Th=AsH analogue were unsuccessful,^[19]^ reflecting the decreased stabilisation of the soft AsH by the larger, harder, and more ionic Th compared to U (the bond radius of Th is typically ≈0.5–0.18 Å larger than U).^[20]^ Indeed, although [Th(Tren^TIPS^){(μ‐AsH)K(15C5)}] (**A**, 15C5=15‐crown‐5 ether, Figure 1) could be prepared, exhaustive attempts to remove the capping {K(15C5)}^+^ resulted in decomposition to intractable/unidentified products highlighting real chemical differences between Th and U.^[19]^ Thus, a terminal Th=AsR species of any type remains unknown. Tren^TIPS^ has proven to be a privileged ligand stabilising many novel An‐L linkages, but the above suggested that a new super‐bulky ligand would be required to isolate a terminal Th=AsH linkage, Figure 1.

Here, we report the synthesis of the new Tren^TCHS^ ligand (Tren^TCHS^={N(CH_2_CH_2_NSiCy_3_)_3_}^3−^, Cy=cyclohexyl) which has enabled the synthesis, isolation, and characterisation of a terminal Th=AsH linkage, Figure 1. The use of this super‐bulky ligand has enabled an examination of the Th=AsH unit free of capping‐group effects on the bonding, which contributes to examining periodic trends across the 5 f‐series more generally, and further highlights the significant chemical behavioural differences between uranium and thorium.

## Results and Discussion

### Syntheses and Solid‐State Structures of New Precursors

The new ligand transfer reagent [(Tren^TCHS^)Li_3_] (Tren^TCHS^={N(CH_2_CH_2_NSiCy_3_)_3_}^3−^, Cy=cyclohexyl) was prepared in multi‐gram scales and good yields (11 g, 56 %) using a similar approach to that of Tren^TIPS^.[^21^, ^22^] As part of this work, a more reliable synthesis of Cy_3_SiCl on bulk scale (28 g, 92 %) was established; indeed obtaining pure Cy_3_SiCl proved essential to preparing Tren^TCHS^H_3_. The solid‐state structure of solvent‐free [(Tren^TCHS^)Li_3_] (Figure S1) was determined, and although the data are poor precluding meaningful discussion of metrical parameters, the structure is consistent with its spectroscopic and elemental analyses data that confirm the bulk formulation.^[22]^

**Scheme 1:**
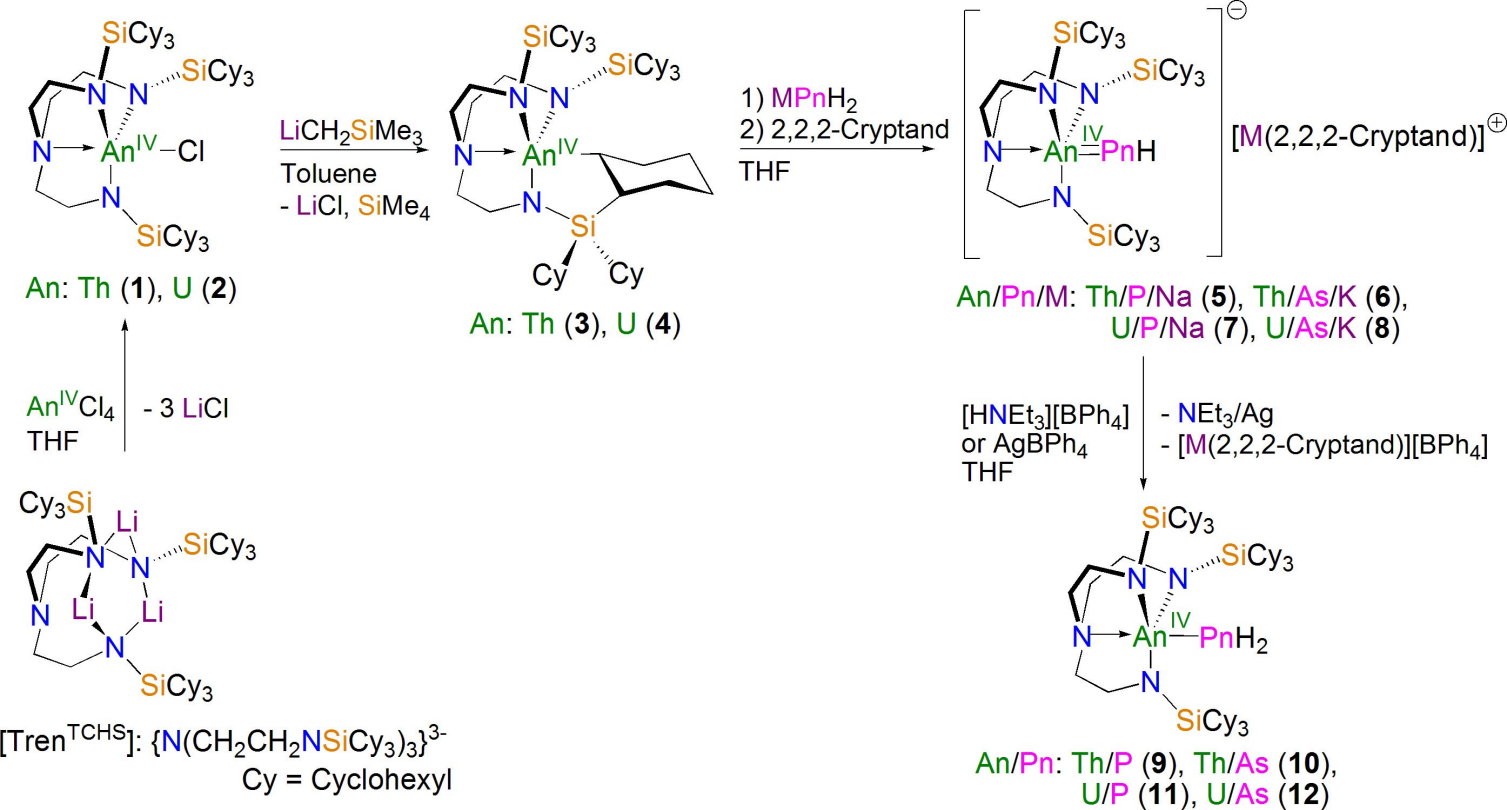
Synthesis of actinide complexes **1**–**12** reported in this study. Salt metathesis reactions of [Tren^TCHS^Li_3_] with AnCl_4_ (An=Th, U) in THF produces **1** and **2**, respectively, which further react with LiCH_2_SiMe_3_ separately in toluene to give the corresponding cyclometallated **3** and **4**. Protonation of the cyclometallated **3** and **4** with NaPH_2_ or KAsH_2_ separately in the presence of 2,2,2‐cryptand using THF as the solvent affords the parent phosphinidene and arsinidene complexes **5**–**8**, respectively. Treatment of **5**–**8** with AgBPh_4_ or [HNEt_3_][BPh_4_] separately yields the parent phosphide and arsenide products **9**–**12**. The structures of **5**, **6**, **9**, and **10** are shown in Figure 2, the remaining structures can be found in the Supporting Information.

Treatment of ThCl_4_(THF)_3.5_ or UCl_4_ with [(Tren^TCHS^)Li_3_] in THF produces [An(Tren^TCHS^)(Cl)] (An=Th, **1**; U, **2**) as colourless and green solids in good yields (63, 74 %), respectively, Scheme 1 1.^[22]^ The ^1^H and ^29^Si{^1^H} NMR spectra of **1** and **2** suggest effective *C*_3v_ geometries in solution (Figure S34–37). The molecular structures of **1** and **2** reveal solvent‐free, monomeric trigonal‐bipyramidal metal centres (Figure S2/3); the amine‐ and Cl‐donors occupy the axial sites, and the latter sits in a pocket defined by the three SiCy_3_ groups that is deeper than Tren^TIPS^, in principle supporting superior stabilisation of reactive moieties. The An−N_amide_, An−N_amine_, and An−Cl distances are unremarkable and statistically indistinguishable to those of [An(Tren^TIPS^)Cl] (An=Th, U),[^21^, ^23^] suggesting that the steric protection of the apical pocket has been increased compared to Tren^TIPS^ without unduly affecting the core TrenAn units.

Treating **1** or **2** with one equivalent of LiCH_2_SiMe_3_ produces the cyclometallate compounds [An{N(CH_2_CH_2_NSiCy_3_)_2_(CH_2_CH_2_NSiCy_2_[CHCH_2_CH_2_CH_2_CH_2_CH])}] (An=Th, **3**; U, **4**) as colourless and red solids, respectively, in good yields (≈65 %), Scheme 1.^[22]^ Complexes **3** and **4** are insoluble in aromatic solvents and poorly soluble even in THF, which has precluded their characterisation in solution, but elemental analyses confirm their bulk formulations and the U^IV^ assignment of **4** is verified by SQUID magnetometry (Figure S65/66). The solid‐state structures of **3** and **4** (Figure S4/5) reveal Th−C and U−C bond lengths of 2.547(5) and 2.492(7) Å that are comparable to those in Tren^TIPS^ cyclometallate An‐compounds.^[23]^

Attempts to prepare the separated ion pairs [An(Tren^TCHS^)(solvent)][BPh_4_] (An=Th or U; solvent=DME or THF) by treating **3** or **4** with [HNEt_3_][BPh_4_] in DME or THF, respectively, were unsuccessful, which we attribute to poor solubility and steric demands leading to kinetic suppression of reactivity. This is notable because this exact methodology works straightforwardly for Tren^TIPS^ analogues, producing [An(Tren^TIPS^)(solvent)][BPh_4_] (An=Th, solvent=DME; An=U, solvent=THF) which are key precursors for subsequent reactions.^[11]^

### Direct Phosphinidene and Arsinidene Syntheses and Characterisation

Given the absence of [An(Tren^TCHS^)(solvent)][BPh_4_], preventing direct preparation of An‐PnH_2_ species—these softer pnictides do not displace halides from TrenAn species in salt elimination reactions—an alternative synthetic approach needed to be considered. Thus, we examined combining **3**, finely ground NaPH_2_ or KAsH_2_, and 2,2,2‐cryptand in 1 : 1 : 1 ratio in THF resulting in white slurries that turned into yellow and orange solutions, respectively, after each stirring at room temperature for 2 hours. After work‐up and recrystallisation from Et_2_O or THF, the terminal thorium parent phosphinidene and arsinidene complexes [Th(Tren^TCHS^)(PH)][Na(2,2,2‐cryptand)] (**5**) and [Th(Tren^TCHS^)(AsH)][K(2,2,2‐cryptand)] (**6**) were isolated as yellow and orange crystalline solids in 52 and 46 % yields, respectively, Scheme 1.^[22]^ The direct synthesis of **6** is particularly notable, because several bonds are concomitantly broken and formed during its formation, presenting numerous opportunities to decompose, yet the yield of this direct route of installing a terminal Th=AsH linkage is still reasonable. All prior attempts to access a terminal Th=AsH linkage stabilised by Tren^TIPS^ were unsuccessful, with only capped species isolable.^[19]^ Thus, the isolation of **6** underscores the super‐bulky nature of Tren^TCHS^ compared to the already sterically demanding nature of Tren^TIPS^.

The ^1^H and ^13^C{^1^H} NMR spectra of **5** and **6** are similar, exhibiting 7 and 9 resonances ranging from −2 and 4 ppm and 25 to 75 ppm, respectively (Figure S38–40 and S44–46). The ^1^H resonances for Th=PH and Th=AsH were identified at −0.41 (doublet, *J*_PH_=68.6 Hz) and −1.55 ppm (singlet) in the respective ^1^H NMR spectra. The ^29^Si{^1^H} NMR spectra reveal single resonances at −4.92 and −5.13 ppm, respectively (Figure S41/47), suggesting pseudo‐*C*_3_ symmetry at the thorium centres on the NMR timescale. In agreement with this, the ^31^P NMR of **5** has a doublet resonance at 266.2 ppm (*J*_PH_=68.6 Hz, Figure S42/43). Attempts to record the ^75^As NMR spectrum of **6** using a saturated solution in D_8_‐THF were unfruitful, likely due to the likely highly asymmetric electric field gradient at the quadrupolar arsenic centre in **6**.

The ATR‐IR spectra of **5** and **6** exhibit broad P−H and As−H stretches at 2072 and 1867 cm^−1^, respectively (Figure S19/20), which are identical to those in [Th(Tren^TIPS^)(PH)][Na(12C4)_2_] (2072 cm^−1^; 12C4=12‐crown‐4 ether)^[11b]^ and **A** (1867 cm^−1^),^[19]^ consistent with the structural data on **1** and **2** that suggested the TrenAn core is affected little by the larger SiCy_3_ substituents compared to SiPr^i^_3_. The Raman spectra of **5** and **6** were collected, with weak inelastic scattering bands at 296 and 197 cm^−1^ assigned as Th−P and Th−As stretches, respectively (Figure S27/28). DFT analytical frequency calculations on **5** and **6** compute P−H, As−H, Th−P, and Th−As vibrations at 2092, 1865, 306, and 199 cm^−1^, respectively, in good agreement with the experimental IR and Raman data.

For comparative purposes, the uranium phosphinidene and arsinidene congeners [U(Tren^TCHS^)(PH)][Na(2,2,2‐cryptand)] (**7**) and [U(Tren^TCHS^)(AsH)][K(2,2,2‐cryptand)] (**8**) were prepared in moderate yields (≈50 %) as dark green solids, Scheme 1.^[22]^ The spectroscopic and magnetic data are consistent with the formulations of **7** and **8**. Of particular note are the ^31^P NMR, ATR‐IR, and Raman data.^[22]^ For the former, in a saturated solution in D_8_‐THF we were able to observe a resonance assigned as the phosphinidene centre at 2629 ppm in the ^31^P NMR spectrum of **7** (Figure S50); the corresponding resonance for [U(Tren^TIPS^)(PH)][K(B15C5)_2_] (B15C5=benzo‐15‐crown‐5 ether) could not be observed, but for comparison the ^31^P NMR spectra of [U(Tren^TIPS^)(PH)(K‐2,2,2‐cryptand)] and [U(Tren^TIPS^)(PH_2_)] exhibited resonances at 2460 and 595 ppm, respectively.^[11c]^ For the vibrational data of **7** and **8** broad bands at 2070, 1867, 305, and 202 cm^−1^ are assigned as the P−H, As−H, U−P, and U−As vibrations, respectively (Figure S21/22 and 29/30), from DFT analytical frequency calculations which compute those vibrations to be at 2066, 1838, 296, and 189 cm^−1^, respectively.

### Solid‐State Phosphinidene and Arsinidene Structures

**Figure 2:**
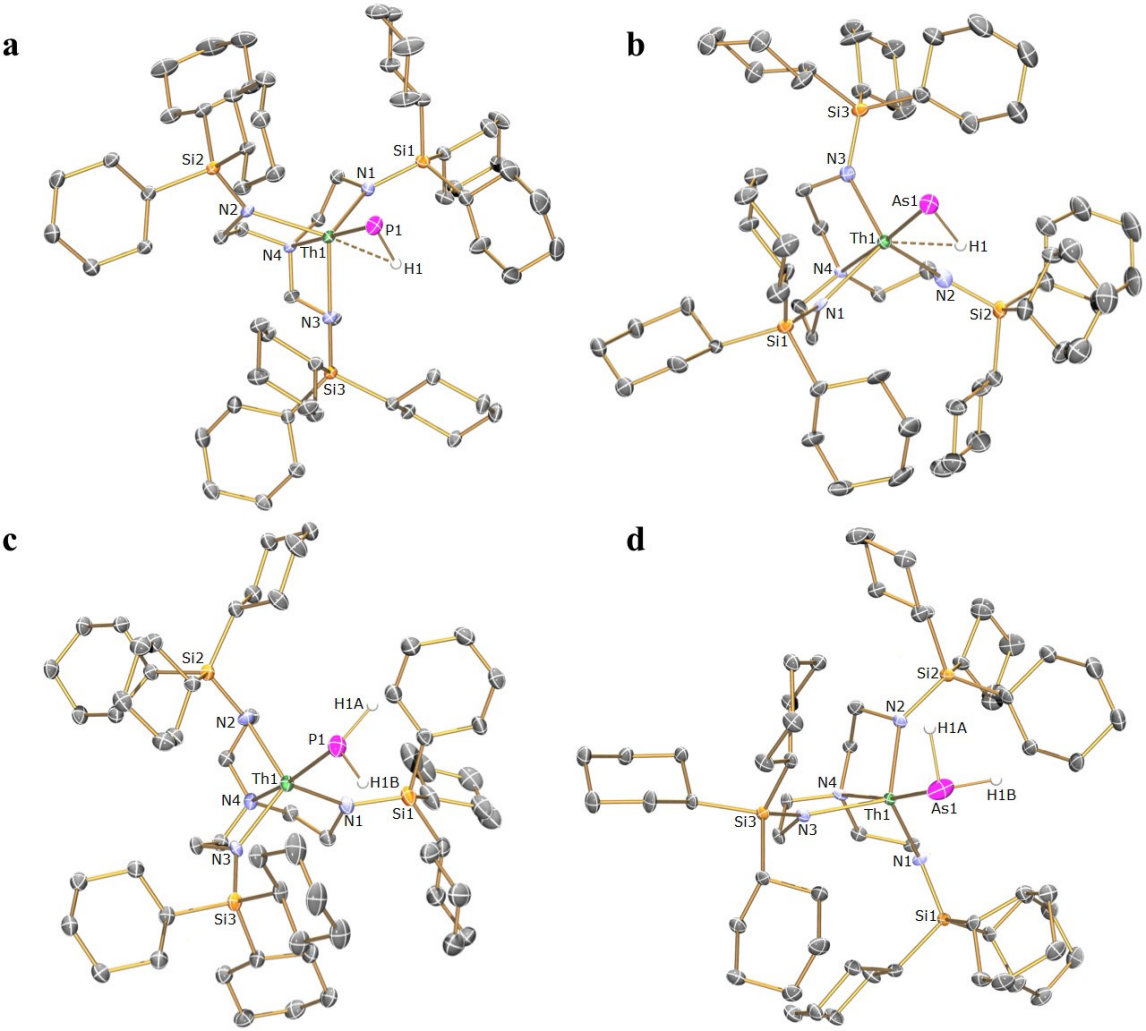
Molecular structures of a) **5**, b) **6**, c) **9**, and d) **10** at 150 K depicted with selective atom labels and 40 % probability displacement ellipsoids. Hydrogen atoms except for the hydrogen atoms at PnH and PnH_2_ groups, cationic [Na(222‐cryptand)]^+^ and [K(222‐cryptand)]^+^ components for **5** and **6** respectively, minor disorder components, and lattice solvent molecules are omitted for clarity. Selected distances (Å): **5**—Th−P=2.7237(9), Th⋅⋅⋅H=2.510(4), P−H=1.466(4); **6**—Th−As=2.8521(8), Th⋅⋅⋅H=2.488(3), As−H=1.574(3); **9—**Th−P=3.0360(15), P−H=1.434(11) and 1.432(10); **10**—Th−As=3.0736(6), As−H=1.550(5) and 1.546(5).

The formulations of **5**–**8** were confirmed by their solid‐state structures, Figure 2a/b and S8/9. The common and salient feature of these structures is that the PH or AsH ligands are terminally coordinated to the An‐centre, being well protected by the SiCy_3_ groups, and the anionic and cationic components are well separated.

The Th−As bond length in **6** is 2.8521(8) Å, which is in‐between the sum of the single and double bond radii of thorium and arsenic elements (2.96 and 2.57 Å, respectively)^[24]^ and close to experimentally determined thorium‐arsenic bond lengths with multiple bonding interactions observed in reported arsinidiide or bridging dithorium arsinido compounds: 2.8565(7) Å in **A**;^[19]^ 2.8787(6) Å in [{(C_5_Me_5_)_2_Th}_2_(μ‐AsMes)_2_] (Mes=2,4,6‐Me_3_C_6_H_2_);^[25]^ 2.7994(4) Å in [{(C_5_Me_5_)_2_Th[μ‐As(H)Tripp](μ‐AsTripp)}K]_2_ (Tripp=2,4,6‐Pr^i^_3_C_6_H_2_);^[26]^ and 2.8063(14)/2.8060(14) Å in [{Th(Tren^TIPS^)}_2_(μ‐As)][K(15C5)_2_].^[19]^ The Th−As distance in **6** is significantly shorter than Th−As single bond lengths of 2.913(2)‐3.044(2), 3.0028(6) and 3.065(3) Å reported in [{Th(η^5^‐1,3‐Bu^t^_2_C_5_H_3_)_2_}_2_(μ‐η^3^ : η^3^‐As_6_)],^[27]^ [Th(η^5^‐C_5_Me_5_)_2_{As(H)Tripp}_2_],^[26]^ and [Th(Tren^TIPS^)(AsH_2_)],^[19]^ respectively.

The Th−P, U−P, and U−As bond lengths in **5**, **7**, and **8** are 2.7237(9), 2.6381(12), and 2.7581(6) Å, respectively,^[22]^ which compare well with the corresponding distances of 2.7584(18), 2.613(2), and 2.7159(13) Å for the structurally similar species [Th(Tren^TIPS^)(PH)][Na(12‐crown‐4)_2_],^[11b]^ [U(Tren^TIPS^)(PH)][K(B15C5)_2_]^[11c]^ and [U(Tren^TIPS^)(AsH)][K(B15C5)_2_],^[13]^ respectively. The Th−/U−N_amide_ and Th−/U−N_amine_ bond lengths are unremarkable.[^11^, ^13^]

Within **5**–**8**, An⋅⋅⋅H interactions are found, contrasting to the linear U=N−H linkage found in [U(Tren^TIPS^)(NH)][K(15C5)_2_],^[6e]^ as evidenced by bent An−Pn−H angles of 65.83(17)°, 60.50(12)°, 65.23(13)°, and 61.18(12)° and An⋅⋅⋅H distances of 2.510(4), 2.488(3), 2.423(3), and 2.429(3) Å for **5**–**8**, respectively. These metrics were determined by a combination of initial atom location in the Fourier difference map combined with crystallographic refinement guided by DFT calculations. Acute M−Pn−H angles (≈59–64°) have previously been found for H_2_M=PnH (M=Ti, Zr, Hf, Th, U)[^7a^, ^7b^] in cryogenic matrix isolation experiments and in structurally authenticated [Zr(Tren^DMBS^)(PH)][K(B15C5)_2_] (Zr−P−H angle: 66.7(8)°; Tren^DMBS^={N(CH_2_CH_2_NSiMe_2_Bu^t^)_3_}^3−^),^[10]^ [Th(Tren^TIPS^)(PH)][Na(12C4)_2_] (Th−P−H angle: 67.45(8)°),^[11b]^ **A** (Th−As−H angle: 79.1(2)°),^[20]^ but this interaction is relaxed in [U(Tren^TIPS^)(PH)][K(B15C5)_2_] (U−P−H angle: 118.8(9)°)^[11c]^ and [U(Tren^TIPS^)(AsH)][K(B15C5)_2_] (U−As−H angle: 90(6)°).^[13]^ We suggest this variance may depend on electronic factors, such as how electron rich M is (e.g. 5f^0^6d^0^ of Th^IV^ vs 5f^2^6d^0^ of U^IV^), the extent of Pn−H hydridic character, and sterics, where the larger SiCy_3_ substituents might enforce acute An−Pn−H angles that can relax with the less sterically demanding SiPr^i^_3_ substituents if electronic factors are congruent. The An⋅⋅⋅H distances of **5**–**8** are similar to those computed for H_2_An=PnH species (An=Th, U) found in matrix isolation experiments (≈2.4–2.5 Å),^[7a]^ and together with the similar An‐Pn−H angles these metrical data suggest that the bonding of **5**–**8** and H_2_An=PnH may be similar. However, we note that the Pn−H stretches for **5**–**8** are ≈600 (Pn=P) and ≈400 (Pn=As) cm^−1^ higher than found for matrix isolated H_2_An=PnH suggesting stronger Pn−H and hence weaker An⋅⋅⋅H bonding interactions in **5**–**8**. In passing it is worth mentioning that the completely separated [Na/K(2,2,2‐cryptand)]^+^ cation components of **5**–**8** are distinct because in Tren^TIPS^ analogues the [K(2,2,2‐cryptand)]^+^ unit remains bonded to U=PnH,[^11c^, ^13^] further indicating the advantage of Tren^TCHS^ ligand system in stabilising true terminal pnictidinene species uniquely including the central target of terminal Th=AsH.

### Reactivity of Phosphinidene and Arsinidene Complexes and Salient Aspects of Phosphide and Arsenide Complexes

Demonstrating the basic and nucleophilic nature of **5**–**8**, we find that treatment of **5**–**8** with [HNEt_3_][BPh_4_] in THF results in the isolation of the parent phosphide and arsenide complexes [An(Tren^TCHS^)(PnH_2_)] (An/Pn=Th/P, **9**; Th/As, **10**; U/P, **11**; U/As, **12**), in reasonable yields (46–65 %), Scheme 1 1.^[22]^ This contrasts to the absence of reactivity between **3**/**4** with [HNEt_3_][BPh_4_], and also that the protonations are selective and do not result in the formation of **3**/**4** and Pn_*x*_H_*y*_‐type species. It is also notable that the AnPnH_2_ species are prepared from AnPnH and not *vice versa* as would be conventionally anticipated.[^11c^, ^13^]

Alternatively, **11** and **12** can be prepared by oxidation of **7** and **8** with AgBPh_4_ in benzene. The formation of **11** and **12** in these oxidation reactions may involve a transient U^V^=PH or U^V^=AsH species which then abstracts a proton in the reaction with concomitant reduction due to the HSAB mismatch of An and Pn; certainly, protonation of U≡As to give U=AsH when stabilising K^+^ ions are abstracted is known.^[13]^ The reactivity of **7** and **8** contrasts to the disproportionation observed for Tren^TIPS^ supported U^V^=NH chemistry which produces U^IV^−NH_2_ and U^VI^≡N.^[6e]^ That said, in parallel we have found that oxidation of [U(Tren^TIPS^)(PH)][K(B15C5)_2_] with AgBPh_4_ produces [U(Tren^TIPS^)(PH_2_)]^[11c]^ and [{U(Tren^TIPS^)}_2_(μ : η^2^‐η^2^‐P_2_)],^[28]^ which implies formation of a transient U^V^=PH linkage that disproportionates to U^IV^−PH_2_ and U^VI^≡P, the latter of which could dimerise. However, we have only been able to identify **11** when **7** is oxidised and so the formation of [{U(Tren^TCHS^)}_2_(μ : η^2^‐η^2^‐P_2_)],^[28]^ and hence a disproportionation rather than proton abstraction mechanism, remains unconfirmed.

As expected, the Th−P, Th−As, U−P, and U−As bond lengths of 3.0360(15), 3.0736(4), 2.8725(13), and 2.9855(8) Å in **9**–**12** are significantly longer An−Pn distances than in **5**–**8**, respectively, Figure 2c/d and S12/13. The ATR‐IR data of **9**–**12** reveal broad Pn−H absorptions at 2262, 2062, 2259, and 2064 cm^−1^, respectively (Figure S23–26), which DFT analytical frequency calculations resolve as H−Pn−H A_1_/E stretches at 2317/2270, 2083/2030, 2294/2274, and 2064/2050 cm^−1^.

### Quantum Chemical Calculations

To probe the electronic structure of the An−Pn linkages in **5**–**12** we performed single‐point energy calculations on the geometry optimised structures of the full anionic components of **5**–**8** (**5′**–**8′**) and neutral **9**–**12**.^[22]^ The calculated bond lengths and angles are within 0.05 Å and 2° of the experimentally determined structures. Therefore we conclude that the calculated structures represent qualitative models of the electronic structures of **5′**–**12**, Table 1.

**Table 1:** Selected computed Density Functional Theory, Natural Bond Orbital, and Quantum Theory of Atoms in Molecules data for **5′**–**12**.

	**Bond lengths and indices**		**MDC atomic charges**		**NBO σ‐component^[f]^**				**NBO π‐component** **(or localised lone pair)^[f]^**				**QTAIM parameters^[g]^**			
**Entry^[a]^**	**An−Pn^[b]^**	**BI^[c]^**	***Q***_***An***_^**[d]**^	***Q***_**Pn**_^**[e]**^	**%An**	**%Pn**	**An s : p : d : f%**	**Pn s : p**	**%An**	**%Pn**	**An s : p : d : f%**	**Pn s : p**	**ρ(r)**	**∇^2^ρ(r)**	***H*** **(r)**	***ϵ*(r)**
**5**	2.7084	1.61	2.18	−0.86	14	86	1 : 0 : 59 : 40	28 : 72	22	78	0 : 0 : 69 : 31	0 : 100	0.07	0.05	−0.03	0.28
**6**	2.8319	1.38	2.35	−0.84	14	86	2 : 0 : 56 : 42	25 : 75	21	79	0 : 0 : 63 : 37	0 : 100	0.06	0.05	−0.02	0.29
**7**	2.6147	1.78	2.33	−0.88	26	74	0 : 0 : 24 : 76	10 : 90	34	66	0 : 0 : 31 : 69	0 : 100	0.08	0.05	−0.04	0.22
**8**	2.7438	1.50	2.25	−0.86	26	74	1 : 0 : 25 : 74	9 : 91	36	64	0 : 0 : 28 : 72	0 : 100	0.07	0.05	−0.03	0.20
**9**	3.0146	0.82	2.45	−0.41	8	92	10 : 1 : 54 : 35	35 : 65	0	100	–	38 : 62	0.05	0.03	−0.02	0.05
**10**	3.1046	0.73	2.59	−0.33	9	91	12 : 1 : 53 : 34	32 : 68	0	100	–	48 : 52	0.04	0.03	−0.01	0.03
**11**	2.9045	0.87	2.79	−0.45	14	86	4 : 1 : 38 : 57	11 : 89	0	100	–	64 : 36	0.05	0.04	−0.02	0.04
**12**	3.0330	0.74	2.94	−0.41	15	85	7 : 1 : 34 : 58	12 : 88	0	100	–	69 : 31	0.05	0.03	−0.02	0.03
**A**	2.8880	1.09	2.55	−1.47	9	91	8 : 0 : 48 : 44	10 : 90	9	91	0 : 0 : 37 : 63	0 : 100	0.06	0.06	−0.02	0.34
AsH	–	–	–	+0.28	–	100	–	0 : 100	–	100	–	0 : 100	–	–	–	
(AsH)^2−^	–	–	–	−1.90	–	100	–	0 : 100	–	100	–	0 : 100	–	–	–	

[a] All molecules geometry optimised without symmetry constraints at the LDA VWN BP TZP/ZORA level. [b] Calculated An−Pn (An=Th, U; Pn=P, As) distances (Å). [c] Mayer bond indices. [d] MDC‐q charges on An metal. [e] MDC‐q charges on Pn. [f] Natural Bond Orbital (NBO) analyses. [g] QTAIM topological electron density [*ρ*(r)], Laplacian [∇^2^ *ρ*(r)], electronic energy density [*H*(r)], and ellipticity [*ϵ(r)*] bond critical point data.

The computed MDC An atomic charges for **5′**–**12** are typical of An^IV^‐Tren complexes,[^11^, ^13^] and the An charges are consistently higher for **9**–**12** compared to **5′**–**8′** reflecting the presence of the HPn^2−^ donors in the latter compared to the H_2_Pn^1−^ donors in the former. We also note that the Pn centres carry approximately double the negative charge for the formal HPn^2−^ dianions (av.≈−0.86) compared to the HPn^1−^ monoanions (av.≈−0.4). For comparison, the av. N_amide_ and N_amine_ MDC atomic charges are computed to be ≈−0.85 and ≈−0.28, respectively, suggesting donation of charge from the Pn centres to An ions in‐line with their deviation from +4 in the wholly ionic extreme bonding picture.

The calculated Mayer bond orders for **5′**–**12** reflect the formal double and single An−Pn interactions in **5′**–**8′** and **9**–**12**, spanning the ranges 1.38–1.78 and 0.73–0.87, respectively. In‐line with the aforementioned points about the bonding of Th being more ionic than that of U, for a given An=P and An=As pair of **5′**–**8′** the U bond always has a larger bond order than for Th, and for Th=Pn and U=Pn it is always the phosphinidene that has the larger bond order. Thus, whilst still Th‐stabilised, **6** has the most polarised linkage of the An=Pn double bonds. The U>Th and P>As bond order pattern is also found for **9**–**12**, though the differences are smaller for these formal single bonds.

The Kohn Sham molecular orbitals (KSMOs) of **5′**–**8′** (Figure S75–78) and **9**–**12** (Figure S79–82) reveal An−Pn interactions that are dominated by P 3p‐ or As 4p‐character along with variable mixtures of 7s‐, 5f‐, and 6d‐orbital An‐character, where Th and U deploy more/less 6d/5f and 5f/6d contributions, respectively. For **5′**–**8′** in each case the An=Pn π‐bond is higher in energy than the σ‐bond, but the energy separations are small (0.058, 0.076, 0.076, and 0.074 eV, respectively). For **9**–**12** the An−Pn single bonds are constructed predominantly from a P 3p‐ or As 4p‐orbital that constitutes a σ‐bonding interaction and pseudo lone pair, with the An contributions following a similar pattern to **5′**–**8′**. Although the An−Pn interactions in **5′**–**8′** and **9**–**12** are reasonably well isolated in their respective KSMOs, they all contain intruding orbital coefficients from other atoms, most prominently nitrogen lone pairs. Therefore, to probe these An−Pn bonding interactions in a chemically clearer way we turned to the Natural Bond Orbital (NBO) approach.

The NBO data show that the bonding of **5′**/**6′** and **7′**/**8′** are quite similar for a given pair where the metal is the same, showing little variation due to the Pn‐element. However, when the metal is varied a consistent pattern arises of greater U contributions to the An=Pn bonds than for Th (12 and 12–15 % increases for σ‐ and π‐bonds from Th to U, respectively). Interestingly, the An contributions to the An=Pn bonds are always higher for the π‐ than the σ‐components for **5′**–**8′**, whereas analogous data for Tren^TIPS^ derivatives were more equally balanced. The Th bonding is weighted in favour of 6d character, but 5f contributions remain substantial (≈40 %), but for U 5f character dominates and more decisively overall (≈75 and 70 % for σ‐ and π‐bonds, respectively). Where **9**–**12** are concerned, the U complexes **11**/**12** exhibit greater (≈15 %) contributions to the bonding than the Th **9**/**10** analogues (≈9 %) and the same 6d vs 5f weightings are found.

To analyse the bonding of **5′**–**8′** and **9**–**12** from a different perspective, we examined the Quantum Theory of Atoms in Molecules (QTAIM) approach providing an electron topology rather than KSMO‐based approach. The data clearly reveal rather polar‐covalent An−Pn bonding interactions but consistently suggest the trend of more covalent U=Pn/U−Pn than Th=Pn/Th−Pn bonds. The ellipticity data confirm the double and single bonds of **5′**–**8′** (0.2–0.29) and **9**–**12** (0.03–0.05), noting that C−C ellipticity values for ethane, benzene, ethene, and acetylene are 0, 0.25, 0.45, and 0, respectively.^[29]^

Since the principal aim of this work was to secure a previously elusive terminal Th=AsH linkage where before only a capped version could be stabilised, isolated, and characterised, it is instructive to examine the differences between the bridging and terminal Th=AsH linkages of **A** and **6**. As suggested by the solid‐state and computational metrical data, removal of the {K(15C5)}^+^ unit results in a shorter Th=As distance and also a more fully developed Th=As double bond. This is reflected by the computed charges, which are lower for the Th and As atoms in **6** than **A**, and also the changes in NBO data; on moving from **A** to **6** the Th contribution to the σ‐bond increases by 5 % and the π‐bond increases by 12 %. These are increases by factors of 1.5 and 2.3, respectively, and it is interesting to note that whilst the σ‐bond responds to the removal of {K(15C5)}^+^ it is clearly the π‐bond that is the most responsive, and hence most perturbed. Consistent with this latter point, the 6d : 5f weighting of the Th=As σ‐bond changes little from **A** to **6**, but the π‐components change markedly, with the dominant 5 f component (5f : 6d=63 : 37 %) of **A** becoming reversed for **6** (5f : 6d=37 : 63 %). Thus, even though the {K(15C5)}^+^ fragment can be considered to be bound to the Th=AsH unit in **A** with a highly electrostatic bonding interaction, which might not be expected to necessarily result in substantial bonding changes when removed, it is evident that removal of the {K(15C5)}^+^ unit indeed results in a significant impact on the Th=AsH bond in terms of a better developed Th=As bond and also a substantial change to the character of the π‐component. Lastly, it is instructive to examine the impact that coordination of (AsH)^2−^ to Th has on the As atom. Since the AsH unit binds orthogonally, it is an orbital of pure 4p‐character that binds to Th to form the Th−As σ‐bond. In this process, the 4p‐character drops from 100 to 75 %, with the induced 25 % 4 s character being drawn from the As “σ‐lone pair”, whose 4s‐character concomitantly falls from 88 % in (AsH)^2−^ to 61 % in the non‐bonding “σ‐lone pair” of **6′**. The As π‐bond retains its 100 % As 4p‐character regardless of being free or coordinated. We conclude that the bridging (AsH)^2−^ unit in **A** is closer in character to the free (AsH)^2−^ dianion than the terminal (AsH)^2−^ moiety in **6**.

## Conclusion

To conclude, we have synthesised the new ligand Tren^TCHS^ which is even more sterically demanding than the Tren^TIPS^ ligand. This has enabled us to directly prepare and isolate a complete series of thorium and uranium terminal parent pnictinidene complexes, including the first example of a terminal thorium arsinidene complex of any kind. The synthesis of these complexes has permitted us to examine the bonding of these An−Pn bonds computationally and meaningfully probe the impact on the parent Th=AsH linkage when it is converted from an electrostatically bridged to terminal form. This has revealed a modest response in the σ‐bond but a much larger response in the π‐bond, and overall a modest change in Th=As distance but a large electronic response to the nature of the Th=AsH bonding on removal of the electrostatically‐bound capping group. The straightforward synthesis of this library of multiply bonded An‐complexes suggests that Tren^TCHS^ could provide access to new examples of unusual An‐L multiple bonds.

## Conflict of interest

The authors declare no conflict of interest.

1

## Supporting information

As a service to our authors and readers, this journal provides supporting information supplied by the authors. Such materials are peer reviewed and may be re‐organized for online delivery, but are not copy‐edited or typeset. Technical support issues arising from supporting information (other than missing files) should be addressed to the authors.

Supporting Information

Supporting Information

Supporting Information

## Data Availability

The data that support the findings of this study are available in the supplementary material of this article.
