# Supplementary material for: Actinide Pnictinidene Chemistry: A Terminal Thorium Parent‐Arsinidene Complex Stabilised by a Super‐Bulky Triamidoamine Ligand
Source: Angew Chem Int Ed Engl. 2022 Nov 16;61(50):e202211627. doi: 10.1002/anie.202211627 (PMC10099757; doi:10.1002/anie.202211627)
Supplement: Supplementary file 2 — Supporting Information [file ANIE-61-0-s002.pdf]

Angewandte Chemie
Eine Zeitschrift der Gesellschaft Deutscher Chemiker

Supporting Information

Actinide Pnictinidene Chemistry: A Terminal Thorium Parent-Arsinidene Complex Stabilised by a Super-Bulky Triamidoamine Ligand

J. Du, G. Balázs, J. A. Seed, J. D. Cryer, A. J. Wooles, M. Scheer, S. T. Liddle**

Experimental

General Considerations

All manipulations were carried out using Schlenk techniques, or an MBraun UniLab glovebox, under an atmosphere of dry nitrogen. Solvents were dried by passage through activated alumina towers and degassed before use. All solvents were stored over potassium mirrors except for ethers, which were stored over activated 4 Å sieves. Deuterated solvent was distilled from potassium, degassed by three freeze-pump-thaw cycles and stored under dinitrogen. Tris(2-aminoethyl)amine was distilled prior to use. Cy_2SiCl_2, ^nBuLi (2.5 M in hexanes), and Me_3SiCH_2Li (1.0 M in pentane) were used as purchased. Triethylammonium tetraphenylborate $[HNEt_3][BPh_4]$,[1] CyLi,[2] $NaPH_2$,[3] $KAsH_2$,[4] KC_8,[5] $ThCl_4(THF)_{3.5}$,[6] and UCl_4[7] were prepared using literature methods. $Th(NO_3)_4(H_2O)_x$ was purchased from Strem Chemicals Inc. and depleted UO_3 was supplied by the National Nuclear Laboratory.

Single crystals were examined variously on a) a Agilent Supernova diffractometer, equipped with an Eos CCD area detector and a Microfocus source with Mo Kα radiation (λ = 0.71073 Å), b) a Rigaku Rigaku XtalLAB Synergy-S diffractometer, equipped with a HyPix 6000HE photon counting pixel array detector with mirror-monochromated Mo Kα (λ = 1.54184 Å) radiation or c) a Rigaku FR-X diffractometer equipped with a Hypix 6000HE photon counting pixel array detector with mirror-monochromated MoKα (λ = 0.71073 Å) or CuKα (λ = 1.5418 Å) radiation. Intensities were integrated from a sphere of data recorded on narrow (0.5 or 1.0°) frames by ω rotation. Cell parameters were refined from the observed positions of all strong reflections in each data set. Gaussian grid face-indexed absorption corrections with a beam profile correction were applied. The structures were solved by dual methods using SHELXT[8] and all non-hydrogen atoms were refined by full-matrix least-squares on all unique F^2 values with anisotropic displacement parameters with exceptions noted in the respective cif files. Except where noted hydrogen atoms were refined with constrained geometries and riding thermal parameters; U_{iso}(H) was set at 1.2 (1.5 for methyl groups) times U_{eq} of the parent atom. The largest features in final difference syntheses were close to heavy atoms and were

of no chemical significance. Some of the structures exhibit disorder, this is either cyclohexyl groups of the $Tren^{TCHS}$ ligand, lattice solvent, or the cryptand counterion component. CrysAlisPro was used for control and integration,[9] and SHELXL and Olex2 were employed for structure refinement.[10,11] ORTEP-3 and POV-Ray were employed for molecular graphics.[12,13] ^{1}H, $^{13}C\{^{1}H\}$, $^{29}Si\{^{1}H\}$, $^{7}Li\{^{1}H\}$, and ^{31}P NMR spectra were recorded on a Bruker 400 spectrometer operating at 400, 101, 79, 155, and 162 MHz, respectively; chemical shifts are quoted in ppm and are relative to TMS (^{1}H, ^{13}C, and ^{29}Si), LiCl (^{7}Li) and 85% H_3PO_4 (^{31}P). ATR-IR spectra were recorded on a Bruker Alpha spectrometer with a Platinum-ATR module in the glovebox. Raman spectra were recorded on a Horiba XploRA Plus Raman microscope using a 638 nm laser with a power of 1.5 mW. The power was adjusted using a power filter for each complex to inhibit sample decomposition. UV/Vis/NIR spectra were recorded on a Perkin Elmer L M D™ 750 spectrometer. Data were collected in a 1mm path-length cuvette loaded in an MBraun glovebox and were run versus the appropriate solvent. Static variable-temperature magnetic moment data were recorded in an applied dc field of 0.5 T on a Quantum Design MPMS3 superconducting quantum interference device (SQUID) magnetometer using doubly recrystallised powdered samples. Care was taken to ensure complete thermalisation of the sample before each data point was measured, samples were immobilised in eicosane to prevent sample reorientation, diamagnetic corrections were applied using tabulated Pascal constants, and measurements were corrected for the effect of the eicosane and blank sample holders. CHN microanalyses were carried out by Martin Jennings and Anne Davies at the University of Manchester.

Preparation of Cy_3SiCl

Cy_2SiCl_2 (26.53 g, 100.00 mmol) was added to a cold slurry of CyLi (9.00 g, 100.00 mmol) at –78 °C in toluene (200 mL). After addition, the white slurry was allowed to warm to room temperature whilst stirring and stirred for further 24 h. The white slurry was filtered through a frit with Celite® to remove the LiCl by-product. Volatiles were removed, and the white product was further dried *in vacuo* to give Cy_3SiCl as white solid, which is pure enough for the next step of the synthesis for

[(Tren^{TCHS})Li_3] without further purification. Yield: 28.80 g, 92%. The product was confirmed by XRD cell check and NMR spectroscopic studies, which are in agreement with reported data.[14]

Preparation of [(Tren^{TCHS})Li_3]

$N(CH_2CH_2NH_2)_3$ (2.93 g, 20.00 mmol) was dissolved in THF (200 mL). At –78 °C, ^{n}BuLi (2.5 M, 24.00 mL, 60.00 mmol) was added in a dropwise manner and the mixture was stirred for 4 hours at room temperature after addition. The white slurry was then cooled to –78 °C again, and the solution of Cy_3SiCl (18.78 g, 60.00 mmol) in toluene (100 mL) was added. The mixture was allowed to warm up to room temperature and stirred further for 16 hours. Volatiles were removed *in vacuo* and the product was extracted with warm toluene (60 °C, 200 mL). The slurry was filtered through a frit with Celite® to remove the LiCl precipitate and then the yellow filtrate was cooled to –78 °C again, and n-BuLi (2.5 M, 24.00 mL, 60.00 mmol) was added dropwise and the mixture was stirred for further 16 hours at room temperature, resulting in [(Tren^{TCHS})Li_3] precipitating out due to its poor suability in aromatic solvent. The white product was collected in a frit by filtration and washed with pentane (50 mL), and then dried *in vacuo* to yield [(Tren^{TCHS})Li_3] as a white powder. Colourless crystals of [(Tren^{TCHS})Li_3] were grown from a concentrated solution in C_6D_6 at room temperature. Yield: 11.12 g, 56%. Anal. Calcd for $C_{60}H_{111}Li_3N_4Si_3$: C, 72.53; H, 11.26; N, 5.64%. Found: C, 70.25; H, 11.16; N, 5.31%. The low C value is attributed to incomplete combustion and SiC formation, which is often observed in the Si-containing ligand systems.[15] ^{1}H NMR (C_6D_6, 298 K): δ 1.38-1.50 (m, 54H, Cy-substituents), 1.88-1.93 (m, 45H, Cy-substituents), 2.35 (t, $^{3}J_{HH}$ = 5.3 Hz, 6H, CH_2CH$_2$), 3.18 (t, $^{3}J_{HH}$ = 5.3 Hz, 6H, CH$_2$CH_2) ppm. ^{29}Si NMR (C_6D_6, 298 K): δ –3.14 ppm. ^{7}Li NMR (C_6D_6, 298 K): δ –2.13 ppm. ATR-IR ν/cm^{-1}: 2913 (vs), 2844 (s), 1444 (m), 1343 (w), 1268 (w), 1191 (w), 1164 (w), 1058 (m), 1030 (m), 997 (m), 939 (s), 888 (m), 846 (m), 816 (m), 770 (s), 733 (m), 584 (w), 526 (s), 472 (m). Attempts to record reliable ^{13}C{^{1}H} NMR data were precluded by the poor solubility of this compound in common aromatic and ethereal solvents; indeed, several of the following complexes are poorly soluble once isolated, which variously precluded acquisition of NMR and optical data.

Preparation of [Th(Tren^{TCHS})(Cl)] (1)

A solution of [(Tren^{TCHS})Li_3] (5.00 g, 5.00 mmol) in THF (50 mL) was added in a dropwise manner to a pre-cold stirring solution of $ThCl_4(THF)_{3.5}$ (3.13 g, 5.00 mmol) in THF (50 mL) at –78 °C. The mixture was allowed to warm to room temperature with stirring over 16 h, during this time, the product precipitated out as a white solid. The reaction mixture was concentrated to nearly half-volume (50 mL) and filtered to remove the LiCl by-product in the yellow solution phase in THF. The product was further washed with Et_2O (2 × 20 mL) to yield **1** as a white solid. Colourless crystals of **1** were obtained by cooling a hot solution in toluene (100 °C) to room temperature. Yield: 3.91 g, 63%. Anal. Calcd for $C_{60}H_{111}ClN_4Si_3Th$: C, 58.10; H, 9.02; N, 4.52%. Found: C, 58.18; H, 9.11; N, 4.47%. ^{1}H NMR (C_6D_6, 298 K): δ 1.30-1.63 (m, 54H, Cy-substituents), 1.84-2.01 (m, 45H, Cy-substituents), 2.68 (t, $^3J_{HH}$ = 4.6 Hz, 6H, CH_2CH$_2$), 3.70 (t, $^3J_{HH}$ = 4.6 Hz, 6H, CH$_2$CH_2) ppm. ^{29}Si NMR (C_6D_6, 298 K): δ –2.43 ppm. ATR-IR ν/cm^{-1}: 2915 (s), 2844 (m), 1444 (m), 1275 (w), 1203 (w), 1164 (w), 1107 (w), 1042 (w), 998 (m), 925 (m), 889 (m), 841 (m), 814 (w), 742 (vs), 721 (s), 699 (m), 681 (m), 556 (w), 485 (w), 442 (w).

Preparation of [U(Tren^{TCHS})(Cl)] (2)

A solution of [(Tren^{TCHS})Li_3] (5.00 g, 5.00 mmol) in THF (50 mL) was added in a dropwise manner to a pre-cold stirring solution of UCl_4 (1.90 g, 5.00 mmol) in THF (50 mL) at –78 °C. The mixture was allowed to warm to room temperature with stirring over 16 h, during this time, the product precipitated out as a green solid. The reaction was concentrated to nearly half-volume (50 mL) and the mixture was filtered to remove the LiCl by-product in the dark brown solution phase in THF. The product was further washed with Et_2O (2 × 20 mL) followed by hexane (20 mL) to yield **2** as a green solid. Green crystals of **2** were obtained by cooling a hot solution in toluene (100 °C) to room temperature or from the washings in hexane. Yield: 4.61 g, 74%. Anal. Calcd for $C_{60}H_{111}ClN_4Si_3U$: C, 57.82; H, 8.98; N, 4.50%. Found: C, 58.15; H, 9.12; N, 4.36%. ^{1}H NMR (C_6D_6, 298 K): δ –39.15

(br), –0.04 (br), 1.29 (br), 2.26 (br), 3.36 (br), 3.44 (br), 6.13 (br), 8.85 (br), 11.78 (br), 12.09 (br) ppm. ^{29}Si NMR (C_6D_6, 298 K): δ 15.26 ppm. ATR-IR ν/cm^{-1}: 2914 (s), 2844 (m), 1443 (m), 1333 (w), 1274 (w), 1202 (w), 1164 (w), 1107 (w), 1038 (w), 998 (w), 924 (m), 888 (m), 842 (w), 814 (w), 737 (vs), 717 (vs), 694 (s), 677 (s), 557 (m), 459 (w).

Preparation of [Th{N(CH₂CH₂NSiCy₃)₂(CH₂CH₂NSiCy₂[CHCH₂CH₂CH₂CH₂CH])}] (3)

Toluene (100 mL) was added to a cold pre-cold mixture of **1** (2.48 g, 2.00 mmol) and Me_3SiCH_2Li (2.10 g, 2.2 mmol) at –78 °C. The white slurry was allowed to warm to room temperature whilst stirring and stirred for further 24 h. The reaction was heated up to 100 °C and filtered from LiCl, and then volatiles were removed *in vacuo* to give a pale yellow solid which was washed with pentane (2 × 30 mL) and dried *in vacuo* to yield **3** as white solid. Colourless crystals of **3** were obtained by cooling a hot solution in toluene (100 °C) to room temperature. Yield: 1.50 g, 62%. Anal. Calcd for $C_{60}H_{110}N_4Si_3Th$(pentane): C, 61.45; H, 9.69; N, 4.34%. Found: C, 61.30; H, 9.33; N, 4.44%. ATR-IR ν/cm^{-1}: 2912 (s), 2843 (s), 1444 (m), 1342 (w), 1269 (w), 1089 (m), 995 (w), 926 (m), 887 (w), 842 (w), 816 (w), 751 (vs), 725 (s), 531 (w), 523 (w), 450 (w).

Preparation of [U{N(CH₂CH₂NSiCy₃)₂(CH₂CH₂NSiCy₂[CHCH₂CH₂CH₂CH₂CH])}] (4)

Toluene (100 mL) was added to a cold pre-cold mixture of **2** (2.50 g, 2.0 mmol) and Me_3SiCH_2Li (2.10 g, 2.2 mmol) at –78 °C. The green slurry was allowed to warm to room temperature whilst stirring and stirred for further 24 h. The reaction was heated up to 100 °C and filtered from LiCl, and then volatiles were removed *in vacuo* to give the red solid which was washed with pentane (2 × 30 mL) and dried *in vacuo* to yield **4** as red solid. Red crystals of **4** were obtained by cooling a hot solution in toluene (100 °C) to room temperature. Yield: 1.65 g, 68%. Anal. Calcd for $C_{60}H_{110}N_4Si_3U$(toluene): C, 61.81; H, 9.14; N, 4.30%. Found: C, 61.63; H, 9.38; N, 3.99%. ATR-IR

ν/cm^{-1}: 2910 (s), 2842 (s), 1443 (m), 1341 (w), 1269 (w), 1187 (w), 1168 (w), 1091 (w), 1051 (w), 995 (w), 921 (m), 888 (m), 842 (w), 816 (w), 746 (vs), 724 (s), 548 (wm, 523 (w), 450 (w).

Preparation of [Th(Tren^{TCHS})(PH)][Na(2,2,2-cryptand)] (5)

THF (20 mL) was added to a mixture of **3** (1.20 g, 1.00 mmol), $NaPH_2$ (0.06 g, 1.00 mmol), and 2,2,2-cryptand (0.38 g, 1.00 mmol) at room temperature. The white slurry was stirred for 2 hours, during this time, the mixture slowly turned into a yellow solution. Volatiles were removed *in vacuo* to give a yellow oily residue which was extracted with Et_2O (5 mL) and filtered. The yellow filtrate was stored at –35 °C for 3 days to give yellow crystals suitable for X-ray diffraction. Complex **5** was obtained as yellow crystalline solid by decanting the mother liquor and dried *in vacuo*. Yield: 0.85 g, 52%. Anal. Calcd for $C_{78}H_{148}N_6NaO_6PSi_3Th$: C, 57.25; H, 9.12; N, 5.14%. Found: C, 57.48; H, 9.35; N, 5.18%. ^{1}H NMR (D_8-THF, 298 K): δ –0.41 (d, $^2J_{PH}$ = 68.6 Hz, 1H, P*H*), 1.29-1.64 (m, 54H, Cy-substituents), 1.71-2.02 (m, 45H, Cy-substituents), 2.49 (t, $^3J_{HH}$ = 4.4 Hz, 6H, C*H*$_2$CH$_2$), 2.67 (t, $^3J_{HH}$ = 4.8 Hz, 12H, C*H*$_2$CH$_2$-cryptand), 3.46 (t, $^3J_{HH}$ = 4.4 Hz, 6H, CH$_2$C*H*$_2$), 3.59 (t, $^3J_{HH}$ = 4.8 Hz, 12H, CH$_2$C*H*$_2$-cryptand), 3.65 (s, 12H, C*H*$_2$C*H*$_2$-cryptand) ppm. ^{13}C{^{1}H} NMR (D_8-THF, 298 K): δ 68.76, 67.93, 63.36, 53.30, 45.07, 29.56, 29.02, 27.78, 25.86 ppm. ^{29}Si NMR (D_8-THF, 298 K): δ –4.92 ppm. ^{31}P NMR (D_8-THF, 298 K): δ 266.16 (d, J_{PH} = 68.6 Hz, *P*H) ppm. ATR-IR ν/cm^{-1}: 2910 (s), 2840 (s), 2072 (br), 1443 (m), 1356 (w), 1299 (w), 1276 (w), 1104 (s), 1054 (s), 1022 (w), 998 (w), 932 (s), 888 (w), 841 (w), 820 (w), 745 (vs), 700 (s), 549 (w), 522 (m), 484 (w), 455 (w). Raman ν/cm^{-1}: 2926 (s), 2845(s), 1440 (s), 1270 (w), 1184 (m), 1024 (w), 841 (s), 814 (w), 728 (s), 678 (w), 296 (s), 206 (m), 75 (s).

Preparation of [Th(Tren^{TCHS})(AsH)][K(2,2,2-cryptand)] (6)

THF (20 mL) was added to a mixture of **3** (1.20 g, 1.00 mmol), $KAsH_2$ (0.12 g, 1.00 mmol), and 2,2,2-cryptand (0.38 g, 1.00 mmol) at room temperature. The white slurry was stirred for 2 hours,

during which time, the mixture slowly turned into an orange solution. Volatiles were removed *in vacuo* to give an orange oily residue which was extracted with THF (4 ml) and filtered. The orange filtrate was stored at –35 °C for 2 days to give orange crystals suitable for X-ray diffraction. Complex **6** was obtained as orange crystalline solid by decanting the mother liquor and dried *in vacuo*. Yield: 0.78 g, 46%. Anal. Calcd for $C_{78}H_{148}N_6KO_6AsSi_3Th$: C, 55.23; H, 8.79; N, 4.95%. Found: C, 55.67; H, 8.86; N, 5.01%. ^{1}H NMR (D_8-THF, 298 K): δ –1.55 (s, 1H, As*H*), 1.29-2.03 (m, 99H, Cy-substituents), 2.49 (t, $^3J_{HH}$ = 4.4 Hz, 6H, CH_2CH$_2$), 2.58 (t, $^3J_{HH}$ = 4.0 Hz, 12H, CH$_2$CH_2-cryptand), 3.46 (t, $^3J_{HH}$ = 4.4 Hz, 6H, CH$_2$CH_2), 3.62 (t, $^3J_{HH}$ = 4.0 Hz, 12H, CH$_2$CH_2-cryptand), 3.64 (s, 12H, CH_2CH_2-cryptand) ppm. ^{13}C{^{1}H} NMR (D_8-THF, 298 K): δ 70.59, 67.75, 63.56, 54.07, 45.00, 29.60, 29.03, 27.80, 25.95 ppm. ^{29}Si NMR (D_8-THF, 298 K): δ –5.13 ppm. ATR-IR ν/cm^{-1}: 2907 (s), 2838 (s), 1867 (br), 1442 (m), 1355 (w), 1295 (w), 1259 (w), 1133 (s), 1103 (m), 1082 (m), 998 (w), 930 (s), 888 (w), 842 (w), 820 (w), 748 (vs), 725 (s), 551 (w), 522 (m), 482 (w), 452 (w), 438 (w). Raman ν/cm^{-1}: 2925 (s), 2845 (s), 1440 (s), 1340 (m), 1288 (m), 1274 (m), 1184 (w), 1204 (m), 912 (s), 815 (m), 723 (w), 681 (w), 436 (w), 257 (w), 197 (s), 60 (s).

Preparation of [U(Tren^{TCHS})(PH)][Na(2,2,2-cryptand)] (7)

THF (20 mL) was added to a mixture of **4** (1.21 g, 1.00 mmol), $NaPH_2$ (0.06 g, 1.00 mmol), and 2,2,2-cryptand (0.38 g, 1.00 mmol) at room temperature. The red slurry was stirred for 2 hours, during which time, the mixture slowly turned into a dark green solution. Volatiles were removed *in vacuo* to give a dark green oily residue which was extracted with Et_2O (5 mL) and filtered. The dark green filtrate was stored at –35 °C for 3 days to give dark green crystals suitable for X-ray diffraction. Complex **7** was obtained as dark green crystalline solid by decanting the mother liquor and dried *in vacuo*. Yield: 0.79 g, 48%. Anal. Calcd for $C_{78}H_{148}N_6NaO_6PSi_3U(Et_2O)$: C, 57.38; H, 9.28; N, 4.90%. Found: C, 57.98; H, 9.54; N, 5.33%. ^{1}H NMR (D_8-THF, 298 K): δ –13.41 (br), –11.76 (br), –10.00 (br), –2.84 (br), –2.81 (br), –1.67 (br), –1.64 (br), 0.03 (br), 0.07 (br), 0.60 (br), 0.63 (br), 1.71 (br), 7.14 (br), 8.34 (br), 8.36 (br), 8.37 (br), 9.27 (br), 9.28 (br), 9.30 (br), 9.50 (br), 51.91 (br), 190.90

(br, P*H*) ppm. ^{29}Si NMR (D$_8$-THF, 298 K): δ –130.15 ppm. ^{31}P NMR (D$_8$-THF, 298 K): δ 2628.50 (br, *P*H) ppm. ATR-IR ν/cm^{-1}: 2906 (s), 2839 (s), 2070 (br), 1443 (m), 1355 (w), 1299 (w), 1275 (w), 1130 (s), 1103 (s), 932 (s), 890 (m), 841 (w), 819 (w), 742 (vs), 725 (s), 532 (m), 484 (w), 454 (w). Raman ν/cm^{-1}: 2930 (s), 2847 (s), 1442 (s), 1343 (w), 1272 (m), 1186 (w), 1025 (s), 843 (w), 812 (s), 679 (w), 437 (w), 305 (w), 208 (m), 62 (s).

Preparation of [U(Tren^{TCHS})(AsH)][K(2,2,2-cryptand)] (8)

THF (20 mL) was added to a mixture of **4** (1.21 g, 1.00 mmol), $KAsH_2$ (0.12 g, 1.00 mmol), and 2,2,2-cryptand (0.38 g, 1.00 mmol) at room temperature. The red slurry was stirred for 2 hours, during which time, the mixture slowly turned into a dark green solution. Volatiles were removed *in vacuo* to give a dark green oily residue which was extracted with THF (4 mL) and filtered. The yellow filtrate was stored at –35 °C for 3 days to give dark green crystals suitable for X-ray diffraction. Complex **8** was obtained as orange crystalline solid by decanting the mother liquor and dried *in vacuo*. Yield: 0.87 g, 51%. Anal. Calcd for $C_{78}H_{148}N_6KO_6AsSi_3U$: C, 55.03; H, 8.76; N, 4.94%. Found: C, 55.36; H, 8.78; N, 4.76%. ^{1}H NMR (D$_8$-THF, 298 K): δ –8.71 (br), –7.63 (br), –6.84 (br), –1.65 (br), –1.64 (br), –1.61 (br), –0.81 (br), –0.78 (br), 0.39 (br), 0.43 (br), 0.46 (br), 0.50 (br), 1.72 (br), 5.12 (br), 5.14 (br), 5.15 (br), 5.20 (br), 6.28 (br), 6.29 (br), 6.30 (br), 6.46 (br), 36.74 (br), 123.57 (br, As*H)* ppm. ^{29}Si NMR (D$_8$-THF, 298 K): δ –116.10 ppm. ATR-IR ν/cm^{-1}: 2908 (s), 2839 (s), 1875 (br), 1443 (m), 1353 (w), 1293 (w), 1259 (w), 1103 (s), 1046 (m), 930 (s), 888 (m), 841 (w), 818 (w), 799 (vs), 741 (s), 521 (m), 484 (w), 456 (w). Raman ν/cm^{-1}: 2908 (m), 2847 (m), 1442 (m), 1026 (w), 910 (w, br), 809 (w), 475 (m, br), 202 (m), 65 (m).

Preparation of [Th(Tren^{TCHS})(PH_2)] (9)

A solution of $[HNEt_3][BPh_4]$ (0.09, 0.2 mmol) in THF (1 mL) was added to a solution of **5** (0.33 g, 0.2 mmol) in benzene (10 mL) at room temperature. The yellow solution instantaneously turned into

a colourless slurry upon combining above two solutions and the mixture was stirred for 10 min and then filtered to remove the white precipitate of [Na(2,2,2-cryptand)][BPh_4] by-product. Removal of the volatiles from the resulting colourless solution afforded an off-white solid, which was washed with Et_2O (2 × 5 mL) and dried *in vacuo* to give **9** as white solid. Yield: 0.16 g, 65%. Colourless crystals suitable for X-ray diffraction were obtained by cooling a hot solution in benzene (80 °C) at room temperature as **9** is poorly soluble in benzene at room temperature. Anal. Calcd for $C_{60}H_{113}N_4PSi_3Th$: C, 58.22; H, 9.20; N, 4.53%. Found: C, 58.23; H, 9.52; N, 4.23%. ^{1}H NMR (D_8-THF, 298 K): δ 0.75-2.31 (dd, J_{PH} = 153.2 Hz, 2H, PH_2), 1.13-1.86 (m, 99H, Cy-substituents), 2.92 (s, 6H, CH_2CH$_2$), 3.74 (s, 6H, CH$_2$CH_2) ppm. ^{13}C{^{1}H} NMR (D_8-THF, 298 K): δ 61.91, 44.08, 27.49, 26.68, 25.29, 22.88 ppm. ^{29}Si NMR (D_8-THF, 298 K): δ –3.62 ppm. ^{31}P NMR (D_8-THF, 298 K): δ –133.01 (t, J_{PH} = 153.2 Hz, PH$_2$) ppm. ATR-IR ν/cm^{-1}: 2913 (s), 2843 (s), 2262 (br), 1443 (m), 1333 (w), 1273 (w), 1210 (w), 1165 (w), 1040 (w), 996 (w), 925 (w), 888 (m), 841 (w), 814 (w), 740 (vs), 697 (m), 563 (m), 521 (w), 486 (w), 458 (w), 420 (w).

Preparation of [Th(Tren^{TCHS})(AsH_2)] (10)

A solution of [$HNEt_3$][BPh_4] (0.09, 0.2 mmol) in THF (1 mL) was added to a solution of **6** (0.34 g, 0.2 mmol) in THF (5 mL) at room temperature. The orange solution instantaneously turned into a yellow solution upon combining above two solutions. Removal of the volatiles from the resulting yellow solution afforded a brown solid, which was extracted with hot toluene (10 mL, 100 °C) and filtered from the [K(222-cryptand)][BPh_4] salt. Yellow crystals suitable for X-ray diffraction were obtained by cooling this hot filtrate to room temperature. Complex **10** was obtained as yellow crystalline solid by decanting the mother liquor and dried *in vacuo*. Yield: 0.18 g, 46%. Anal. Calcd for $C_{60}H_{113}N_4AsSi_3Th(Toluene)_{0.5}$: C, 57.44; H, 8.88; N, 4.22%. Found: C, 57.95; H, 8.78; N, 3.70%. ATR-IR ν/cm^{-1}: 2912 (s), 2842 (s), 2062 (br), 1442 (m), 1342 (w), 1273 (w), 1201 (w), 1164 (w), 1106 (w), 1039 (w), 996 (w), 925 (w), 887 (w), 841 (w), 815 (w), 741 (vs), 720 (s), 553 (w), 522 (m),

457 (w). Reliable NMR spectra were not obtained due to the very poor solubility in common solvents like benzene, toluene, and THF.

Preparation of [U(Tren^{TCHS})(PH$_2$)] (11)

A solution of [$HNEt_3$][BPh_4] (0.09, 0.2 mmol) in THF (1 mL) was added to a solution of **7** (0.33 g, 0.2 mmol) in benzene (10 mL) at room temperature. The dark green solution instantaneously turned into a brown slurry upon combining above two solutions and the mixture was stirred for 10 min and then filtered to remove the white precipitate of [Na(2,2,2-cryptand)][BPh_4] by-product. Removal of the volatiles from the resulting brown solution afforded a brown solid, which was washed with Et_2O (2 × 5 mL) and dried *in vacuo* to give the **11** as a brown solid. Yield: 0.16 g, 63%. Dark brown crystals suitable for X-ray diffraction were obtained by cooling a hot solution in benzene (80 °C) at room temperature. $C_{60}H_{113}N_4PSi_3U$: C, 57.94; H, 9.16; N, 4.50%. Found: C, 57.63; H, 9.24; N, 4.17%. ^{1}H NMR (D_8-THF, 298 K): δ –39.31 (br), 0.79 (br), 2.58 (br), 3.65 (br), 4.23 (br), 10.30 (br), 11.74 (br), 12.96 (br), –134.83 to –134.44 (d, br, PH_2) ppm. ^{29}Si NMR (D_8-THF, 298 K): δ 31.53 ppm. ^{31}P NMR (D_8-THF, 298 K): δ 605.91 (t, J_{PH} = 150.5 Hz, PH_2) ppm. ATR-IR ν/cm^{-1}: 2913 (s), 2843 (s), 2259 (br), 1443 (m), 1262 (w), 1202 (w), 1165 (w), 1105 (w), 1068 (w), 996 (w), 925 (w), 887 (m), 841 (m), 820 (w), 736 (vs), 679 (m), 565 (m), 533 (m), 522 (m), 486 (w), 457 (w), 418 (w).

Preparation of [U(Tren^{TCHS})(AsH$_2$)] (12)

A solution of [$HNEt_3$][BPh_4] (0.09, 0.2 mmol) in THF (1 mL) was added to a solution of **8** (0.34 g, 0.2 mmol) in THF (5 mL) at room temperature. The dark green solution instantaneously turned into a red solution upon combining above two solutions. Storing the reaction in a glass vial at room temperature for 16 hours gave dark red crystals of **12** suitable for X-ray diffraction, and the [K(222-cryptand)][BPh_4] salt stays in solution phase. Complex **12** was obtained as red crystalline solid by decanting the mother liquor and dried *in vacuo*. Yield: 0.13 g, 48%. $C_{60}H_{113}N_4AsSi_3U(THF)_2$: C,

57.04; H, 9.08; N, 3.91%. Found: C, 58.03; H, 8.76; N, 3.64%. ATR-IR ν/cm^{-1}: 2913 (s), 2843 (s), 2064 (br), 1443 (m), 1332 (w), 1271 (w), 1255 (w), 1202 (w), 1165 (w), 1131 (w), 996 (w), 926 (w), 887 (m), 841 (m), 815 (w), 736 (vs), 717 (s), 550 (m), 533 (w), 522 (m), 457 (w), 418 (w).

Computational Details

General

Geometry optimisations for **9-12** and the anion components of **5-8** were performed using coordinates derived from their crystal structures as the starting points. Geometry optimisations for neutral triplet AsH and singlet $(AsH)^{2-}$ dianion were performed on an initial structure with an As-H bond distance of 1.53 Å. No constraints were imposed on the structures during the geometry optimisations, other than computing open-shell species with unrestricted calculations and closed-shell species with restricted calculations. The calculations were performed using the Amsterdam Density Functional (ADF) suite version 2017 with standard convergence criteria (Tables S3-S10).[16,17] The DFT geometry optimisations employed Slater type orbital (STO) triple-ζ-plus polarisation all-electron basis sets (from the Dirac and ZORA/TZP database of the ADF suite). Scalar relativistic approaches (spin-orbit neglected) were used within the ZORA Hamiltonian[18-20] for the inclusion of relativistic effects and the local density approximation (LDA) with the correlation potential due to Vosko *et al* used in all of the calculations.[21] Generalised gradient approximation corrections were performed using the functionals of Becke and Perdew.[22,23] Analytical frequency calculations were carried out using the ADF code. Natural Bond Order (NBO) analyses were carried out with NBO 6.0.19.[24] The Quantum Theory of Atoms in Molecules analysis[25,26] was carried out within the ADF program. The ADF-GUI (ADFview) was used to prepare the three-dimensional plots of the electron density.

Figures

Molecular Structures

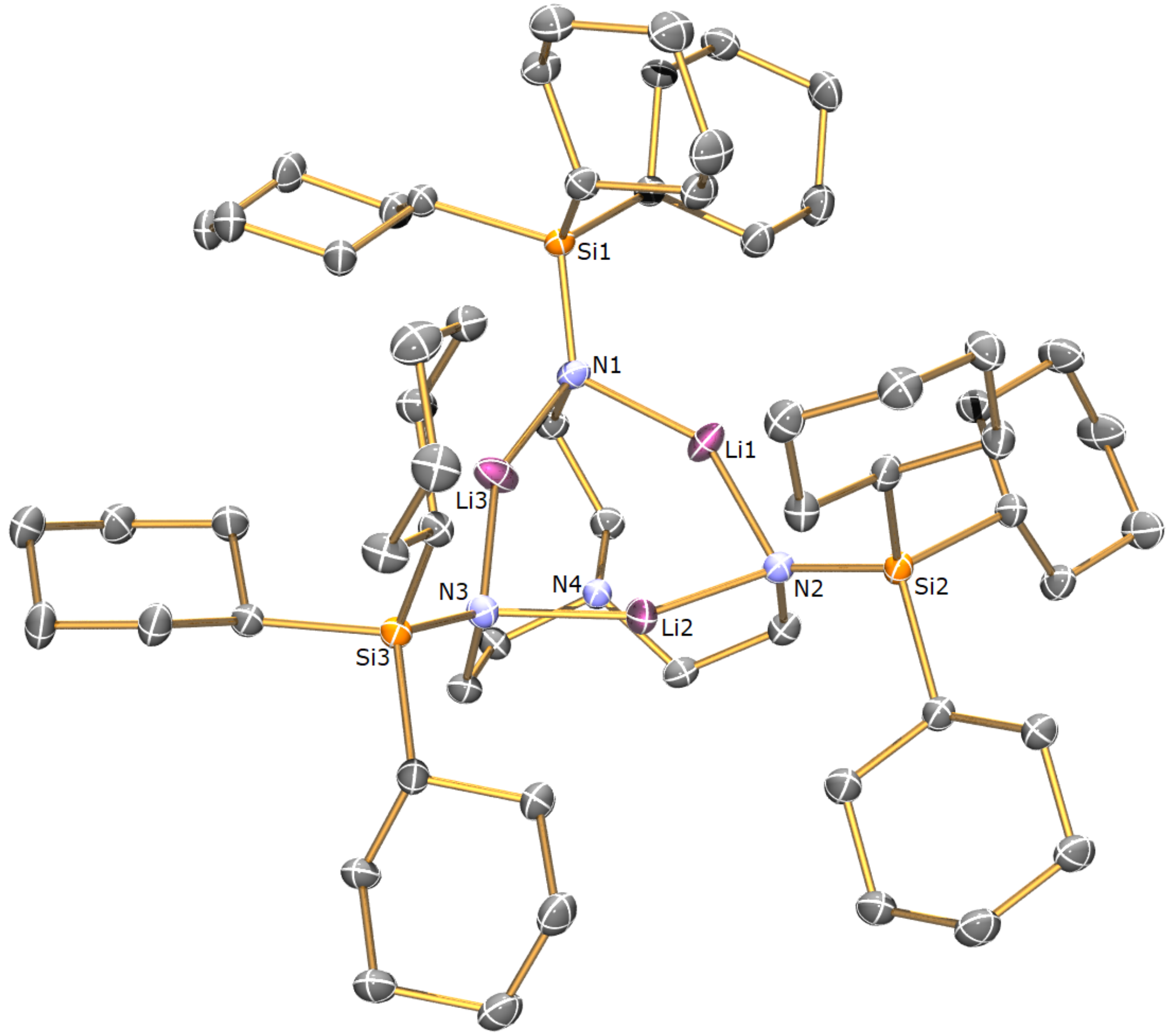

Figure S1. Molecular structure of [Tren^{TCHS}Li$_3$] at 150 K. Displacement ellipsoids are set at 40% and hydrogen atoms, and benzene molecules in the lattice are omitted for clarity.

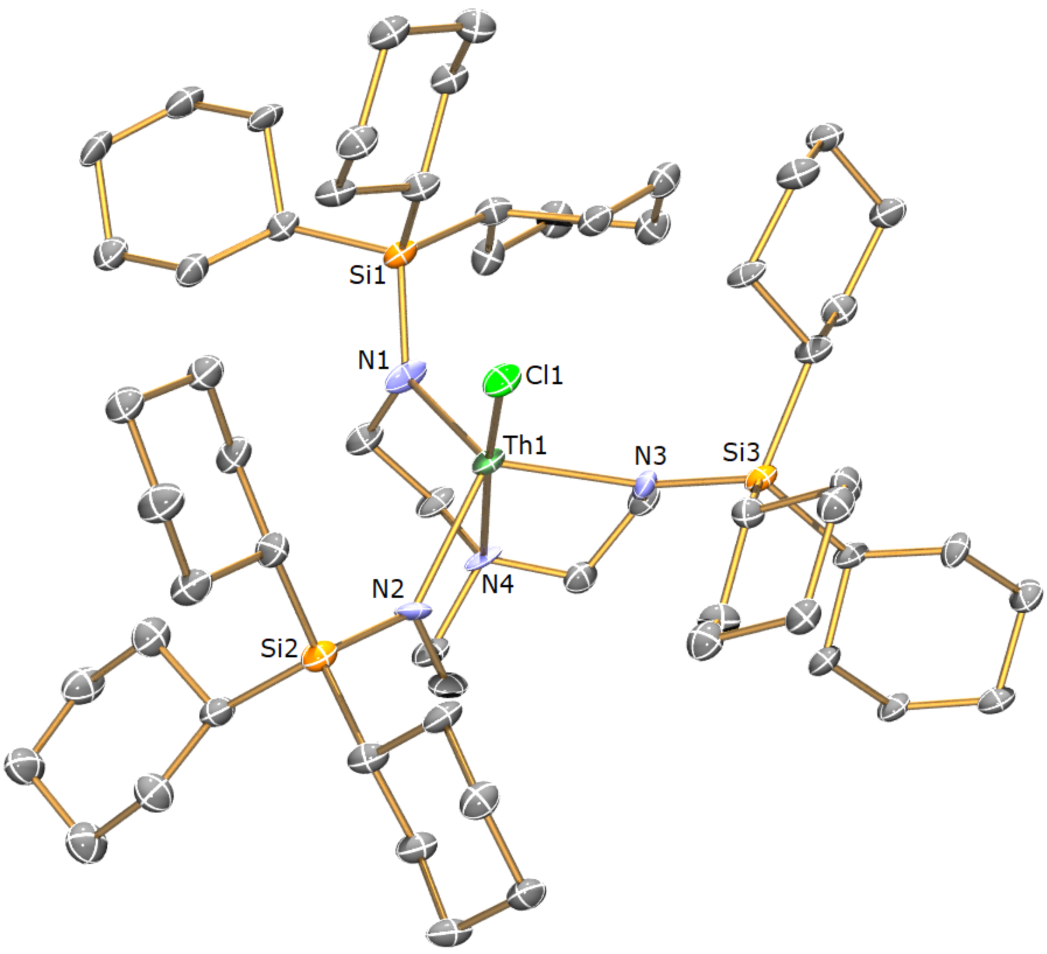

Figure S2. Molecular structure of **1** at 150 K. Displacement ellipsoids are set at 40% and hydrogen atoms, and toluene molecules in the lattice are omitted for clarity.

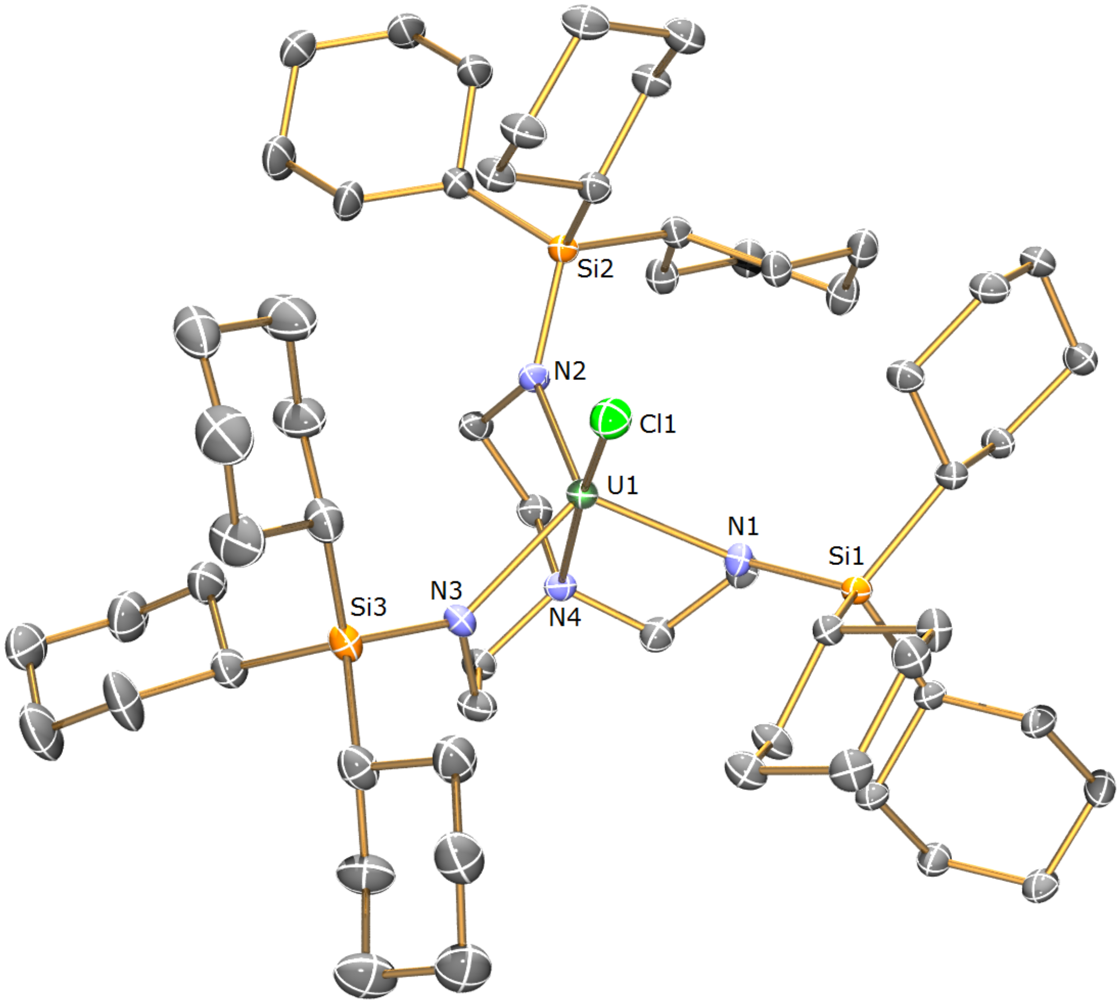

Figure S3. Molecular structure of **2** at 150 K. Displacement ellipsoids are set at 40% and hydrogen atoms, and hexane molecules in the lattice are omitted for clarity.

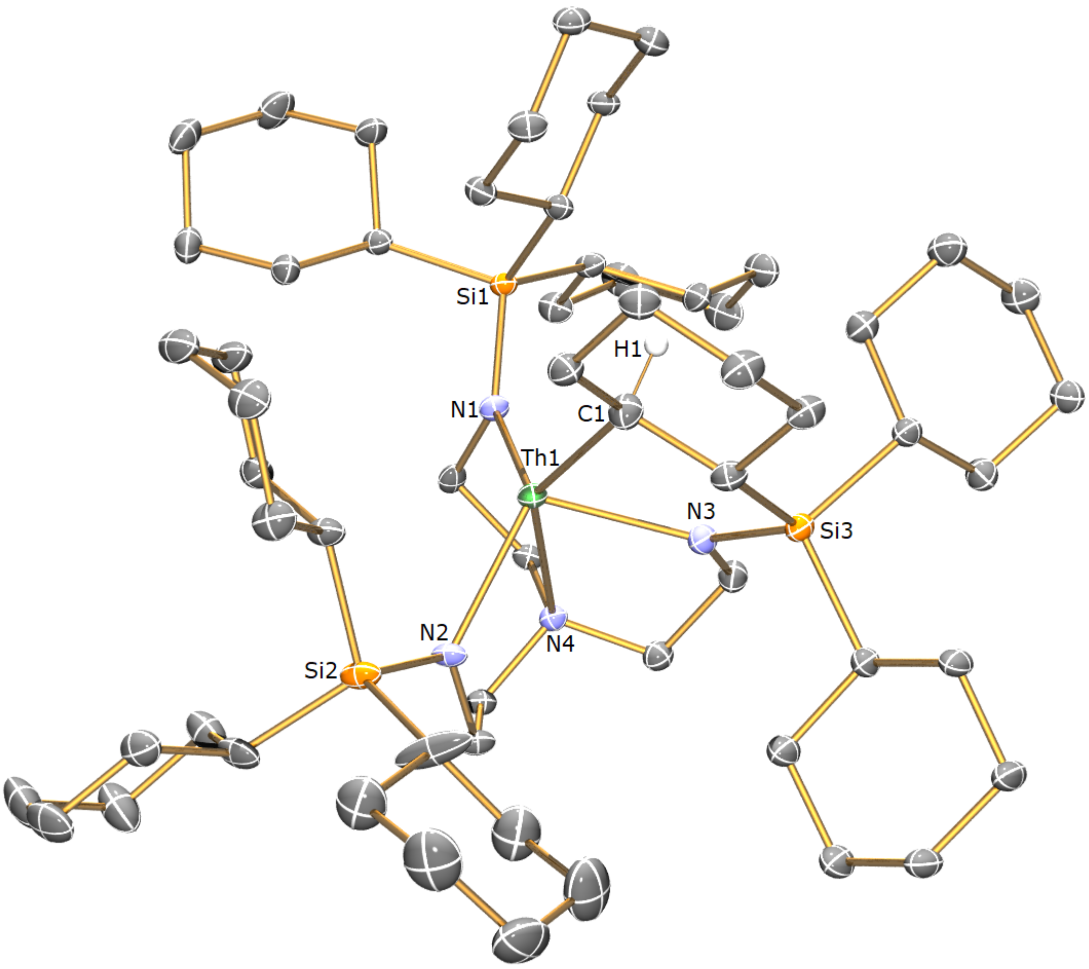

Figure S4. Molecular structure of **3** at 150 K. Displacement ellipsoids are set at 40% and hydrogen atoms except H1, disordered components, and pentane molecules in the lattice are omitted for clarity.

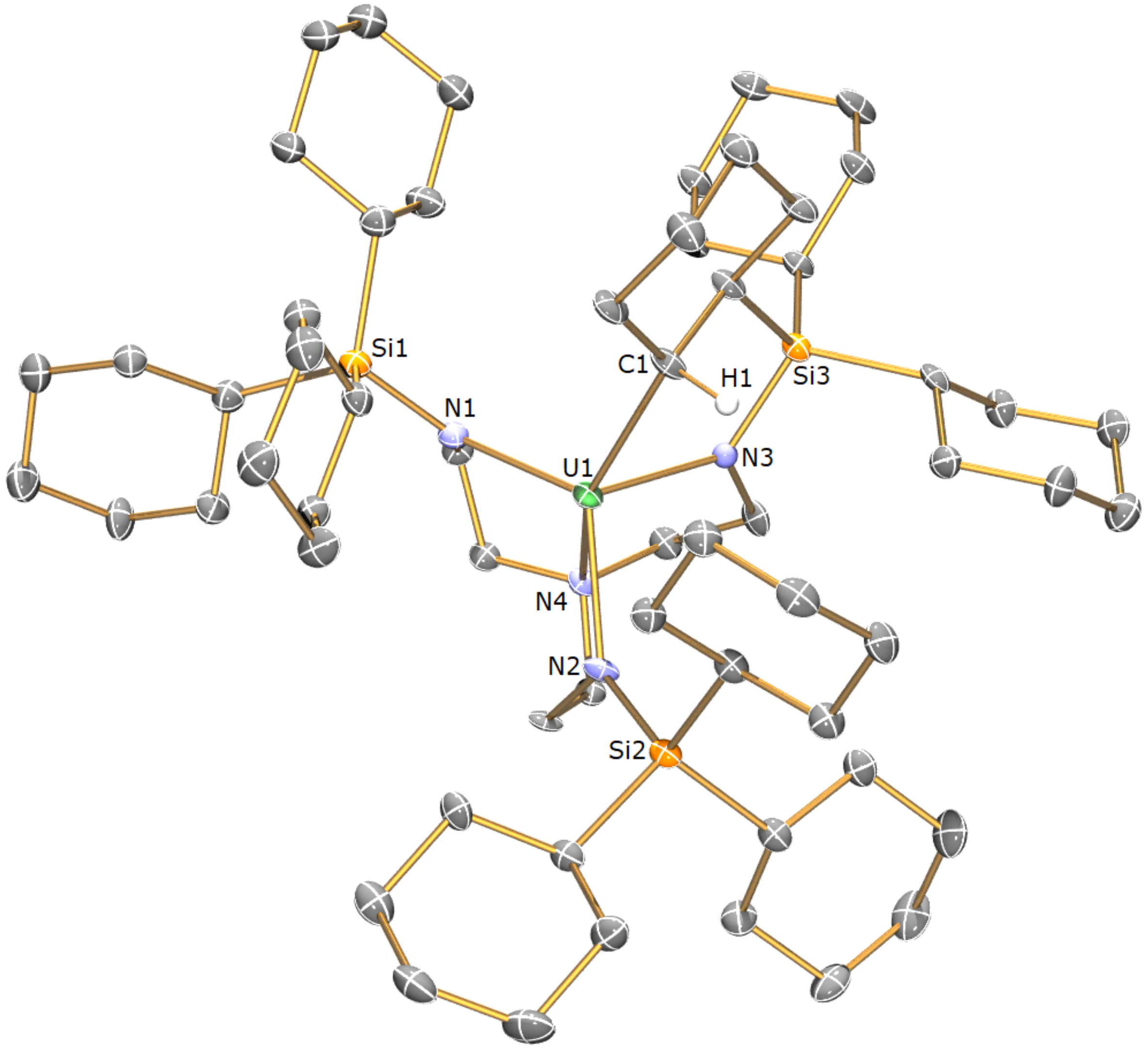

Figure S5. Molecular structure of **4** at 150 K. Displacement ellipsoids are set at 40% and hydrogen atoms except H1, and toluene molecules in the lattice are omitted for clarity.

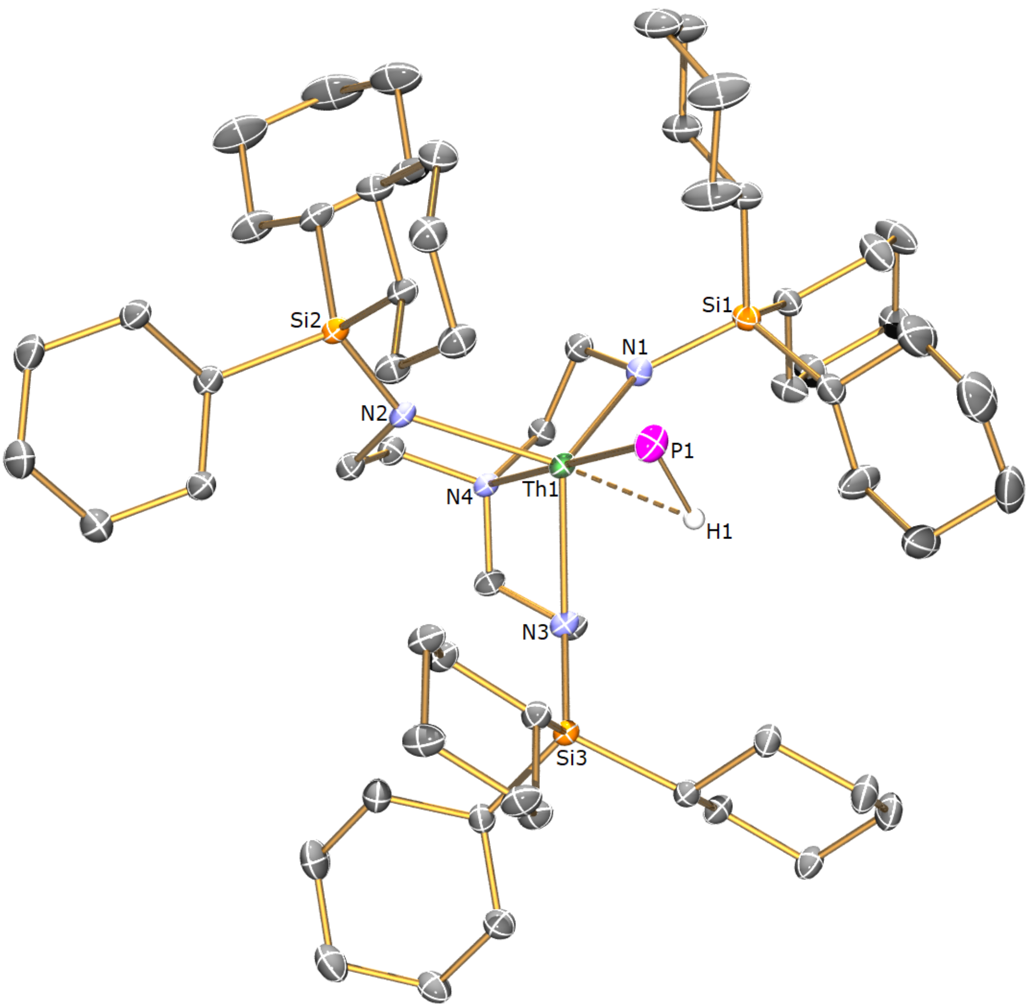

Figure S6. Molecular structure of the anion component of **5** at 150 K. Displacement ellipsoids are set at 40% and hydrogen atoms except H1, disordered components, Et_2O molecules in the lattice, and cationic component $[Na(2,2,2\text{-cryptand})]^+$ are omitted for clarity.

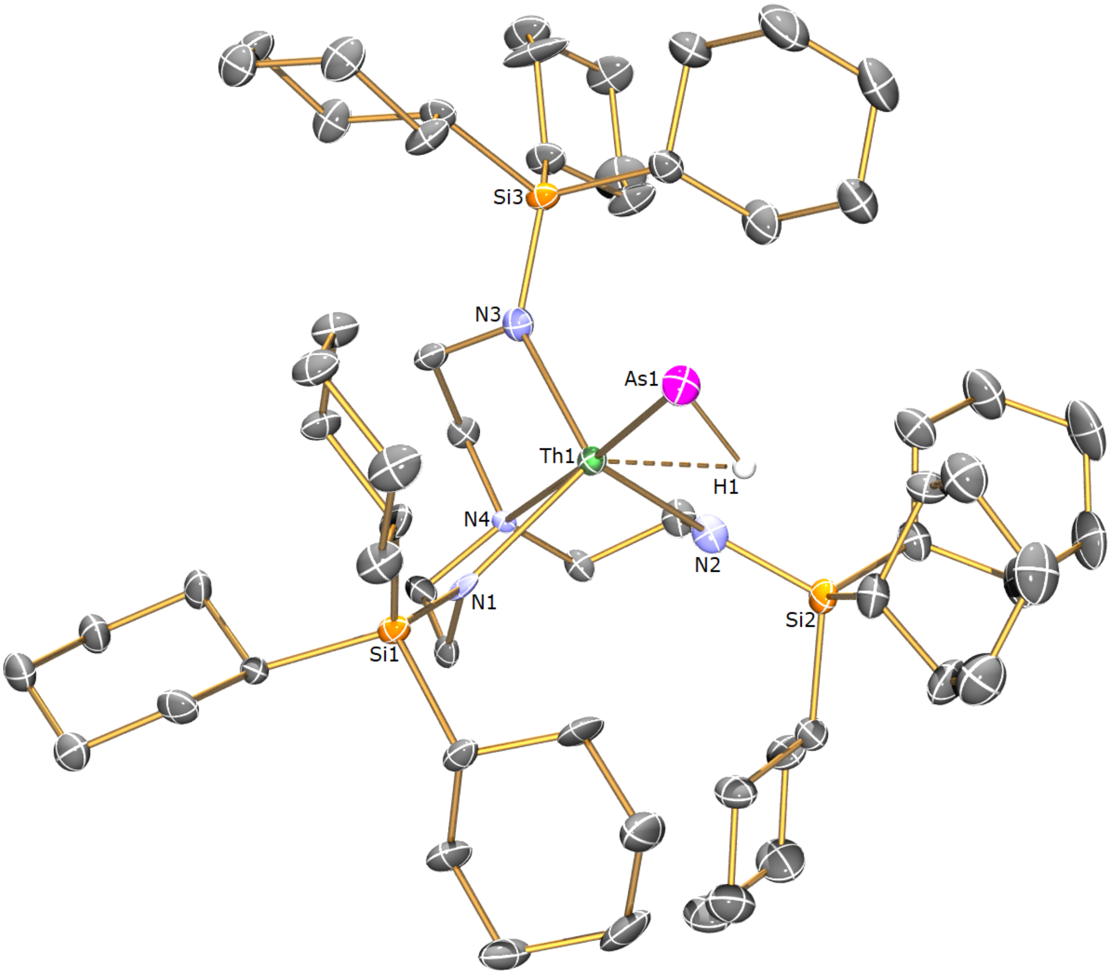

Figure S7. Molecular structure of the anion component of **6** at 150 K. Displacement ellipsoids are set at 40% and hydrogen atoms except H1, disordered components, THF molecules in the lattice, and cationic component [K(2,2,2-cryptand)]$^+$ are omitted for clarity.

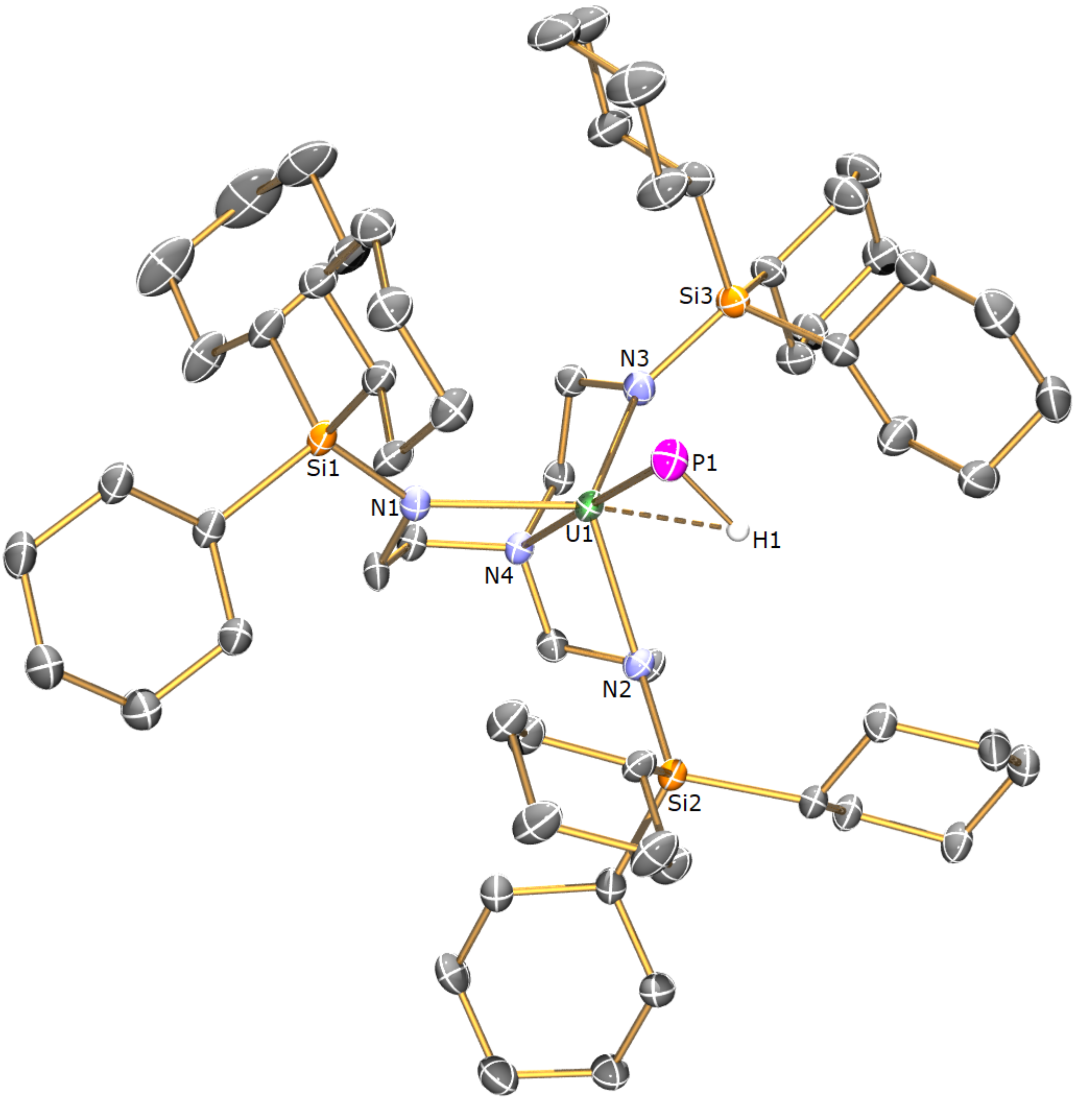

Figure S8. Molecular structure of the anion component of **7** at 150 K. Displacement ellipsoids are set at 40% and hydrogen atoms except H1, disordered components, Et_2O molecules in the lattice, and cationic component $[Na(2,2,2\text{-cryptand})]^+$ are omitted for clarity.

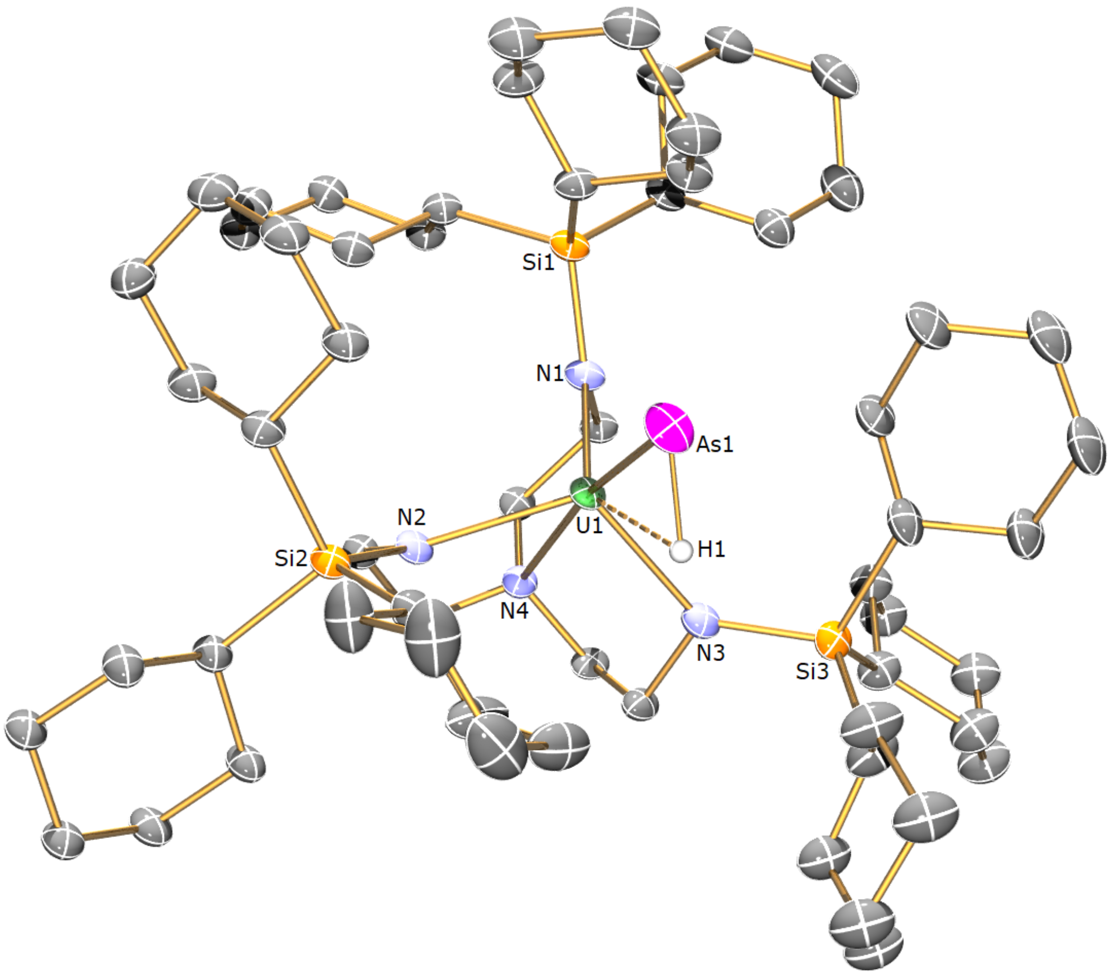

Figure S9. Molecular structure of the anion component of **8** at 150 K. Displacement ellipsoids are set at 40% and hydrogen atoms except H1, disordered components, Et_2O/toluene molecules in the lattice, and cationic component $[K(2,2,2\text{-cryptand})]^+$ are omitted for clarity.

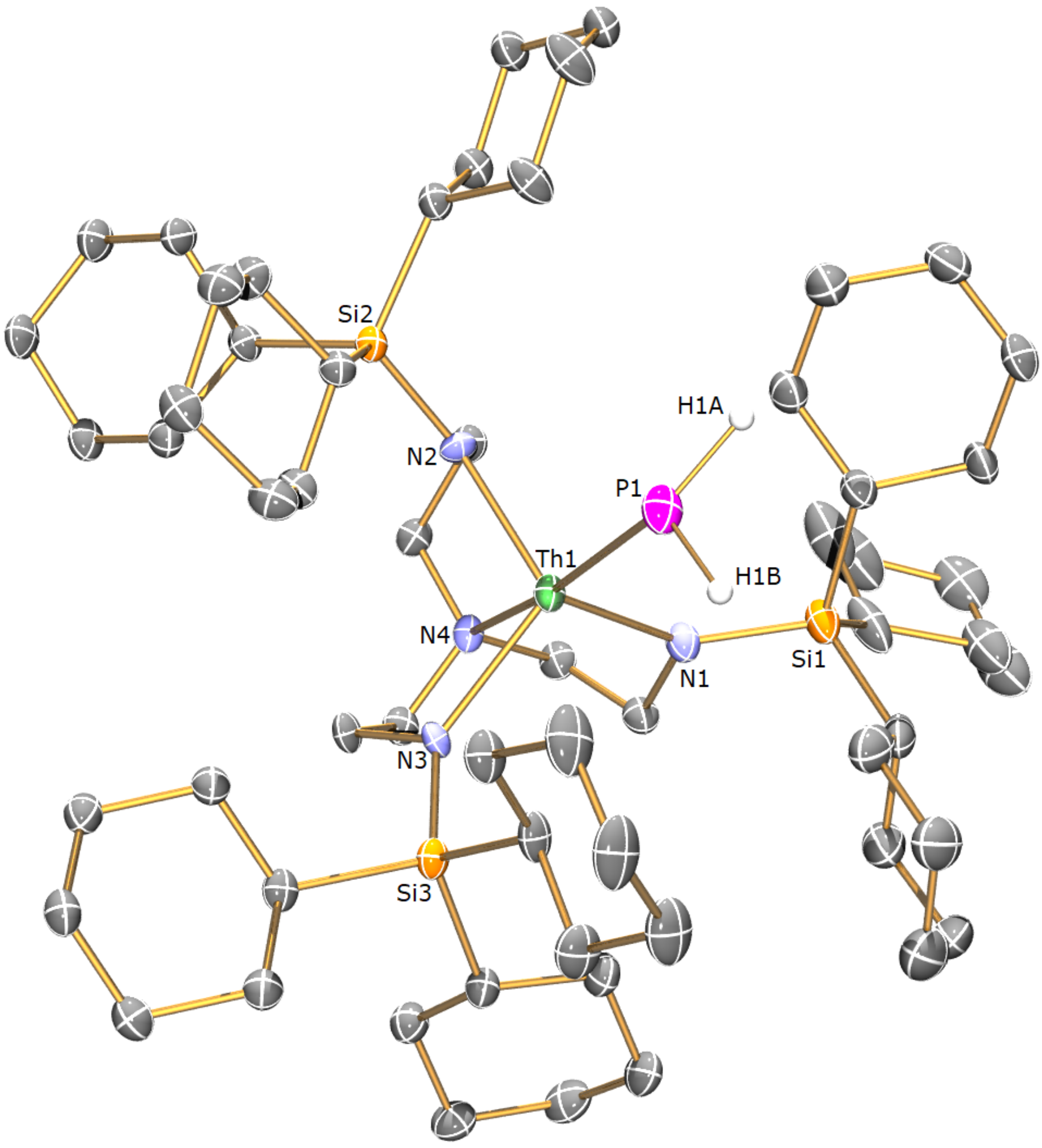

Figure S10. Molecular structure of **9** at 150 K. Displacement ellipsoids are set at 40% and hydrogen atoms except H1A and H1B, disordered components, and benzene molecules in the lattice are omitted for clarity.

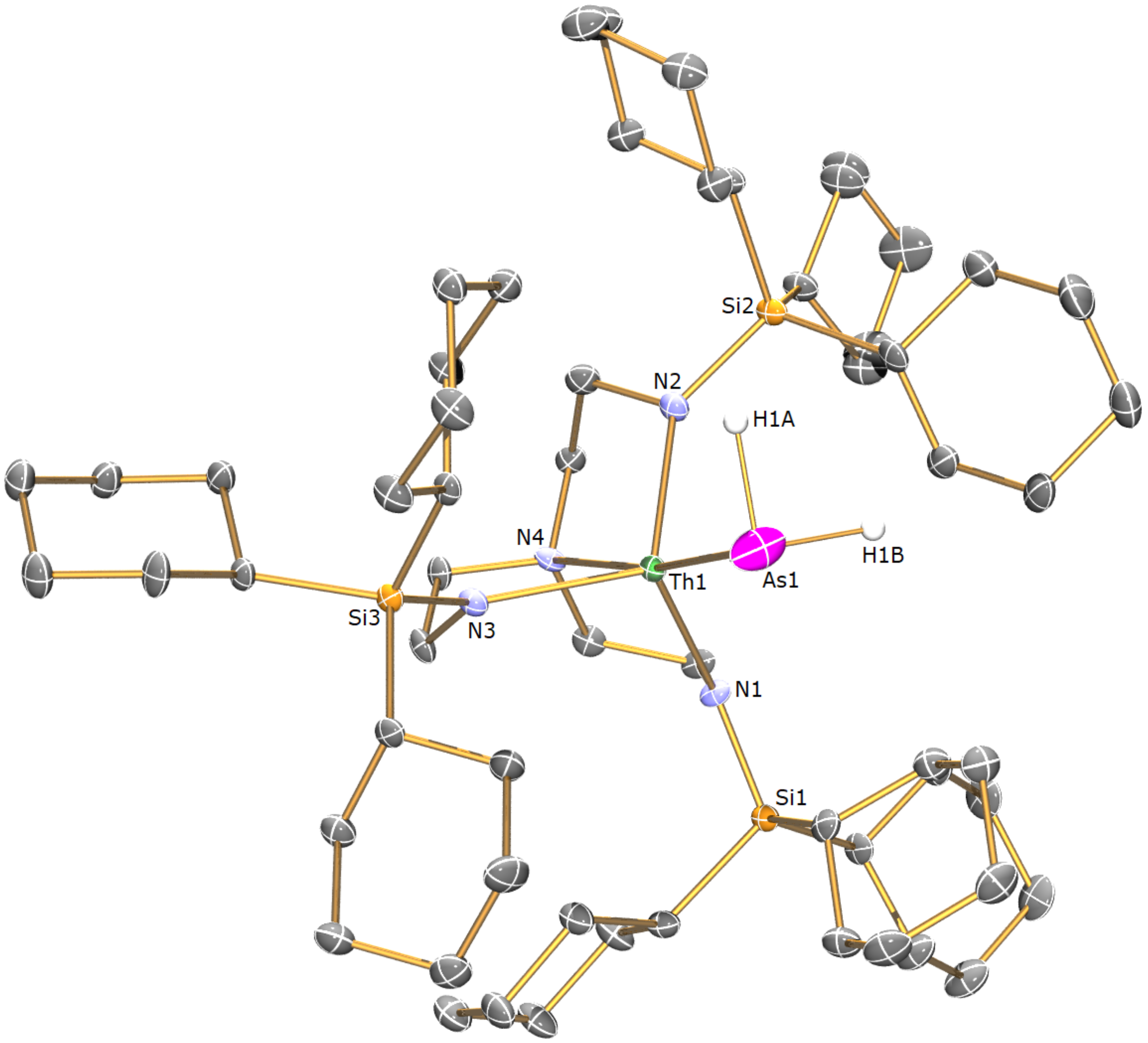

Figure S11. Molecular structure of **10** at 150 K. Displacement ellipsoids are set at 40% and hydrogen atoms except H1A and H1B, and toluene molecules in the lattice are omitted for clarity.

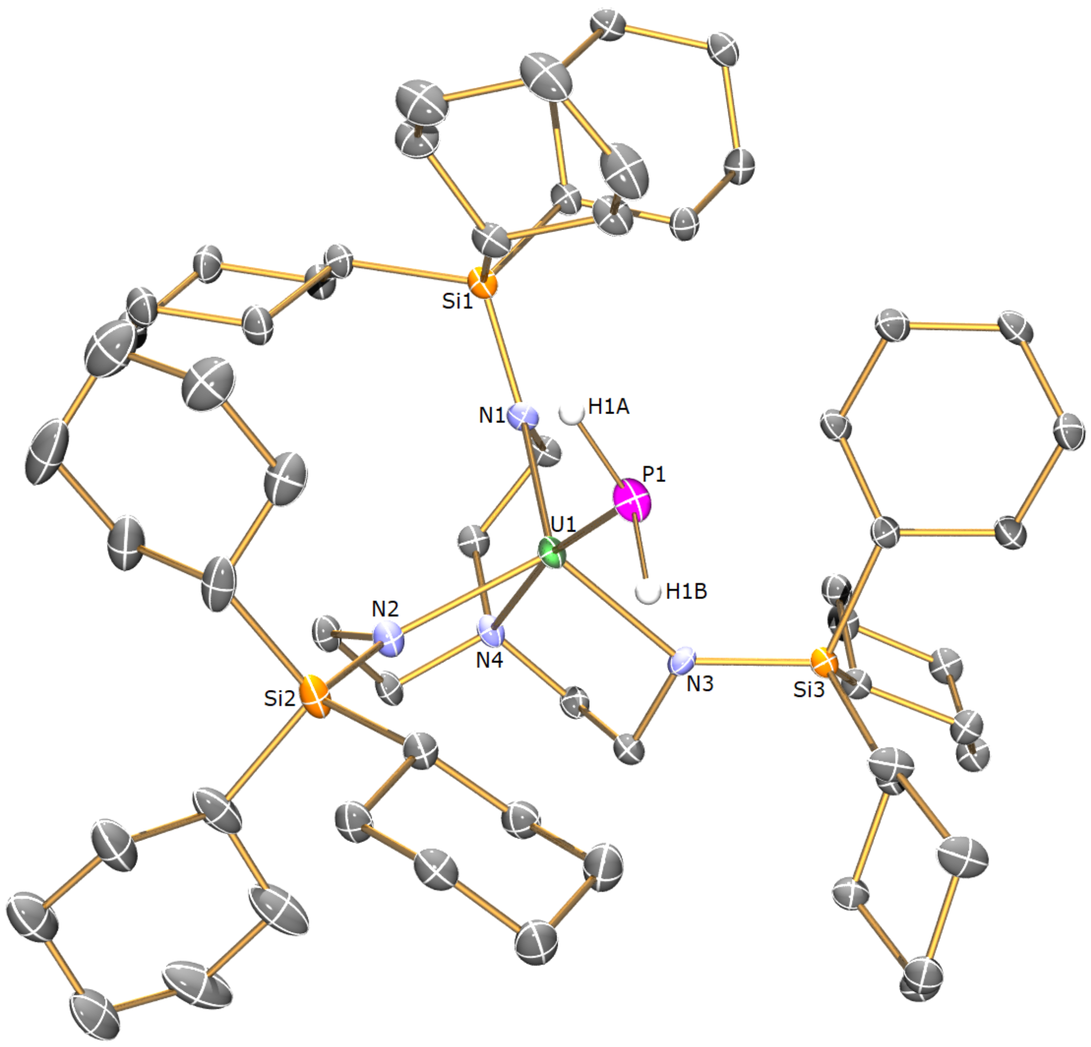

Figure S12. Molecular structure of **11** at 150 K. Displacement ellipsoids are set at 40% and hydrogen atoms except H1A and H1B are omitted for clarity.

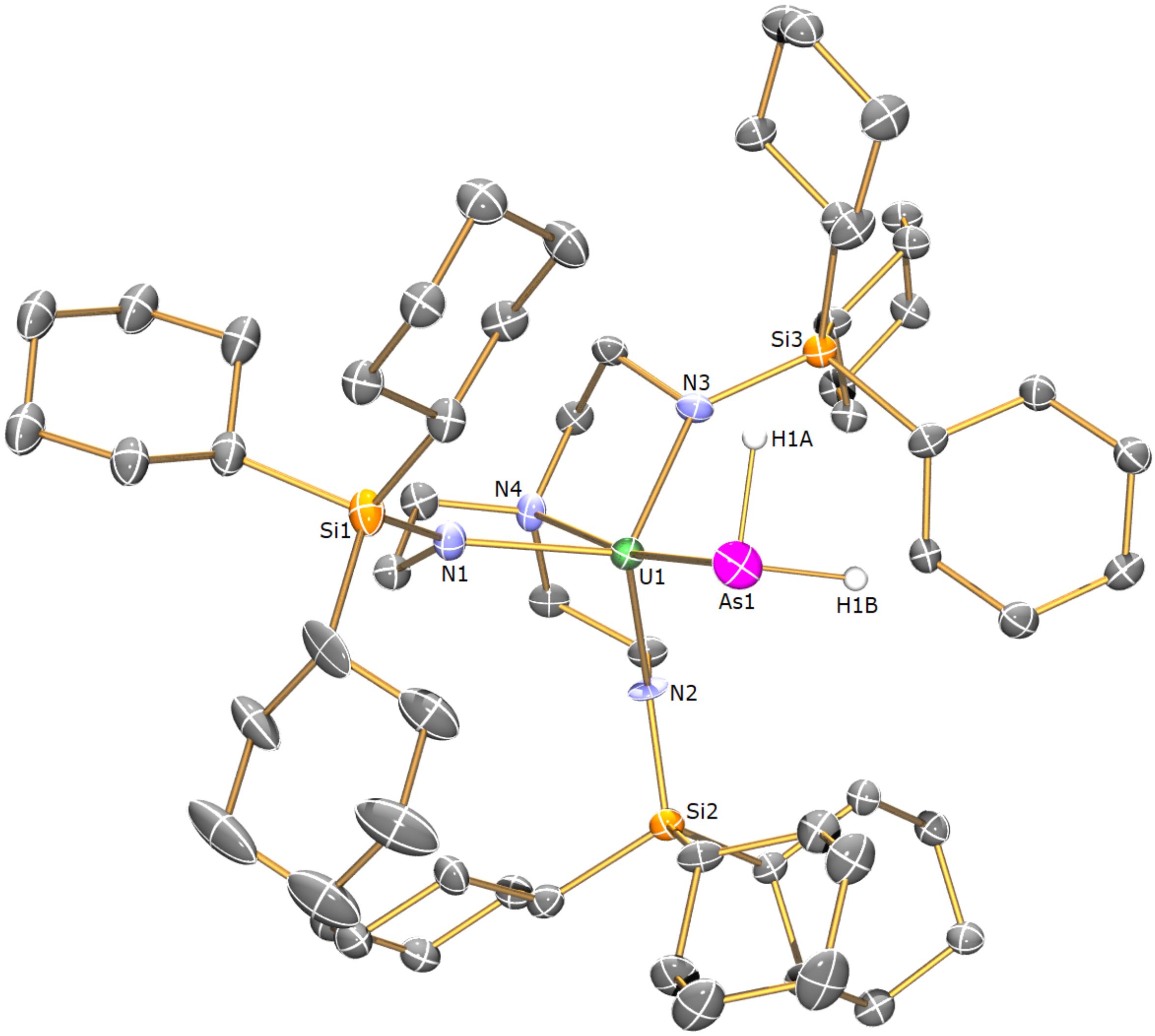

Figure S13. Molecular structure of **12** at 150 K. Displacement ellipsoids are set at 40% and hydrogen atoms except H1A and H1B, and disordered components are omitted for clarity.

ATR-IR Spectra

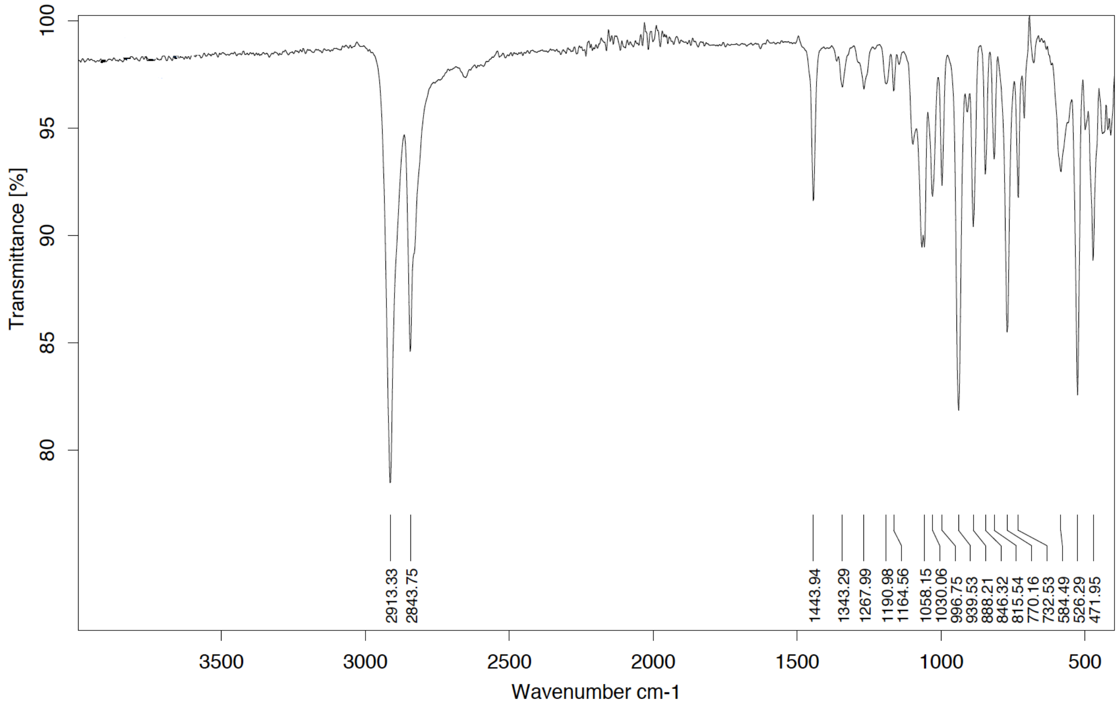

Figure S14. ATR-IR spectrum of [Tren^{TCHS}Li$_3$].

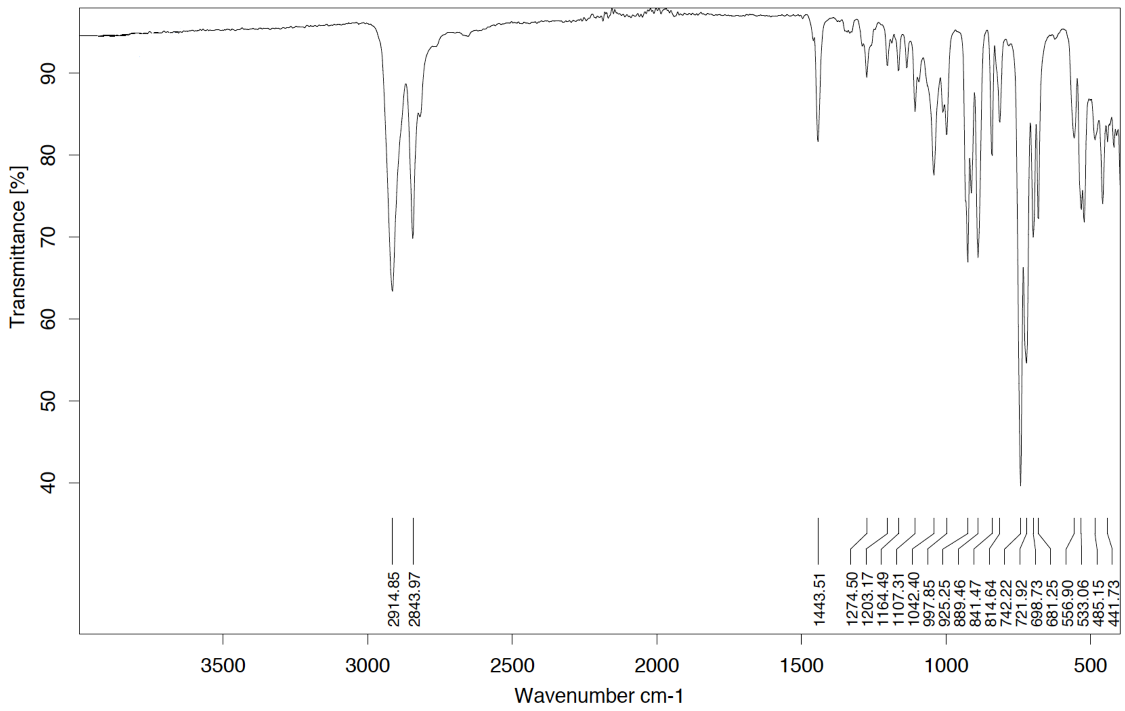

Figure S15. ATR-IR spectrum of **1**.

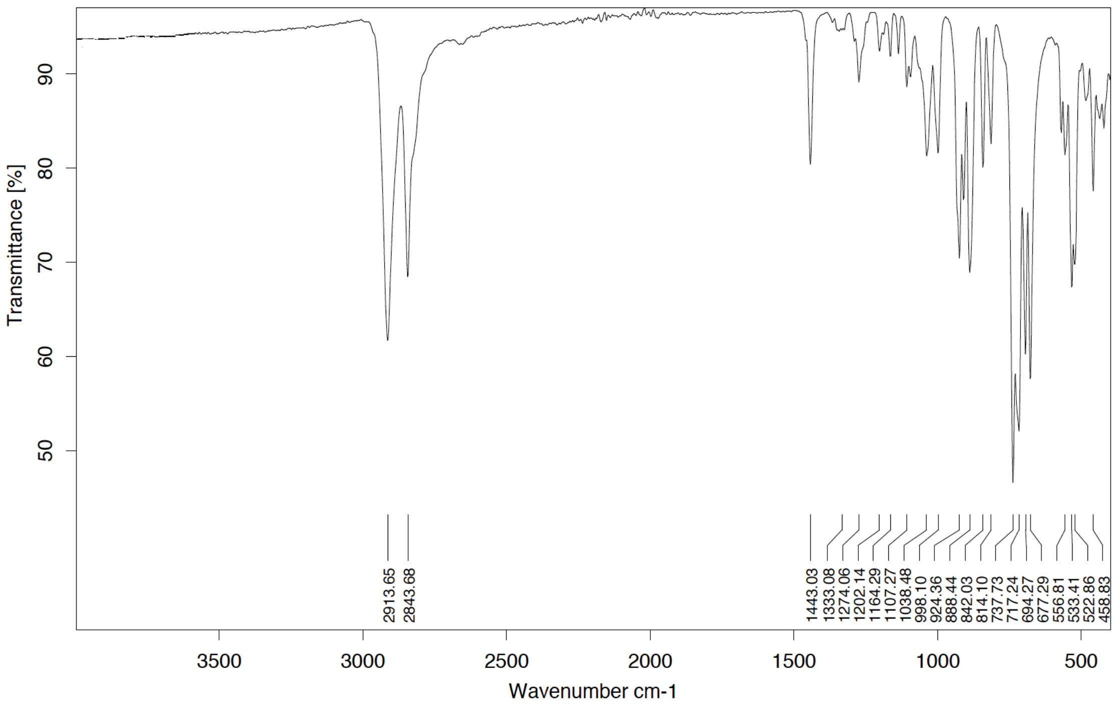

Figure S16. ATR-IR spectrum of **2**.

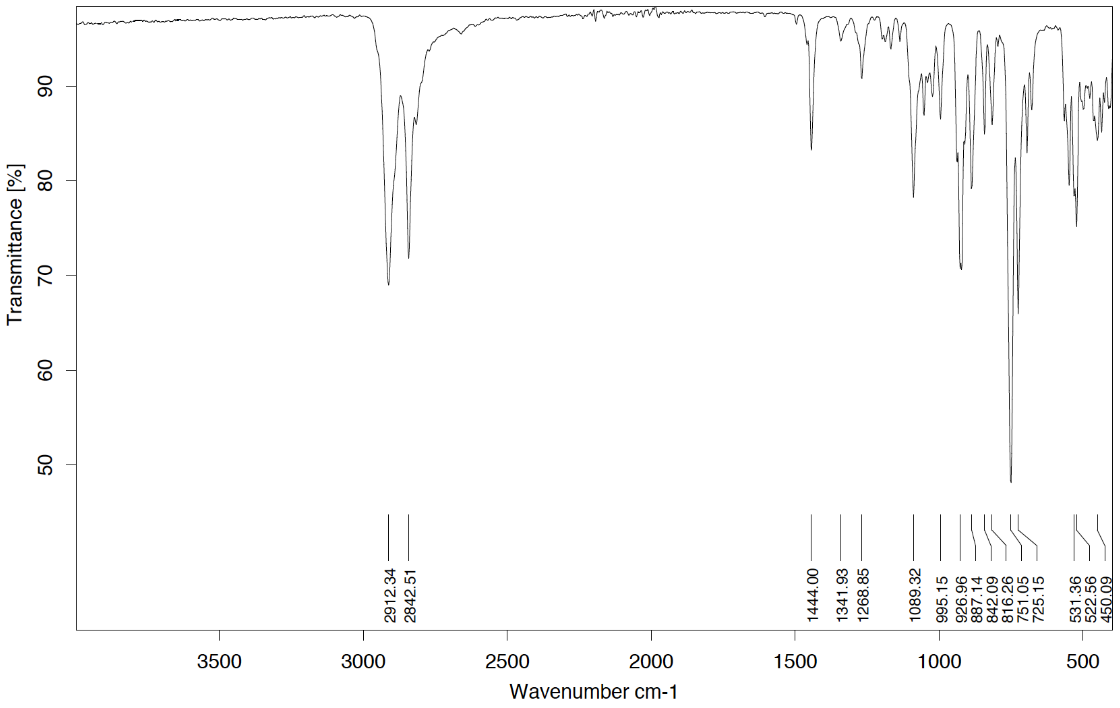

Figure S17. ATR-IR spectrum of **3**.

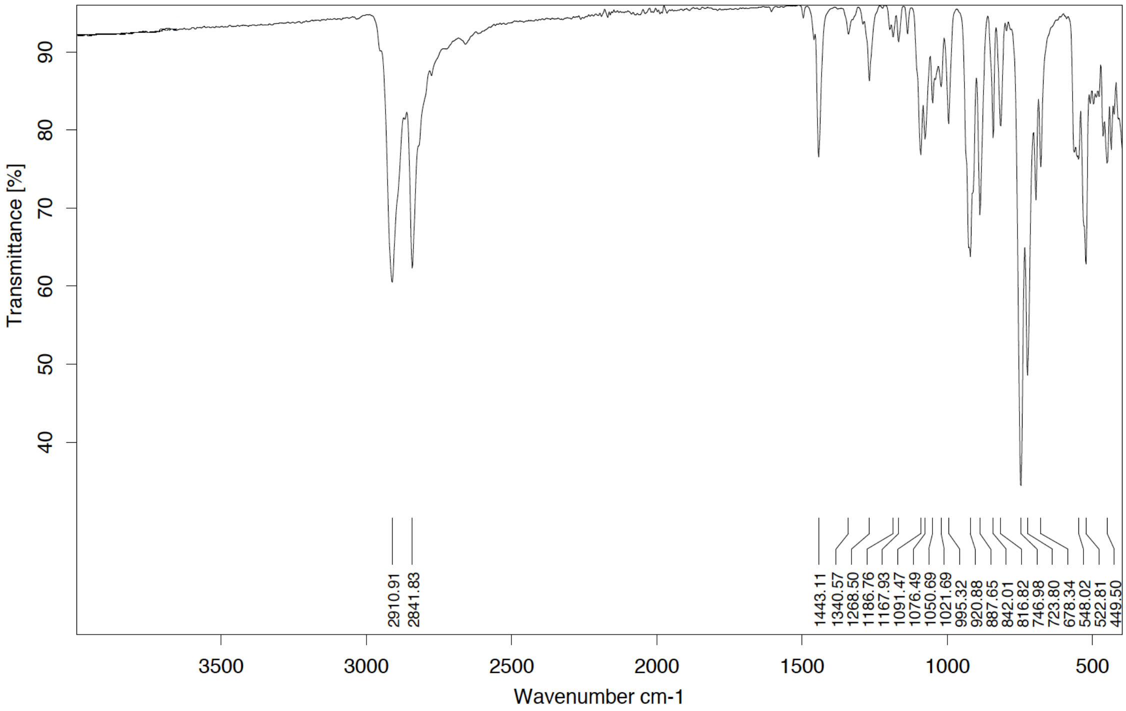

Figure S18. ATR-IR spectrum of **4**.

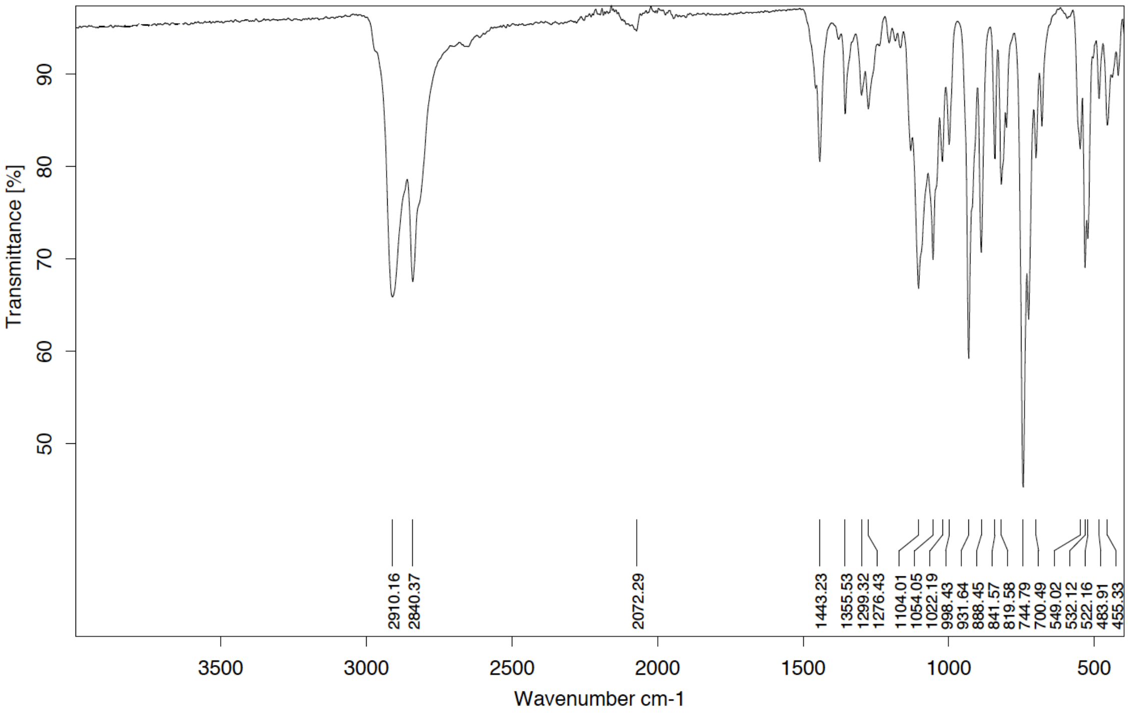

Figure S19. ATR-IR spectrum of **5**.

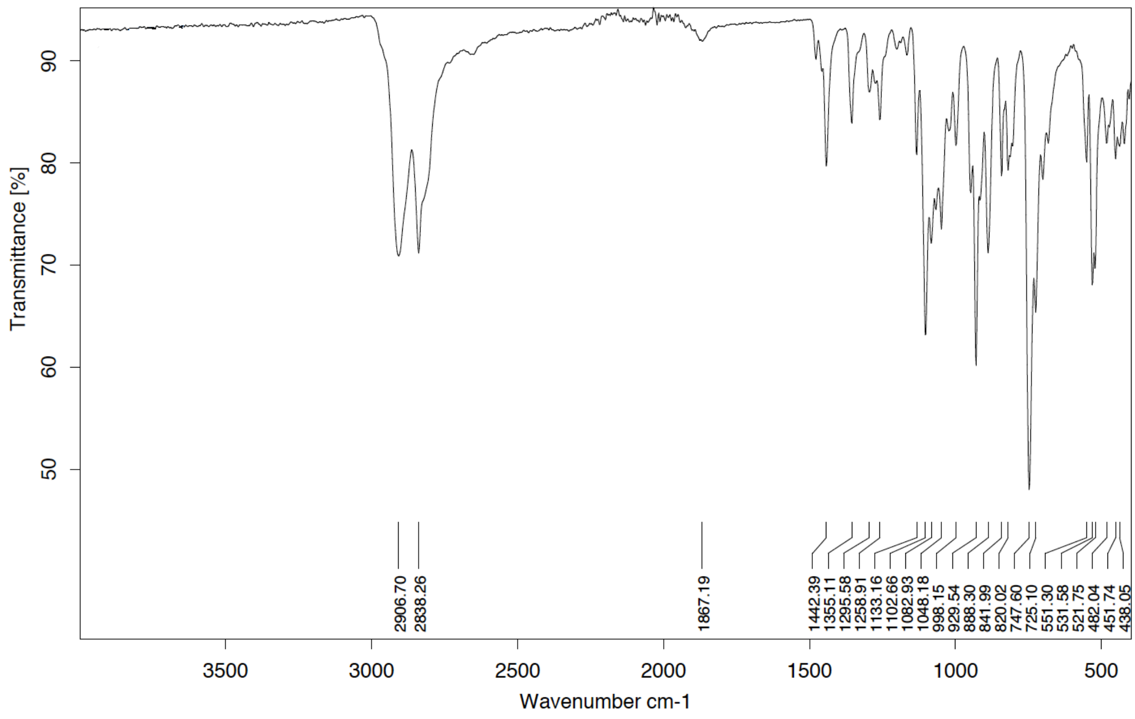

Figure S20. ATR-IR spectrum of **6**.

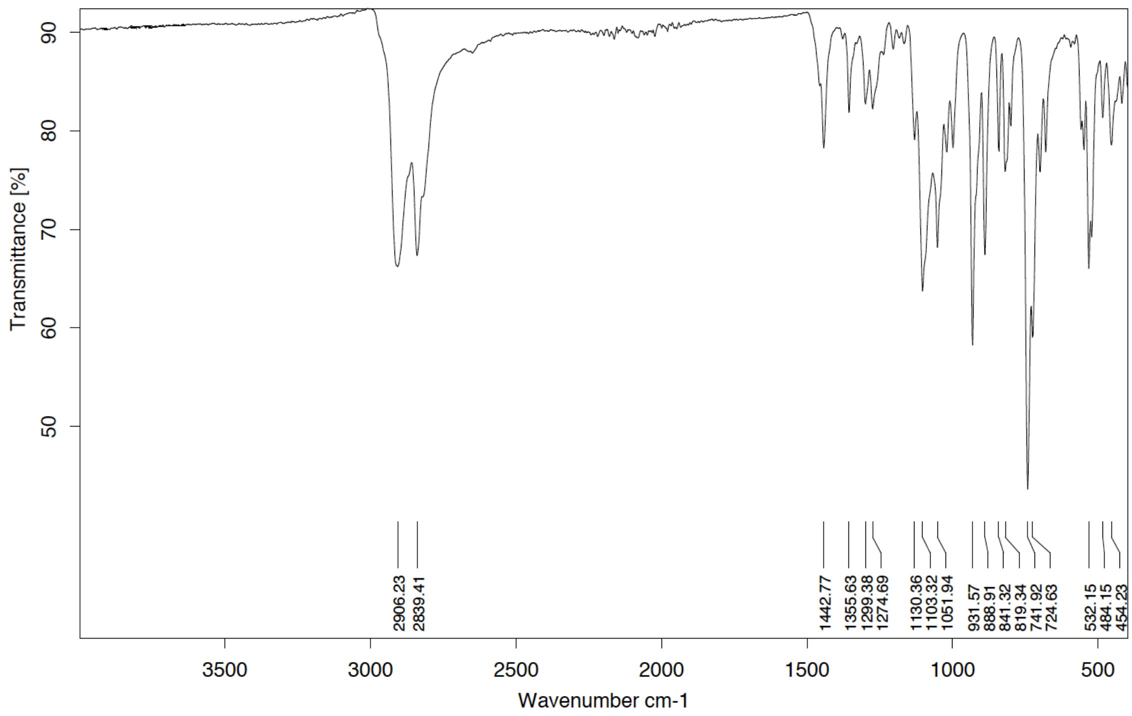

Figure S21. ATR-IR spectrum of **7**.

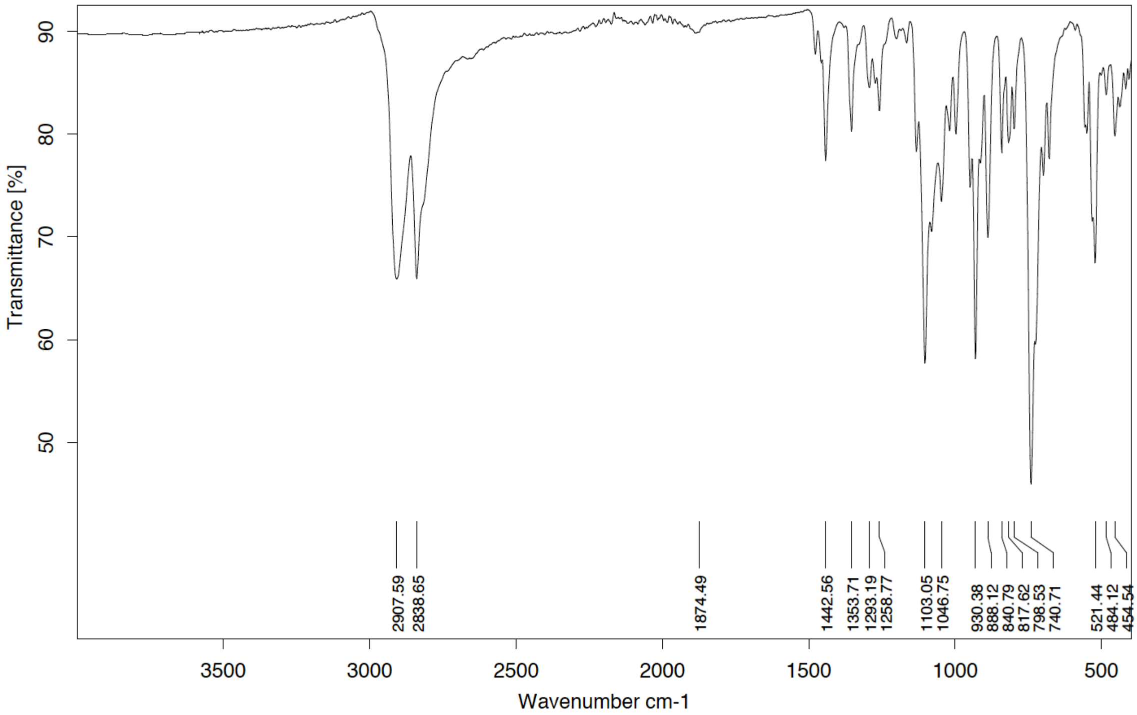

Figure S22. ATR-IR spectrum of **8**.

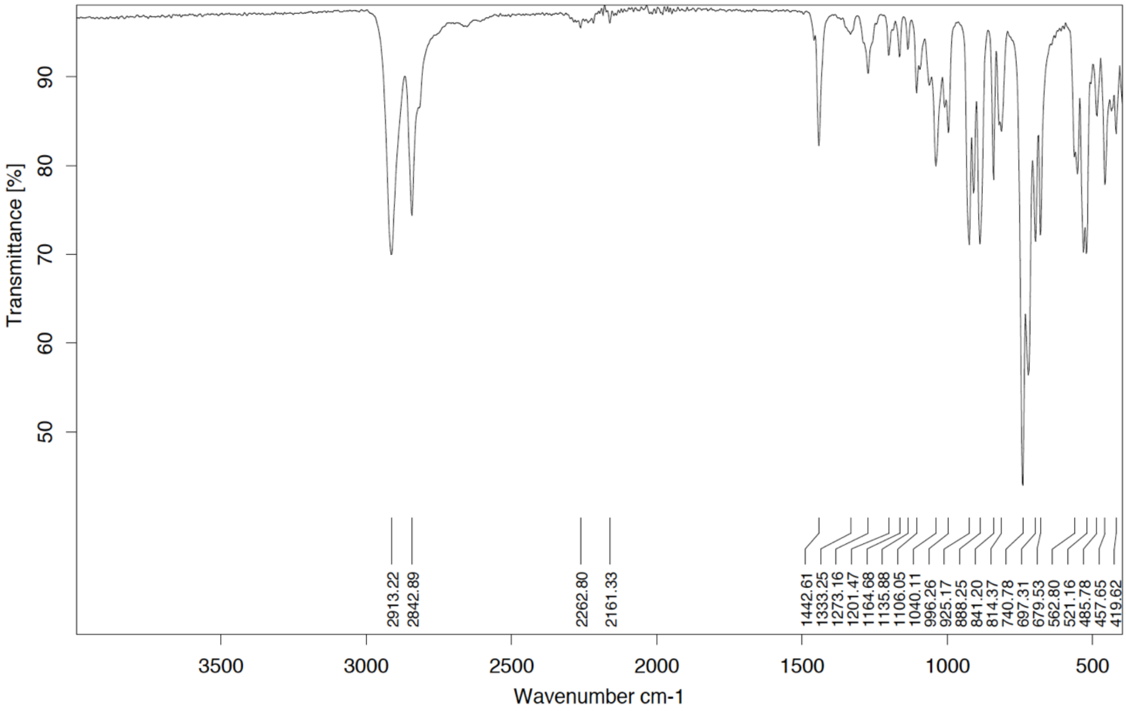

Figure S23. ATR-IR spectrum of **9**.

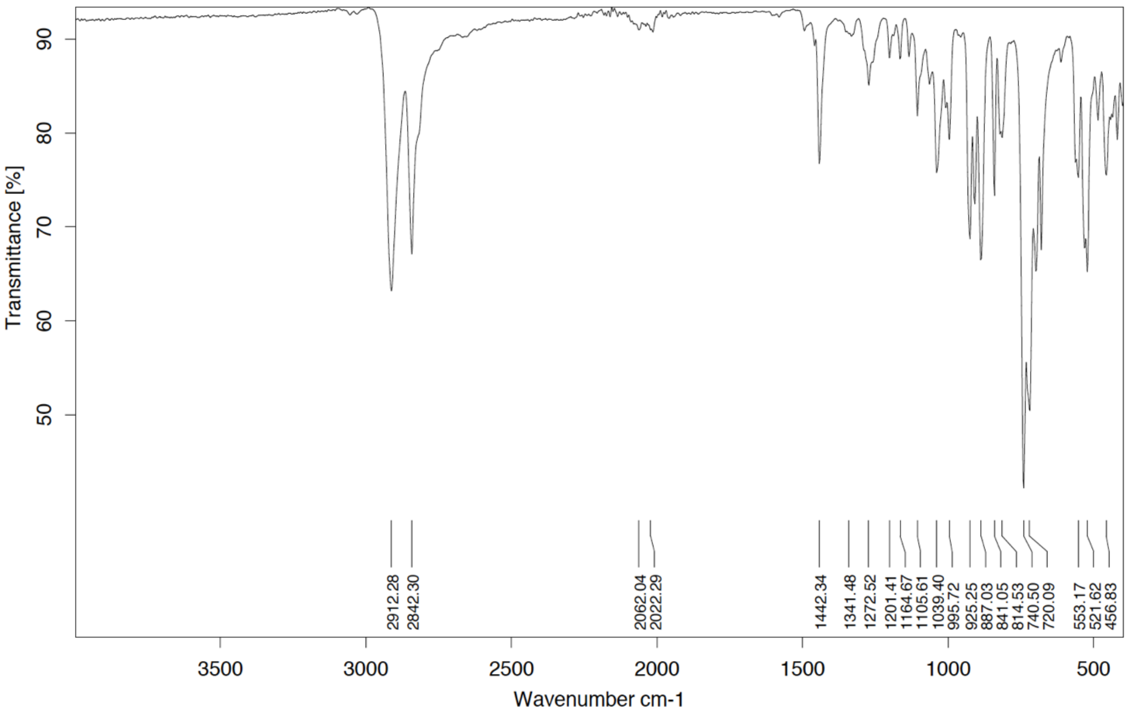

Figure S24. ATR-IR spectrum of **10**.

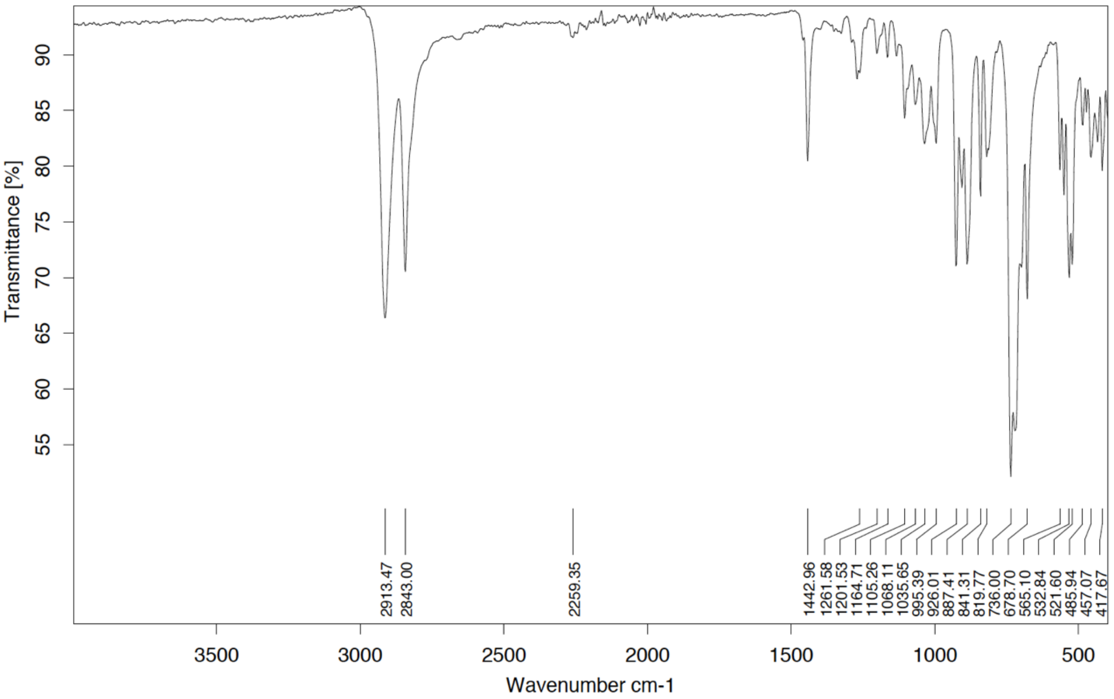

Figure S25. ATR-IR spectrum of **11**.

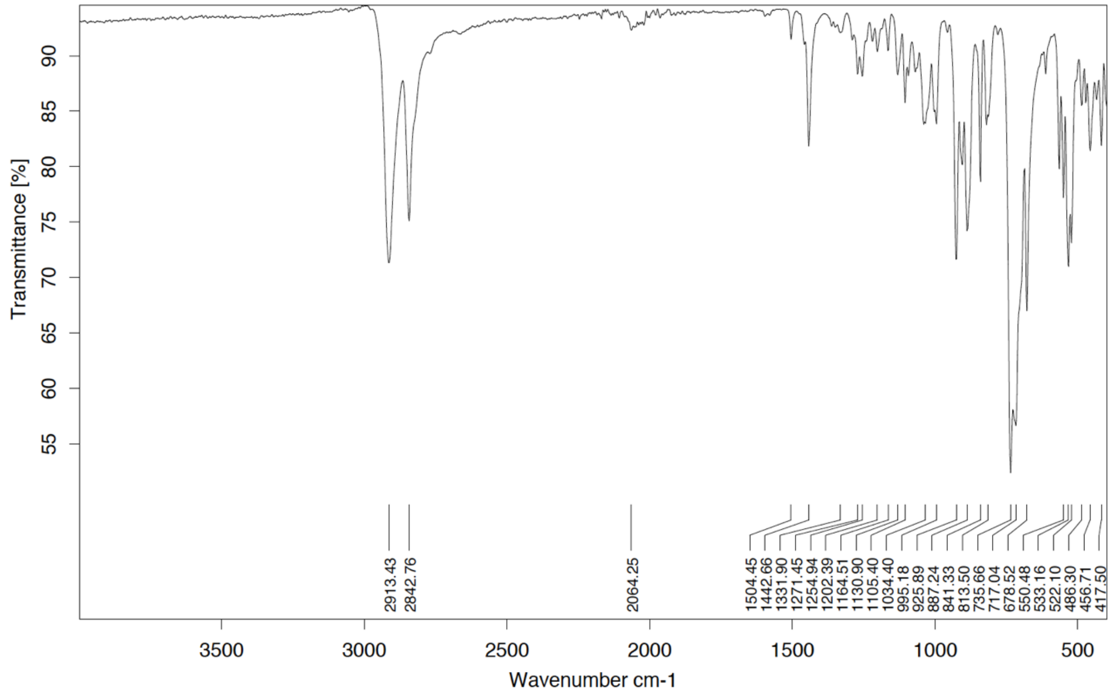

Figure S26. ATR-IR spectrum of **12**.

Raman Spectra

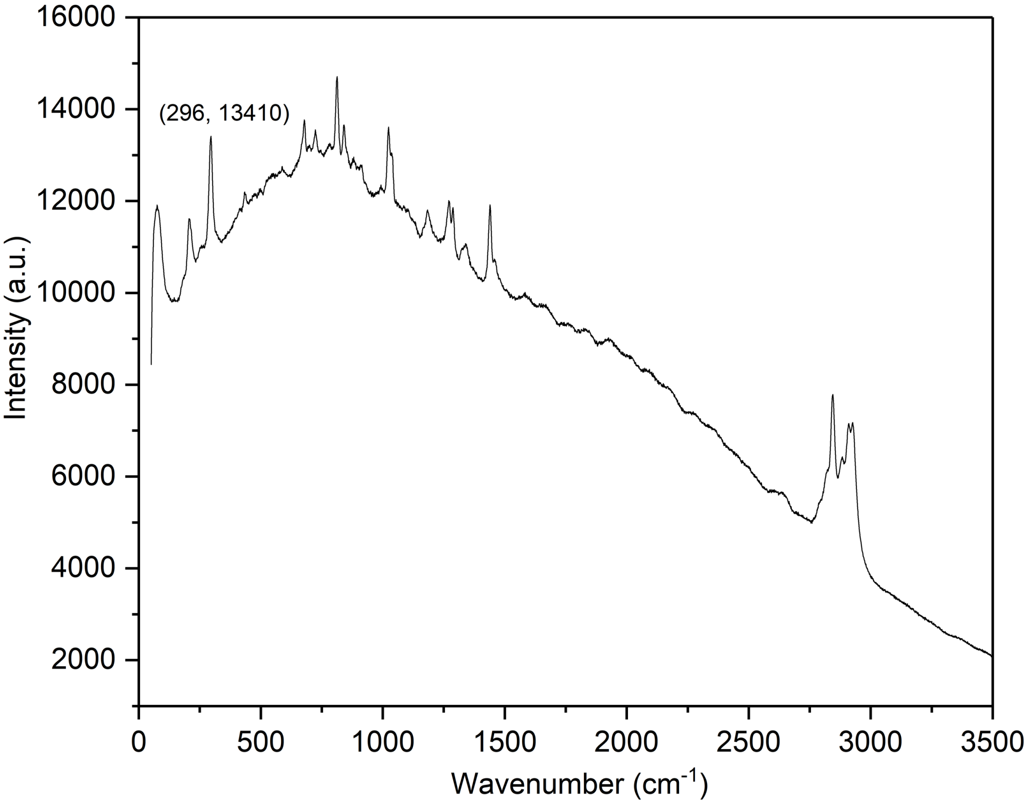

Figure S27. Raman spectrum of crystalline **5** recorded over the range of 0 to 3500 cm^{-1}.

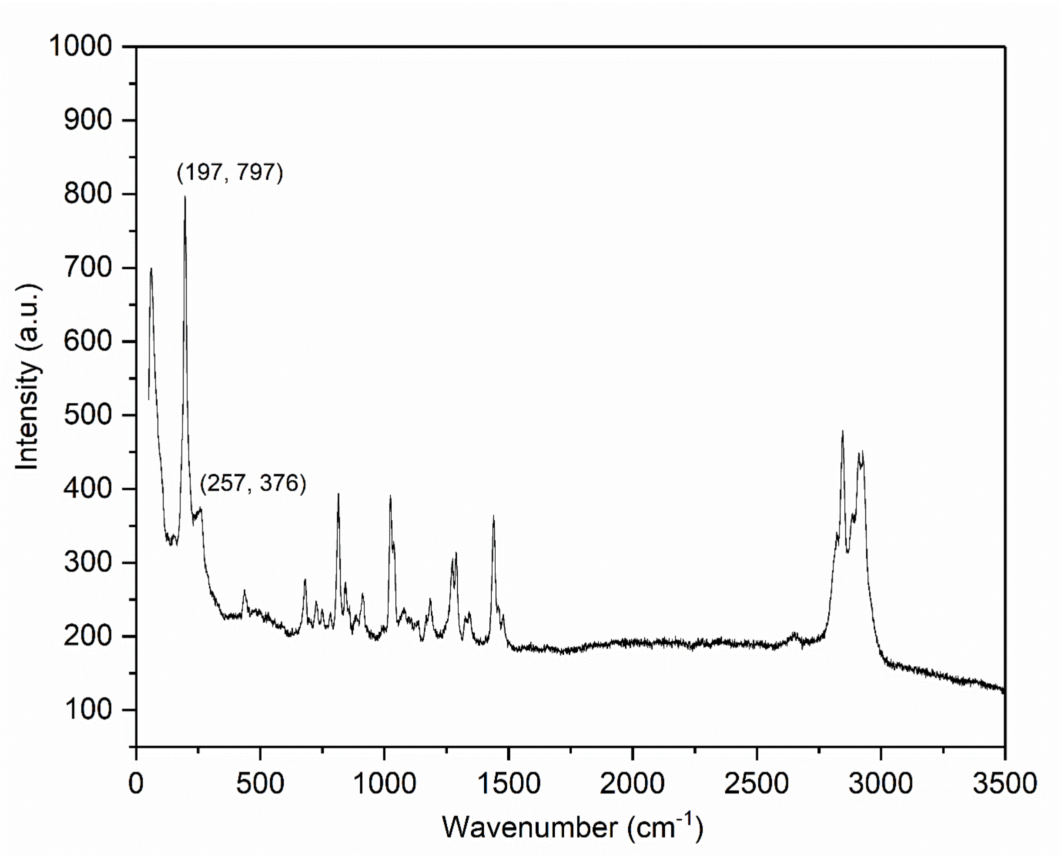

Figure S28. Raman spectrum of crystalline **6** recorded over the range of 0 to 3500 cm^{-1}.

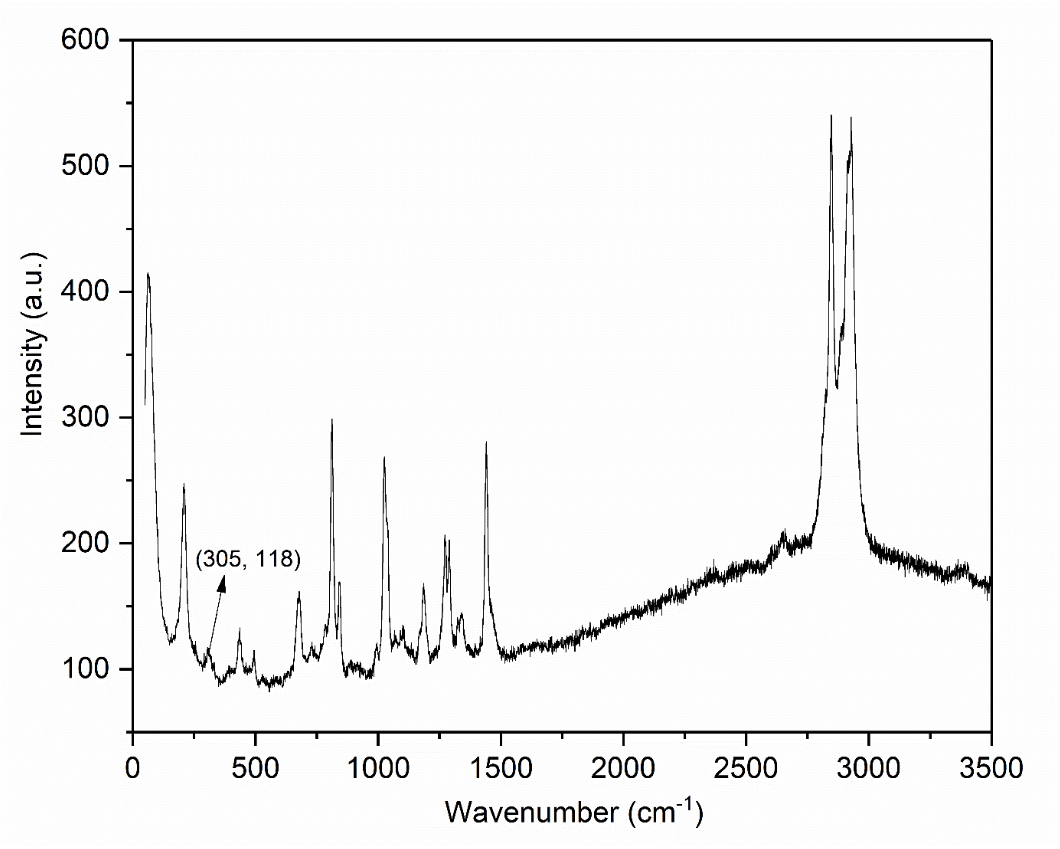

Figure S29. Raman spectrum of crystalline **7** recorded over the range of 0 to 3500 cm^{-1}.

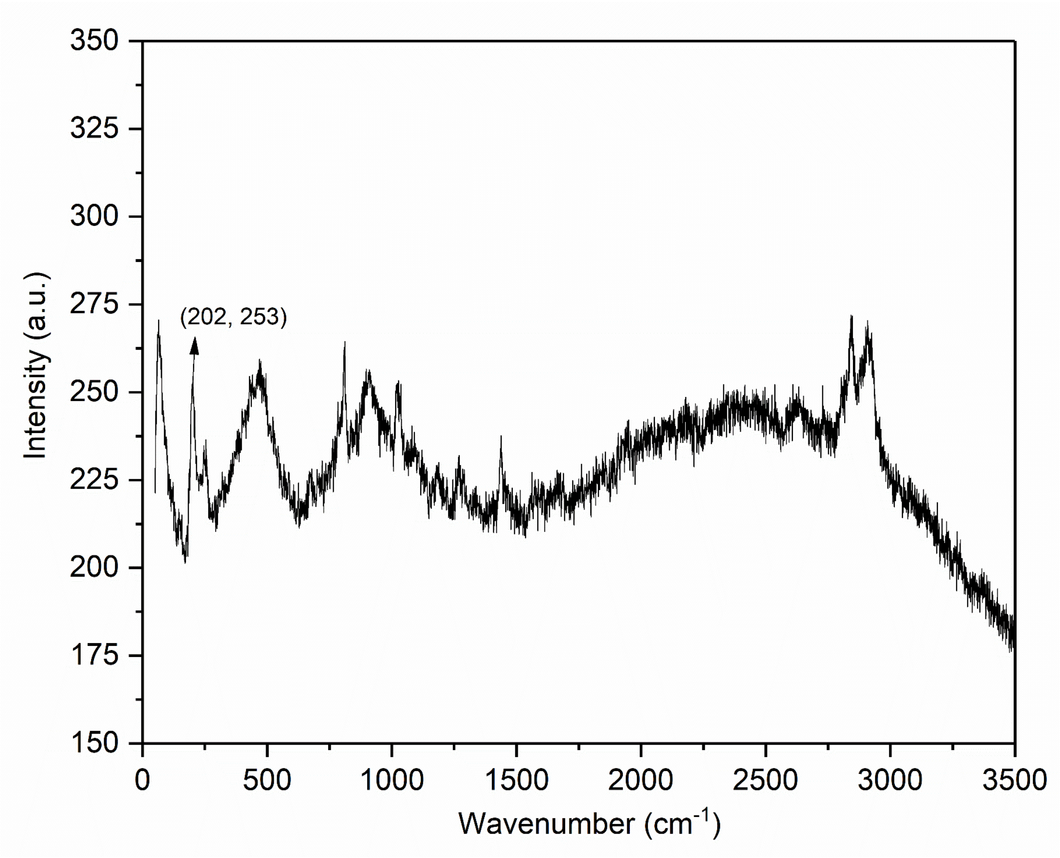

Figure S30. Raman spectrum of crystalline **8** recorded over the range of 0 to 3500 cm^{-1}.

NMR Spectra

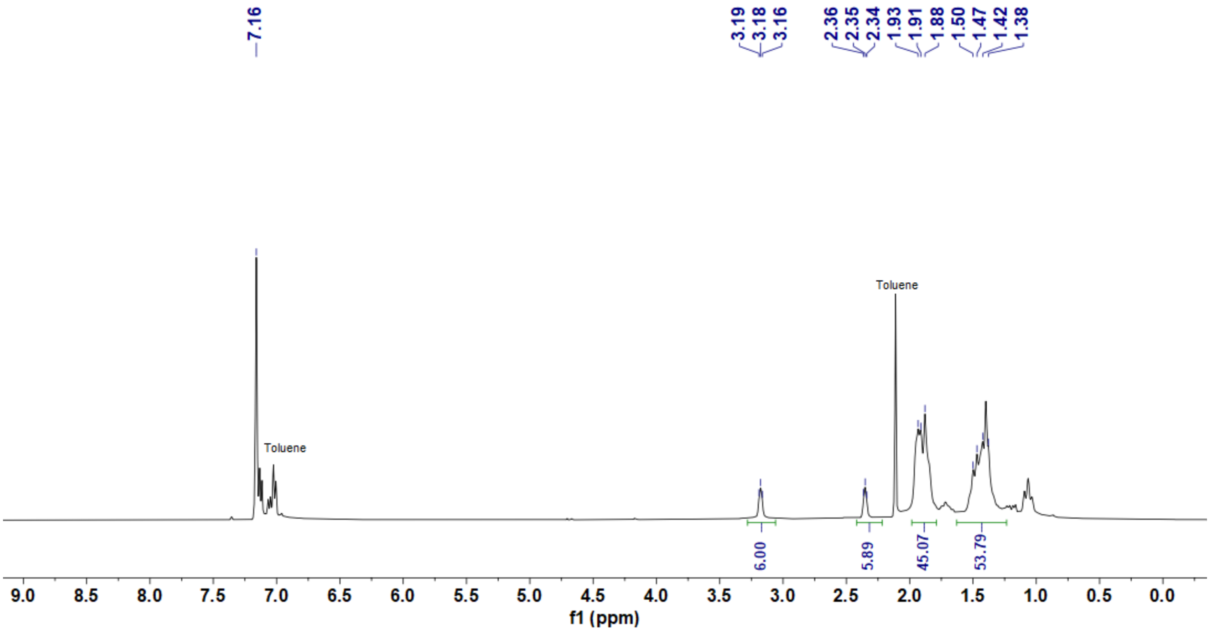

Figure S31. ^{1}H NMR (C_6D_6, 298 K) of [$Tren^{TCHS}Li_3$].

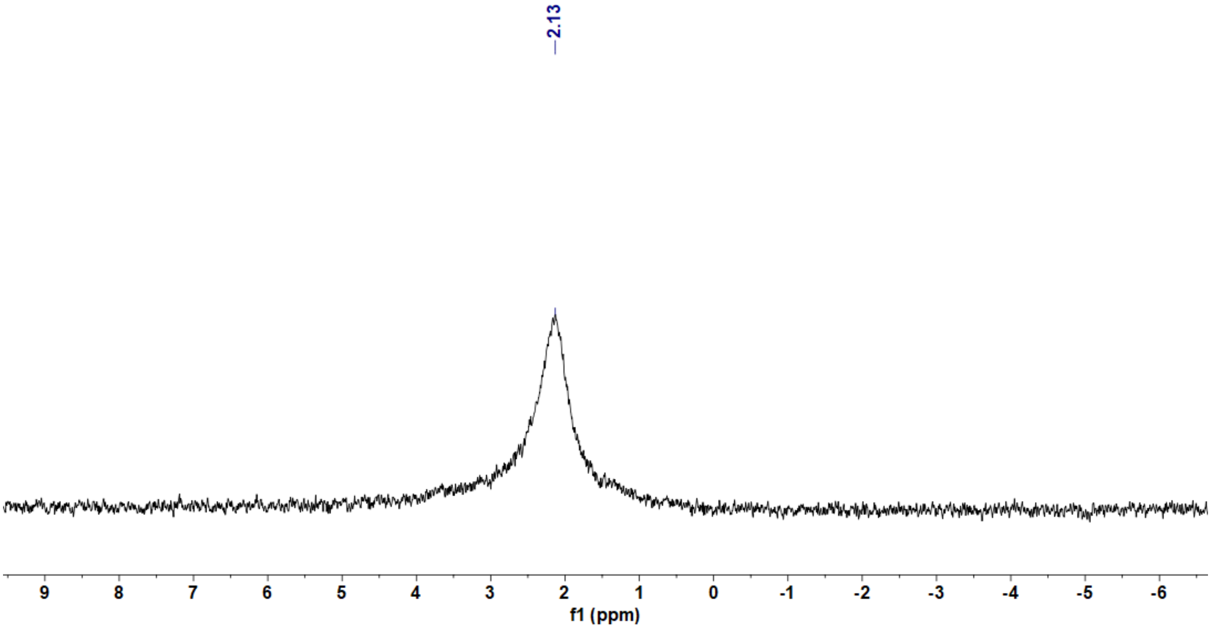

Figure S32. $^{7}Li\{^{1}H\}$ NMR (C_6D_6, 298 K) of [Tren^{TCHS}Li$_3$].

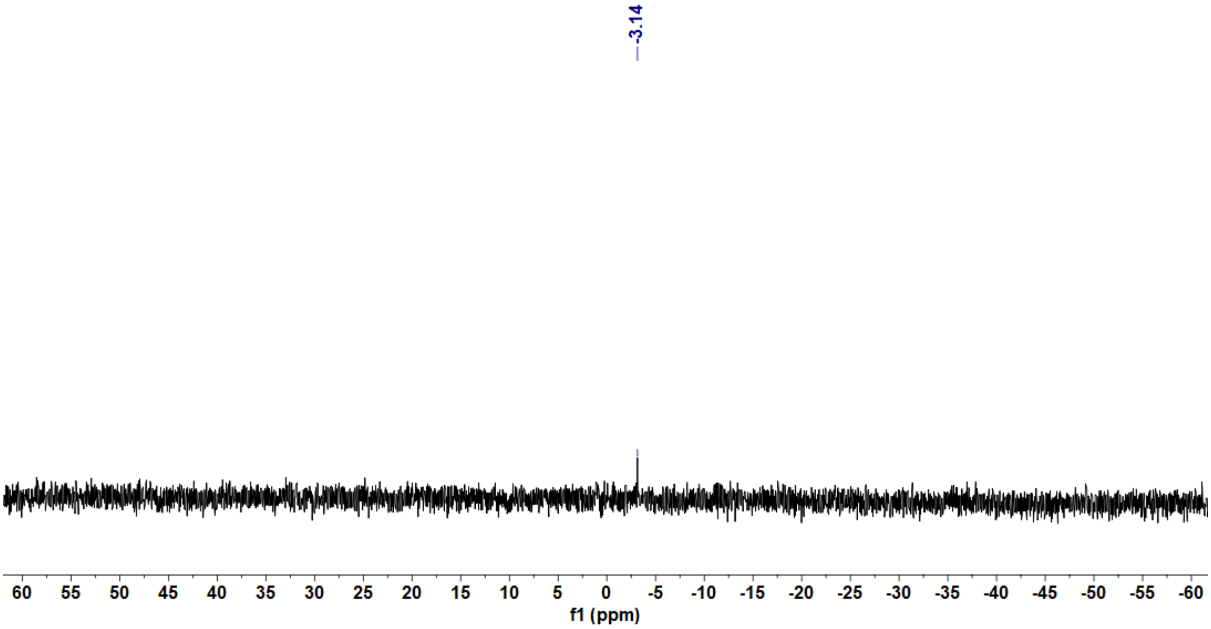

Figure S33. $^{29}Si\{^{1}H\}$ NMR (C_6D_6, 298 K) of [Tren^{TCHS}Li$_3$].

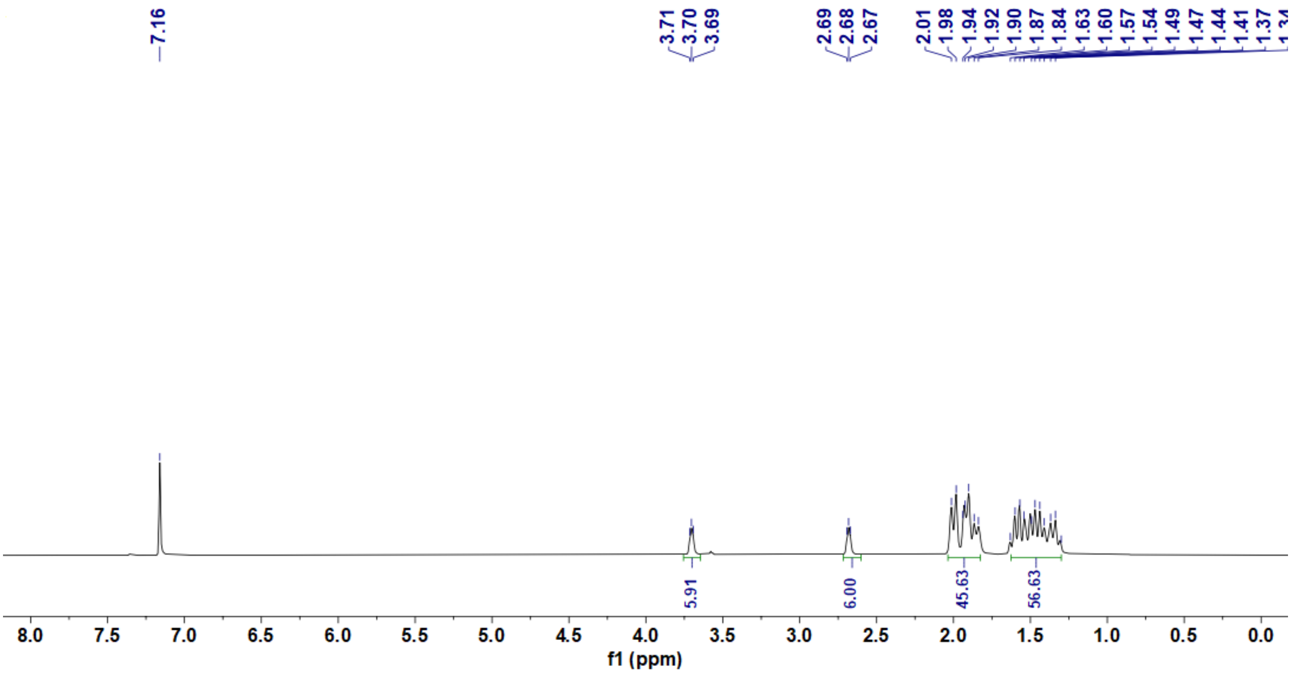

Figure S34. ^{1}H NMR (C_6D_6, 298 K) of **1**.

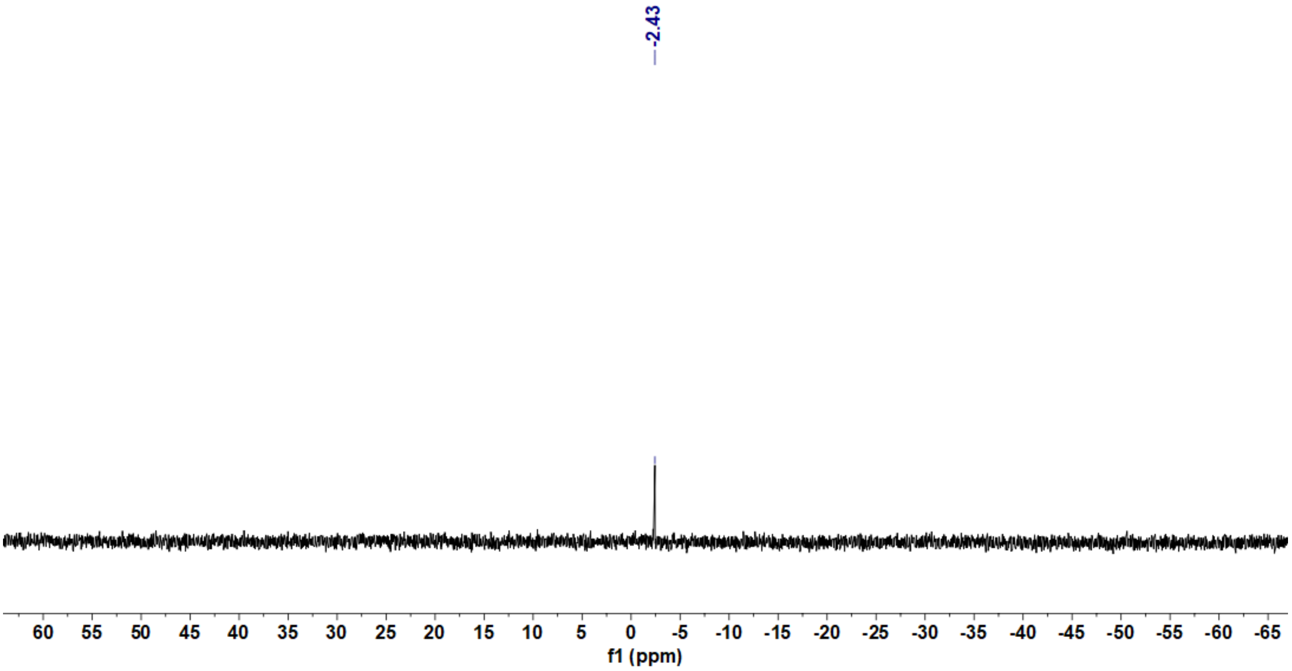

Figure S35. $^{29}Si\{^{1}H\}$ NMR (C_6D_6, 298 K) of **1**.

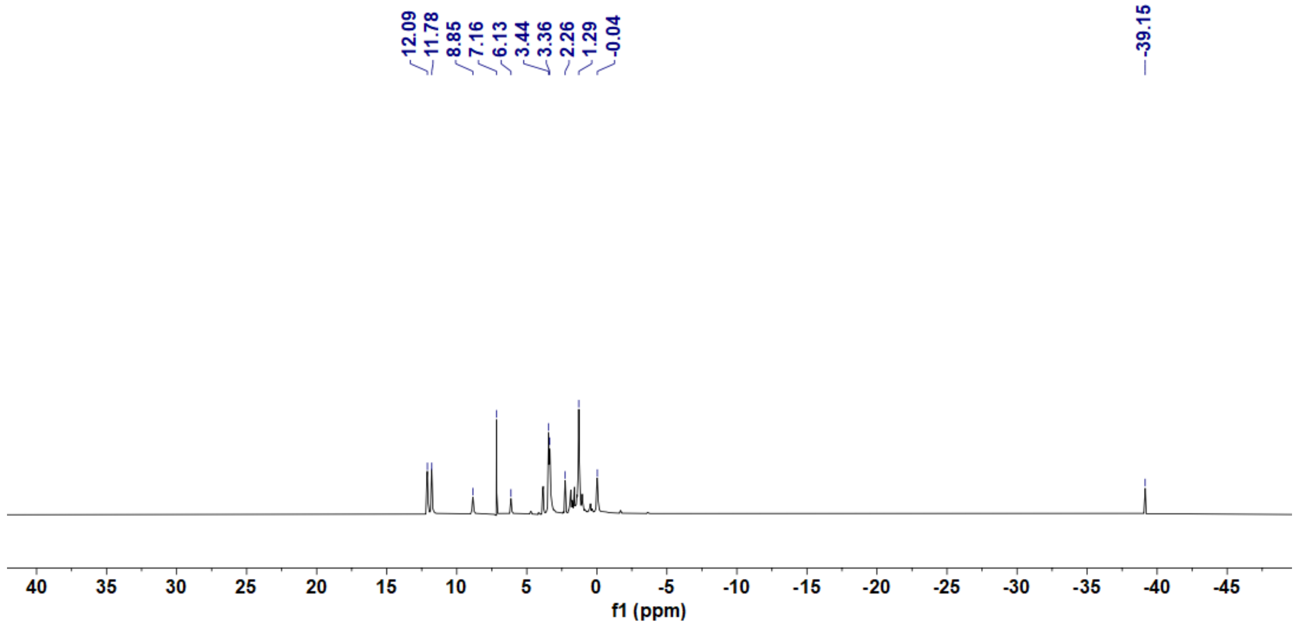

Figure S36. ^{1}H NMR (C_6D_6, 298 K) of **2**.

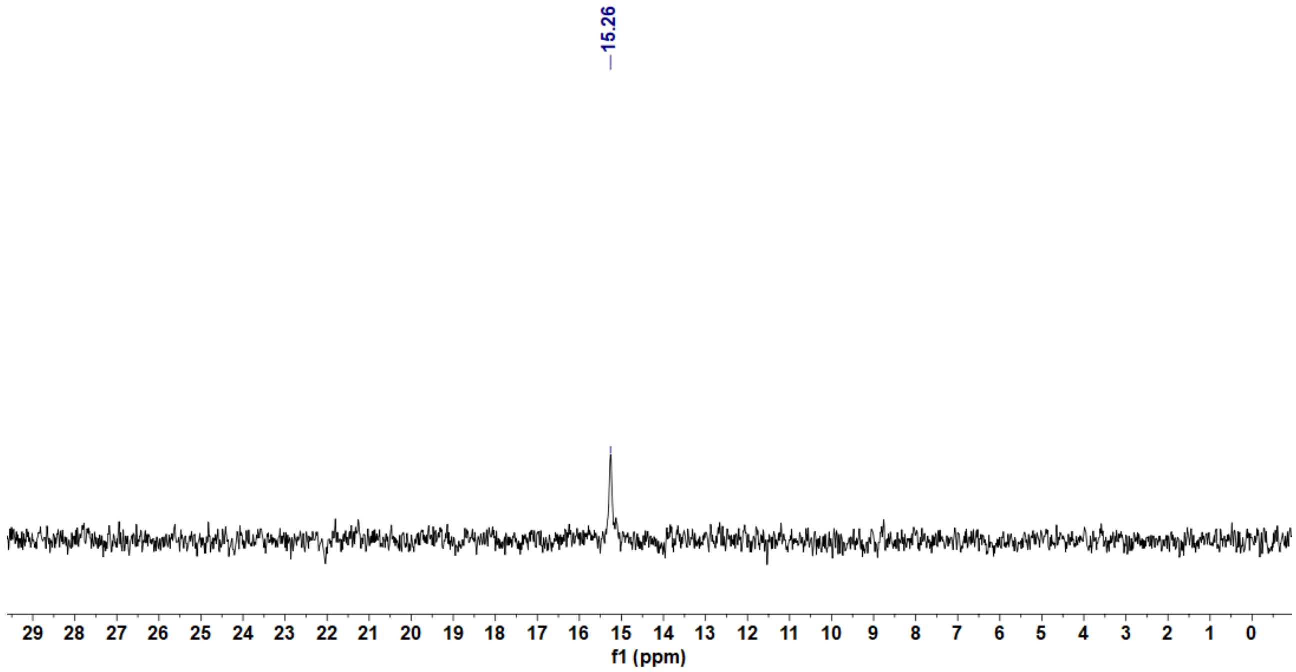

Figure S37. $^{29}Si\{^{1}H\}$ NMR (C_6D_6, 298 K) of **2**.

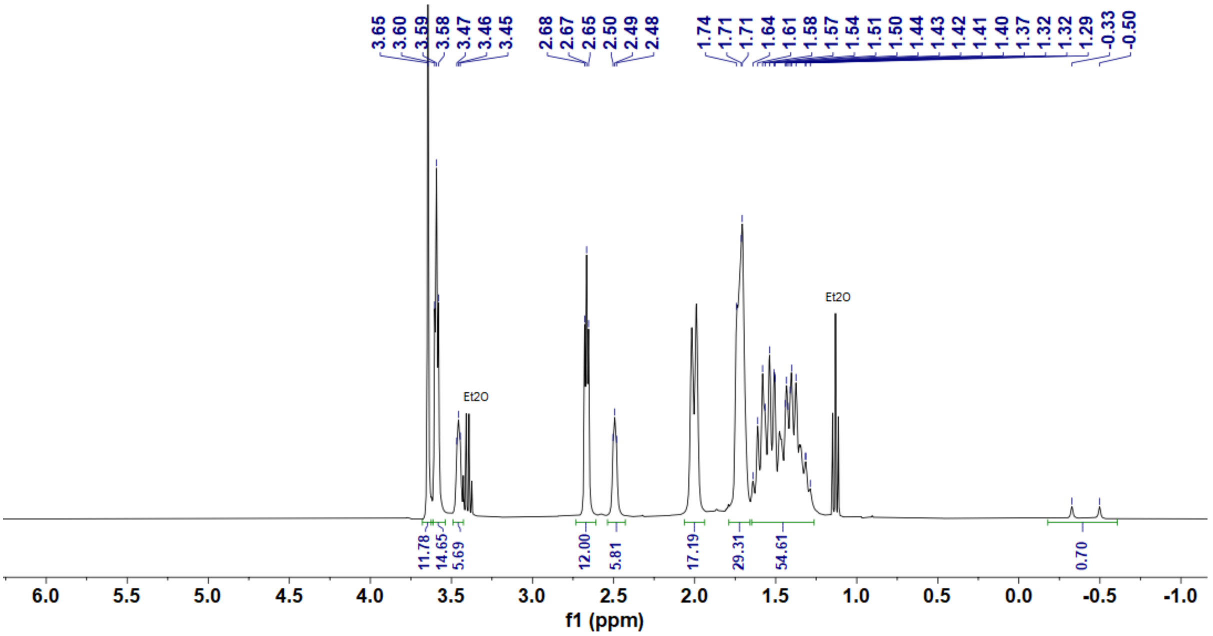

Figure S38. ^{1}H NMR (D_8-THF, 298 K) of **5**.

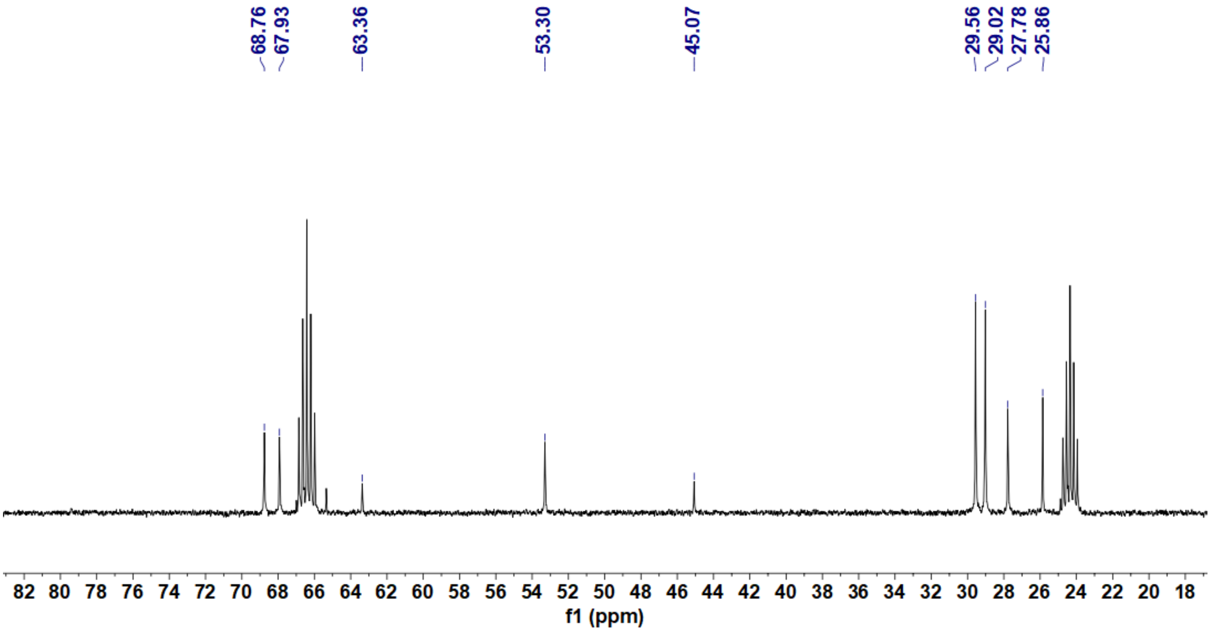

Figure S39. $^{13}C\{^{1}H\}$ NMR (D_8-THF, 298 K) of **5**.

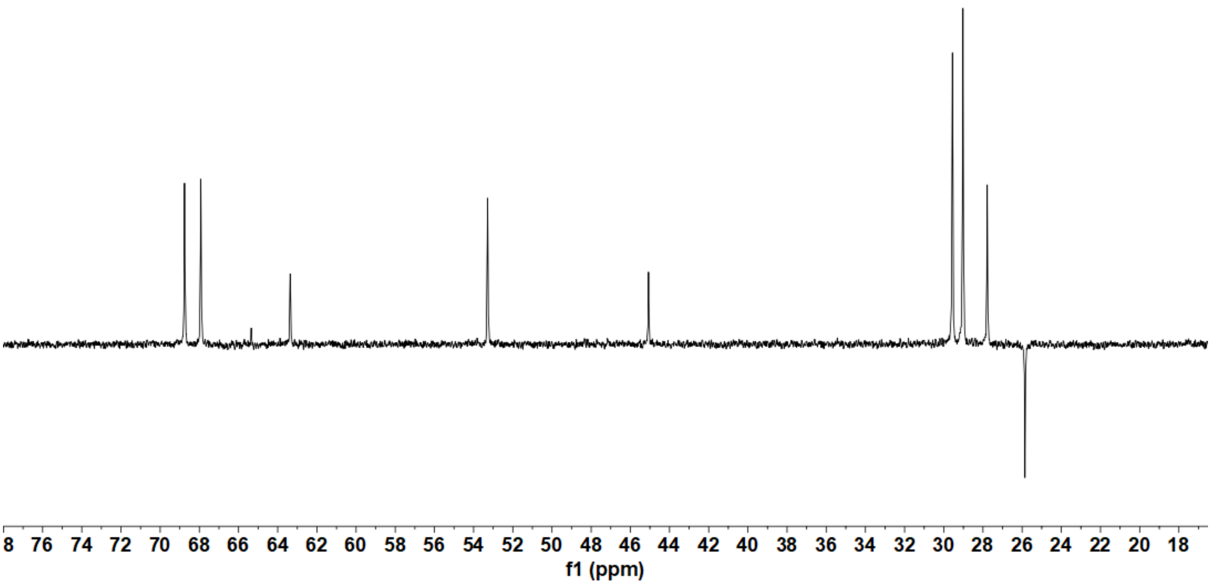

Figure S40. $^{13}C\{^1H\}$-DEPT 135 NMR (C_6D_6, 298 K) of **5**.

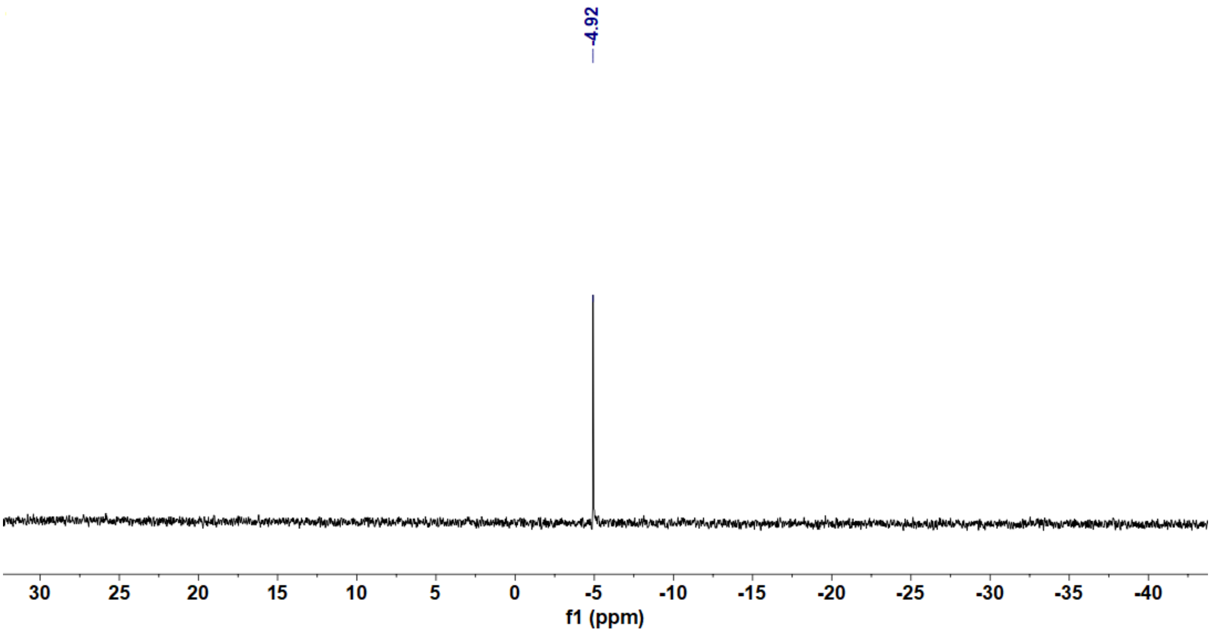

Figure S41. $^{29}Si\{^1H\}$ NMR (D_8-THF, 298 K) of **5**.

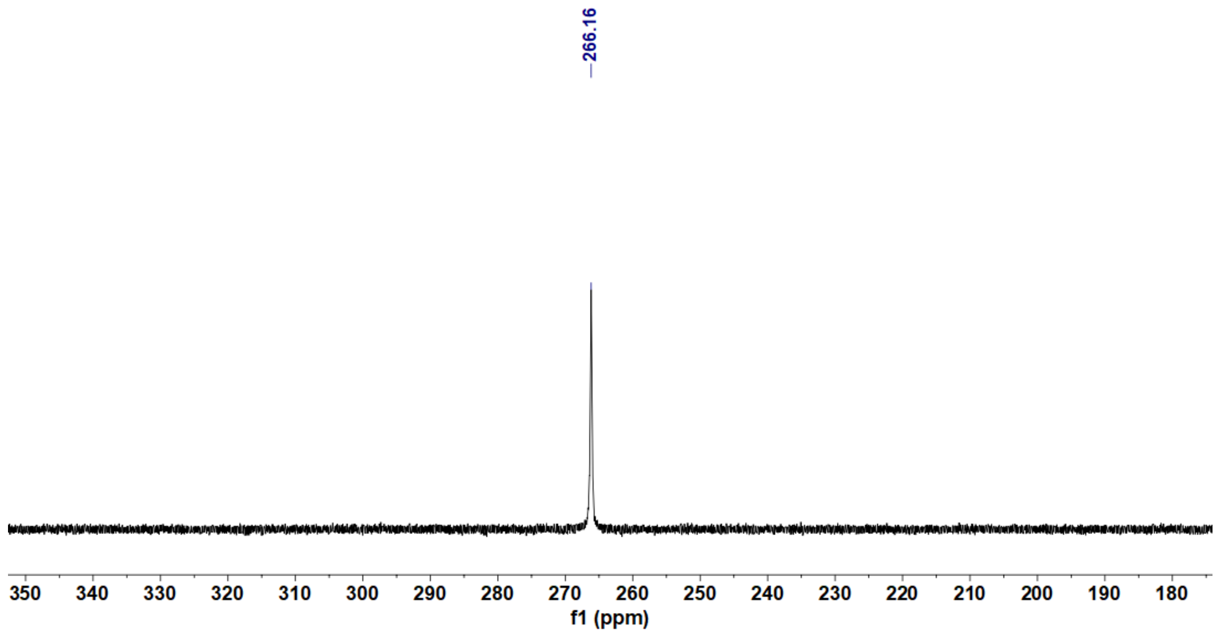

Figure S42. $^{31}P\{^{1}H$ decoupled$\}$ NMR (D_8-THF, 298 K) of **5**.

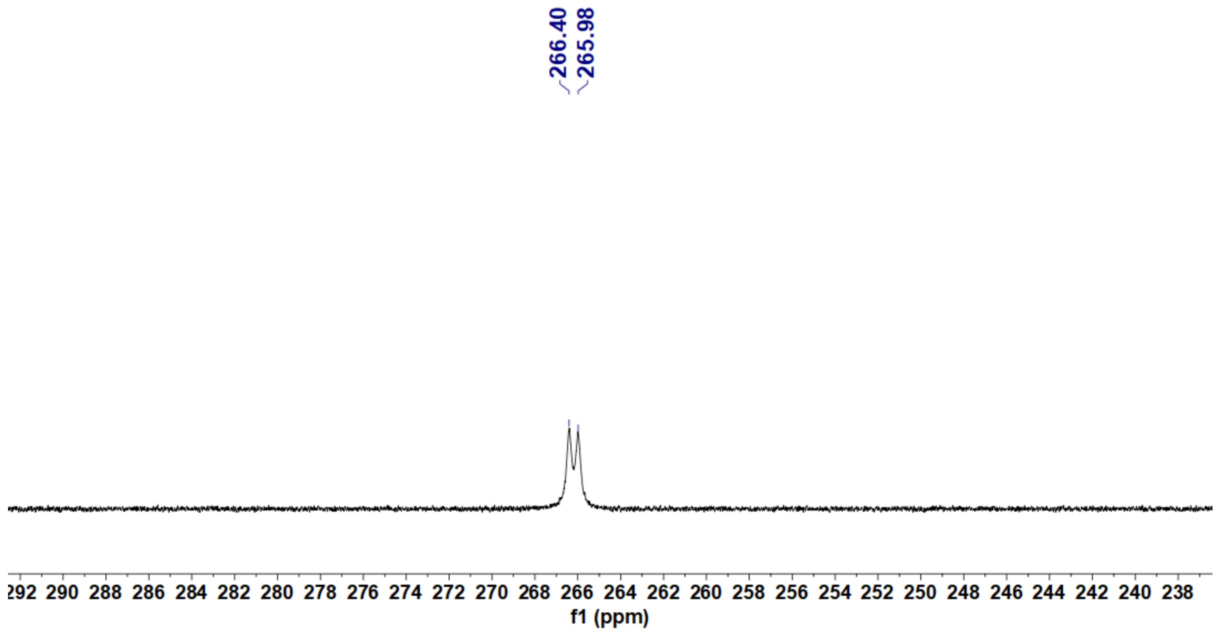

Figure S43. $^{31}P\{$Non-decoupled$\}$ NMR (D_8-THF, 298 K) of **5**.

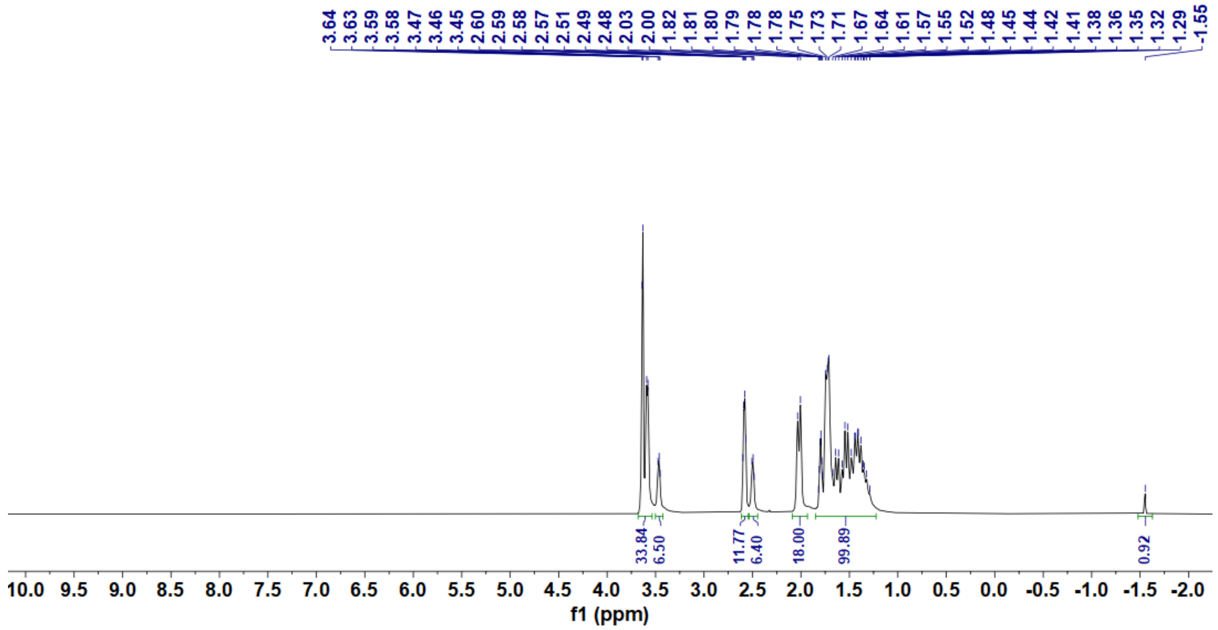

Figure S44. ^{1}H NMR (D_8-THF, 298 K) of **6**.

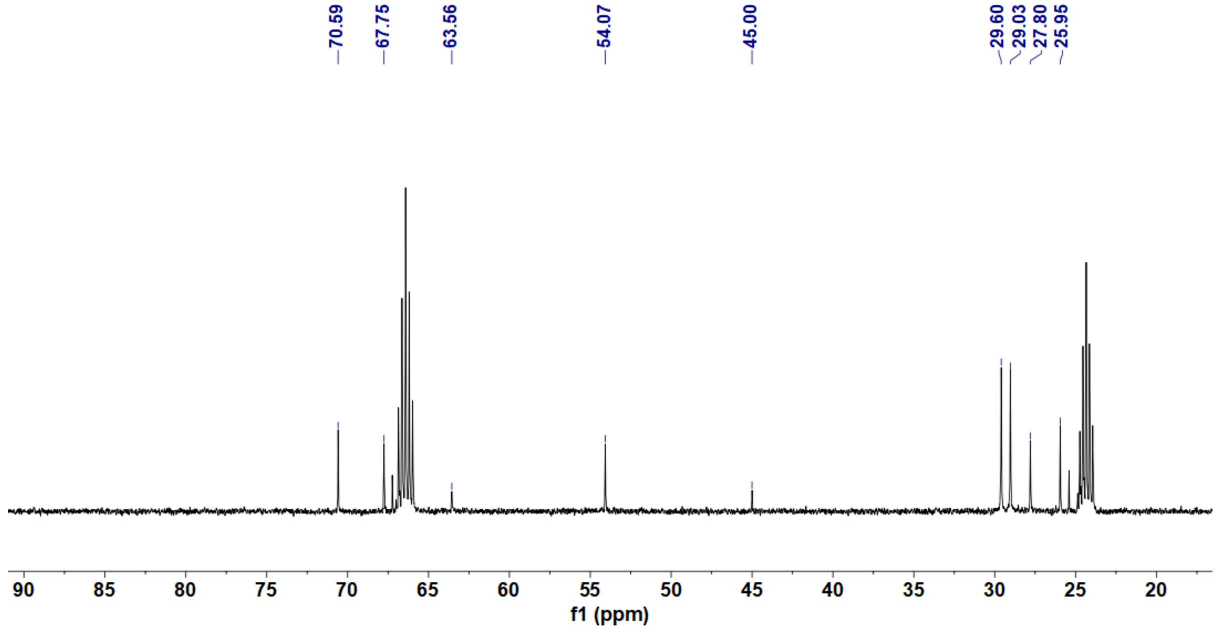

Figure S45. $^{13}C\{^{1}H\}$ NMR (D_8-THF, 298 K) of **6**.

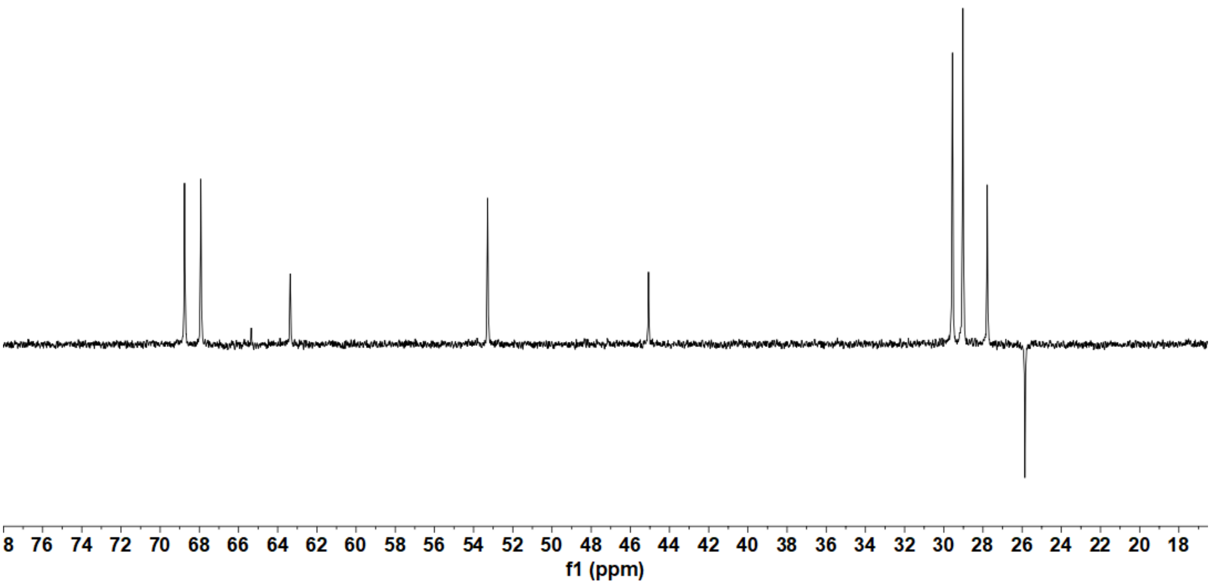

Figure S46. $^{13}C\{^1H\}$-DEPT 135 NMR (D_8-THF, 298 K) of **6**.

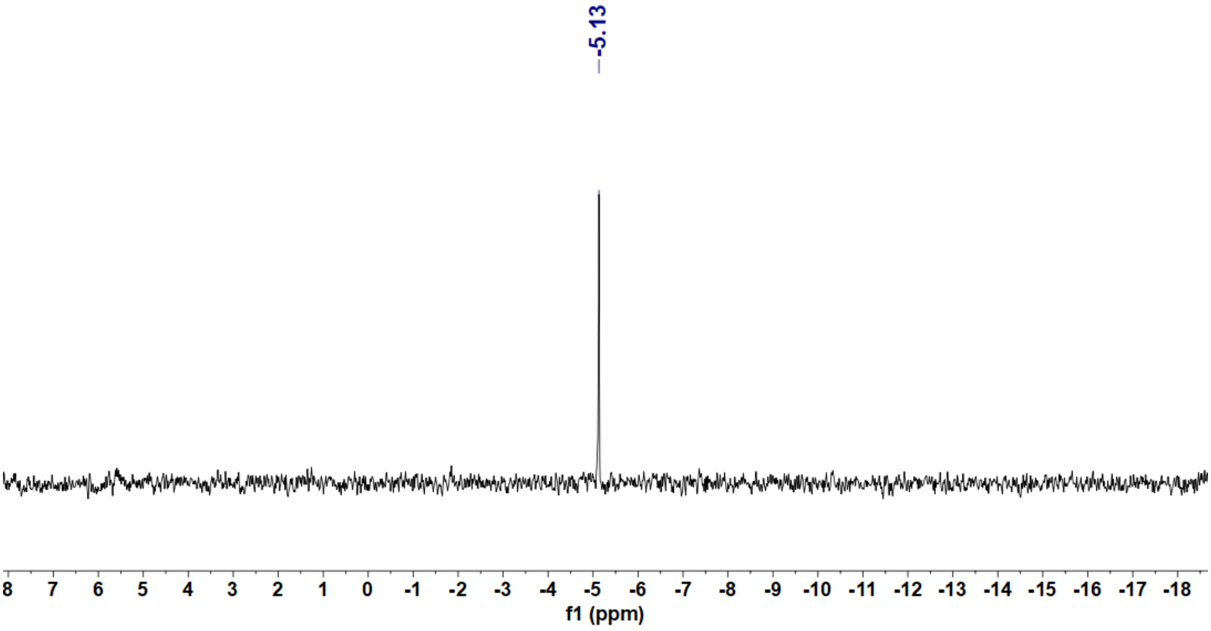

Figure S47. $^{29}Si\{^1H\}$ NMR (D_8-THF, 298 K) of **6**.

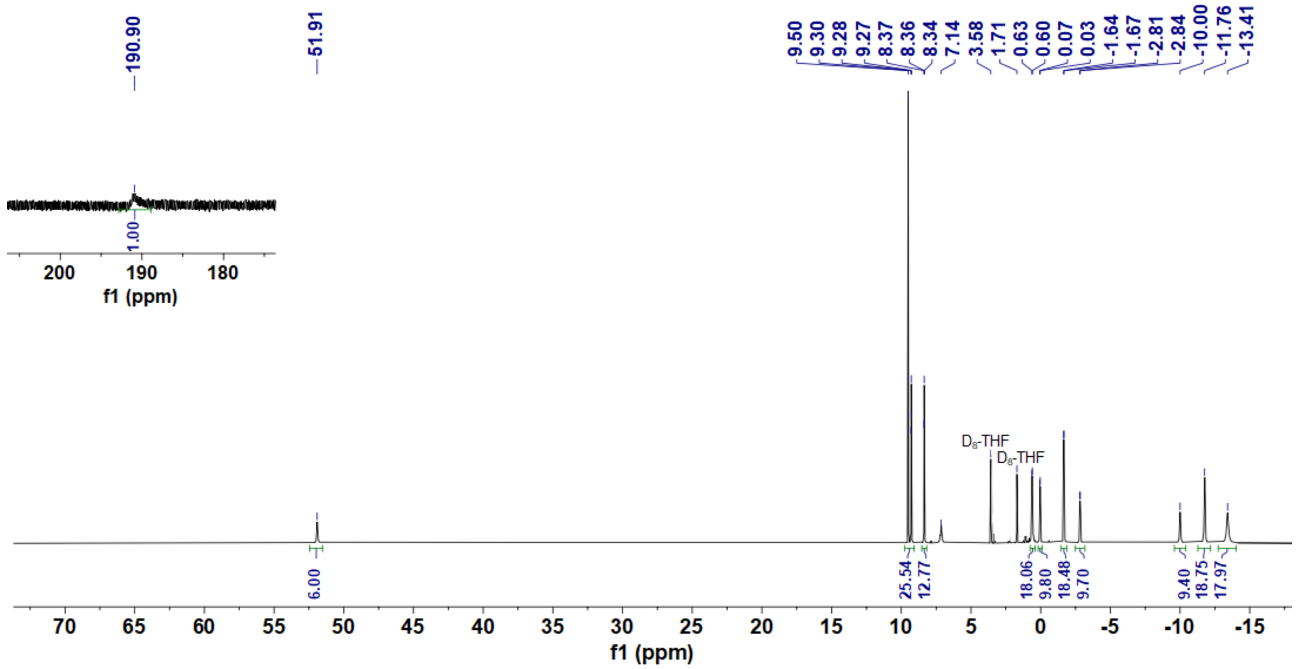

Figure S48. ^{1}H NMR (D_8-THF, 298 K) of **7**.

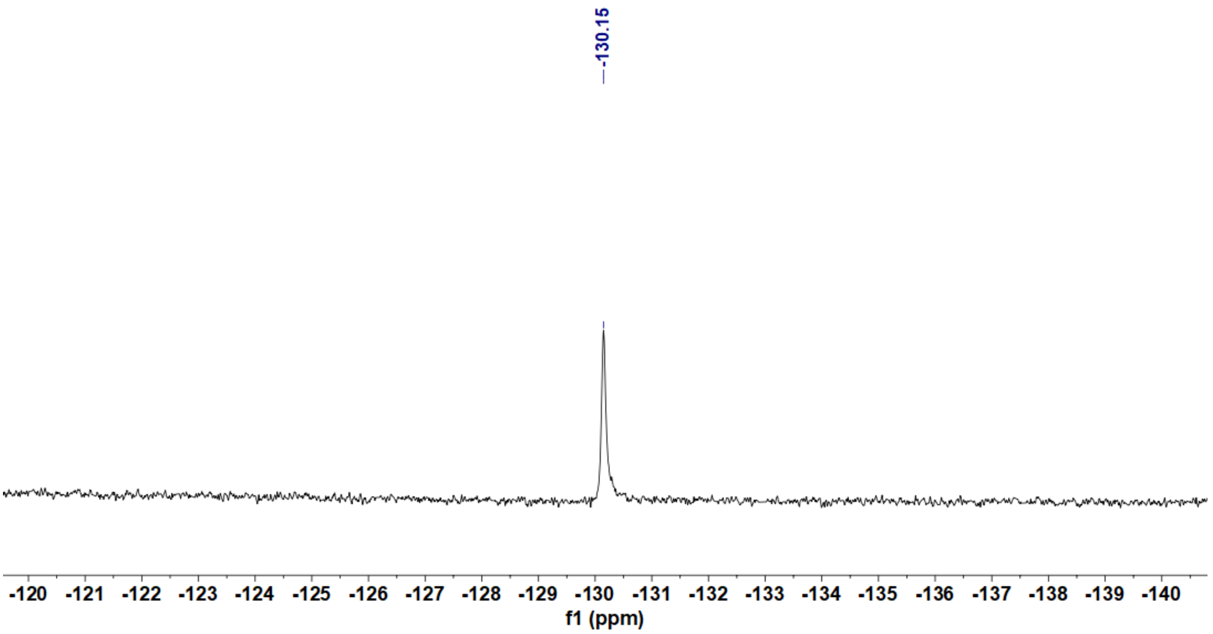

Figure S49. $^{29}Si\{^{1}H\}$ NMR (D_8-THF, 298 K) of **7**.

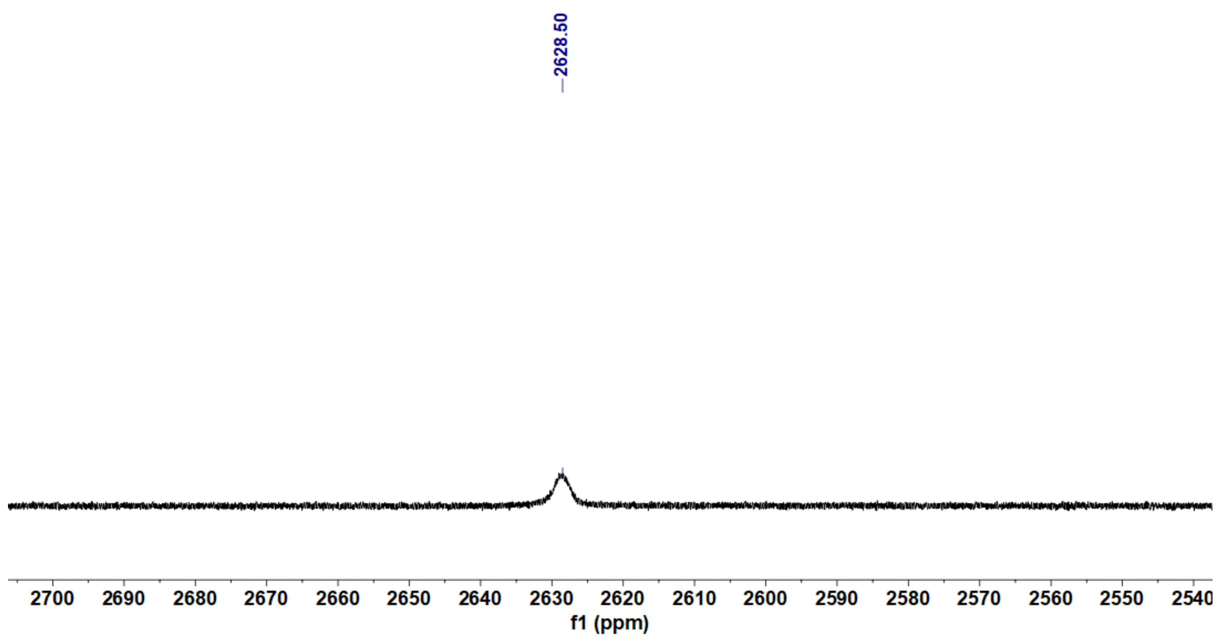

Figure S50. ^{31}P NMR (D_8-THF, 298 K) of **7**.

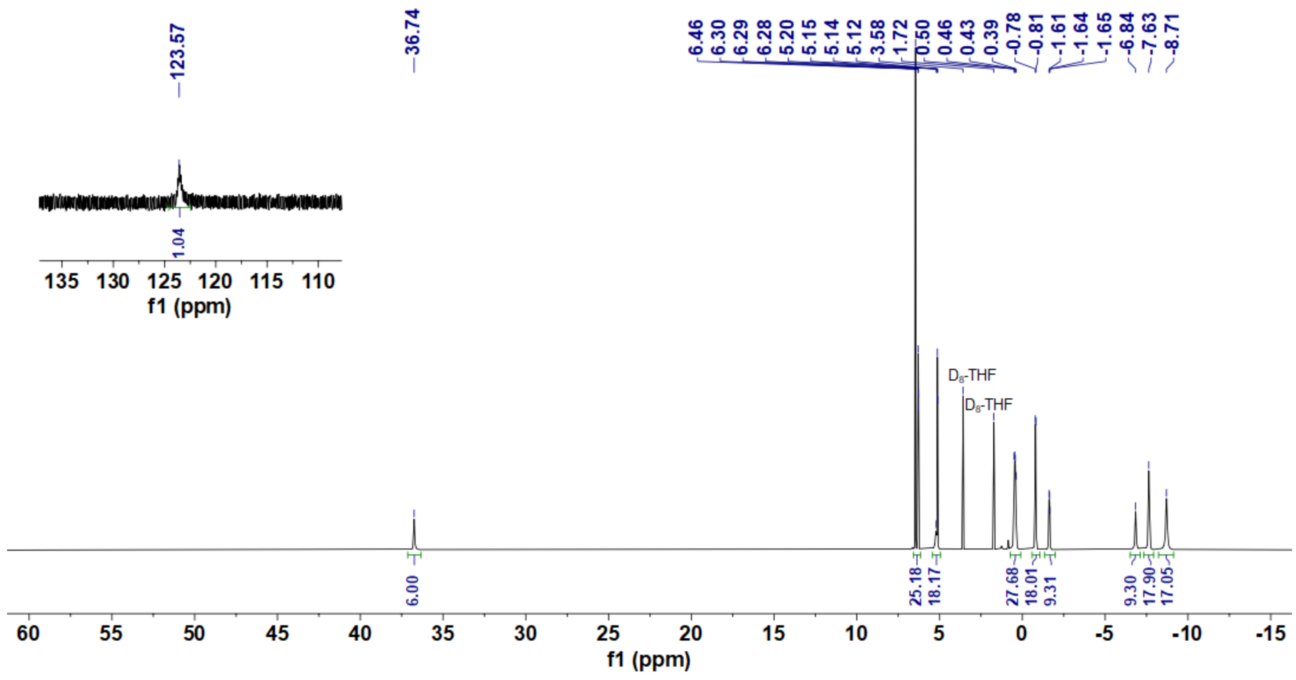

Figure S51. ^{1}H NMR (D_8-THF, 298 K) of **8**.

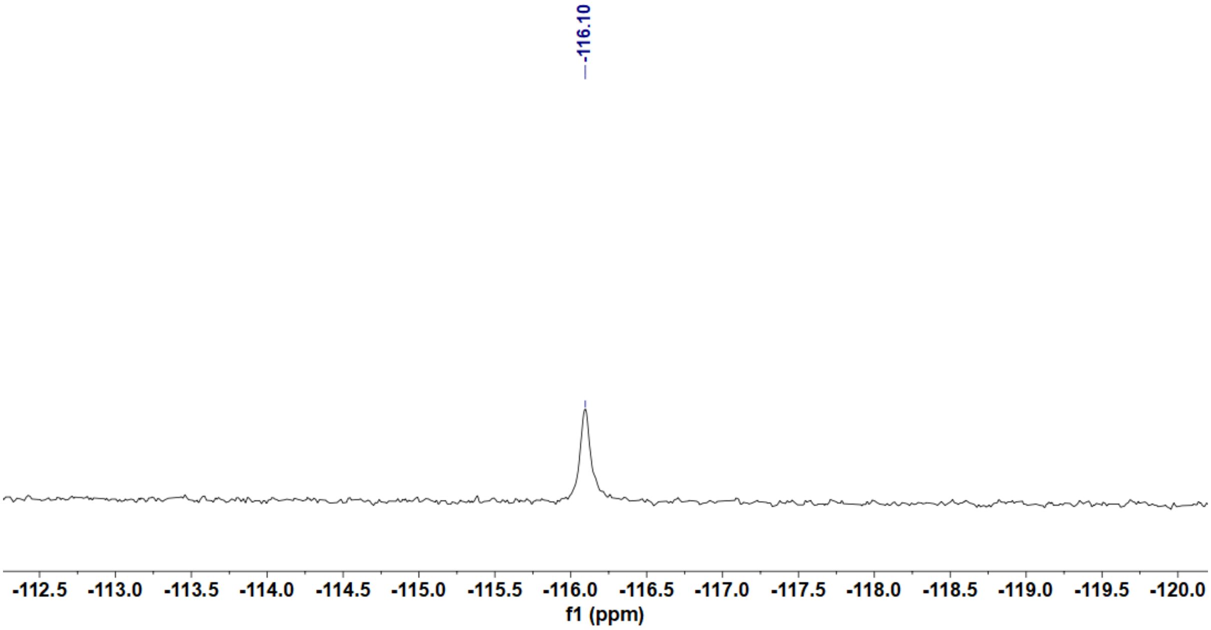

Figure S52. ^{29}Si{^{1}H} NMR (D_8-THF, 298 K) of **8**.

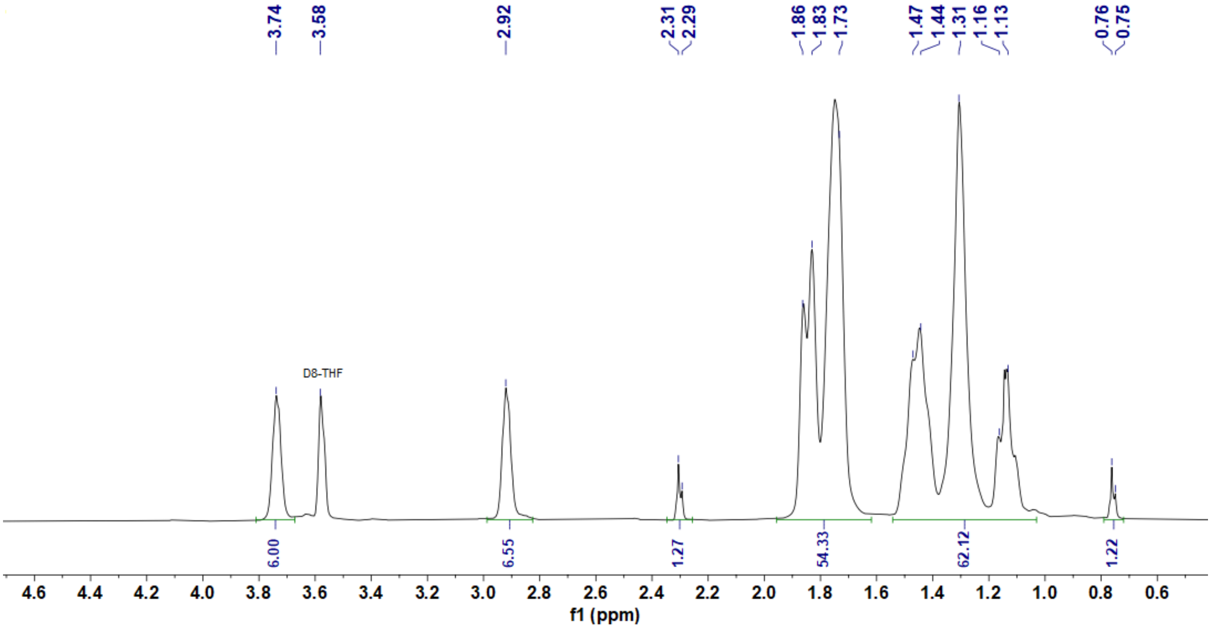

Figure S53. ^{1}H NMR (D_8-THF, 298 K) of **9**.

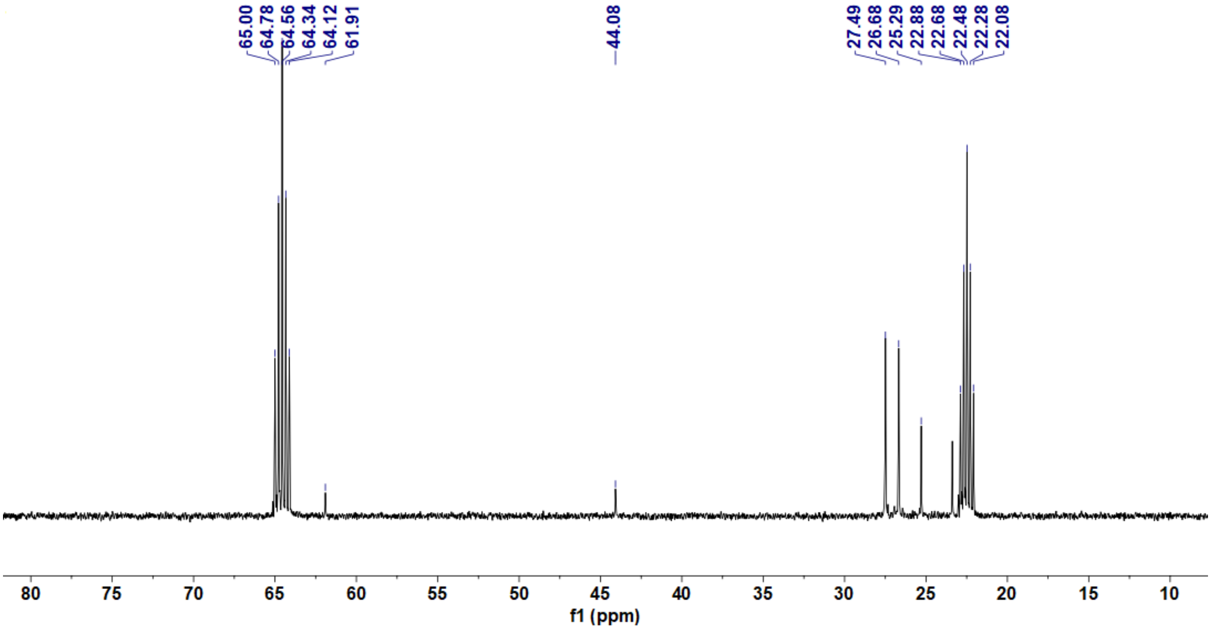

Figure S54. $^{13}C\{^1H\}$ NMR (D_8-THF, 298 K) of **9**.

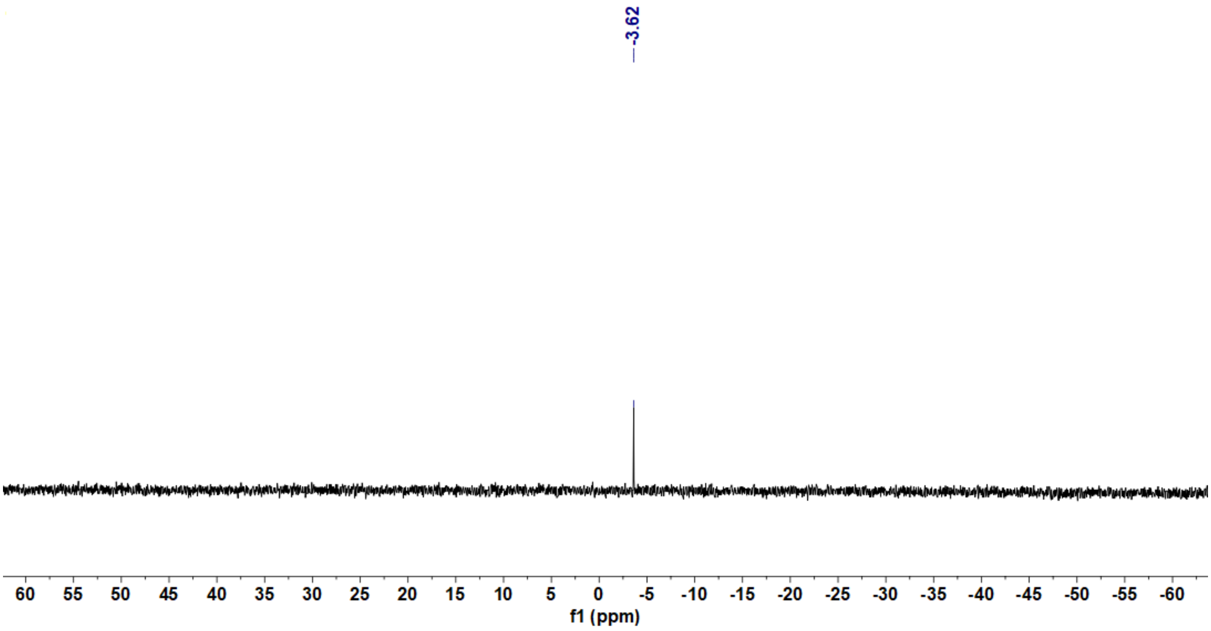

Figure S55. $^{29}Si\{^1H\}$ NMR (D_8-THF, 298 K) of **9**.

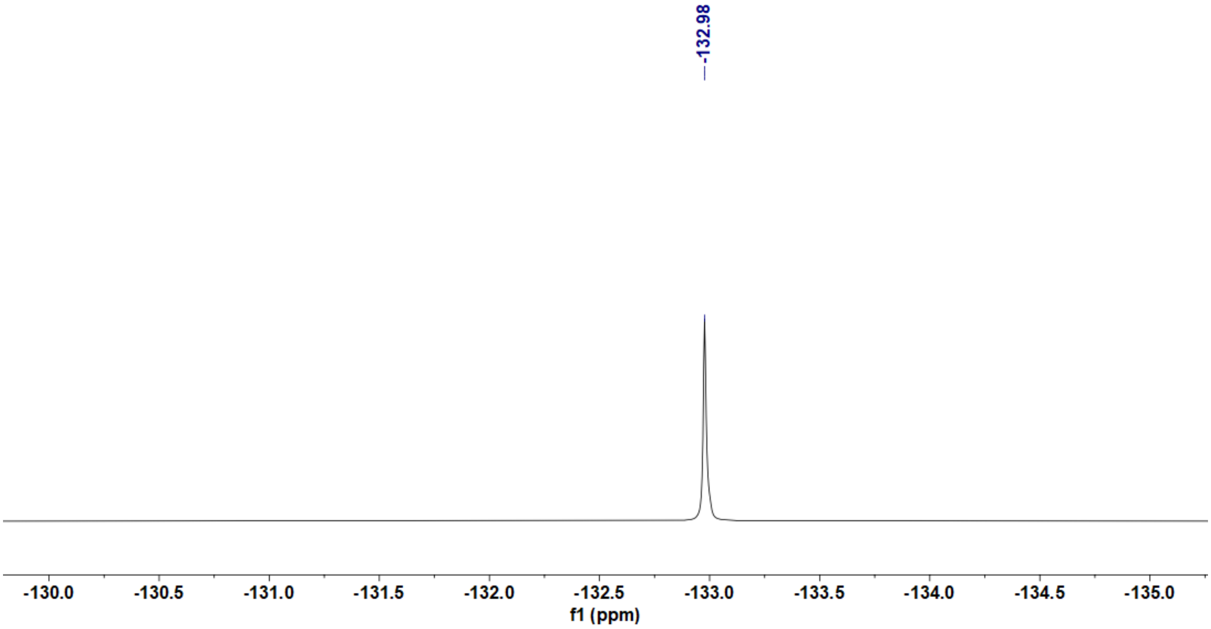

Figure S56. $^{31}P\{^{1}H$ decoupled$\}$ NMR (D_8-THF, 298 K) of **9**.

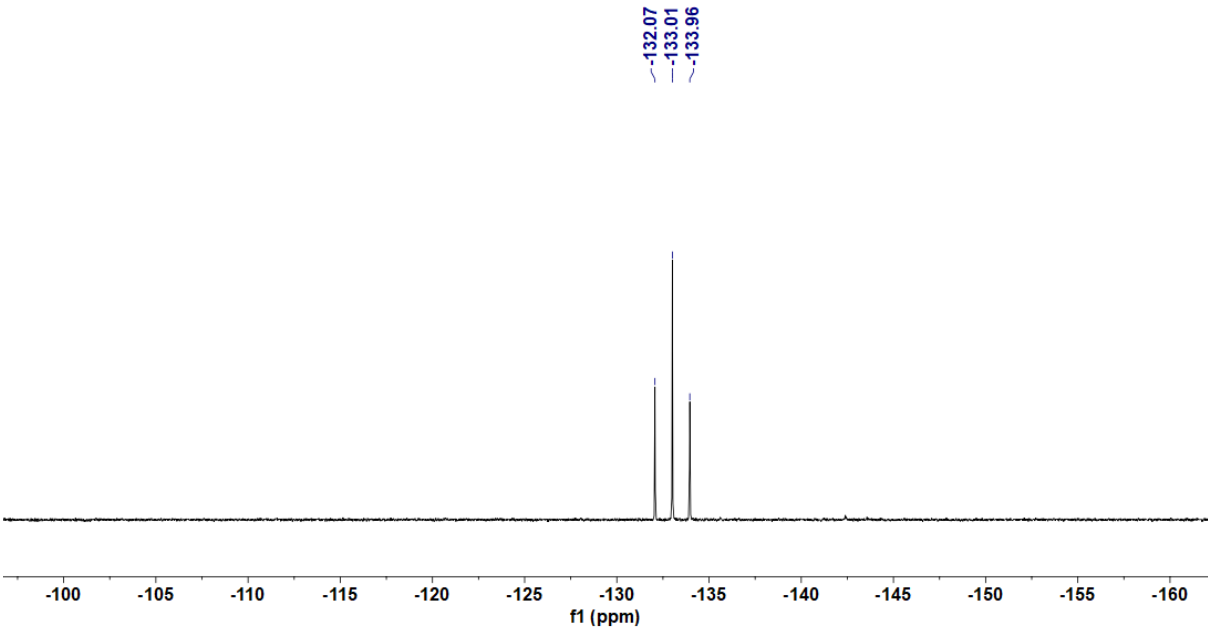

Figure S57. $^{31}P\{$Non-decouple $^{1}H\}$ NMR (D_8-THF, 298 K) of **9**.

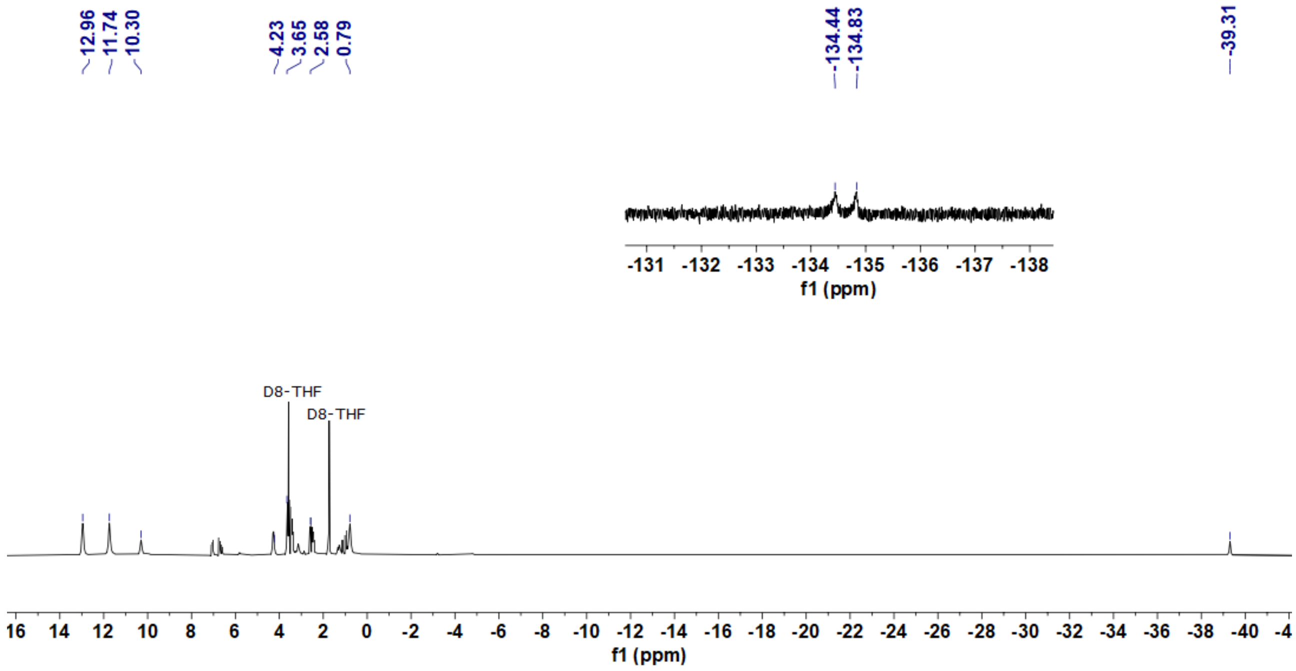

Figure S58. ^{1}H NMR (D_8-THF, 298 K) of **11**.

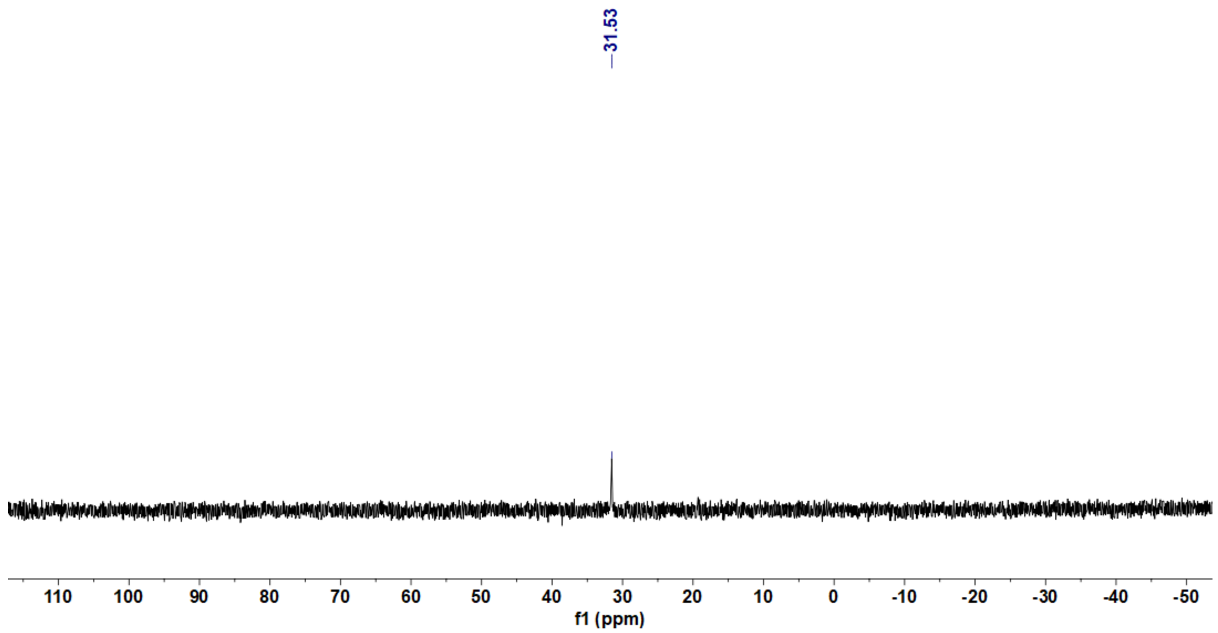

Figure S59. ^{29}Si{^{1}H} NMR (D_8-THF, 298 K) of **11**.

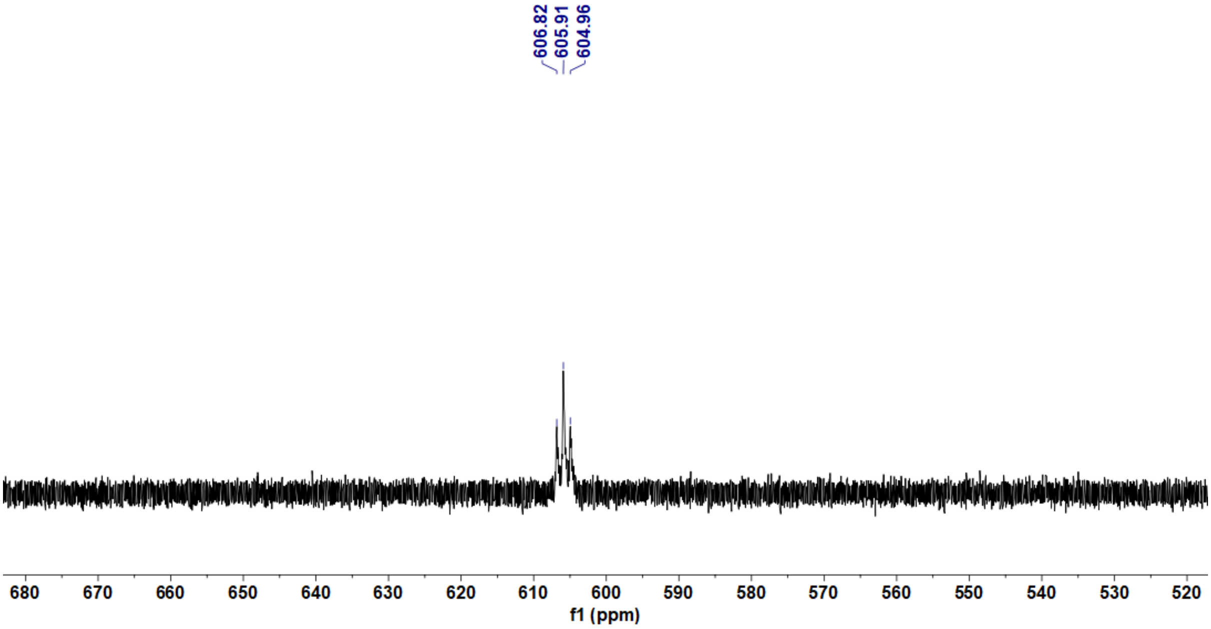

Figure S60. $^{31}P\{^{1}H$ Coupled$\}$ NMR(D_8-THF, 298 K) of **11**.

UV/Vis/NIR Spectra

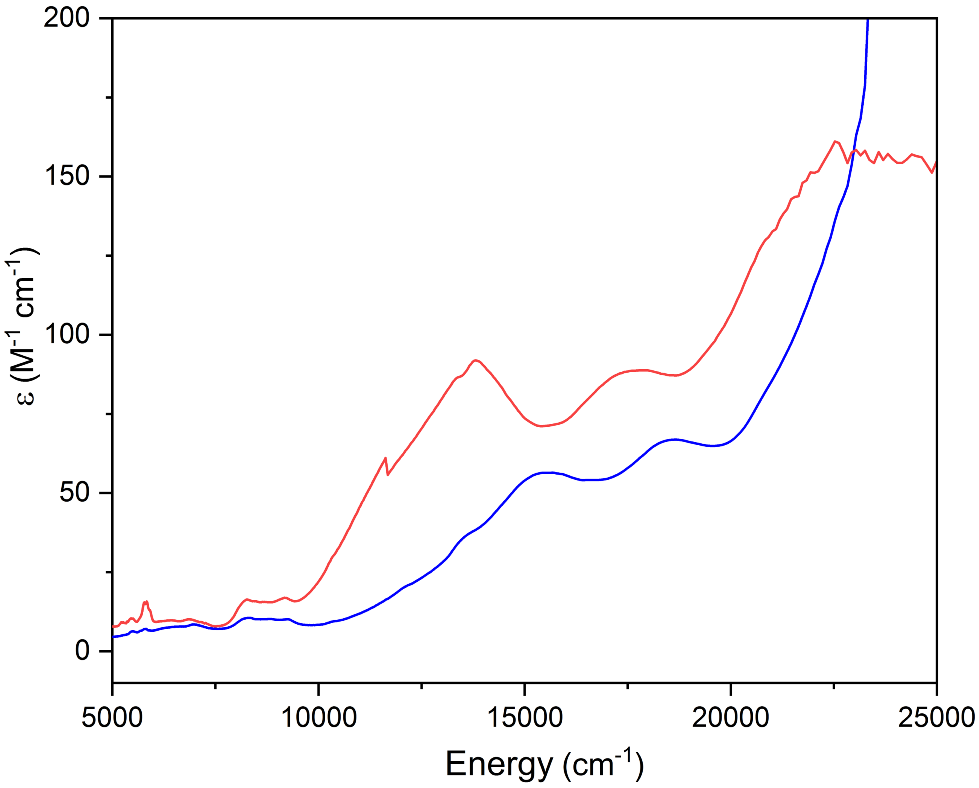

Figure 61. UV/Vis/NIR spectra of **7** (blue, 25 mM) and **8** (red, 25 mM) in THF over the range 5000-25000 cm^{-1}.

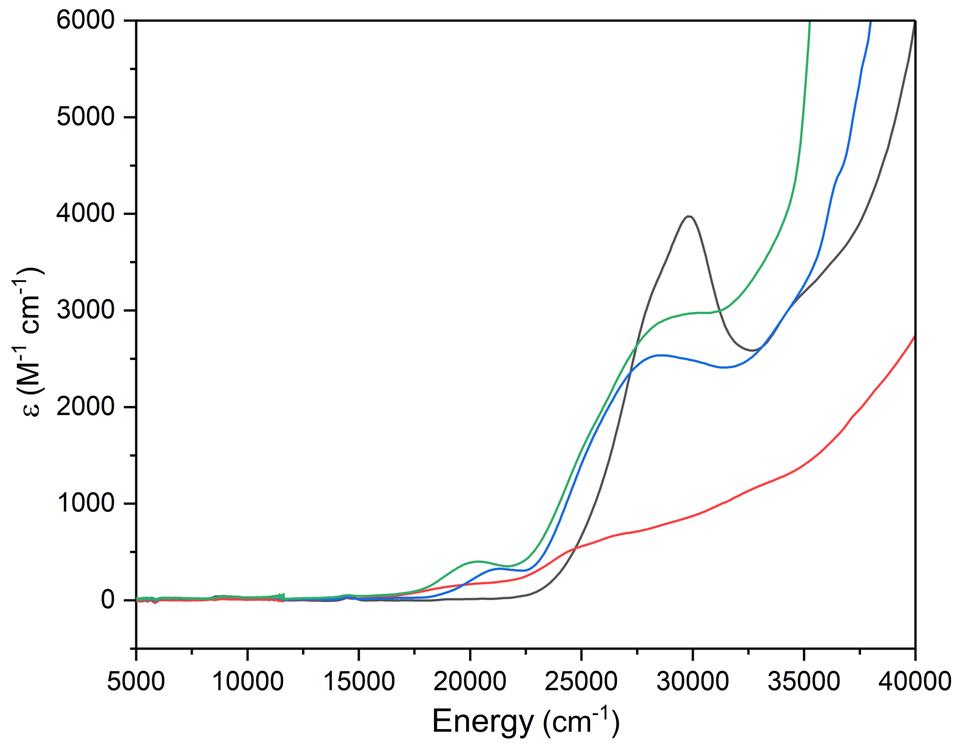

Figure 62. UV/Vis/NIR spectra of **2** (black), **4** (red), **11** (blue), and **12** (green) recorded in a saturated solution in THF (~6 mM) due to the poor solubility of these complexes in common solvents over the range 5000-40000 cm^{-1}.

Magnetism Data

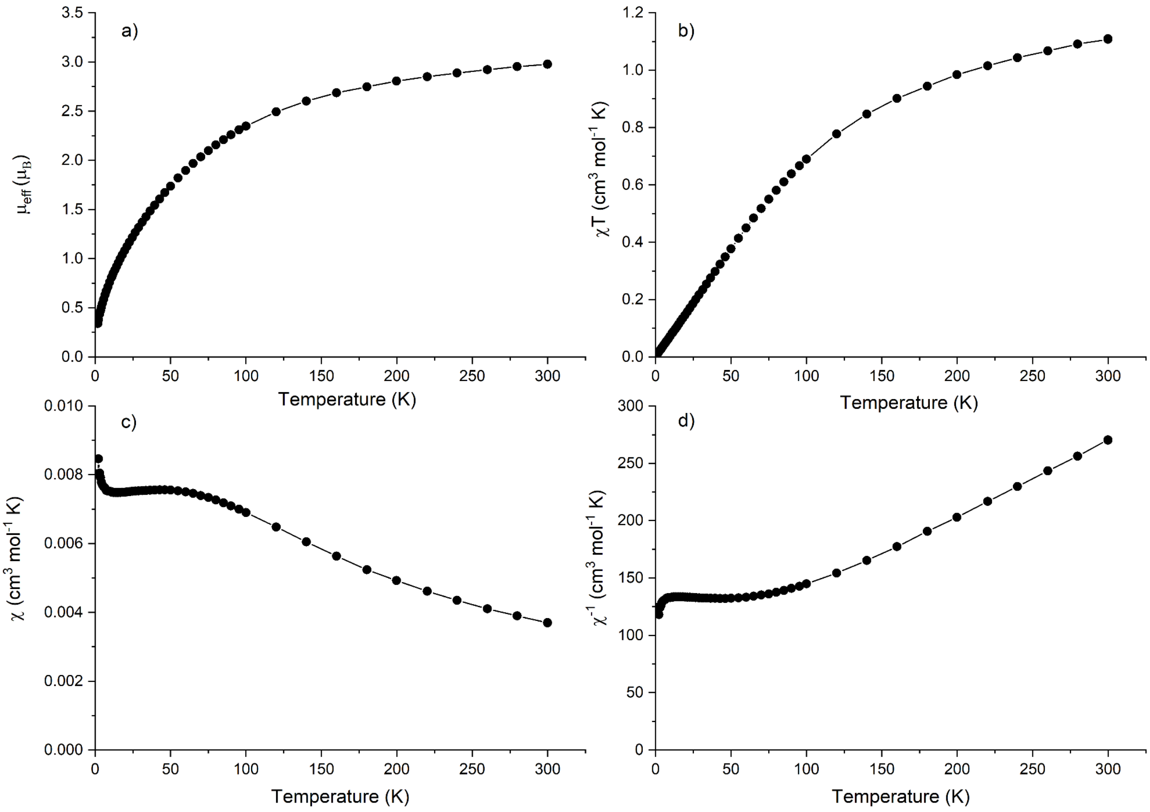

Figure S63. Variable-temperature SQUID magnetometry of **2** over the temperature range 1.8-300 K: a) μ_{eff} vs T; b) χT vs T; c) χ vs T; d) 1/χ vs T. μ_{eff} (300 K) = 2.98 μ_B, μ_{eff} (1.8 K) = 0.34 μ_B.

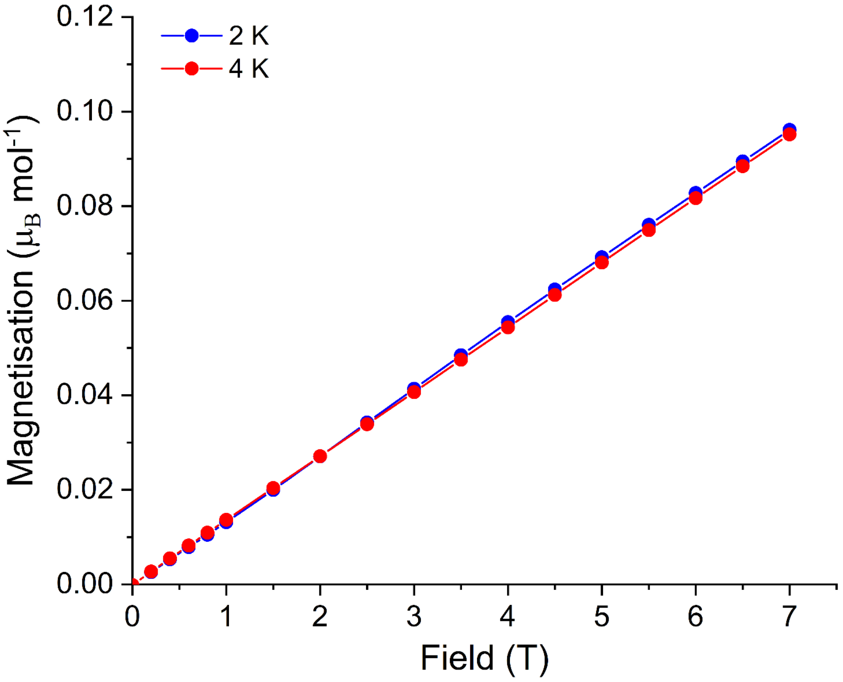

Figure S64. Magnetisation vs Field data for **2** at 2 and 4 K.

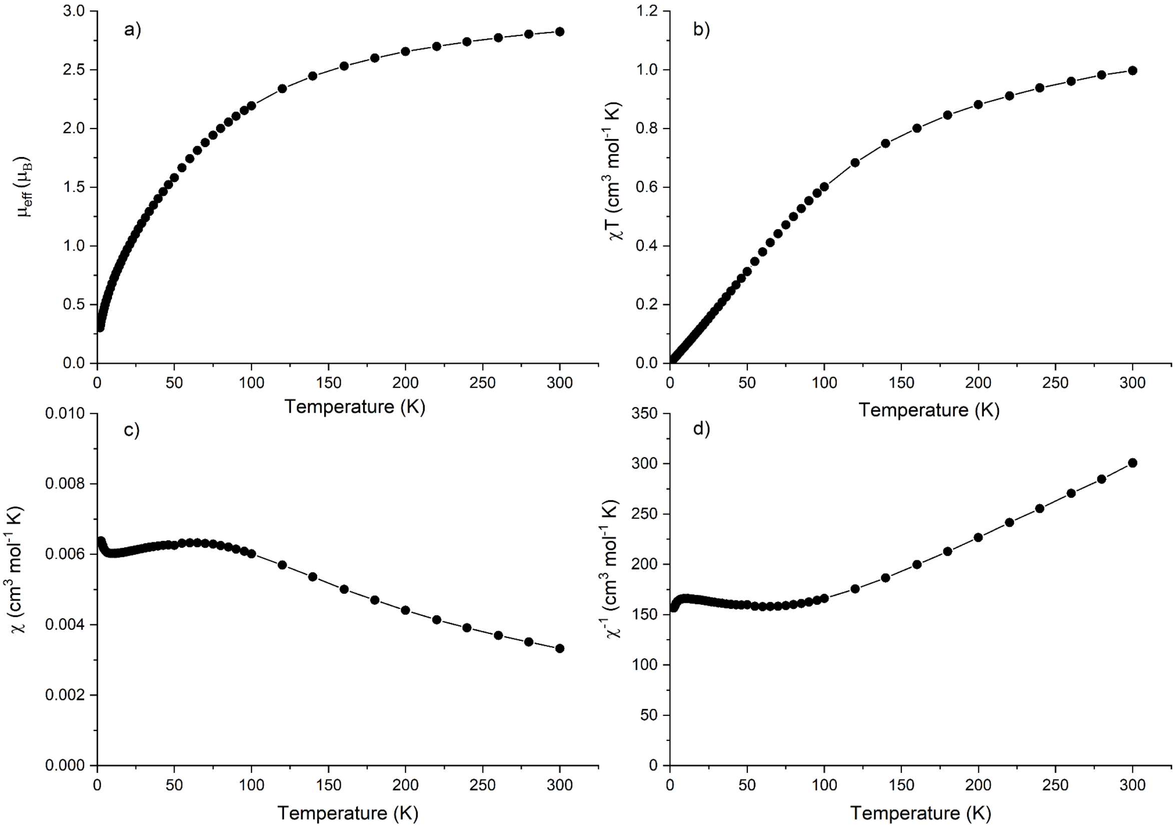

Figure S65. Variable-temperature SQUID magnetometry of **4** over the temperature range 1.8-300 K: a) μ_{eff} vs T; b) χT vs T; c) χ vs T; d) 1/χ vs T. μ_{eff} (300 K) = 2.82 μ_B, μ_{eff} (1.8 K) = 0.30 μ_B.

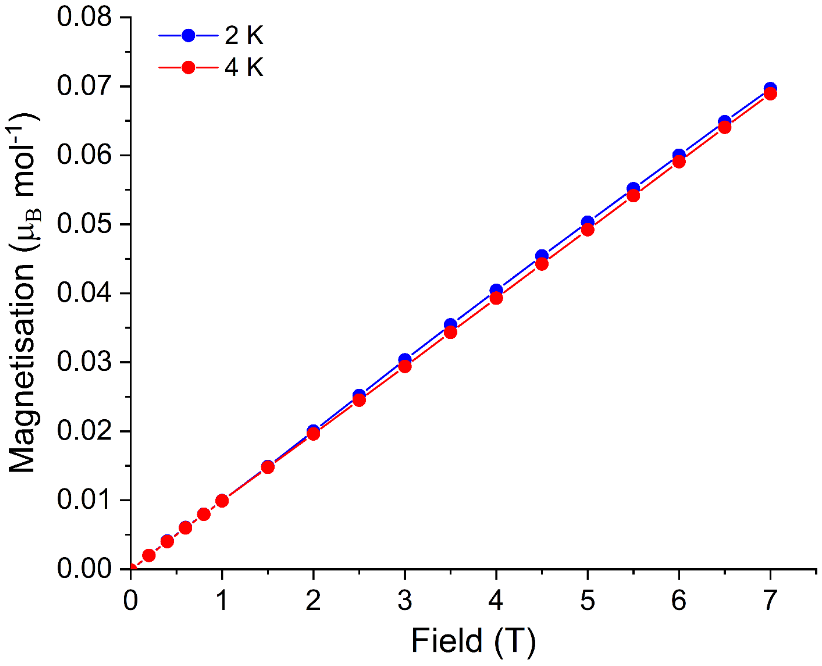

Figure S66. Magnetisation vs Field data for **4** at 2 and 4 K.

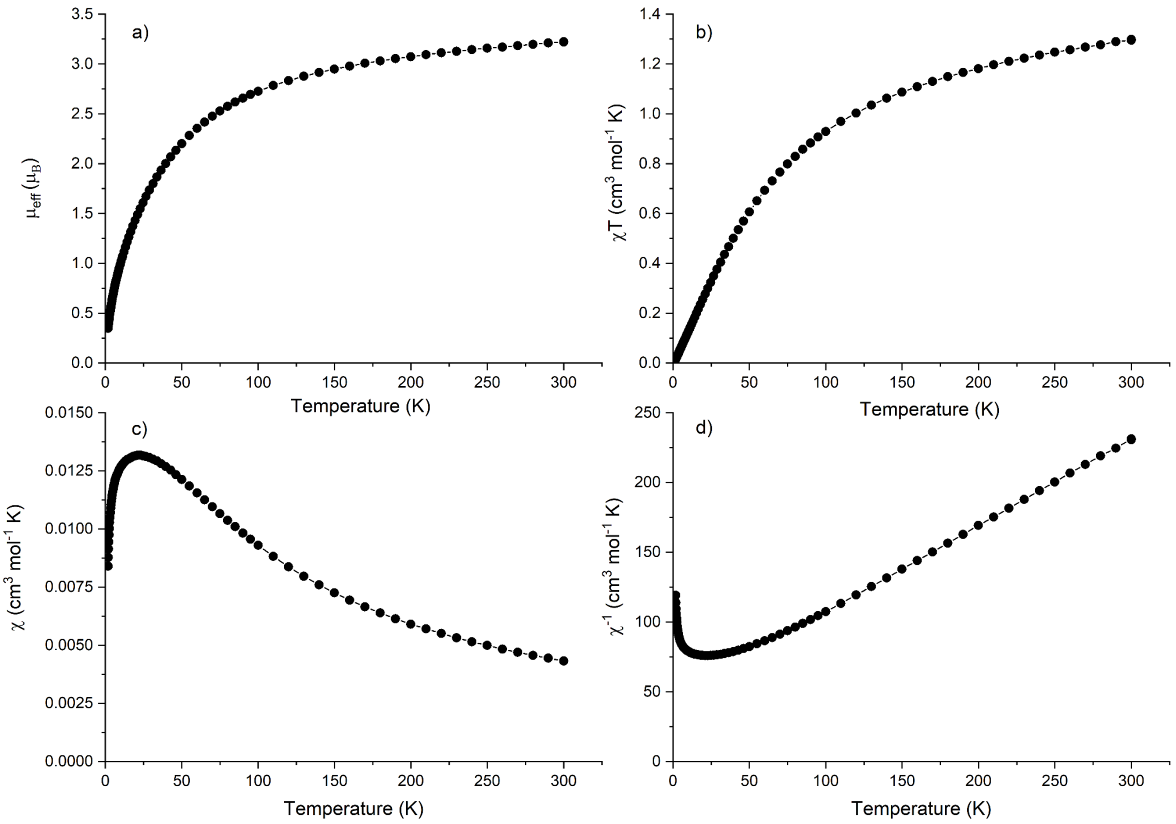

Figure S67. Variable-temperature SQUID magnetometry of **7** over the temperature range 1.8-300 K: a) μ_{eff} vs T; b) χT vs T; c) χ vs T; d) 1/χ vs T. μ_{eff} (300 K) 3.22 μ_B, μ_{eff} (1.8 K) 0.35 μ_B.

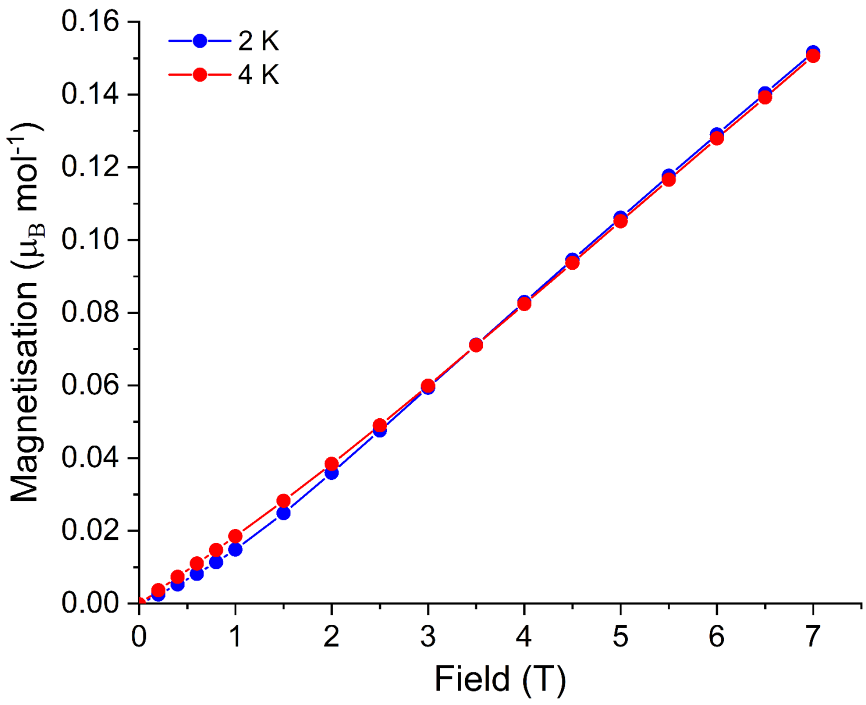

Figure S68. Magnetisation vs Field data for **7** at 2 and 4 K.

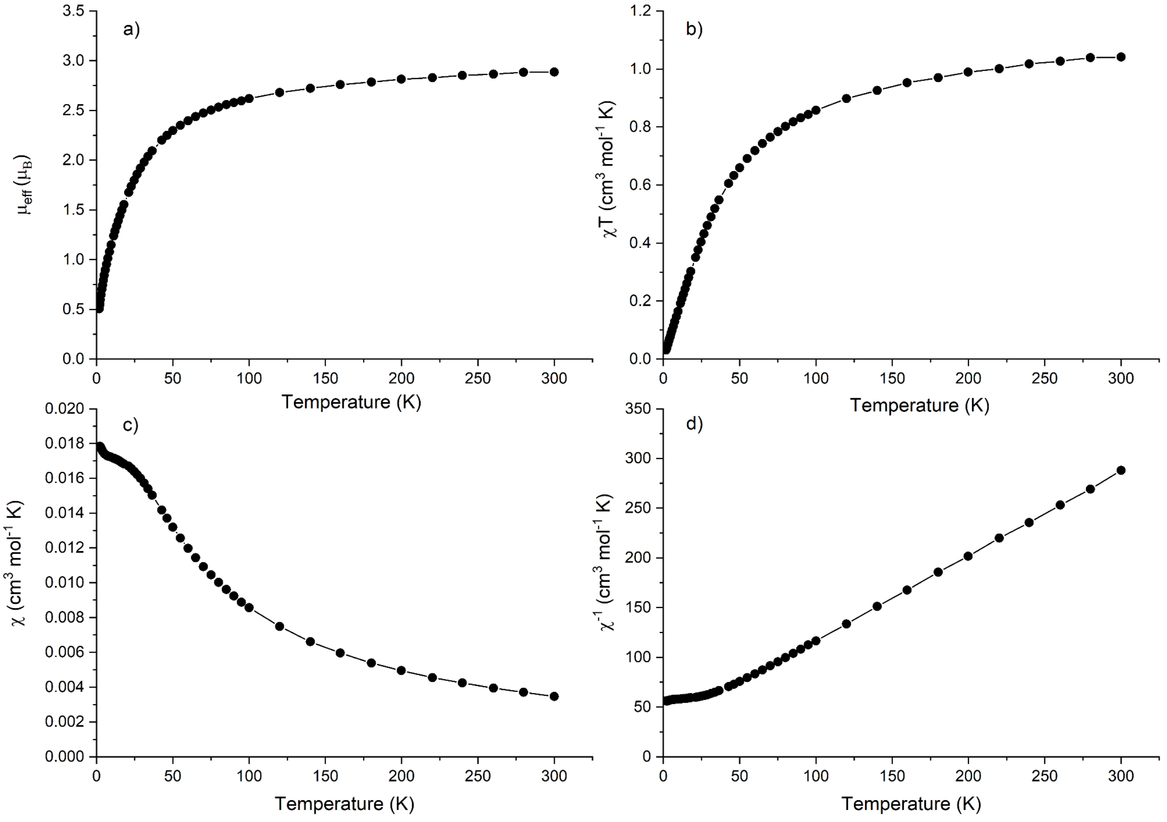

Figure S69. Variable-temperature SQUID magnetometry of **8** over the temperature range 1.8-300 K: a) μ_{eff} vs T; b) χT vs T; c) χ vs T; d) 1/χ vs T. μ_{eff} (300 K) 2.89 μ_B, μ_{eff} (1.8 K) 0.51 μ_B.

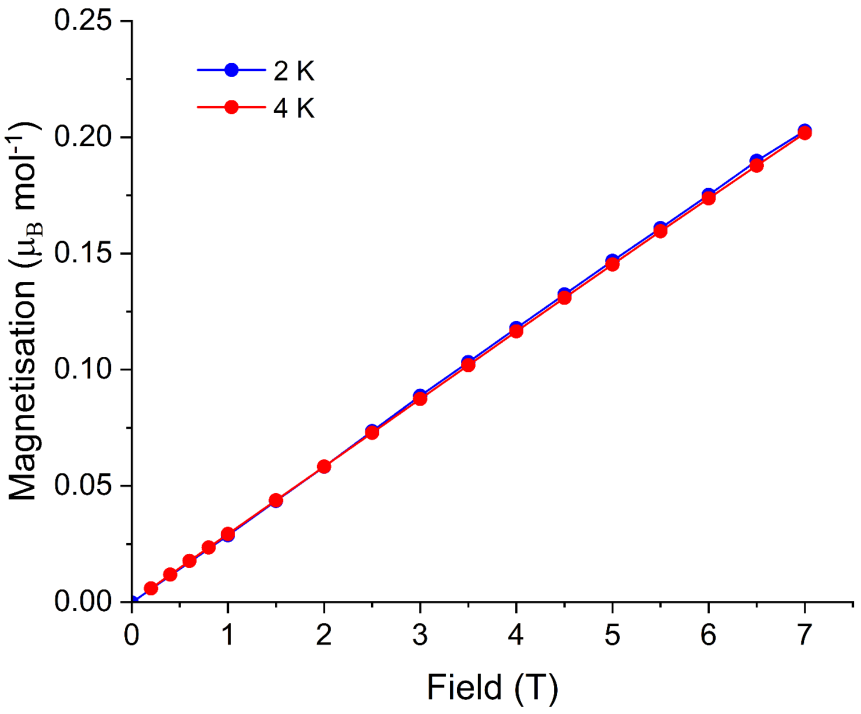

Figure S70. Magnetisation vs Field data for **8** at 2 and 4 K.

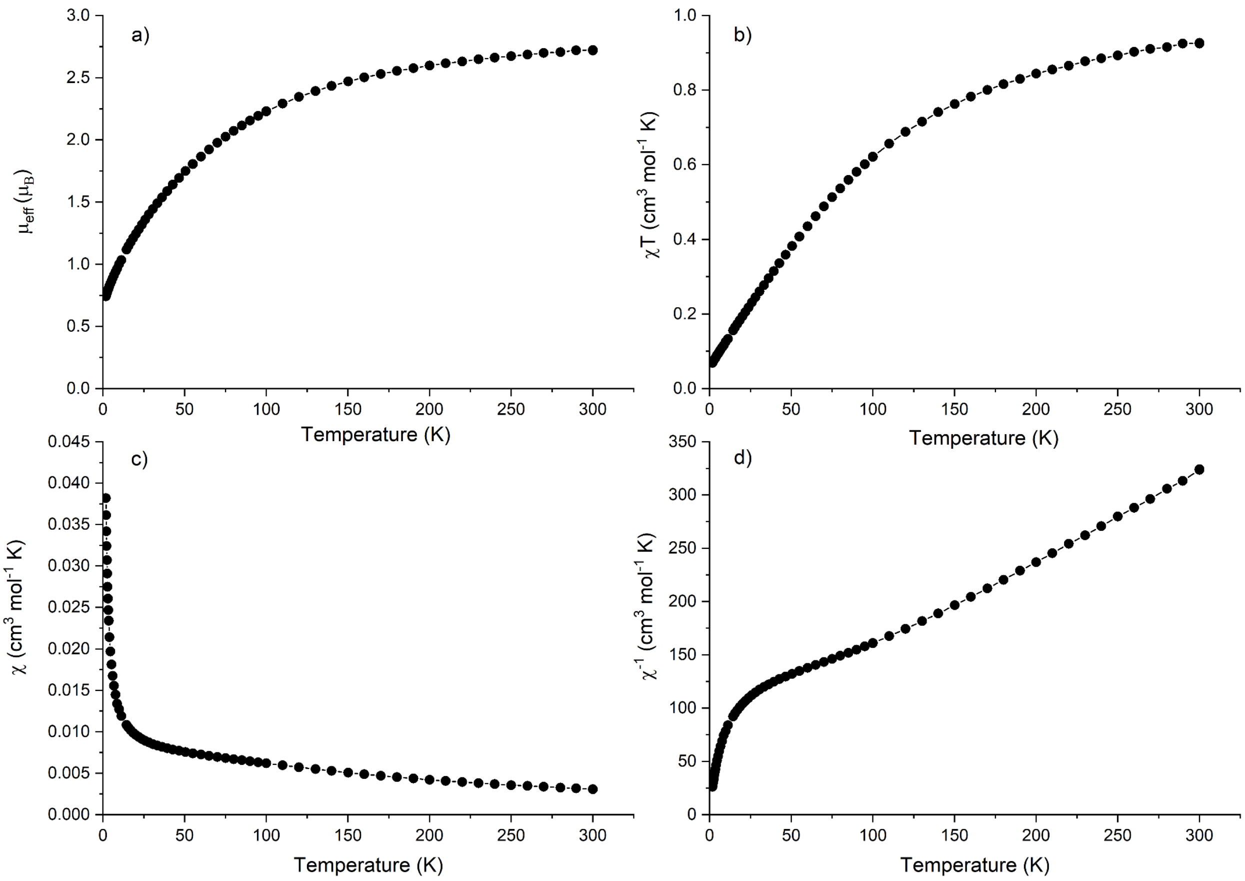

Figure S71. Variable-temperature SQUID magnetometry of **11** over the temperature range 1.8-300 K: a) μ_{eff} vs T; b) χT vs T; c) χ vs T; d) 1/χ vs T. μ_{eff} (300 K) 2.72 μ_B, μ_{eff} (1.8 K) 0.74 μ_B.

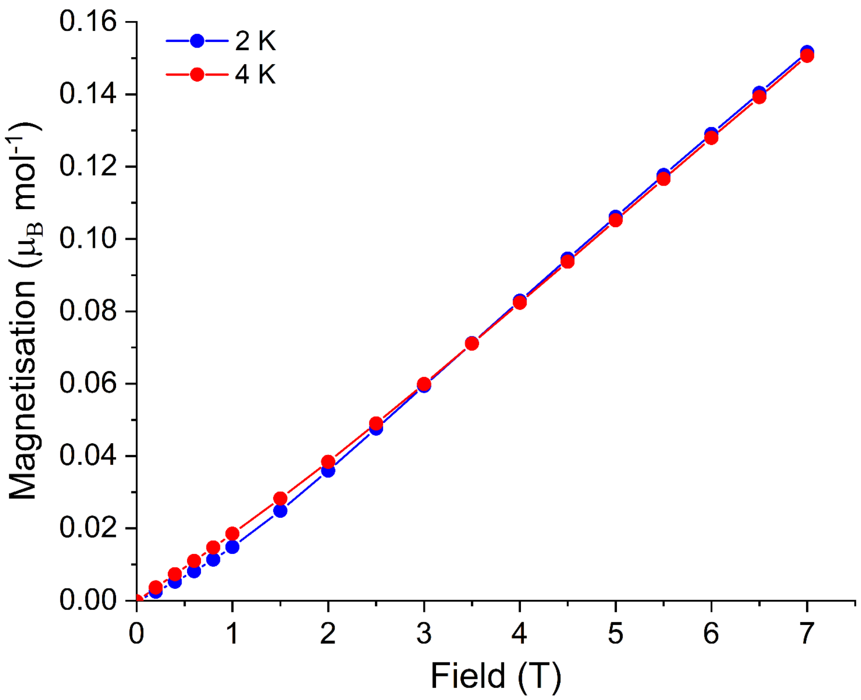

Figure S72. Magnetisation vs Field data for **11** at 2 and 4 K.

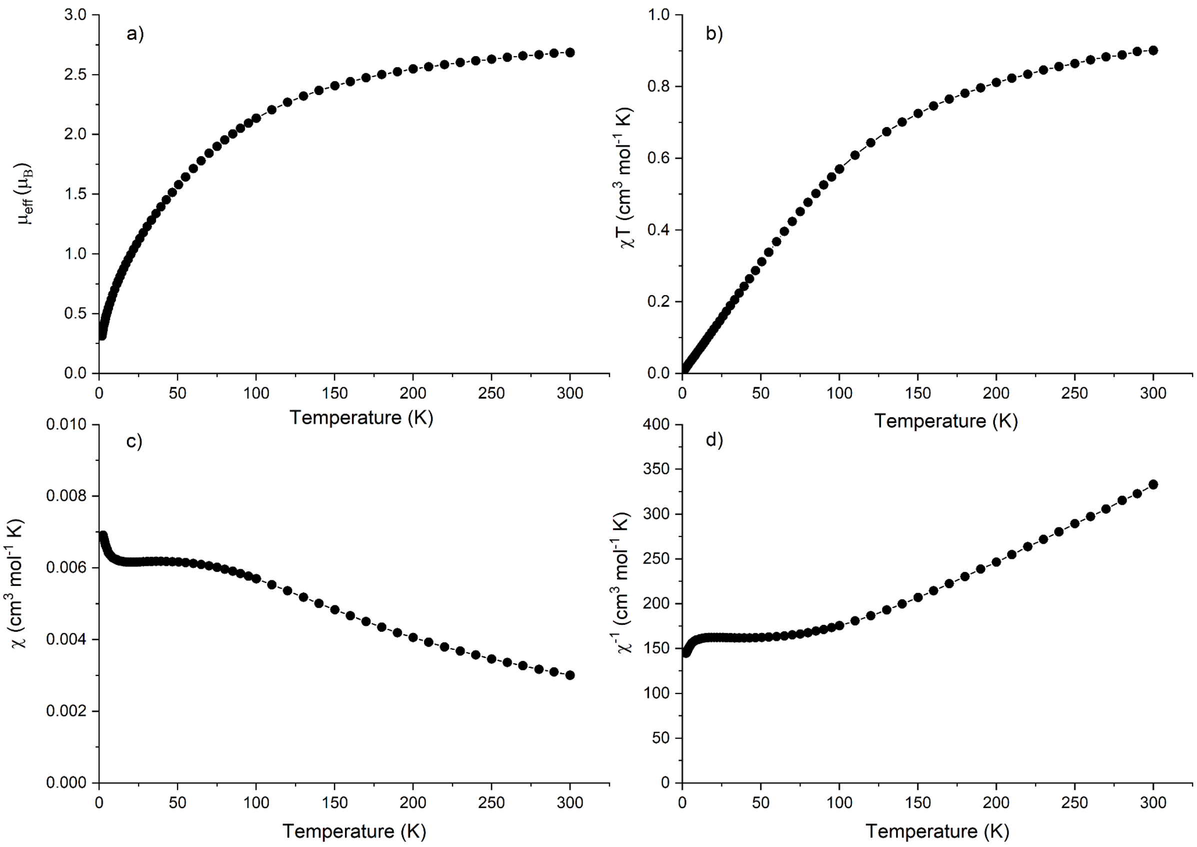

Figure S73. Variable-temperature SQUID magnetometry of **12** over the temperature range 1.8-300 K: a) μ_{eff} vs T; b) χT vs T; c) χ vs T; d) 1/χ vs T. μ_{eff} (300 K) 2.68 μ_B, μ_{eff} (1.8 K) 0.31 μ_B.

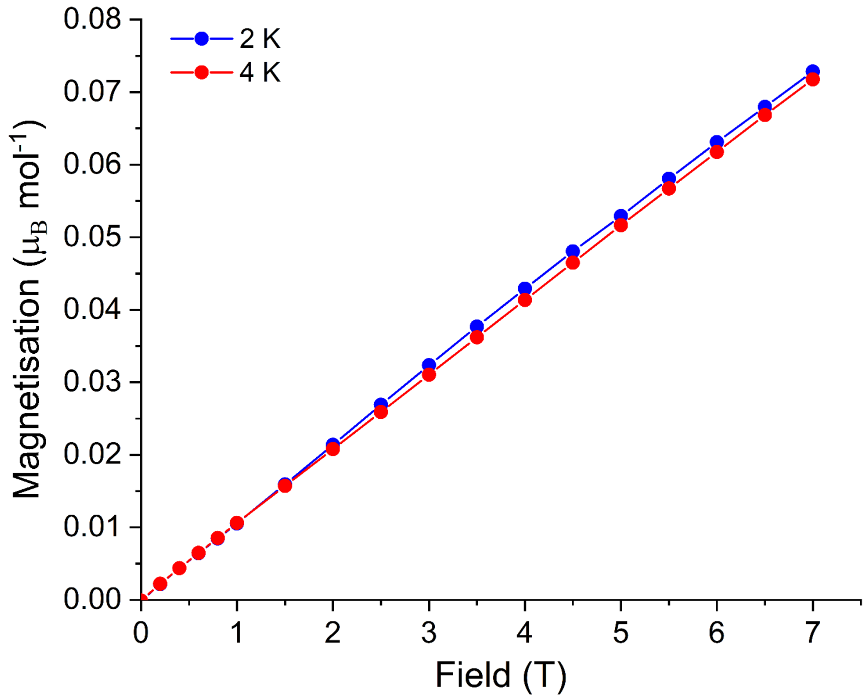

Figure S74. Magnetisation vs Field data for **12** at 2 and 4 K.

Selected Computed Kohn Sham Molecular Orbitals and Natural Bond Orbitals

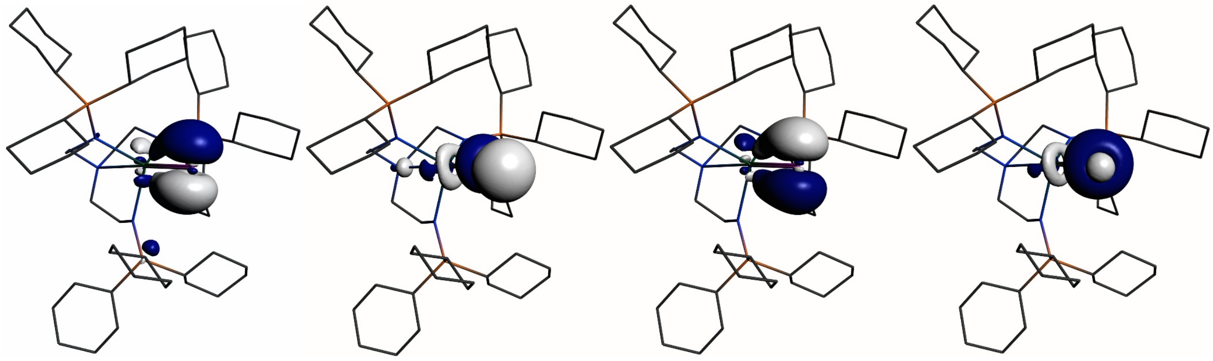

Figure S75. Kohn Sham Molecular Orbitals and Natural Bond Orbitals for **5**. Left to right: HOMO (324, −0.971 eV), HOMO−1 (323, −1.029 eV), Th=P π-NBO, Th=P σ-NBO. H-atoms omitted for clarity.

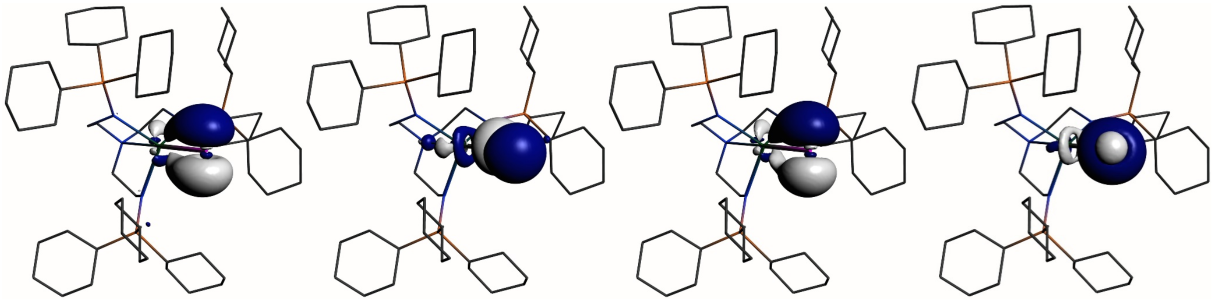

Figure S76. Kohn Sham Molecular Orbitals and Natural Bond Orbitals for **6**. Left to right: HOMO (333, −0.927 eV), HOMO−1 (332, −1.003 eV), Th=As π-NBO, Th=As σ-NBO. H-atoms omitted for clarity.

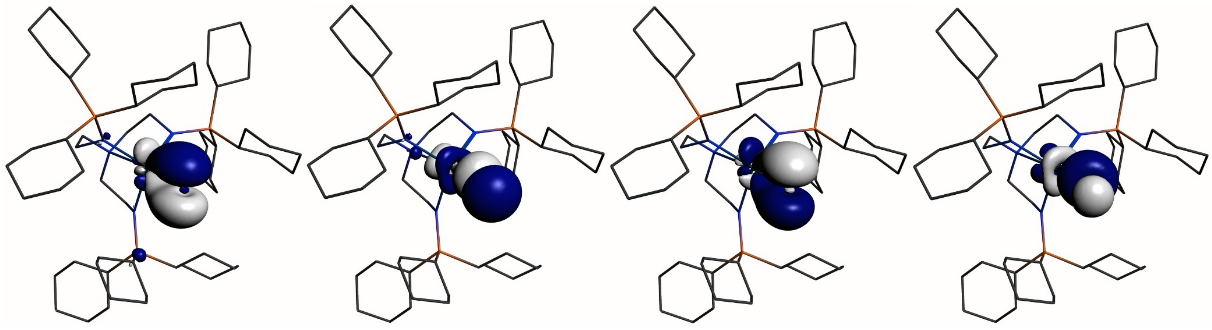

Figure S77. Kohn Sham Molecular Orbitals and Natural Bond Orbitals for **7**. Left to right: HOMO−2 (324a, −1.242 eV), HOMO−3 (323a, −1.357 eV), U=P π-NBO, U=P σ-NBO. H-atoms omitted for clarity.

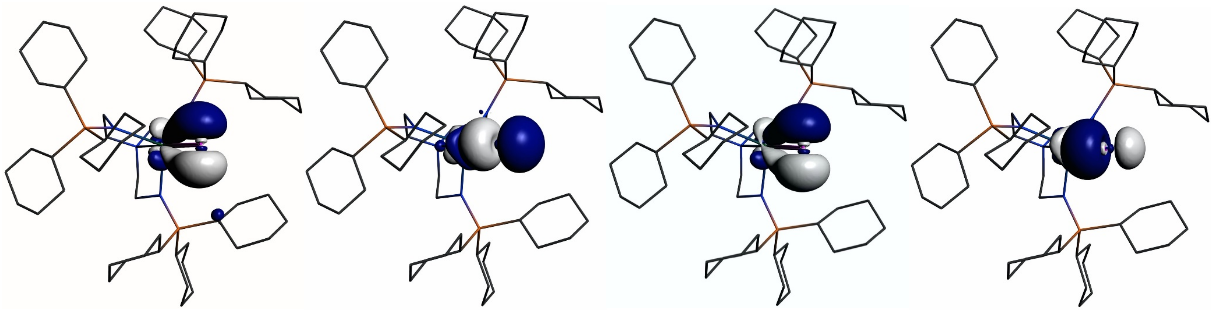

Figure S78. Kohn Sham Molecular Orbitals and Natural Bond Orbitals for **8**. Left to right: HOMO−2 (333a, −1.072 eV), HOMO−3 (332a, −1.234 eV), U=As π-NBO, U=As σ-NBO. H-atoms omitted for clarity.

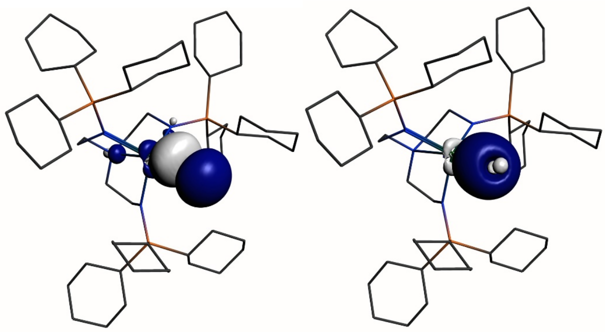

Figure S79. Kohn Sham Molecular Orbitals and Natural Bond Orbitals for **9**. Left to right: HOMO (324, −4.706 eV), Th-P σ-NBO. H-atoms omitted for clarity.

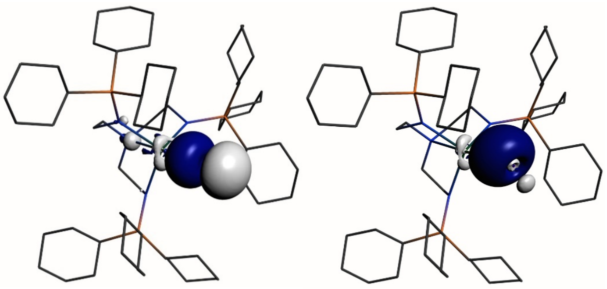

Figure S80. Kohn Sham Molecular Orbitals and Natural Bond Orbitals for **10**. Left to right: HOMO (333, −4.646 eV), Th-As σ-NBO. H-atoms omitted for clarity.

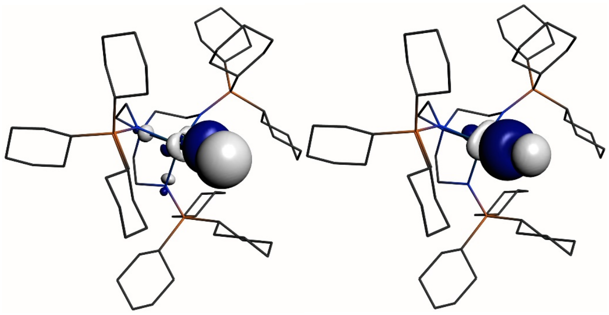

Figure S81. Kohn Sham Molecular Orbitals and Natural Bond Orbitals for **11**. Left to right: HOMO–2 (324a, –4.777 eV), U-P σ-NBO. H-atoms omitted for clarity.

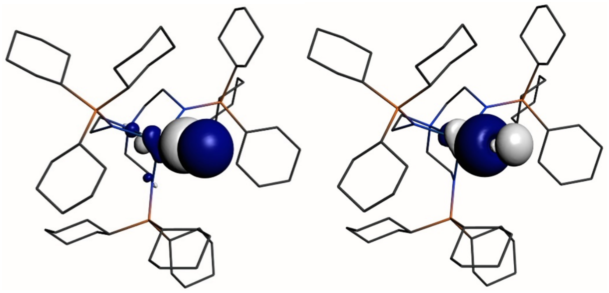

Figure S82. Kohn Sham Molecular Orbitals and Natural Bond Orbitals for **12**. Left to right: HOMO–2 (333a, –4.663 eV), U-As σ-NBO. H-atoms omitted for clarity.

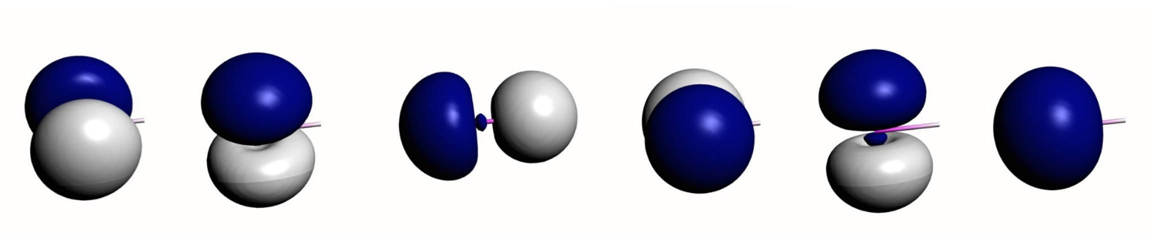

Figure S83. Kohn Sham Molecular Orbitals and Natural Bond Orbitals for neutral triplet AsH. Left to right: HOMO (18, –5.930 eV), HOMO–1 (17, –5.930 eV), HOMO–2 (16, –8.389 eV), As π-NBO, As π-NBO, As σ-NBO.

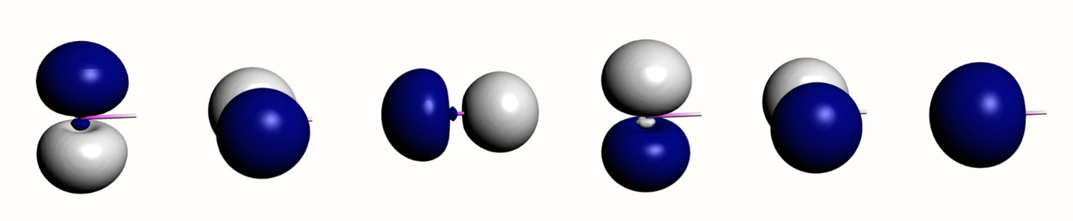

Figure S84. Kohn Sham Molecular Orbitals and Natural Bond Orbitals for singlet $(AsH)^{2-}$ dianion. Left to right: HOMO (18, 7.018 eV), HOMO–1 (17, 7.018 eV), HOMO–2 (16, 3.987 eV), As π-NBO, As π-NBO, As σ-NBO.

Tables

Table S1. Selected bond lengths (Å) and angles (o) for 5-12

Entry	An-P	An-As	<An-P-H	<An-As-H
**5**	2.7237(9)	-	65.83(17)	-
**6**	-	2.8521(8)	-	60.50(12)
**7**	2.6381(12)	-	65.23(13)	-
**8**	-	2.7581(6)	-	61.18(12)
**9**	3.0360(15)	-	78.16(13), 78.18(13)	-
**10**	-	3.0736(4)	-	92.2(4), 92.3(4)
**11**	2.8725(13)	-	84.3(5), 84.0(5)	-
**12**	-	2.9855(8)	-	96.0(5), 96.0(5)

Table S2. Solid Magnetic moments (SQUID) for 2, 4, 7, 8, 11, and 12.

Entry	**μ_{eff} (μ_B, 1.8 K)**	**μ_{eff} (μ_B, 300 K)**
**2**	0.34	2.98
**4**	0.30	2.82
**7**	0.35	3.22
**8**	0.51	2.89
**11**	0.74	2.72
**12**	0.31	2.68

Table S3. Geometry Optimised Final Coordinates and Single Point Energy for 5'

1.C	-2.555150	0.998178	-6.185721
2.C	-1.071930	0.665551	-5.971717
3.C	-2.862767	2.447404	-5.781170
4.C	1.261010	-3.049265	-5.794276
5.C	2.299023	-1.917519	-5.738257
6.C	0.426216	-3.129474	-4.501922
7.C	-0.628322	0.970191	-4.531358
8.C	3.174784	-2.018670	-4.480906
9.C	-2.411945	2.745766	-4.339522
10.C	-0.917754	2.425966	-4.111047
11.C	3.819456	2.780385	-3.857844

12.C	2.299031	3.014374	-3.820571
13.C	1.314206	-3.261221	-3.245498
14.C	-0.331989	5.716204	-3.003798
15.C	2.322212	-2.092336	-3.203710
16.C	-1.039603	7.066590	-2.781781
17.C	-1.117700	-7.404830	-2.679287
18.C	4.539393	3.547340	-2.740490
19.C	-0.117990	-6.296810	-2.299401
20.C	1.685710	2.656024	-2.445092
21.C	-0.823013	4.645218	-2.004132
22.C	-0.830304	-4.990973	-1.878701
23.C	-2.118519	-7.679385	-1.546770
24.C	-1.881773	-1.925750	-1.752481
25.C	-0.893708	7.557458	-1.332722
26.C	3.947266	3.204629	-1.367209
27.C	2.427667	3.428228	-1.329239
28.C	-2.831237	-6.390825	-1.109626
29.C	-3.157521	0.860543	-0.864836
30.C	-1.823134	-5.290924	-0.733588
31.C	-2.985166	-1.592757	-0.744901
32.C	2.873411	-4.738110	-0.710699
33.C	-0.631895	5.150754	-0.557585
34.C	-2.224227	2.053646	-0.643624
35.C	-1.350960	6.490642	-0.325370
36.C	1.696348	-3.847274	-0.247028
37.C	3.974860	-4.830392	0.360099
38.C	1.146993	-4.370545	1.098053
39.C	-3.240299	-0.280297	1.320094
40.C	3.417696	-5.343077	1.694642
41.C	2.248252	-4.468869	2.166943
42.C	2.128001	3.637949	2.426581
43.C	-2.230310	-0.900030	2.291928
44.C	0.834681	2.831452	2.625439
45.C	2.682633	4.165449	3.756099
46.C	1.032747	1.667782	3.622925
47.C	-2.425286	2.670271	4.107258
48.C	1.410750	-1.601244	4.634972
49.C	2.894993	3.018265	4.752822
50.C	-3.709232	3.228053	4.744650
51.C	-1.965294	1.348671	4.762306
52.C	1.600887	2.212070	4.956159
53.C	0.119941	-0.890554	5.093733
54.C	-0.963601	-1.944929	5.419315
55.C	1.900297	-2.647248	5.651929
56.C	0.806953	-3.670345	5.992740
57.C	-3.538577	3.422270	6.258924
58.C	-1.806544	1.556149	6.287711
59.C	-0.482600	-2.977097	6.454854
60.C	-3.082496	2.121898	6.937190
61.H	-2.842574	0.822060	-7.234885
62.H	-0.462121	1.263978	-6.672372
63.H	0.601645	-2.916585	-6.669235

64.H	2.921969	-1.924274	-6.647534
65.H	-2.335858	3.132701	-6.469033
66.H	-0.880186	-0.392127	-6.214162
67.H	1.783782	-4.012219	-5.938797
68.H	-3.940112	2.655142	-5.895442
69.H	-3.168961	0.312859	-5.573788
70.H	1.771645	-0.947688	-5.728851
71.H	4.220951	3.068105	-4.844212
72.H	-0.345773	3.083241	-4.797821
73.H	-0.278320	-3.973584	-4.577121
74.H	3.803912	-2.924895	-4.546372
75.H	1.824117	2.436003	-4.626791
76.H	0.442004	0.737759	-4.416502
77.H	3.865414	-1.161640	-4.427968
78.H	-0.189483	-2.216635	-4.415807
79.H	-2.623253	3.800560	-4.104017
80.H	2.098478	4.078131	-4.045527
81.H	-0.490500	5.385193	-4.042427
82.H	-1.155329	0.288421	-3.840977
83.H	4.016619	1.701243	-3.737042
84.H	-1.672321	-7.092434	-3.582256
85.H	-3.028019	2.144889	-3.646368
86.H	-0.648723	7.825257	-3.480654
87.H	1.902782	-4.194275	-3.366982
88.H	-0.580432	-8.330475	-2.945368
89.H	0.565601	-6.112893	-3.142758
90.H	-2.112896	6.946854	-3.015747
91.H	4.436667	4.633029	-2.925766
92.H	0.756948	5.858329	-2.886754
93.H	1.763969	-1.145790	-3.089202
94.H	5.619330	3.327277	-2.760229
95.H	-1.444252	-4.690254	-2.753028
96.H	-1.871555	-1.133443	-2.529512
97.H	2.968331	-2.147520	-2.315325
98.H	-2.203196	-2.836669	-2.288782
99.H	1.893688	1.579523	-2.263489
100.H	-2.852954	-8.441021	-1.857161
101.H	-1.920627	4.579489	-2.158245
102.H	-3.467157	-6.030222	-1.938324
103.H	-3.117423	0.581743	-1.926901
104.H	0.510300	-6.657118	-1.466225
105.H	-1.460504	8.492355	-1.187490
106.H	3.309128	-4.347754	-1.642051
107.H	2.221194	4.508914	-1.435610
108.H	0.166379	7.800890	-1.140288
109.H	-3.983635	-1.574147	-1.231356
110.H	4.150630	2.149869	-1.116005
111.H	-1.576459	-8.100711	-0.681332
112.H	-2.679346	2.921273	-1.155415
113.H	2.512890	-5.758016	-0.939436
114.H	4.431640	3.807268	-0.581878
115.H	-2.440477	6.336313	-0.429342

116.H	-4.210191	1.120343	-0.623570
117.H	-3.506744	-6.597679	-0.262673
118.H	-2.359715	-4.377535	-0.439301
119.H	2.048143	3.115896	-0.346366
120.H	0.443207	5.281294	-0.351033
121.H	-3.001291	-2.379915	0.022471
122.H	4.792884	-5.482555	0.009884
123.H	-1.265095	-5.612765	0.161080
124.H	2.124430	-2.838111	-0.050144
125.H	-0.988560	4.400235	0.162892
126.H	-2.249609	2.315586	0.434160
127.H	-1.181376	6.844071	0.704763
128.H	4.403834	-3.825483	0.510115
129.H	0.704004	-5.373620	0.957433
130.H	-4.223940	-0.793406	1.370992
131.H	3.069238	-6.385246	1.569651
132.H	2.082591	-0.696544	1.515887
133.H	0.331841	-3.721499	1.458896
134.H	1.947437	4.473432	1.731600
135.H	-3.394576	0.768529	1.609264
136.H	0.504815	2.452113	1.641165
137.H	2.871074	2.975588	1.950220
138.H	-2.061850	-1.956333	1.998662
139.H	4.210755	-5.366131	2.460112
140.H	2.624203	-3.454733	2.379780
141.H	0.032945	3.505337	2.972373
142.H	1.831894	-4.863772	3.108553
143.H	-2.566373	2.534333	3.023487
144.H	-2.722602	-0.958934	3.280377
145.H	1.813075	1.022499	3.169295
146.H	3.627161	4.708152	3.587038
147.H	1.223044	-2.096736	3.667259
148.H	1.973671	4.895656	4.189290
149.H	-1.628527	3.425489	4.218282
150.H	-3.989473	4.181873	4.267793
151.H	3.679549	2.346004	4.366166
152.H	2.212367	-0.871958	4.444730
153.H	-1.236238	-2.479575	4.492807
154.H	-4.542517	2.526128	4.560700
155.H	-2.803612	0.631532	4.638243
156.H	0.585878	-4.272554	5.093818
157.H	2.793240	-3.162008	5.262865
158.H	0.853375	2.868865	5.437349
159.H	3.254210	3.409529	5.720052
160.H	1.787948	1.388385	5.662708
161.H	-1.886296	-1.462286	5.781283
162.H	0.354922	-0.367779	6.043378
163.H	-2.780179	4.205836	6.436775
164.H	2.212344	-2.130960	6.577832
165.H	-1.271263	-3.724755	6.644066
166.H	-0.972171	2.252437	6.481418
167.H	1.163592	-4.375391	6.761792

168.H	-4.475835	3.785479	6.711501
169.H	-1.535134	0.611017	6.781074
170.H	-3.889494	1.371251	6.856514
171.H	-0.296390	-2.464496	7.415981
172.H	-2.917794	2.291152	8.014462
173.N	-0.562876	-2.071508	-1.099887
174.N	-0.838574	1.774296	-1.089586
175.N	-2.713677	-0.305143	-0.066050
176.N	-0.963001	-0.138685	2.314481
177.P	2.769036	-0.083381	0.374200
178.Si	-0.227239	2.831943	-2.343963
179.Si	0.366132	-3.484799	-1.577719
180.Si	-0.461178	0.489477	3.869670
181.Th	0.070662	-0.094598	0.141112

Energy: −1005.66150906 eV

Table S4. Geometry Optimised Final Coordinates and Single Point Energy for 6'

1.C	-2.546190	1.361252	-7.069135
2.C	-2.785523	2.782888	-6.537398
3.C	-1.502929	0.605501	-6.224835
4.C	3.603252	1.085239	-5.233247
5.C	3.353797	-0.427082	-5.150185
6.C	-3.167841	2.762274	-5.049607
7.C	1.853108	-0.754447	-5.063720
8.C	-2.518132	-4.191477	-4.947761
9.C	-1.868990	0.585541	-4.722514
10.C	-2.405414	-2.764809	-4.380958
11.C	-0.938345	-2.298458	-4.234158
12.C	-1.720157	-5.198483	-4.107105
13.C	-2.112440	2.022292	-4.211518
14.C	-0.256923	-4.758334	-3.956763
15.C	2.925593	1.820319	-4.069612
16.C	1.427671	1.493416	-3.990475
17.C	1.167776	-0.028772	-3.879606
18.C	-0.148757	-3.328464	-3.398766
19.C	3.613211	-5.074151	-2.105346
20.C	4.211103	-3.713495	-1.719455
21.C	0.105884	4.597287	-1.781745
22.C	0.162239	6.094000	-1.448789
23.C	-2.553266	-0.374871	-1.651716
24.C	3.282754	-5.914649	-0.862701
25.C	-0.861953	3.847346	-0.851926
26.C	3.307658	-2.959663	-0.730273
27.C	-2.883437	-1.530861	-0.701856
28.C	0.525837	6.312647	0.025891
29.C	2.382592	-5.147347	0.124049
30.C	-5.648158	3.529967	0.672656
31.C	-4.123548	3.361727	0.556118
32.C	2.996573	-3.789983	0.534406
33.C	-0.497815	4.044778	0.636838
34.C	-0.436501	5.557745	0.958772
35.C	-0.539576	-3.071973	0.849778

36.C	-6.011010	4.874247	1.322357
37.C	-1.909660	-2.571190	1.309297
38.C	-3.102170	-0.433174	1.491542
39.C	-3.412895	3.499616	1.920730
40.C	-5.301603	5.051698	2.673562
41.C	-3.778003	4.864128	2.549135
42.C	3.115860	-1.330135	2.412380
43.C	4.620938	-1.670868	2.517683
44.C	-2.211687	0.433861	2.383164
45.C	5.470129	-0.427216	2.835423
46.C	1.526266	-3.993142	3.245833
47.C	-0.860344	3.533677	3.653524
48.C	2.646954	-0.621691	3.703416
49.C	0.661318	3.308631	3.794282
50.C	2.746091	-4.592363	3.982297
51.C	3.494082	0.623747	4.012533
52.C	4.986122	0.277827	4.109973
53.C	0.507786	-3.455285	4.274962
54.C	2.333328	-5.671685	4.999961
55.C	-1.594785	2.865475	4.838804
56.C	1.199455	3.786185	5.153524
57.C	0.081559	-4.533654	5.285402
58.C	1.296795	-5.141682	6.002985
59.C	-1.070612	3.357110	6.199457
60.C	0.444754	3.140504	6.324915
61.H	-2.228065	1.398430	-8.124878
62.H	-3.499879	0.803226	-7.047411
63.H	-3.563916	3.288880	-7.131829
64.H	-1.861402	3.375621	-6.661380
65.H	-1.389271	-0.420243	-6.611164
66.H	-0.521943	1.093542	-6.357114
67.H	3.203018	1.464176	-6.192748
68.H	3.801626	-0.940071	-6.018616
69.H	-2.131988	-4.198951	-5.983022
70.H	1.368620	-0.454673	-6.011306
71.H	4.685592	1.293275	-5.241283
72.H	-0.502524	-2.306818	-5.254573
73.H	-4.143696	2.255746	-4.934547
74.H	1.724562	-1.844643	-4.986362
75.H	0.235937	-4.797690	-4.944707
76.H	-3.577112	-4.496069	-5.004658
77.H	-2.969918	-2.072237	-5.024430
78.H	-3.301865	3.790251	-4.674950
79.H	0.922387	1.889896	-4.889351
80.H	-1.776604	-6.204419	-4.554145
81.H	-2.847913	0.067311	-4.655159
82.H	3.855633	-0.823494	-4.251013
83.H	3.066871	2.908690	-4.170963
84.H	-1.169267	2.591937	-4.243476
85.H	0.286884	-5.463454	-3.307140
86.H	0.911165	-3.038967	-3.324958
87.H	-2.902279	-2.744404	-3.394762

88.H	-2.178394	-5.276150	-3.104464
89.H	-2.415275	2.001399	-3.154163
90.H	3.390594	1.533042	-3.110181
91.H	4.299152	-5.624798	-2.769823
92.H	0.995636	2.011849	-3.122417
93.H	1.683351	-0.354450	-2.950681
94.H	-0.190181	4.451205	-2.832841
95.H	2.686333	-4.907645	-2.682275
96.H	4.378239	-3.101165	-2.620331
97.H	-3.154188	-0.524642	-2.568061
98.H	-0.531511	-3.317680	-2.362461
99.H	0.887505	6.604093	-2.103063
100.H	-0.822885	6.553560	-1.652138
101.H	1.111225	4.158540	-1.665927
102.H	5.202677	-3.869624	-1.257573
103.H	2.802272	-6.861883	-1.160574
104.H	1.425804	1.836994	-1.017210
105.H	-2.947458	0.561695	-1.208105
106.H	-1.889101	4.202407	-1.042885
107.H	2.354335	-2.713307	-1.231230
108.H	-2.460786	-2.455417	-1.119841
109.H	-0.862293	2.775909	-1.120137
110.H	4.222879	-6.186538	-0.349781
111.H	-6.120297	3.447858	-0.320567
112.H	-3.980057	-1.674689	-0.603450
113.H	3.754435	-1.987011	-0.473459
114.H	1.400779	-4.976734	-0.352046
115.H	-3.740237	4.134567	-0.131973
116.H	-0.598712	-3.323818	-0.228992
117.H	0.520762	7.388939	0.267528
118.H	-3.880997	2.394324	0.089235
119.H	1.551932	5.948331	0.201274
120.H	-5.706415	5.693074	0.646118
121.H	-1.444642	6.000481	0.855103
122.H	2.193425	-5.776208	1.009112
123.H	0.529176	3.637169	0.754608
124.H	3.963993	-4.015138	1.028104
125.H	-6.058124	2.707306	1.285770
126.H	-7.102899	4.961434	1.446637
127.H	-3.691015	0.231254	0.844424
128.H	-2.695784	-3.338255	1.149322
129.H	-0.368175	-4.040588	1.355881
130.H	4.980278	-2.121677	1.580834
131.H	-0.134478	5.720786	2.004611
132.H	3.016004	-0.583515	1.592641
133.H	-3.369427	5.678483	1.925563
134.H	5.400481	0.274101	1.986808
135.H	-3.814455	-1.042693	2.086659
136.H	-1.857755	-2.365788	2.387671
137.H	-3.865545	2.731585	2.582709
138.H	-5.532022	6.043433	3.096932
139.H	1.031875	-4.845525	2.734765

140.H	6.532054	-0.709215	2.932825
141.H	-5.695792	4.305955	3.387731
142.H	1.210258	3.808711	2.982597
143.H	-1.606877	-0.232146	3.032937
144.H	-2.874423	0.977700	3.080694
145.H	4.782705	-2.426328	3.308816
146.H	3.464093	-5.018071	3.263606
147.H	-3.312862	4.975069	3.540408
148.H	3.340689	1.359115	3.204330
149.H	-1.040712	4.624814	3.741500
150.H	1.585467	-0.336210	3.620274
151.H	0.878580	2.233915	3.672033
152.H	-0.375914	-3.050047	3.760848
153.H	5.579170	1.188780	4.292297
154.H	1.902044	-6.530590	4.455038
155.H	3.283649	-3.788563	4.515423
156.H	2.715729	-1.319412	4.558234
157.H	-0.462300	-5.333961	4.751641
158.H	-2.681526	3.040246	4.776885
159.H	0.950365	-2.608076	4.824679
160.H	5.152716	-0.386084	4.979253
161.H	-1.451465	1.772670	4.784312
162.H	3.149399	1.093614	4.948511
163.H	1.094476	4.884448	5.217967
164.H	2.277046	3.568769	5.229305
165.H	3.219102	-6.051896	5.535381
166.H	-0.622740	-4.112078	6.021630
167.H	-1.293975	4.434254	6.303641
168.H	0.978384	-5.945877	6.686445
169.H	0.651668	2.055335	6.331446
170.H	1.767811	-4.364605	6.630881
171.H	-1.600141	2.844604	7.020608
172.H	0.811877	3.535312	7.286575
173.As	2.620042	1.357854	-0.105173
174.N	-1.098660	-0.265740	-1.922089
175.N	-2.269191	-1.306392	0.628126
176.N	0.523302	-2.079690	1.122833
177.N	-1.350038	1.327019	1.576434
178.Si	-0.681503	-0.473961	-3.612077
179.Si	1.986658	-2.760044	1.819367
180.Si	-1.522416	3.038983	1.907889
181.Th	0.108821	0.072678	0.143330

Energy: −1005.00523203 eV

Table S5. Geometry Optimised Final Coordinates and Single Point Energy for 7'

1.C	0.217262	2.640787	-7.212175
2.C	0.498173	3.970443	-6.493393
3.C	0.716517	1.438474	-6.395753
4.C	2.759760	-5.559499	-4.923474
5.C	-0.050215	3.973106	-5.053347
6.C	0.155861	1.457128	-4.963622
7.C	3.051161	-4.129745	-4.443004

8.C	-3.402256	-2.713802	-4.449911
9.C	0.491924	2.778407	-4.237810
10.C	2.366280	-6.469185	-3.750946
11.C	-2.700403	-1.540319	-3.753727
12.C	-3.546850	-3.906688	-3.495659
13.C	1.866536	-3.557377	-3.646126
14.C	1.188464	-5.886644	-2.948865
15.C	-1.338891	-1.949426	-3.168737
16.C	-2.184956	-4.321937	-2.913170
17.C	-2.652924	2.030621	-2.753605
18.C	-4.657744	3.563435	-2.444332
19.C	1.473669	-4.451254	-2.449902
20.C	-4.135224	2.123239	-2.359018
21.C	2.449127	4.612589	-2.091879
22.C	-1.469214	-3.147772	-2.202801
23.C	-2.301442	4.423895	-2.014902
24.C	2.074172	0.903348	-2.123919
25.C	-1.754338	2.977709	-1.925102
26.C	-3.784514	4.514021	-1.613550
27.C	3.028845	5.941793	-1.573512
28.C	0.970707	4.417020	-1.689477
29.C	3.119188	0.850698	-1.009361
30.C	3.146397	-1.505759	-0.284948
31.C	2.865404	6.077231	-0.052331
32.C	0.821365	4.575440	-0.161715
33.C	-0.382204	-5.118770	-0.092906
34.C	1.398797	5.899752	0.363727
35.C	2.105190	-2.508142	0.217534
36.C	0.782150	-5.714376	0.732002
37.C	-1.545789	-4.748870	0.851160
38.C	0.339531	-6.914728	1.587168
39.C	3.168261	0.298480	1.386851
40.C	-1.993077	-5.931727	1.728034
41.C	2.178863	1.287458	2.005191
42.C	-0.822531	-6.537369	2.516385
43.C	-1.789254	5.319558	3.119818
44.C	-1.938837	2.794106	2.881763
45.C	-2.719334	4.097922	3.113918
46.C	0.127916	3.825983	3.894215
47.C	-0.637967	5.143225	4.122204
48.C	-0.807694	2.597547	3.914083
49.C	-1.279080	-0.444873	3.979932
50.C	-1.897277	-2.911768	4.230438
51.C	-0.754666	-1.883395	4.182002
52.C	-3.464994	-1.138843	5.110995
53.C	-2.322350	-0.109036	5.072308
54.C	2.376969	-0.357482	5.106323
55.C	-2.929819	-2.563055	5.310629
56.C	1.426719	0.860647	5.125050
57.C	3.513323	-0.226276	6.135210
58.C	0.876401	1.062419	6.555383
59.C	2.962870	-0.003130	7.551542

60.C	2.005094	1.197330	7.594470
61.H	0.678894	2.644085	-8.213599
62.H	-0.871430	2.539664	-7.369572
63.H	0.073854	4.809643	-7.069563
64.H	0.445979	0.497593	-6.902525
65.H	1.590223	4.135983	-6.455840
66.H	1.820571	1.463724	-6.349454
67.H	1.928091	-5.530694	-5.650704
68.H	3.629622	-5.973486	-5.459180
69.H	3.285089	-3.479374	-5.302061
70.H	-2.812943	-3.024960	-5.332597
71.H	-1.153005	3.924526	-5.089353
72.H	-0.938806	1.329485	-5.006306
73.H	-4.389605	-2.400452	-4.827122
74.H	0.201214	4.928919	-4.566365
75.H	2.116546	-7.478847	-4.117711
76.H	0.536415	0.597263	-4.394560
77.H	-2.572649	-0.701817	-4.456940
78.H	1.597900	2.871802	-4.275220
79.H	-4.015386	-4.760976	-4.013473
80.H	1.002922	-3.463338	-4.326981
81.H	3.950032	-4.140315	-3.800656
82.H	-0.647110	-2.191972	-3.993720
83.H	-2.554475	2.279944	-3.825936
84.H	-1.552773	-4.696071	-3.739324
85.H	-4.648800	3.895439	-3.499395
86.H	0.290654	-5.882793	-3.590898
87.H	3.235117	-6.585272	-3.077878
88.H	2.094576	-2.535768	-3.303457
89.H	2.562167	4.568102	-3.186515
90.H	-4.733832	1.460682	-3.005653
91.H	-2.192817	4.794365	-3.050516
92.H	-3.330807	-1.172894	-2.925849
93.H	-4.219596	-3.625204	-2.667333
94.H	2.478691	1.562552	-2.914724
95.H	-2.314113	0.993563	-2.625386
96.H	-0.901600	-1.080094	-2.646164
97.H	-2.323681	-5.168594	-2.222657
98.H	2.011538	-0.100207	-2.591547
99.H	-5.706474	3.615901	-2.108854
100.H	0.956810	-6.552879	-2.104396
101.H	2.502622	6.778253	-2.067830
102.H	0.402071	5.245070	-2.160682
103.H	4.092320	6.030070	-1.852440
104.H	-4.140047	5.553244	-1.716348
105.H	2.375907	-4.524780	-1.806745
106.H	3.055669	3.784481	-1.684395
107.H	-1.715278	5.107755	-1.382756
108.H	-4.251308	1.739319	-1.331570
109.H	-2.134981	-2.795302	-1.387957
110.H	4.125650	0.596928	-1.402659
111.H	3.215276	-1.599395	-1.377372

112.H	-1.851445	2.651665	-0.867798
113.H	-3.882120	4.250108	-0.546208
114.H	-0.747449	-5.927315	-0.759345
115.H	3.181252	1.843735	-0.542916
116.H	0.804069	6.739403	-0.038144
117.H	1.615080	-6.016631	0.076209
118.H	3.249635	7.051674	0.291330
119.H	-0.234963	4.487487	0.135588
120.H	2.517127	-3.521766	0.059315
121.H	4.152678	-1.702838	0.138624
122.H	3.477721	5.305618	0.447630
123.H	1.339067	3.735845	0.334885
124.H	-2.406337	-4.369889	0.280233
125.H	0.020408	-7.735852	0.919889
126.H	-1.894122	-1.491404	0.745171
127.H	-2.434959	-6.712654	1.082970
128.H	1.305806	5.946511	1.460843
129.H	4.194085	0.720583	1.334835
130.H	1.182749	-4.936702	1.404847
131.H	2.021579	-2.406570	1.319978
132.H	2.150906	2.196770	1.371601
133.H	-1.228207	-3.914750	1.499238
134.H	1.189486	-7.298963	2.175779
135.H	-1.368012	5.454051	2.108556
136.H	-1.497286	2.810674	1.869225
137.H	3.204081	-0.598228	2.020912
138.H	-3.498669	4.215859	2.343706
139.H	-2.788278	-5.608656	2.419223
140.H	-1.157612	-7.415355	3.092563
141.H	0.639303	3.873867	2.916490
142.H	2.617858	1.624930	2.961848
143.H	-2.627404	1.936555	2.872594
144.H	-2.355856	6.238023	3.346009
145.H	-0.463866	-5.797273	3.253498
146.H	-1.820071	-0.434150	3.008798
147.H	-2.399039	-2.928427	3.248849
148.H	-0.049736	-2.146646	3.376475
149.H	0.051419	6.002639	4.063403
150.H	-3.244605	4.036959	4.084711
151.H	-4.016013	-1.090874	4.156509
152.H	2.797335	-0.503887	4.101260
153.H	-1.492420	-3.922728	4.401527
154.H	0.919660	3.730076	4.654917
155.H	-1.050601	5.141749	5.147637
156.H	-2.745956	0.891948	4.906218
157.H	-1.281013	2.571009	4.917432
158.H	2.057632	1.750792	4.920409
159.H	-0.186886	-1.946684	5.128115
160.H	-3.757529	-3.291438	5.298322
161.H	1.804135	-1.273238	5.328093
162.H	-4.181566	-0.882182	5.909662
163.H	4.156303	0.628396	5.857260

164.H	-1.837591	-0.080411	6.065520
165.H	4.155667	-1.122481	6.113392
166.H	-2.457410	-2.639457	6.307700
167.H	0.226360	1.949782	6.600416
168.H	0.242837	0.201403	6.831103
169.H	2.575043	2.120640	7.385581
170.H	2.418294	-0.909344	7.871442
171.H	3.787691	0.135929	8.269475
172.H	1.582724	1.311388	8.606684
173.N	0.753058	1.332924	-1.612798
174.N	0.801237	-2.292788	-0.444617
175.N	2.715402	-0.121137	0.037456
176.N	0.840994	0.680175	2.154350
177.P	-2.597133	-0.279482	0.304894
178.Si	0.118772	2.797081	-2.335493
179.Si	0.127139	-3.674510	-1.278644
180.Si	0.075921	0.888870	3.722278
181.U	0.004659	-0.136516	0.088507

Energy: −1005.82895615 eV

Table S6. Geometry Optimised Final Coordinates and Single Point Energy for 8'

1.C	-3.386999	-0.154833	-6.669880
2.C	-2.054122	-0.646125	-6.086307
3.C	-4.575653	-0.674563	-5.847723
4.C	4.590440	-3.109981	-5.446849
5.C	1.721580	3.174252	-5.122978
6.C	5.714554	-2.626199	-4.517751
7.C	3.255411	-3.231853	-4.696763
8.C	3.129871	3.362546	-4.542020
9.C	-1.918915	-0.270451	-4.601517
10.C	-4.440993	-0.302618	-4.359788
11.C	3.663242	2.057788	-3.926033
12.C	5.340074	-1.320782	-3.789293
13.C	2.899258	-1.919708	-3.979062
14.C	0.765558	2.581825	-4.079221
15.C	-3.102731	-0.784101	-3.755590
16.C	1.305822	1.271857	-3.484185
17.C	4.011550	-1.457354	-3.012029
18.C	2.699820	1.481015	-2.860149
19.C	-2.523942	2.389381	-2.221406
20.C	-2.691668	3.823058	-1.693762
21.C	-0.870713	-2.264918	-1.804706
22.C	-4.168573	4.200733	-1.523415
23.C	-4.460336	-3.005880	-1.510672
24.C	-3.243074	1.347530	-1.333147
25.C	-4.732162	1.742128	-1.182028
26.C	-5.678654	-3.718864	-0.893711
27.C	5.092587	0.750206	-1.110038
28.C	-4.359748	-1.529114	-1.066527
29.C	-4.900714	3.175202	-0.647790
30.C	-0.563317	-3.222277	-0.651867
31.C	4.881397	2.108324	-0.402291

32.C	5.855630	-0.208949	-0.168811
33.C	6.199984	2.716267	0.105470
34.C	7.182559	0.392335	0.329346
35.C	1.861010	-2.980478	-0.245421
36.C	2.787021	-1.797212	0.040042
37.C	-5.682471	-3.616895	0.638887
38.C	-4.362237	-1.455108	0.473421
39.C	6.960797	1.745894	1.019751
40.C	-5.578045	-2.156495	1.100728
41.C	0.297435	-3.047660	1.647537
42.C	-0.719304	-2.131393	2.327633
43.C	-2.495975	2.013473	3.164547
44.C	-4.984780	1.600934	3.476018
45.C	-3.864059	2.649702	3.459916
46.C	3.232570	2.423251	3.640675
47.C	2.336706	1.180914	3.782525
48.C	0.849267	1.567692	3.930909
49.C	-3.285693	-0.165360	4.168757
50.C	-2.150980	0.883160	4.155702
51.C	-4.664273	0.459464	4.452161
52.C	3.041693	3.398499	4.811442
53.C	1.564149	3.783377	4.973941
54.C	0.673717	2.537022	5.121956
55.C	1.132927	-1.924047	5.371187
56.C	-0.195241	-1.136951	5.349692
57.C	1.142169	-3.019273	6.452560
58.C	-0.472564	-0.551159	6.752983
59.C	0.840210	-2.439403	7.842564
60.C	-0.471457	-1.640252	7.841252
61.H	-3.485542	-0.460356	-7.724424
62.H	-3.397977	0.949791	-6.661125
63.H	-1.209974	-0.231968	-6.661476
64.H	4.470457	-2.388465	-6.274677
65.H	-5.522926	-0.283164	-6.255012
66.H	-1.997092	-1.744932	-6.190654
67.H	4.862373	-4.073257	-5.909173
68.H	-4.622996	-1.774681	-5.940013
69.H	1.777570	2.496295	-5.994752
70.H	3.820901	3.726302	-5.321779
71.H	6.648114	-2.492944	-5.089625
72.H	2.450982	-3.518754	-5.394114
73.H	1.330705	4.134604	-5.498494
74.H	3.800582	1.316560	-4.735182
75.H	5.253465	-0.508402	-4.532202
76.H	2.733032	-1.137454	-4.740033
77.H	-1.858139	0.827001	-4.519903
78.H	-4.512503	0.795161	-4.263586
79.H	-0.226964	2.416686	-4.527341
80.H	3.329996	-4.043803	-3.950885
81.H	5.917684	-3.407659	-3.763217
82.H	-0.970827	-0.653593	-4.198033
83.H	3.096075	4.138363	-3.757550

84.H	1.344957	0.499383	-4.271367
85.H	-5.293066	-0.716986	-3.797679
86.H	-3.098980	-1.889387	-3.856089
87.H	4.662974	2.236577	-3.500039
88.H	6.159326	-1.032276	-3.113438
89.H	1.943372	-2.027926	-3.442945
90.H	0.627380	3.298065	-3.250800
91.H	-2.930270	2.335496	-3.248044
92.H	-4.511173	-3.080698	-2.608215
93.H	-4.654991	4.241603	-2.515680
94.H	4.165504	-2.290849	-2.295169
95.H	0.593769	0.906088	-2.723697
96.H	-2.188700	4.531936	-2.370340
97.H	-1.604858	-2.770422	-2.460806
98.H	-1.450536	2.158487	-2.280747
99.H	-5.232754	1.668221	-2.164548
100.H	0.041614	-2.155771	-2.424176
101.H	2.551829	2.255902	-2.080292
102.H	5.756980	0.936590	-1.979606
103.H	-6.600970	-3.255824	-1.288459
104.H	-5.700264	-4.777025	-1.204470
105.H	-5.287809	-1.032189	-1.417071
106.H	-4.260088	5.209083	-1.087618
107.H	-3.547183	-3.547144	-1.205532
108.H	6.837283	2.970064	-0.761408
109.H	4.388190	2.824952	-1.074464
110.H	1.824644	-3.138146	-1.331778
111.H	7.860828	0.531188	-0.531791
112.H	-0.261903	-4.225141	-1.019203
113.H	6.053762	-1.173082	-0.666524
114.H	-5.971833	3.426602	-0.570981
115.H	-2.172193	3.902817	-0.723550
116.H	-5.260894	1.044026	-0.516439
117.H	-2.780426	1.432876	-0.325766
118.H	3.820360	-2.104781	-0.202350
119.H	-1.474994	-3.344827	-0.050913
120.H	5.999313	3.661680	0.635191
121.H	-4.491308	3.221348	0.376146
122.H	2.219892	-3.919261	0.224278
123.H	4.188341	1.972874	0.445067
124.H	7.685074	-0.309069	1.016371
125.H	5.222633	-0.433531	0.706558
126.H	-6.496243	-1.616377	0.807953
127.H	-6.588523	-4.089236	1.052990
128.H	1.620426	1.976554	0.676907
129.H	7.924423	2.182428	1.330802
130.H	-4.318910	-0.409560	0.814464
131.H	-4.823082	-4.183398	1.040263
132.H	-3.439226	-1.927824	0.852831
133.H	2.801160	-1.616052	1.135487
134.H	6.374456	1.588540	1.941854
135.H	0.000950	-4.115300	1.716904

136.H	-5.521734	-2.107341	2.200206
137.H	-1.692154	-2.239596	1.807162
138.H	1.262414	-2.931833	2.161503
139.H	-2.498074	1.606937	2.136845
140.H	-5.101587	1.185211	2.459907
141.H	-4.085652	3.434031	2.718165
142.H	2.971530	2.933072	2.698008
143.H	2.469170	0.527411	2.904986
144.H	-1.714573	2.787553	3.159113
145.H	0.589543	2.136301	3.011349
146.H	-3.317056	-0.668985	3.186726
147.H	-5.950160	2.067153	3.733480
148.H	-0.893238	-2.544197	3.336882
149.H	4.291277	2.124289	3.563297
150.H	-5.450687	-0.312929	4.406187
151.H	-3.824385	3.152303	4.443780
152.H	1.238286	4.347318	4.083898
153.H	3.658665	4.300160	4.663097
154.H	1.335098	-2.368079	4.384865
155.H	2.667139	0.597895	4.661515
156.H	-3.083745	-0.952159	4.913600
157.H	-2.118050	1.339637	5.166610
158.H	-4.672804	0.855647	5.483995
159.H	-0.997822	-1.884793	5.182036
160.H	-0.377983	2.846995	5.221448
161.H	3.397219	2.924385	5.745265
162.H	1.966487	-1.227333	5.564501
163.H	1.435282	4.450048	5.843550
164.H	0.936719	2.026988	6.066788
165.H	0.377891	-3.777713	6.203691
166.H	2.112679	-3.542914	6.461559
167.H	-1.435718	-0.019191	6.768087
168.H	0.297808	0.199168	7.000860
169.H	-1.315014	-2.330089	7.657677
170.H	1.667881	-1.771233	8.140869
171.H	0.800340	-3.243695	8.595993
172.H	-0.644527	-1.190431	8.833096
173.As	0.350008	2.784928	0.202420
174.N	-1.332574	-0.951975	-1.303641
175.N	2.338100	-0.595768	-0.694591
176.N	0.490604	-2.657563	0.228808
177.N	-0.254968	-0.729056	2.339911
178.Si	3.467580	0.028215	-1.877749
179.Si	-2.926491	-0.480786	-1.849851
180.Si	-0.398584	0.116876	3.874135
181.U	0.249166	0.043531	0.145770

Energy: −1005.10448956 eV

Table S7. Geometry Optimised Final Coordinates and Single Point Energy for 9

1.C	5.675191	3.278279	-3.411534
2.C	6.495300	1.981519	-3.204663
3.C	-5.449460	3.451246	-3.150454

4.C	-0.964829	-6.553500	-2.764244
5.C	5.559833	0.775351	-3.068420
6.C	0.822170	1.261078	-3.176285
7.C	-1.509206	0.481905	-3.158825
8.C	0.328793	-1.157119	-3.129287
9.C	-2.459051	-6.241678	-2.594647
10.C	4.278433	3.194882	-2.747309
11.C	-6.733160	3.937397	-2.461243
12.C	-2.702106	-4.739727	-2.382337
13.C	-4.404930	2.971387	-2.126837
14.C	0.873333	2.377081	-2.135121
15.C	-2.503240	-0.076359	-2.145227
16.C	1.339024	-1.699393	-2.120683
17.C	4.690740	0.900167	-1.790992
18.C	-7.306432	2.863480	-1.525625
19.C	-0.126556	-6.000214	-1.596829
20.C	4.325809	2.371395	-1.436594
21.C	-4.971786	1.870016	-1.203339
22.C	-0.349940	-4.481599	-1.414559
23.C	-1.854412	-4.189769	-1.223114
24.C	-6.258322	2.380237	-0.507784
25.C	5.087730	-3.739040	-0.384907
26.C	-5.488362	-1.320194	-0.264383
27.C	2.572979	-4.204003	-0.371550
28.C	4.248327	-6.090459	0.045974
29.C	3.352973	6.904351	-0.030187
30.C	2.510814	4.495085	-0.109954
31.C	3.692131	-3.186628	-0.051213
32.C	3.742301	5.418023	0.050804
33.C	5.357167	-5.068493	0.334235
34.C	-6.229381	-2.495188	0.398342
35.C	2.855983	-5.527127	0.381501
36.C	1.045217	6.346262	0.863865
37.C	-4.679586	-0.489976	0.759053
38.C	-5.267449	-3.401106	1.179325
39.C	2.271927	7.259461	1.001030
40.C	1.433151	4.860018	0.935729
41.C	-2.846484	3.615460	1.038025
42.C	-3.728606	-1.411659	1.553611
43.C	3.592800	0.348211	1.492850
44.C	3.232168	1.852574	1.493717
45.C	-3.232649	2.182148	1.475631
46.C	0.127187	-3.557449	1.613122
47.C	-4.458465	-2.598023	2.206983
48.C	-0.293895	-4.972227	2.088017
49.C	-2.345365	4.474366	2.211049
50.C	4.323287	2.651586	2.249145
51.C	-4.249122	2.239357	2.643418
52.C	1.108010	-2.938032	2.637423
53.C	3.892314	-0.204477	2.896224
54.C	-3.370800	4.519714	3.351851
55.C	4.982108	0.609384	3.604981

56.C	-0.857336	-4.964762	3.520151
57.C	4.599449	2.093179	3.655037
58.C	-3.743575	3.103707	3.810932
59.C	0.539983	-2.926897	4.065909
60.C	0.125104	-4.333502	4.516142
61.H	5.552498	3.476133	-4.488351
62.H	7.205226	1.840863	-4.032522
63.H	0.528069	1.637198	-4.174967
64.H	-1.688101	0.079972	-4.174786
65.H	4.919447	0.715569	-3.964524
66.H	0.746577	-1.138399	-4.155221
67.H	-5.697662	2.617409	-3.831292
68.H	-3.025976	-6.599323	-3.468334
69.H	-0.604857	-6.100119	-3.705195
70.H	-5.027248	4.252751	-3.777524
71.H	-7.483574	4.231051	-3.212055
72.H	6.230633	4.135978	-3.001297
73.H	-0.811561	-7.639929	-2.863142
74.H	3.574746	2.714078	-3.446369
75.H	1.824112	0.820133	-3.265359
76.H	-1.634047	1.572717	-3.200988
77.H	-0.549730	-1.816508	-3.135985
78.H	-2.441575	-4.194887	-3.307677
79.H	6.129633	-0.165280	-3.038493
80.H	7.104559	2.061725	-2.289677
81.H	-3.506676	2.612593	-2.651949
82.H	3.892004	4.210656	-2.582237
83.H	-2.847266	-6.796593	-1.722706
84.H	1.525223	3.178139	-2.521569
85.H	-7.646500	2.001712	-2.126407
86.H	-3.516947	0.139271	-2.522522
87.H	1.707667	-2.666478	-2.507111
88.H	3.774351	0.298309	-1.916066
89.H	-6.506316	4.844752	-1.874064
90.H	-3.771526	-4.549591	-2.197887
91.H	-0.070081	-4.026921	-2.388163
92.H	2.227014	-1.041554	-2.109871
93.H	-2.430214	-1.180803	-2.131056
94.H	-0.128603	2.839591	-2.048293
95.H	0.937070	-6.215916	-1.779116
96.H	-5.299702	1.045605	-1.868769
97.H	-4.078331	3.832788	-1.522037
98.H	-8.194679	3.248962	-1.000277
99.H	5.161043	-3.895098	-1.475979
100.H	2.659443	-4.441231	-1.452890
101.H	4.276708	-6.365579	-1.023314
102.H	5.235500	0.447074	-0.950137
103.H	5.166343	2.785194	-0.843995
104.H	-0.400660	-6.535552	-0.671105
105.H	2.974295	7.123771	-1.044246
106.H	-4.808805	-1.721673	-1.037677
107.H	2.072937	4.743846	-1.099005

108.H	-2.024485	-3.105941	-1.115288
109.H	-6.215170	-0.683307	-0.791400
110.H	4.504893	5.193268	-0.710216
111.H	-6.772465	-3.078736	-0.362346
112.H	3.525387	-2.242236	-0.588338
113.H	-2.201195	-4.647792	-0.280433
114.H	6.338226	-5.473290	0.040381
115.H	0.554968	6.541604	-0.106484
116.H	-6.696005	1.595653	0.128983
117.H	5.861122	-2.999561	-0.122059
118.H	2.088631	-6.283332	0.159223
119.H	-6.004154	3.217438	0.165218
120.H	4.242682	7.537482	0.116180
121.H	4.427284	-7.018113	0.613207
122.H	-4.572829	-3.884209	0.469768
123.H	-2.086114	3.586109	0.239687
124.H	-3.730699	4.109528	0.599088
125.H	1.976968	8.315421	0.899013
126.H	0.539750	4.231212	0.795743
127.H	-2.964614	-1.809146	0.858122
128.H	-6.991483	-2.093716	1.089432
129.H	4.480475	0.183693	0.861563
130.H	4.223176	5.235721	1.025554
131.H	3.655615	-2.934777	1.022481
132.H	5.407088	-4.886679	1.422437
133.H	2.793707	-0.257991	1.024376
134.H	2.803823	-5.344309	1.468545
135.H	-5.821659	-4.212971	1.676009
136.H	0.304440	6.583251	1.643501
137.H	-5.409974	-0.073057	1.480327
138.H	-1.036201	-5.407369	1.402690
139.H	5.264500	2.637835	1.670147
140.H	-0.792847	-2.934652	1.620738
141.H	2.691996	7.148516	2.016391
142.H	-2.117426	5.492589	1.858251
143.H	1.810982	4.637316	1.949297
144.H	-2.319370	1.716214	1.902503
145.H	2.296336	1.961464	2.077868
146.H	0.581938	-5.641876	2.058471
147.H	-5.207490	2.650297	2.280044
148.H	-3.179030	-0.843491	2.321384
149.H	-3.732661	-3.252085	2.715731
150.H	4.028007	3.706450	2.334267
151.H	1.380816	-1.911513	2.344071
152.H	-1.400333	4.051685	2.592700
153.H	-5.137397	-2.216879	2.989622
154.H	2.047908	-3.516035	2.632914
155.H	4.188178	-1.262784	2.823129
156.H	-4.278786	5.044281	3.004105
157.H	-4.464685	1.225813	3.011416
158.H	5.938255	0.497137	3.063368
159.H	2.972900	-0.174819	3.504096

160.H	-0.070902	1.928603	2.869684
161.H	-1.801670	-4.393245	3.533611
162.H	0.989461	0.214312	3.357806
163.H	-1.107943	-5.992465	3.828363
164.H	-2.976327	5.105202	4.196785
165.H	5.393409	2.681149	4.142163
166.H	-0.333577	-2.254230	4.102216
167.H	3.693063	2.212377	4.273471
168.H	-2.856439	2.618226	4.252207
169.H	-4.508819	3.147094	4.602561
170.H	5.149153	0.218405	4.620464
171.H	1.023104	-4.971931	4.595662
172.H	1.282628	-2.504136	4.760536
173.H	-0.321765	-4.296299	5.521988
174.N	-0.117750	0.200349	-2.721954
175.N	1.330077	1.845603	-0.821544
176.N	-2.259594	0.505440	-0.799569
177.N	0.747295	-1.816509	-0.756875
178.P	-0.362898	0.527212	3.007711
179.Si	2.826624	2.597589	-0.231863
180.Si	0.779531	-3.503442	-0.189352
181.Si	-3.744900	1.029505	0.028448
182.Th	-0.090574	0.212830	0.021981

Energy: −1007.73321565 eV

Table S8. Geometry Optimised Final Coordinates and Single Point Energy for 10

1.C	4.992538	-3.538877	-3.396170
2.C	-7.043832	-1.785430	-2.918716
3.C	6.319745	-4.079189	-2.844061
4.C	-0.411291	1.231435	-3.160907
5.C	2.291159	5.550995	-2.994583
6.C	1.312938	-0.527335	-3.146379
7.C	-5.900526	-0.763232	-3.003102
8.C	-1.068107	-1.145544	-3.133691
9.C	-6.505706	-3.205351	-2.690160
10.C	3.592157	4.813623	-2.649990
11.C	1.053955	4.796069	-2.476053
12.C	4.040010	-3.120211	-2.262949
13.C	2.348005	-0.014801	-2.147239
14.C	6.972589	-3.080835	-1.876708
15.C	-1.326272	1.896334	-2.135513
16.C	-1.141527	-2.256128	-2.090310
17.C	-4.987003	-0.847358	-1.768916
18.C	-5.584686	-3.278787	-1.459209
19.C	-4.415297	-2.268453	-1.555819
20.C	-4.376273	5.156142	-1.163158
21.C	4.689783	-2.083997	-1.318589
22.C	3.685000	4.540280	-1.143520
23.C	-2.839673	7.147057	-0.806525
24.C	-4.265115	6.598359	-0.645018
25.C	-2.439933	-5.187922	-0.912758
26.C	1.129707	4.499244	-0.959963

27.C	6.008904	-2.662955	-0.751908
28.C	-1.897493	4.773026	-0.721037
29.C	-2.436979	-6.675681	-0.521647
30.C	-3.330503	4.235093	-0.509131
31.C	2.452118	3.776009	-0.632973
32.C	-1.795565	6.215704	-0.166630
33.C	5.345355	1.047081	-0.009289
34.C	-2.891819	-4.278935	0.254406
35.C	-1.550178	-6.924839	0.705694
36.C	6.274893	1.978046	0.791714
37.C	2.207064	-3.749234	0.865764
38.C	4.615200	0.039846	0.904811
39.C	2.942427	-2.487115	1.371242
40.C	-2.001386	-4.541763	1.488076
41.C	-3.776453	-0.000927	1.418143
42.C	-3.578695	-1.533504	1.469048
43.C	-0.157380	3.524850	1.699425
44.C	-1.962051	-6.028921	1.881721
45.C	5.528822	2.704798	1.920888
46.C	1.702727	-4.631311	2.020790
47.C	3.853894	0.793798	2.015852
48.C	-4.828063	-2.197459	2.104919
49.C	4.065065	-2.891293	2.361452
50.C	0.573590	4.742487	2.318546
51.C	-1.465057	3.258320	2.482851
52.C	4.777782	1.718874	2.826158
53.C	2.833312	-5.018676	2.981458
54.C	-4.132004	0.592371	2.792611
55.C	3.544718	-3.768180	3.511499
56.C	-5.357401	-0.092101	3.413537
57.C	-5.159877	-1.611294	3.486241
58.C	0.815611	4.571131	3.827686
59.C	-1.231496	3.099628	3.993849
60.C	-0.492765	4.307207	4.584301
61.H	4.511973	-4.293891	-4.038893
62.H	5.195579	-2.663277	-4.038616
63.H	2.212639	5.702120	-4.083275
64.H	-7.659200	-1.750280	-3.831024
65.H	1.515017	-0.158099	-4.170203
66.H	-0.874347	1.204640	-4.166636
67.H	-0.834584	-1.535255	-4.143320
68.H	7.008615	-4.319081	-3.669009
69.H	-5.304158	-0.956417	-3.912691
70.H	-5.936273	-3.524259	-3.581192
71.H	0.515703	1.815181	-3.234475
72.H	1.361527	-1.624346	-3.169754
73.H	3.622702	3.852765	-3.194521
74.H	-2.046934	-0.648544	-3.181501
75.H	-6.307831	0.255902	-3.104811
76.H	4.464731	5.391704	-2.990979
77.H	-7.340195	-3.916578	-2.581752
78.H	0.969451	3.845951	-3.032751

79.H	2.315553	6.556348	-2.538753
80.H	3.103311	-2.730069	-2.688183
81.H	0.148962	5.375201	-2.715491
82.H	6.128790	-5.027192	-2.310715
83.H	7.280658	-2.180264	-2.436514
84.H	3.343767	-0.278359	-2.537724
85.H	-1.612202	2.884116	-2.533018
86.H	-4.222754	5.151680	-2.257628
87.H	-7.710404	-1.512332	-2.081658
88.H	-3.870497	-2.526775	-2.487060
89.H	-1.832150	-3.029991	-2.462872
90.H	2.325100	1.091258	-2.143467
91.H	-2.609793	7.256455	-1.881277
92.H	-2.271906	1.324799	-2.074312
93.H	4.998340	-1.230258	-1.956731
94.H	-0.158770	-2.760864	-2.022942
95.H	3.758912	-4.016563	-1.685302
96.H	-3.078900	-5.040204	-1.798627
97.H	7.891266	-3.511973	-1.447645
98.H	-1.767445	4.869513	-1.819178
99.H	-4.174656	-0.106332	-1.847160
100.H	-4.985434	7.249040	-1.165110
101.H	-5.205712	-4.305342	-1.347537
102.H	-2.096148	-7.289493	-1.370679
103.H	-5.391892	4.766667	-0.988531
104.H	-1.415486	-4.908824	-1.215026
105.H	-3.429040	3.214924	-0.908849
106.H	4.603779	3.979154	-0.912770
107.H	2.438434	2.772220	-1.093975
108.H	-5.578946	-0.561256	-0.883157
109.H	-6.180335	-3.070833	-0.553157
110.H	5.932954	0.522354	-0.778992
111.H	3.761237	5.500520	-0.603866
112.H	-2.766870	8.154866	-0.367101
113.H	-3.470544	-6.993753	-0.296998
114.H	1.167810	5.481686	-0.446947
115.H	4.599904	1.657375	-0.548075
116.H	-0.787938	6.629518	-0.326973
117.H	5.785458	-3.545984	-0.129022
118.H	6.505453	-1.935765	-0.091846
119.H	-4.540491	6.619479	0.424282
120.H	6.753073	2.709162	0.119893
121.H	2.891648	-4.346099	0.238644
122.H	-0.499837	-6.710742	0.436761
123.H	-3.543084	4.161351	0.570395
124.H	1.362308	-3.470718	0.212210
125.H	-3.920871	-4.596364	0.520800
126.H	2.545343	3.605864	0.450958
127.H	-1.585825	-7.985463	0.998956
128.H	-1.954497	6.199175	0.926034
129.H	-4.576306	0.253211	0.705638
130.H	7.090877	1.375021	1.227372

131.H	-2.871674	0.505939	1.029130
132.H	5.402771	-0.557825	1.406620
133.H	-0.976273	-4.198830	1.267712
134.H	-5.698970	-2.052178	1.441166
135.H	4.805163	3.411607	1.480135
136.H	1.214856	-5.533514	1.619655
137.H	4.848886	-3.448446	1.819859
138.H	3.053532	1.401274	1.554159
139.H	1.532326	4.925183	1.811953
140.H	0.501438	2.641986	1.865193
141.H	2.207733	-1.904457	1.965956
142.H	-2.964031	-6.333518	2.233545
143.H	-0.035700	5.649326	2.158773
144.H	-4.685523	-3.284778	2.192598
145.H	-1.974740	2.365560	2.090367
146.H	-2.732728	-1.718819	2.160218
147.H	-2.341506	-3.948484	2.350164
148.H	6.232329	3.310355	2.513649
149.H	-2.156575	4.103502	2.319483
150.H	3.562101	-5.659173	2.453526
151.H	-1.275979	-6.173858	2.730919
152.H	4.552506	-1.997806	2.777439
153.H	0.932549	-4.078022	2.584473
154.H	3.345897	0.094165	2.696467
155.H	-4.307384	1.675427	2.699219
156.H	-6.251714	0.129972	2.804572
157.H	-0.200208	-1.887224	2.751444
158.H	5.507727	1.099173	3.376151
159.H	-1.260093	-0.049347	3.263743
160.H	4.196161	2.263849	3.586244
161.H	-3.270770	0.468959	3.471188
162.H	2.435009	-5.617985	3.814828
163.H	-6.060396	-2.096734	3.895176
164.H	4.378812	-4.050397	4.173546
165.H	1.502835	3.721979	3.987347
166.H	2.838847	-3.179216	4.122058
167.H	1.319293	5.464455	4.230027
168.H	-4.333627	-1.838807	4.181840
169.H	-0.637339	2.187929	4.175305
170.H	-5.551478	0.319485	4.416131
171.H	-1.137956	5.201441	4.518710
172.H	-2.194939	2.949617	4.506341
173.H	-0.291012	4.144148	5.654259
174.As	0.216524	-0.439145	3.094475
175.N	-0.055551	-0.140545	-2.714248
176.N	-0.676047	1.982853	-0.798591
177.N	2.100969	-0.569367	-0.788836
178.N	-1.550772	-1.704873	-0.767651
179.Si	-0.425073	3.636553	-0.202210
180.Si	-3.070610	-2.405658	-0.165418
181.Si	3.535554	-1.273146	0.005494
182.Th	-0.044211	-0.116353	0.017738

Energy: −1007.06907896 eV

Table S9. Geometry Optimised Final Coordinates and Single Point Energy for 11

1.C	1.989445	-0.136168	-6.283200
2.C	2.300632	-1.553254	-5.781193
3.C	-3.559980	-0.999100	-5.557118
4.C	2.344079	5.448057	-5.226885
5.C	-2.118955	-1.519106	-5.466780
6.C	1.521349	0.778189	-5.138275
7.C	-4.507377	-1.848155	-4.697674
8.C	2.796313	4.180270	-4.479748
9.C	2.077031	6.613771	-4.262977
10.C	3.308563	-1.526551	-4.624279
11.C	-1.643736	-1.603941	-4.007142
12.C	2.544291	0.831485	-3.979996
13.C	1.763153	3.763692	-3.403312
14.C	2.847924	-0.598938	-3.486431
15.C	-4.030109	-1.935161	-3.236253
16.C	1.083888	6.216356	-3.159811
17.C	-2.576124	-2.450377	-3.116643
18.C	1.302355	-5.186579	-2.734180
19.C	-0.089373	-4.543060	-2.622691
20.C	1.534895	4.940818	-2.427273
21.C	-0.721833	1.921049	-2.417385
22.C	4.784577	2.612870	-1.915892
23.C	1.718989	-5.866206	-1.424330
24.C	-0.186792	-3.533912	-1.448707
25.C	-1.622612	2.648097	-1.424130
26.C	3.478392	2.056662	-1.294064
27.C	5.971197	2.567343	-0.940194
28.C	-3.398399	-5.170741	-1.027352
29.C	-4.581627	-5.930984	-0.401353
30.C	-3.143635	0.878824	-0.624349
31.C	-3.238558	-3.751787	-0.427716
32.C	1.651939	-4.879816	-0.251710
33.C	0.266819	-4.222576	-0.137969
34.C	-2.972226	-0.505021	-0.002581
35.C	5.644039	3.315915	0.357949
36.C	3.174319	2.779600	0.037648
37.C	-4.472807	-5.991883	1.129125
38.C	4.370332	2.755596	1.003933
39.C	-3.127612	-3.838168	1.108968
40.C	-2.115407	2.474358	0.982984
41.C	-4.316825	-4.588247	1.732036
42.C	-0.746211	2.529018	1.661397
43.C	-0.680219	-2.048885	3.333191
44.C	-0.193758	-3.480813	3.612579
45.C	0.174635	-1.005081	4.080629
46.C	-3.107734	0.512007	4.231591
47.C	2.597811	1.346169	4.358097
48.C	-4.437054	1.137409	4.687212

49.C	1.099648	3.372467	4.513417
50.C	-1.899165	1.424817	4.540565
51.C	1.163114	1.834107	4.658919
52.C	3.553331	3.577807	5.140410
53.C	-0.181351	-3.791896	5.115125
54.C	3.642861	2.048412	5.243262
55.C	2.128038	4.077370	5.416057
56.C	0.196144	-1.329636	5.596485
57.C	0.662951	-2.766531	5.882335
58.C	-4.407329	1.482428	6.183278
59.C	-1.875336	1.742600	6.055768
60.C	-3.200812	2.366344	6.529179
61.H	1.227086	-0.171927	-7.078376
62.H	2.898163	0.295260	-6.739208
63.H	-3.899619	-0.984607	-6.604528
64.H	2.678691	-2.178205	-6.605407
65.H	3.100534	5.737986	-5.973698
66.H	-2.065393	-2.522472	-5.925157
67.H	-1.441482	-0.873204	-6.047922
68.H	1.420177	5.221654	-5.788642
69.H	1.321593	1.787189	-5.531765
70.H	1.364819	-2.026067	-5.435816
71.H	-4.561723	-2.867246	-5.119503
72.H	-3.590614	0.048016	-5.205362
73.H	2.958501	3.366932	-5.202223
74.H	1.703885	7.489995	-4.815361
75.H	-5.529542	-1.438317	-4.736860
76.H	4.284386	-1.172290	-5.000975
77.H	0.559484	0.398890	-4.750394
78.H	3.487776	1.239516	-4.395893
79.H	3.029362	6.921393	-3.796244
80.H	3.774326	4.374000	-4.009272
81.H	3.474548	-2.544796	-4.238325
82.H	-0.613536	-1.990661	-3.972828
83.H	0.813411	3.637804	-3.961518
84.H	1.313590	-5.909199	-3.565613
85.H	0.091071	6.039623	-3.610272
86.H	-2.572294	-3.479136	-3.530698
87.H	-0.344927	-4.060706	-3.576659
88.H	-1.591835	-0.581853	-3.594621
89.H	-0.562516	2.586182	-3.280328
90.H	2.040800	-4.404931	-2.986747
91.H	-4.717527	-2.580264	-2.667839
92.H	1.933767	-1.021550	-3.027800
93.H	5.038712	2.065208	-2.836437
94.H	-4.106019	-0.929445	-2.785593
95.H	0.960484	7.044339	-2.443332
96.H	3.610375	-0.585470	-2.692184
97.H	-1.267912	1.050811	-2.821652
98.H	-0.835471	-5.342811	-2.474344
99.H	4.625586	3.663431	-2.214006
100.H	-3.529556	-5.121779	-2.119693

101.H	2.475828	5.154240	-1.891113
102.H	-2.541742	3.036404	-1.903540
103.H	6.866121	2.998262	-1.417430
104.H	0.797921	4.671657	-1.653467
105.H	2.732054	-6.287343	-1.515136
106.H	0.548116	-2.725945	-1.663421
107.H	-3.187101	0.768651	-1.716354
108.H	1.042367	-6.717539	-1.227102
109.H	-4.640271	-6.948041	-0.821083
110.H	3.667618	0.994240	-1.041466
111.H	-1.071690	3.503731	-1.011041
112.H	6.211696	1.515923	-0.705658
113.H	-2.474869	-5.749118	-0.851434
114.H	-5.521765	-5.422566	-0.678036
115.H	-4.191834	-3.225525	-0.640527
116.H	2.414977	-4.095108	-0.387387
117.H	-4.081637	1.366052	-0.297066
118.H	3.017458	-1.603184	-0.368367
119.H	-3.862702	-1.102887	-0.263271
120.H	5.500431	4.387920	0.133055
121.H	2.890596	3.827528	-0.157453
122.H	-0.468546	-5.002114	0.127140
123.H	1.899261	-5.387355	0.693241
124.H	0.271702	-3.504241	0.695459
125.H	6.487468	3.254282	1.063020
126.H	2.300891	2.318942	0.535878
127.H	-2.539328	3.480542	0.805076
128.H	3.241977	-0.140868	1.101377
129.H	-2.991790	-0.412615	1.099294
130.H	-0.029195	3.021721	0.973100
131.H	-3.595500	-6.602570	1.406523
132.H	-5.352645	-6.498985	1.555186
133.H	4.556877	1.714348	1.318200
134.H	-2.197655	-4.363166	1.379632
135.H	-5.242184	-4.011658	1.554441
136.H	-3.045744	-2.832758	1.547084
137.H	-2.809565	1.922374	1.629143
138.H	4.124482	3.320001	1.916930
139.H	-0.664416	-1.862788	2.243886
140.H	-4.193261	-4.653756	2.824722
141.H	-0.794016	3.199780	2.534067
142.H	-0.828431	-4.206413	3.082082
143.H	-3.153110	0.272324	3.156776
144.H	0.826449	-3.593222	3.207826
145.H	2.830741	1.534968	3.296219
146.H	1.305552	3.646724	3.464019
147.H	-1.737380	-1.960707	3.635386
148.H	1.209513	-1.133376	3.704814
149.H	-4.621084	2.058952	4.105527
150.H	3.852686	3.890265	4.124598
151.H	-2.103545	2.392702	4.037436
152.H	-5.274572	0.455852	4.469023

153.H	2.679407	0.258359	4.498860
154.H	-2.975487	-0.453058	4.750900
155.H	0.089943	3.750380	4.743664
156.H	4.655474	1.707170	4.975104
157.H	2.072736	5.169078	5.276112
158.H	0.197797	-4.810450	5.291035
159.H	-1.216952	-3.771839	5.500410
160.H	4.266600	4.050947	5.833313
161.H	1.719899	-2.869131	5.581822
162.H	0.972129	1.614268	5.729619
163.H	-3.323806	3.352428	6.046248
164.H	-0.819674	-1.200894	6.010450
165.H	-5.344002	1.978555	6.481281
166.H	3.479743	1.747164	6.293222
167.H	0.841241	-0.620383	6.137515
168.H	1.871206	3.882445	6.472089
169.H	-1.046248	2.422591	6.299741
170.H	-4.350371	0.546587	6.766572
171.H	-1.691747	0.815681	6.625998
172.H	0.620826	-2.966985	6.964814
173.H	-3.164398	2.553566	7.614409
174.N	0.547695	1.501593	-1.767482
175.N	-1.699932	-1.129719	-0.452351
176.N	-1.969592	1.733979	-0.301462
177.N	-0.285185	1.155423	2.012376
178.P	2.470525	-1.340580	0.934589
179.Si	2.044606	2.038072	-2.569341
180.Si	-1.905701	-2.672207	-1.315533
181.Si	-0.223078	0.838440	3.762328
182.U	0.140215	0.140836	0.033933

Energy: −1007.97759693 eV

Table S10. Geometry Optimised Final Coordinates and Single Point Energy for 12

1.C	-0.402930	0.073713	-7.521808
2.C	-0.412481	-1.451580	-7.342372
3.C	-1.122475	0.774320	-6.360275
4.C	0.166184	-1.871185	-5.978316
5.C	-0.533952	0.357708	-5.002758
6.C	-0.556323	-1.171812	-4.800414
7.C	1.512976	4.690597	-4.011190
8.C	4.798721	-1.628732	-3.651857
9.C	1.492165	3.239722	-3.511200
10.C	2.647101	-2.961168	-3.418858
11.C	-2.375944	-5.411398	-3.257385
12.C	-2.029677	-3.912297	-3.303802
13.C	2.581836	-0.436710	-3.292952
14.C	4.161494	-2.931631	-3.153484
15.C	4.097832	-0.410481	-3.037287
16.C	1.571823	5.674258	-2.836256
17.C	-0.545802	-3.634835	-2.967712
18.C	1.911630	-1.742671	-2.801214
19.C	0.330984	2.994187	-2.532876

20.C	-4.961173	5.516106	-2.082952
21.C	-4.657877	4.011855	-2.142635
22.C	-2.005219	-6.036595	-1.905544
23.C	-2.189563	-0.451327	-2.048880
24.C	0.425589	5.426584	-1.842891
25.C	-3.169873	3.732957	-1.872813
26.C	-0.524268	-5.800893	-1.582040
27.C	-0.173512	-4.305250	-1.629741
28.C	0.384806	3.962017	-1.334127
29.C	-4.481865	6.133072	-0.760632
30.C	-3.063878	-0.960776	-0.905824
31.C	-2.997230	5.830871	-0.485922
32.C	-2.708517	4.310801	-0.515099
33.C	3.132728	-5.479295	0.735812
34.C	-3.216128	0.888790	0.749785
35.C	3.885852	-4.201094	1.130511
36.C	-0.395261	4.927946	1.461620
37.C	2.923821	-3.058280	1.499284
38.C	-2.714457	-1.403616	1.492448
39.C	-1.482032	-2.300935	1.510693
40.C	-2.170422	1.751490	1.455966
41.C	2.133417	-5.896584	1.823937
42.C	1.095080	4.805629	1.850373
43.C	1.172171	-4.748486	2.178107
44.C	1.924155	-3.465476	2.602933
45.C	1.521117	5.885147	2.860386
46.C	-1.266437	4.893934	2.738387
47.C	-0.857123	5.988420	3.740879
48.C	1.833451	-0.583897	3.761637
49.C	0.629876	5.889334	4.110099
50.C	1.092699	0.757755	3.964610
51.C	2.022061	1.867424	4.484229
52.C	-0.392781	-2.764313	4.525282
53.C	2.600726	-0.972182	5.051659
54.C	-1.305337	-1.739291	5.236152
55.C	0.292098	-3.667047	5.580276
56.C	3.532176	0.144613	5.547581
57.C	2.757278	1.450728	5.764795
58.C	-2.350456	-2.428191	6.130967
59.C	-0.749963	-4.374532	6.464606
60.C	-1.690636	-3.371192	7.148917
61.H	-0.864800	0.349000	-8.482851
62.H	0.153313	-1.934182	-8.155288
63.H	0.642410	0.427892	-7.564719
64.H	-1.451131	-1.818442	-7.421207
65.H	-1.063787	1.868309	-6.478150
66.H	-2.195784	0.513577	-6.385915
67.H	1.238964	-1.611942	-5.956223
68.H	0.110815	-2.966483	-5.876650
69.H	-1.619522	-1.480988	-4.869805
70.H	0.506797	0.715224	-4.943937
71.H	2.365347	4.848083	-4.690234

72.H	4.719320	-1.583280	-4.752742
73.H	0.600357	4.883812	-4.603609
74.H	2.480236	-2.969966	-4.509980
75.H	1.424037	2.546830	-4.362791
76.H	-2.282294	-3.510026	-4.296911
77.H	2.406756	-0.330466	-4.377427
78.H	-1.824348	-5.934042	-4.058454
79.H	-1.074081	0.862022	-4.188540
80.H	4.640291	-3.802869	-3.628935
81.H	0.054964	-4.129371	-3.757899
82.H	5.874033	-1.611204	-3.415007
83.H	-3.447661	-5.557658	-3.467227
84.H	4.526238	0.522067	-3.437777
85.H	1.533488	6.713060	-3.201684
86.H	-4.450026	6.018057	-2.923495
87.H	-4.953355	3.600748	-3.121291
88.H	2.444181	3.018751	-2.997940
89.H	2.230261	-3.904877	-3.038600
90.H	-0.624200	3.107584	-3.072094
91.H	-2.573106	-0.890523	-2.986483
92.H	2.115615	0.439801	-2.818065
93.H	-2.572709	4.186976	-2.682453
94.H	-2.678246	-3.385173	-2.581631
95.H	-6.038797	5.696026	-2.221089
96.H	2.536390	5.557469	-2.312713
97.H	0.098553	-6.344972	-2.313490
98.H	-0.529800	5.662474	-2.344149
99.H	4.338618	-3.028657	-2.068074
100.H	-2.234485	-7.113693	-1.902015
101.H	0.355560	1.940438	-2.195759
102.H	4.282626	-0.393694	-1.949360
103.H	-2.332821	0.639732	-2.155435
104.H	-2.973852	2.649720	-1.924733
105.H	2.077188	-1.795745	-1.702155
106.H	-5.266695	3.487472	-1.384160
107.H	0.895541	-4.166406	-1.407348
108.H	-2.381296	6.340631	-1.246316
109.H	-2.626331	-5.582887	-1.112127
110.H	-4.651270	7.221682	-0.766848
111.H	0.512613	6.128337	-0.999334
112.H	-4.131228	-0.715548	-1.063528
113.H	2.821453	1.774772	-0.802304
114.H	-0.715532	-3.793782	-0.816663
115.H	1.347691	3.766364	-0.822114
116.H	-2.976970	-2.054781	-0.859344
117.H	-0.277349	-6.214342	-0.591300
118.H	-5.087760	5.727040	0.068858
119.H	2.585713	-5.301278	-0.206511
120.H	-3.526229	1.393455	-0.173787
121.H	-3.376726	3.868581	0.252443
122.H	-2.710789	6.264999	0.483324
123.H	4.551872	-3.882736	0.312826

124.H	3.842869	-6.296243	0.532742
125.H	-1.413793	-2.825762	0.542131
126.H	2.358679	-2.760351	0.596121
127.H	1.738591	4.872622	0.961048
128.H	-0.527916	5.936834	1.019251
129.H	1.563377	-6.782163	1.500261
130.H	-4.118342	0.725396	1.369700
131.H	2.524450	1.905457	1.355763
132.H	-3.650434	-1.984370	1.383089
133.H	0.549083	-4.522570	1.294964
134.H	4.535742	-4.415268	1.996990
135.H	-2.651225	2.663809	1.844922
136.H	3.503590	-2.172009	1.796853
137.H	1.463134	6.873648	2.370822
138.H	-1.642682	-3.094065	2.256401
139.H	1.276918	3.806652	2.281671
140.H	2.687289	-6.194725	2.731265
141.H	-1.814287	1.217285	2.359448
142.H	-2.334903	5.006085	2.490581
143.H	-2.767515	-0.857149	2.443539
144.H	0.481281	-5.079598	2.969436
145.H	2.575321	5.738945	3.144264
146.H	-1.055412	6.977297	3.291327
147.H	2.595529	-0.413958	2.974850
148.H	-1.158102	3.909025	3.225444
149.H	0.633065	1.093060	3.016706
150.H	2.517889	-3.716957	3.505065
151.H	2.765569	2.104881	3.704256
152.H	-1.070891	-3.446712	3.974571
153.H	-1.479340	5.925507	4.647967
154.H	0.914106	6.715833	4.780075
155.H	0.798125	4.957201	4.677438
156.H	-1.809098	-1.088592	4.503916
157.H	0.258312	0.624041	4.673944
158.H	1.443112	2.787631	4.658646
159.H	3.177924	-1.896448	4.893681
160.H	4.330616	0.312829	4.804931
161.H	0.934320	-4.417290	5.096025
162.H	-3.041659	-3.007682	5.493080
163.H	-1.344685	-5.061421	5.835838
164.H	1.873571	-1.193295	5.852041
165.H	-0.685651	-1.071894	5.860839
166.H	3.436329	2.253301	6.092000
167.H	0.954560	-3.062895	6.222265
168.H	4.026863	-0.168564	6.480825
169.H	2.024525	1.308097	6.579336
170.H	-2.962733	-1.674669	6.652140
171.H	-0.246850	-4.999112	7.219713
172.H	-2.460439	-3.903336	7.729385
173.H	-1.111825	-2.771882	7.873349
174.As	2.975839	0.801169	0.380776
175.N	-0.758459	-0.769479	-1.800310

176.N	-2.584684	-0.410732	0.390618
177.N	-1.038260	2.045508	0.532514
178.N	-0.255850	-1.501714	1.771512
179.Si	0.000400	-1.781984	-3.054261
180.Si	-0.935422	3.749557	0.035979
181.Si	0.751016	-2.041090	3.140435
182.U	0.036281	0.085187	0.167283

Energy: –1007.02852553 eV

Table S11. Geometry Optimised Final Coordinates and Single Point Energy for neutral triplet AsH

1.As	0.100000	0.100000	0.099842
2.H	0.100000	0.100000	1.641775

Energy: –5.68034740 eV

Table S12. Geometry Optimised Final Coordinates and Single Point Energy for singlet $(AsH)^{2-}$

1.As	0.100000	0.100000	0.099350
2.H	0.100000	0.100000	1.678340

Energy: –2.26424576 eV

References

1. B. J. Barker, P. G. Sears. *J. Phys. Chem.* **1974**, *78*, 2687.
2. M. F. Kühnel, D. Lentz. *Dalton Trans.* **2009**, 4747.
3. R. Klement, G. Brauer. *Handbuch der Präparativen Anorganischen Chemie*, Ferdinand Enke Verlag, 1975.
4. W. C. Johnson, A. Pechukas. *J. Am. Chem. Soc*. **1937**, *59*, 2068.
5. D. E. Bergbreiter, J. M. Killough. *J. Am. Chem. Soc.* **1978**, *100*, 2126.
6. T. Cantat, B. L. Scott, J. L. Kiplinger. *Chem. Commun.* **2010**, *46*, 919.
7. J. L. Kiplinger, K. D. John, D. E. Morris, B. L. Scott, C. J. Burns. *Organometallics*, **2002**, *21*, 4306.
8. G. M. Sheldrick. *Acta Cryst. Sect. A* **2015**, *A71*, 3.
9. CrysAlisPRO version 39.46, Oxford Diffraction/Agilent Technologies UK Ltd, Yarnton, England.
10. G. M. Sheldrick. *Acta Cryst. Sect. C* **2015**, *C71*, 3.

11. O. V. Dolomanov, L. J. Bourhis, R. J. Gildea, J. A. K. Howard, H. Puschmann. *J. Appl. Cryst.* **2009**, *42*, 339.

12. L. J. Farugia. *J. Appl. Cryst.* **2012**, *45*, 849.

13. Persistence of Vision (TM) Raytracer, Persistence of Vision Pty. Ltd., Williamstown, Victoria, Australia.

14. B. E. Lindfors, J. L. Male, D. R. Tyler, T. J. R. Weakley. *Acta Cryst.* **1998**. *C54*, 694.

15. P. B. Hitchcock, M. F. Lappert, L. Maron, A. V. Protchenko. *Angew. Chem. Int. Ed.* **2008**, *47*, 1488.

16. C. Fonseca Guerra, J. G. Snijders, G. Te Velde, E. J. Baerends, *Theor. Chem. Acc.* **1998**, *99*, 391.

17. G. Te Velde, F. M. Bickelhaupt, S. J. van Gisbergen, A. C. Fonseca Guerra, E. J. Baerends, J. G. Snijders, T. Ziegler, *J. Comput. Chem.* **2001**, 22, 931.

18. E. Van Lenthe, E. J. Baerends, J. G. Snijders, *J. Chem. Phys.* **1993**, *99*, 4597.

19. E. Van Lenthe, E. J. Baerends, J. G. Snijders, *J. Chem. Phys.* **1994**, *101*, 9783.

20. E. Van Lenthe, A. E. Ehlers, E. J. Baerends, *J. Chem. Phys.* **1999**, *110*, 8943.

21. S. H. Vosko, L. Wilk, M. Nusair, *Can. J. Phys*. **1980**, *58*, 1200.

22. A. D. Becke, *Phys. Rev. A*. **1988**, *38*, 3098.

23. J. P. Perdew, *Phys. Rev. B*. **1986**, *33*, 8822.

24. NBO 6.0. E. D. Glendening, J. K. Badenhoop, A. E. Reed, J. E. Carpenter, J. A. Bohmann, C. M. Morales, C. R. Landis, F. Weinhold (Theoretical Chemistry Institute, University of Wisconsin, Madison, WI, 2013); http://nbo6.chem.wisc.edu/.

25. R. F. W. Bader, *Atoms in Molecules: A Quantum Theory*, Oxford University Press, New York, 1990.

26. R. F. W. Bader, *J. Phys. Chem. A* **1998**, *102*, 7314.
