# Supplementary material for: Actinide Pnictinidene Chemistry: A Terminal Thorium Parent‐Arsinidene Complex Stabilised by a Super‐Bulky Triamidoamine Ligand
Source: Angew Chem Int Ed Engl. 2022 Nov 16;61(50):e202211627. doi: 10.1002/anie.202211627 (PMC10099757; doi:10.1002/anie.202211627)

checkCIF/PLATON report

Structure factors have been supplied for datablock(s) 1, 10, 11, 12, 2, 3, 4, 5, 6, 7, 8, 9, TRENTCHSLi3

THIS REPORT IS FOR GUIDANCE ONLY. IF USED AS PART OF A REVIEW PROCEDURE FOR PUBLICATION, IT SHOULD NOT REPLACE THE EXPERTISE OF AN EXPERIENCED CRYSTALLOGRAPHIC REFEREE.

No syntax errors found. CIF dictionary Interpreting this report

Datablock: TRENTCHSLi3

Bond precision: C-C = 0.0088 A Wavelength=1.54184

Cell:	a=13.3044(3)	b=13.3300(4)	c=22.3221(6)
	alpha=76.010(2)	beta=80.531(2)	gamma=70.341(2)
Temperature:	150 K		

	Calculated	Reported
Volume	3602.42(17)	3602.42(18)
Space group	P -1	P -1
Hall group	-P 1	-P 1
Moiety formula	C60 H111 Li3 N4 Si3, 2.5(C6 H6)	C60 H111 Li3 N4 Si3, 2.5(C6 H6)
Sum formula	C75 H126 Li3 N4 Si3	C75 H126 Li3 N4 Si3
Mr	1188.89	1188.88
Dx,g cm-3	1.096	1.096
Z	2	2
Mu (mm-1)	0.913	0.913
F000	1310.0	1310.0
F000'	1314.30	
h,k,lmax	16,16,27	16,16,27
Nref	13672	13633
Tmin,Tmax	0.630,0.751	0.254,1.000
Tmin'	0.569	

Correction method= # Reported T Limits: Tmin=0.254 Tmax=1.000
AbsCorr = GAUSSIAN

Data completeness= 0.997 Theta(max)= 70.074

R(reflections)= 0.1191(11484) wR2(reflections)= 0.3329(13633)

S = 1.123 Npar= 766

The following ALERTS were generated. Each ALERT has the format
test-name_ALERT_alert-type_alert-level.
Click on the hyperlinks for more details of the test.

Alert level B

```
PLAT097_ALERT_2_B Large Reported Max.  (Positive) Residual Density       1.79 eA-3
```

Author Response: There are several large residual density peaks, that appear to be the silicon atoms of a minor twinned component. A twin law could not be found so this was left as is.

Alert level C

```
DIFMX02_ALERT_1_C  The maximum difference density is > 0.1*ZMAX*0.75
            The relevant atom site should be identified.
PLAT082_ALERT_2_C High R1 Value ..................................       0.12 Report
PLAT084_ALERT_3_C High wR2 Value (i.e. > 0.25) ...................       0.33 Report
PLAT094_ALERT_2_C Ratio of Maximum / Minimum Residual Density ....       3.57 Report
PLAT241_ALERT_2_C High   'MainMol' Ueq as Compared to Neighbors of        Li1 Check
PLAT250_ALERT_2_C Large U3/U1 Ratio for Average U(i,j) Tensor ....        2.7 Note
PLAT331_ALERT_2_C Small Aver Phenyl C-C Dist C61      --C66      .       1.37 Ang.
PLAT340_ALERT_3_C Low Bond Precision on  C-C Bonds ...............    0.00881 Ang.
PLAT906_ALERT_3_C Large K Value in the Analysis of Variance ......     12.800 Check
PLAT906_ALERT_3_C Large K Value in the Analysis of Variance ......      2.346 Check
PLAT911_ALERT_3_C Missing FCF Refl Between Thmin & STh/L=    0.600         17 Report
PLAT971_ALERT_2_C Check Calcd Resid. Dens.  2.04Ang From C54             1.95 eA-3
PLAT971_ALERT_2_C Check Calcd Resid. Dens.  2.32Ang From C24             1.93 eA-3
PLAT971_ALERT_2_C Check Calcd Resid. Dens.  1.90Ang From Li2             1.87 eA-3
```

Alert level G

```
PLAT002_ALERT_2_G Number of Distance or Angle Restraints on AtSite         15 Note
PLAT072_ALERT_2_G SHELXL First  Parameter in WGHT  Unusually Large       0.17 Report
PLAT083_ALERT_2_G SHELXL Second Parameter in WGHT  Unusually Large       8.51 Why ?
PLAT154_ALERT_1_G The s.u.'s on the Cell Angles are Equal ..(Note)      0.002 Degree
PLAT172_ALERT_4_G The CIF-Embedded .res File Contains DFIX Records          1 Report
PLAT802_ALERT_4_G CIF Input Record(s) with more than 80 Characters          1 Info
PLAT860_ALERT_3_G Number of Least-Squares Restraints .............         14 Note
PLAT912_ALERT_4_G Missing # of FCF Reflections Above STh/L=  0.600         22 Note
PLAT933_ALERT_2_G Number of HKL-OMIT Records in Embedded .res File          3 Note
PLAT941_ALERT_3_G Average HKL Measurement Multiplicity ...........        3.9 Low
PLAT978_ALERT_2_G Number C-C Bonds with Positive Residual Density.          1 Info
PLAT992_ALERT_5_G Repd & Actual _reflns_number_gt Values Differ by          2 Check
```

0 **ALERT level A** = Most likely a serious problem - resolve or explain
1 **ALERT level B** = A potentially serious problem, consider carefully
14 **ALERT level C** = Check. Ensure it is not caused by an omission or oversight
12 **ALERT level G** = General information/check it is not something unexpected

2 ALERT type 1 CIF construction/syntax error, inconsistent or missing data

```
 14 ALERT type 2 Indicator that the structure model may be wrong or deficient
  7 ALERT type 3 Indicator that the structure quality may be low
  3 ALERT type 4 Improvement, methodology, query or suggestion
  1 ALERT type 5 Informative message, check
```

Datablock: 1

Bond precision: C-C = 0.0180 A Wavelength=1.54184

Cell:	a=9.7011(3)	b=12.8832(4)	c=26.2215(10)
	alpha=97.198(3)	beta=100.144(3)	gamma=92.856(2)
Temperature:	150 K		

	Calculated	Reported
Volume	3191.86(19)	3191.86(19)
Space group	P -1	P -1
Hall group	-P 1	-P 1
Moiety formula	2(C60 H111 Cl N4 Si3 Th), C7 H8	C60 H111 Cl N4 Si3 Th, 0.5(C7 H8)
Sum formula	C127 H230 Cl2 N8 Si6 Th2	C63.50 H115 Cl N4 Si3 Th
Mr	2572.72	1286.35
Dx,g cm-3	1.339	1.338
Z	1	2
Mu (mm-1)	8.749	8.749
F000	1346.0	1346.0
F000’	1342.60	
h,k,lmax	12,16,33	12,16,32
Nref	13722	12981
Tmin,Tmax	0.504,0.634	0.770,0.902
Tmin’	0.453	

Correction method= # Reported T Limits: Tmin=0.770 Tmax=0.902
AbsCorr = GAUSSIAN

Data completeness= 0.946 Theta(max)= 78.477

R(reflections)= 0.0853(9435) wR2(reflections)= 0.2430(12981)

S = 1.054 Npar= 686

The following ALERTS were generated. Each ALERT has the format
test-name_ALERT_alert-type_alert-level.
Click on the hyperlinks for more details of the test.

Alert level A

PLAT971_ALERT_2_A Check Calcd Resid. Dens. 0.92Ang From N1 3.55 eA-3

Author Response: Residual density is close to a heavy atom, and is present despite applying the correct absorption correction. It is not chemically significant.

PLAT972_ALERT_2_A Check Calcd Resid. Dens. 0.80Ang From Th1 -7.40 eA-3

Author Response: Residual density is close to a heavy atom, and is present despite applying the correct absorption correction. It is not chemically significant.

PLAT972_ALERT_2_A Check Calcd Resid. Dens. 0.84Ang From Th1 -4.96 eA-3

Author Response: Residual density is close to a heavy atom, and is present despite applying the correct absorption correction. It is not chemically significant.

PLAT972_ALERT_2_A Check Calcd Resid. Dens. 0.85Ang From Th1 -4.69 eA-3

Author Response: Residual density is close to a heavy atom, and is present despite applying the correct absorption correction. It is not chemically significant.

Alert level B

PLAT971_ALERT_2_B Check Calcd Resid. Dens. 1.42Ang From Th1 3.09 eA-3

Author Response: Residual density is close to a heavy atom, and is present despite applying the correct absorption correction. It is not chemically significant.

PLAT972_ALERT_2_B Check Calcd Resid. Dens. 0.91Ang From Th1 -3.36 eA-3

Author Response: Residual density is close to a heavy atom, and is present despite applying the correct absorption correction. It is not chemically significant.

```
PLAT973_ALERT_2_B Check Calcd Positive Resid. Density on       Th1       1.97 eA-3
```

Author Response: Residual density is close to a heavy atom, and is present despite applying the correct absorption correction. It is not chemically significant.

Alert level C

```
RINTA01_ALERT_3_C  The value of Rint is greater than 0.12
            Rint given   0.135
PLAT020_ALERT_3_C The Value of Rint is Greater Than 0.12 .........      0.135 Report
PLAT042_ALERT_1_C Calc. and Reported MoietyFormula Strings  Differ     Please Check
PLAT213_ALERT_2_C Atom N4              has ADP max/min Ratio .....        3.2 oblate
PLAT342_ALERT_3_C Low Bond Precision on  C-C Bonds ..............    0.01802 Ang.
PLAT911_ALERT_3_C Missing FCF Refl Between Thmin & STh/L=    0.600         74 Report
PLAT971_ALERT_2_C Check Calcd Resid. Dens.  1.48Ang From Th1           2.08 eA-3
```

Author Response: Residual density is close to a heavy atom, and is present despite applying the correct absorption correction. It is not chemically significant.

```
PLAT971_ALERT_2_C Check Calcd Resid. Dens.  1.23Ang From N1            1.98 eA-3
```

Author Response: Residual density is close to a heavy atom, and is present despite applying the correct absorption correction. It is not chemically significant.

```
PLAT971_ALERT_2_C Check Calcd Resid. Dens.  1.35Ang From Th1           1.82 eA-3
```

Author Response: Residual density is close to a heavy atom, and is present despite applying the correct absorption correction. It is not chemically significant.

```
PLAT971_ALERT_2_C Check Calcd Resid. Dens.  1.38Ang From Th1           1.72 eA-3
```

Author Response: Residual density is close to a heavy atom, and is present despite applying the correct absorption correction. It is not chemically significant.

```
PLAT971_ALERT_2_C Check Calcd Resid. Dens.  1.02Ang From N2            1.52 eA-3
```

Author Response: Residual density is close to a heavy atom, and is present despite applying the correct absorption correction. It is not chemically significant.

```
PLAT972_ALERT_2_C Check Calcd Resid. Dens.  0.79Ang From Th1           -2.32 eA-3
```

Author Response: Residual density is close to a heavy atom, and is present despite applying the correct absorption correction. It is not chemically significant.

```
PLAT972_ALERT_2_C Check Calcd Resid. Dens.  1.67Ang From C22           -1.83 eA-3
```

Author Response: Residual density is close to a heavy atom, and is present despite applying the correct absorption correction. It is not chemically significant.

```
PLAT972_ALERT_2_C Check Calcd Resid. Dens.  1.86Ang From Th1           -1.59 eA-3
```

Author Response: Residual density is close to a heavy atom, and is present despite applying the correct absorption correction. It is not chemically significant.

```
PLAT976_ALERT_2_C Check Calcd Resid. Dens.  0.99Ang From N2      .      -1.15 eA-3
PLAT977_ALERT_2_C Check Negative Difference Density on H10A      .      -0.38 eA-3
PLAT977_ALERT_2_C Check Negative Difference Density on H12A      .      -0.33 eA-3
PLAT977_ALERT_2_C Check Negative Difference Density on H15       .      -0.36 eA-3
PLAT977_ALERT_2_C Check Negative Difference Density on H20A      .      -0.44 eA-3
PLAT977_ALERT_2_C Check Negative Difference Density on H24A      .      -0.40 eA-3
PLAT977_ALERT_2_C Check Negative Difference Density on H25A      .      -0.37 eA-3
```

Alert level G

```
PLAT002_ALERT_2_G Number of Distance or Angle Restraints on AtSite          7 Note
PLAT003_ALERT_2_G Number of Uiso or Uij Restrained non-H Atoms ...          7 Report
PLAT045_ALERT_1_G Calculated and Reported Z Differ by a Factor ...      0.500 Check
PLAT072_ALERT_2_G SHELXL First  Parameter in WGHT  Unusually Large       0.14 Report
PLAT172_ALERT_4_G The CIF-Embedded .res File Contains DFIX Records          2 Report
PLAT176_ALERT_4_G The CIF-Embedded .res File Contains SADI Records          1 Report
PLAT178_ALERT_4_G The CIF-Embedded .res File Contains SIMU Records          1 Report
PLAT187_ALERT_4_G The CIF-Embedded .res File Contains RIGU Records          1 Report
PLAT300_ALERT_4_G Atom Site Occupancy of C61        Constrained at        0.5 Check
PLAT300_ALERT_4_G Atom Site Occupancy of C62        Constrained at        0.5 Check
PLAT300_ALERT_4_G Atom Site Occupancy of C63        Constrained at        0.5 Check
PLAT300_ALERT_4_G Atom Site Occupancy of C64        Constrained at        0.5 Check
PLAT300_ALERT_4_G Atom Site Occupancy of C65        Constrained at        0.5 Check
PLAT300_ALERT_4_G Atom Site Occupancy of C66        Constrained at        0.5 Check
PLAT300_ALERT_4_G Atom Site Occupancy of C67        Constrained at        0.5 Check
PLAT300_ALERT_4_G Atom Site Occupancy of H61A       Constrained at        0.5 Check
PLAT300_ALERT_4_G Atom Site Occupancy of H61B       Constrained at        0.5 Check
PLAT300_ALERT_4_G Atom Site Occupancy of H61C       Constrained at        0.5 Check
PLAT300_ALERT_4_G Atom Site Occupancy of H63        Constrained at        0.5 Check
PLAT300_ALERT_4_G Atom Site Occupancy of H64        Constrained at        0.5 Check
PLAT300_ALERT_4_G Atom Site Occupancy of H65        Constrained at        0.5 Check
PLAT300_ALERT_4_G Atom Site Occupancy of H66        Constrained at        0.5 Check
PLAT300_ALERT_4_G Atom Site Occupancy of H67        Constrained at        0.5 Check
```

```
PLAT302_ALERT_4_G Anion/Solvent/Minor-Residue Disorder (Resd  2  )       100% Note
PLAT304_ALERT_4_G Non-Integer Number of Atoms in ..... (Resd  2  )       7.50 Check
PLAT789_ALERT_4_G Atoms with Negative _atom_site_disorder_group  #         15 Check
PLAT794_ALERT_5_G Tentative Bond Valency for Th1       (IV)      .       4.25 Info
PLAT802_ALERT_4_G CIF Input Record(s) with more than 80 Characters          2 Info
PLAT860_ALERT_3_G Number of Least-Squares Restraints ............          95 Note
PLAT910_ALERT_3_G Missing # of FCF Reflection(s) Below Theta(Min).          1 Note
PLAT912_ALERT_4_G Missing # of FCF Reflections Above STh/L=  0.600        654 Note
PLAT941_ALERT_3_G Average HKL Measurement Multiplicity ...........        2.6 Low
PLAT978_ALERT_2_G Number C-C Bonds with Positive Residual Density.          0 Info
```

---

4 **ALERT level A** = Most likely a serious problem - resolve or explain
3 **ALERT level B** = A potentially serious problem, consider carefully
21 **ALERT level C** = Check. Ensure it is not caused by an omission or oversight
33 **ALERT level G** = General information/check it is not something unexpected

2 ALERT type 1 CIF construction/syntax error, inconsistent or missing data
27 ALERT type 2 Indicator that the structure model may be wrong or deficient
7 ALERT type 3 Indicator that the structure quality may be low
24 ALERT type 4 Improvement, methodology, query or suggestion
1 ALERT type 5 Informative message, check

Datablock: 2

Bond precision: C-C = 0.0100 A Wavelength=1.54184

Cell:	a=25.6645(5)	b=9.5986(2)	c=25.7546(4)
	alpha=90	beta=97.994(2)	gamma=90
Temperature:	150 K		

	Calculated	Reported
Volume	6282.8(2)	6282.8(2)
Space group	P 2/c	P 1 2/c 1
Hall group	-P 2yc	-P 2yc
Moiety formula	C60 H111 Cl N4 Si3 U, 0.5(C6 H14)	C60 H111 Cl N4 Si3 U, 0.5 C6 H14
Sum formula	C63 H118 Cl N4 Si3 U	C63 H118 Cl N4 Si3 U
Mr	1289.37	1289.36
Dx,g cm-3	1.363	1.363
Z	4	4
Mu (mm-1)	8.505	8.505
F000	2700.0	2700.0
F000’	2692.38	
h,k,lmax	32,12,32	32,11,32
Nref	13217	13012
Tmin,Tmax	0.406,0.481	0.681,1.000
Tmin’	0.216	

```
Correction method= # Reported T Limits: Tmin=0.681 Tmax=1.000
AbsCorr = GAUSSIAN

Data completeness= 0.984          Theta(max)= 76.489

R(reflections)= 0.0455( 10745)              wR2(reflections)= 0.1245( 13012)

S = 1.083                  Npar= 650
```

The following ALERTS were generated. Each ALERT has the format
test-name_ALERT_alert-type_alert-level.
Click on the hyperlinks for more details of the test.

Alert level B

```
PLAT972_ALERT_2_B Check Calcd Resid. Dens.  0.95Ang From U1            -2.85 eA-3
```

Author Response: Residual density is close to a heavy atom and present despite applying the corect absorption correction. It is not chemically significant.

```
PLAT972_ALERT_2_B Check Calcd Resid. Dens.  0.83Ang From U1            -2.58 eA-3
```

Author Response: Residual density is close to a heavy atom and present despite applying the corect absorption correction. It is not chemically significant.

Alert level C

```
PLAT042_ALERT_1_C Calc. and Reported MoietyFormula Strings  Differ     Please Check
PLAT260_ALERT_2_C Large Average Ueq of Residue Including      C101      0.156 Check
PLAT342_ALERT_3_C Low Bond Precision on  C-C Bonds ..............    0.01003 Ang.
PLAT411_ALERT_2_C Short Inter H...H Contact  H31B      ..H44B     .       2.13 Ang.
                                               1-x,y,3/2-z  =      2_656 Check
PLAT411_ALERT_2_C Short Inter H...H Contact  H10D      ..H43A     .       2.12 Ang.
                                                1-x,-y,1-z  =      3_656 Check
PLAT911_ALERT_3_C Missing FCF Refl Between Thmin & STh/L=    0.600          2 Report
PLAT972_ALERT_2_C Check Calcd Resid. Dens.  0.89Ang From U1            -2.46 eA-3
```

Author Response: Residual density is close to a heavy atom and present despite applying the corect absorption correction. It is not chemically significant.

```
PLAT972_ALERT_2_C Check Calcd Resid. Dens.  0.99Ang From U1            -2.25 eA-3
```

Author Response: Residual density is close to a heavy atom and present despite applying the corect absorption correction. It is not chemically significant.

```
PLAT977_ALERT_2_C Check Negative Difference Density on H10G      .      -0.38 eA-3
PLAT977_ALERT_2_C Check Negative Difference Density on H10H      .      -0.50 eA-3
PLAT977_ALERT_2_C Check Negative Difference Density on H10I      .      -0.38 eA-3
```

Alert level G

```
PLAT002_ALERT_2_G Number of Distance or Angle Restraints on AtSite          3 Note
PLAT003_ALERT_2_G Number of Uiso or Uij Restrained non-H Atoms ...          3 Report
PLAT083_ALERT_2_G SHELXL Second Parameter in WGHT  Unusually Large       8.04 Why ?
PLAT172_ALERT_4_G The CIF-Embedded .res File Contains DFIX Records          1 Report
PLAT178_ALERT_4_G The CIF-Embedded .res File Contains SIMU Records          1 Report
PLAT187_ALERT_4_G The CIF-Embedded .res File Contains RIGU Records          1 Report
PLAT232_ALERT_2_G Hirshfeld Test Diff (M-X)  U1       --Cl1      .       17.2 s.u.
PLAT794_ALERT_5_G Tentative Bond Valency for U1        (IV)      .       3.37 Info
PLAT860_ALERT_3_G Number of Least-Squares Restraints .............         26 Note
PLAT912_ALERT_4_G Missing # of FCF Reflections Above STh/L=  0.600        173 Note
PLAT933_ALERT_2_G Number of HKL-OMIT Records in Embedded .res File          2 Note
PLAT978_ALERT_2_G Number C-C Bonds with Positive Residual Density.          0 Info
```

```
  0 ALERT level A = Most likely a serious problem - resolve or explain
  2 ALERT level B = A potentially serious problem, consider carefully
 11 ALERT level C = Check. Ensure it is not caused by an omission or oversight
 12 ALERT level G = General information/check it is not something unexpected

  1 ALERT type 1 CIF construction/syntax error, inconsistent or missing data
 16 ALERT type 2 Indicator that the structure model may be wrong or deficient
  3 ALERT type 3 Indicator that the structure quality may be low
  4 ALERT type 4 Improvement, methodology, query or suggestion
  1 ALERT type 5 Informative message, check
```

Datablock: 3

```
Bond precision:   C-C = 0.0088 A          Wavelength=1.54184

Cell:            a=10.9797(3)       b=14.7369(3)      c=20.1781(4)
                 alpha=89.705(2)    beta=76.023(2)    gamma=79.473(2)
Temperature:     150 K
```

	Calculated	Reported
Volume	3112.36(13)	3112.36(13)
Space group	P -1	P -1
Hall group	-P 1	-P 1
Moiety formula	2(C60 H110 N4 Si3 Th), C5 H12	C60 H110 N4 Si3 Th, 0.5(C5 H12)
Sum formula	C125 H232 N8 Si6 Th2	C62.50 H116 N4 Si3 Th
Mr	2479.82	1239.90
Dx,g cm-3	1.323	1.323
Z	1	2
Mu (mm-1)	8.566	8.566
F000	1302.0	1302.0
F000’	1297.81	
h,k,lmax	13,18,25	13,18,25
Nref	13051	12435
Tmin,Tmax	0.447,0.568	0.691,0.920
Tmin’	0.290	

Correction method= # Reported T Limits: Tmin=0.691 Tmax=0.920
AbsCorr = GAUSSIAN

Data completeness= 0.953 Theta(max)= 76.335

R(reflections)= 0.0425(11553) wR2(reflections)= 0.1017(12435)

S = 1.072 Npar= 706

The following ALERTS were generated. Each ALERT has the format
test-name_ALERT_alert-type_alert-level.
Click on the hyperlinks for more details of the test.

Alert level C

```
PLAT042_ALERT_1_C Calc. and Reported MoietyFormula Strings  Differ     Please Check
PLAT342_ALERT_3_C Low Bond Precision on  C-C Bonds ..............    0.00882 Ang.
PLAT906_ALERT_3_C Large K Value in the Analysis of Variance ......     2.340 Check
PLAT911_ALERT_3_C Missing FCF Refl Between Thmin & STh/L=    0.600       208 Report
PLAT971_ALERT_2_C Check Calcd Resid. Dens.  1.00Ang From Th1            2.04 eA-3
PLAT971_ALERT_2_C Check Calcd Resid. Dens.  0.96Ang From Th1            2.00 eA-3
PLAT972_ALERT_2_C Check Calcd Resid. Dens.  0.69Ang From Th1           -1.79 eA-3
PLAT972_ALERT_2_C Check Calcd Resid. Dens.  1.02Ang From Th1           -1.69 eA-3
PLAT972_ALERT_2_C Check Calcd Resid. Dens.  1.30Ang From C6            -1.62 eA-3
```

Alert level G

```
PLAT002_ALERT_2_G Number of Distance or Angle Restraints on AtSite        16 Note
PLAT003_ALERT_2_G Number of Uiso or Uij Restrained non-H Atoms ...        16 Report
PLAT045_ALERT_1_G Calculated and Reported Z Differ by a Factor ...     0.500 Check
```

```
PLAT083_ALERT_2_G SHELXL Second Parameter in WGHT  Unusually Large       6.47 Why ?
PLAT154_ALERT_1_G The s.u.'s on the Cell Angles are Equal ..(Note)      0.002 Degree
PLAT172_ALERT_4_G The CIF-Embedded .res File Contains DFIX Records          1 Report
PLAT176_ALERT_4_G The CIF-Embedded .res File Contains SADI Records          1 Report
PLAT178_ALERT_4_G The CIF-Embedded .res File Contains SIMU Records          1 Report
PLAT187_ALERT_4_G The CIF-Embedded .res File Contains RIGU Records          1 Report
PLAT300_ALERT_4_G Atom Site Occupancy of C61        Constrained at        0.5 Check
PLAT300_ALERT_4_G Atom Site Occupancy of C62        Constrained at        0.5 Check
PLAT300_ALERT_4_G Atom Site Occupancy of C63        Constrained at        0.5 Check
PLAT300_ALERT_4_G Atom Site Occupancy of C64        Constrained at        0.5 Check
PLAT300_ALERT_4_G Atom Site Occupancy of C65        Constrained at        0.5 Check
PLAT300_ALERT_4_G Atom Site Occupancy of H61A       Constrained at        0.5 Check
PLAT300_ALERT_4_G Atom Site Occupancy of H61B       Constrained at        0.5 Check
PLAT300_ALERT_4_G Atom Site Occupancy of H61C       Constrained at        0.5 Check
PLAT300_ALERT_4_G Atom Site Occupancy of H62A       Constrained at        0.5 Check
PLAT300_ALERT_4_G Atom Site Occupancy of H62B       Constrained at        0.5 Check
PLAT300_ALERT_4_G Atom Site Occupancy of H63A       Constrained at        0.5 Check
PLAT300_ALERT_4_G Atom Site Occupancy of H63B       Constrained at        0.5 Check
PLAT300_ALERT_4_G Atom Site Occupancy of H64A       Constrained at        0.5 Check
PLAT300_ALERT_4_G Atom Site Occupancy of H64B       Constrained at        0.5 Check
PLAT300_ALERT_4_G Atom Site Occupancy of H65A       Constrained at        0.5 Check
PLAT300_ALERT_4_G Atom Site Occupancy of H65B       Constrained at        0.5 Check
PLAT300_ALERT_4_G Atom Site Occupancy of H65C       Constrained at        0.5 Check
PLAT301_ALERT_3_G Main Residue  Disorder .............(Resd  1  )         7% Note
PLAT302_ALERT_4_G Anion/Solvent/Minor-Residue Disorder (Resd  2  )       100% Note
PLAT304_ALERT_4_G Non-Integer Number of Atoms in ..... (Resd  2  )       8.50 Check
PLAT410_ALERT_2_G Short Intra H...H Contact  H18A     ..H40D     .       2.00 Ang.
                                                    x,y,z  =      1_555 Check
PLAT410_ALERT_2_G Short Intra H...H Contact  H24      ..H36C     .       1.70 Ang.
                                                    x,y,z  =      1_555 Check
PLAT411_ALERT_2_G Short Inter H...H Contact  H27B     ..H39C     .       1.91 Ang.
                                                 -1+x,y,z  =      1_455 Check
PLAT779_ALERT_4_G Suspect or Irrelevant (Bond) Angle(s) in CIF ...      34.44 Deg.
              N1   -SI1  -TH1     1_555   1_555   1_555 ......     #   22 Check
PLAT779_ALERT_4_G Suspect or Irrelevant (Bond) Angle(s) in CIF ...      33.76 Deg.
              N2   -SI2  -TH1     1_555   1_555   1_555 ......     #   32 Check
PLAT789_ALERT_4_G Atoms with Negative _atom_site_disorder_group  #         17 Check
PLAT793_ALERT_4_G Model has Chirality at C1          (Centro SPGR)          S Verify
PLAT793_ALERT_4_G Model has Chirality at C6          (Centro SPGR)          S Verify
PLAT802_ALERT_4_G CIF Input Record(s) with more than 80 Characters          2 Info
PLAT860_ALERT_3_G Number of Least-Squares Restraints ............         271 Note
PLAT912_ALERT_4_G Missing # of FCF Reflections Above STh/L=  0.600        409 Note
PLAT941_ALERT_3_G Average HKL Measurement Multiplicity ...........        3.3 Low
PLAT978_ALERT_2_G Number C-C Bonds with Positive Residual Density.          0 Info
```

---

   0 **ALERT level A** = Most likely a serious problem - resolve or explain
   0 **ALERT level B** = A potentially serious problem, consider carefully
   9 **ALERT level C** = Check. Ensure it is not caused by an omission or oversight
  42 **ALERT level G** = General information/check it is not something unexpected

```
   3 ALERT type 1 CIF construction/syntax error, inconsistent or missing data
  12 ALERT type 2 Indicator that the structure model may be wrong or deficient
   6 ALERT type 3 Indicator that the structure quality may be low
  30 ALERT type 4 Improvement, methodology, query or suggestion
   0 ALERT type 5 Informative message, check
```

Datablock: 4

Bond precision:	C-C = 0.0125 A		Wavelength=1.54184
Cell:	a=10.9402(5)	b=14.8642(8)	c=20.1789(7)
	alpha=89.645(3)	beta=76.097(4)	gamma=79.090(4)
Temperature:	150 K		

	Calculated	Reported
Volume	3125.1(3)	3125.1(3)
Space group	P -1	P -1
Hall group	-P 1	-P 1
Moiety formula	2(C60 H110 N4 Si3 U), C7 H8	C60 H110 N4 Si3 U, 0.5(C7 H8)
Sum formula	C127 H228 N8 Si6 U2	C63.50 H114 N4 Si3 U
Mr	2511.79	1255.88
Dx,g cm-3	1.335	1.335
Z	1	2
Mu (mm-1)	8.152	8.152
F000	1314.0	1314.0
F000'	1309.46	
h,k,lmax	13,18,25	13,18,25
Nref	13113	12523
Tmin,Tmax	0.529,0.722	0.784,0.942
Tmin'	0.465	

Correction method= # Reported T Limits: Tmin=0.784 Tmax=0.942
AbsCorr = GAUSSIAN

Data completeness= 0.955 Theta(max)= 76.354

R(reflections)= 0.0586(10672) wR2(reflections)= 0.1545(12523)

S = 1.044 Npar= 637

The following ALERTS were generated. Each ALERT has the format
test-name_ALERT_alert-type_alert-level.
Click on the hyperlinks for more details of the test.

Alert level A
PLAT972_ALERT_2_A Check Calcd Resid. Dens. 0.85Ang From U1 -4.50 eA-3

Author Response: Residual density is close to a heavy atom. It is present despite applying absorption corrections. It is not chemically significant

```
PLAT972_ALERT_2_A Check Calcd Resid. Dens.  0.90Ang From U1           -4.16 eA-3
```

Author Response: Residual density is close to a heavy atom. It is present despite applying absorption corrections. It is not chemically significant

```
PLAT972_ALERT_2_A Check Calcd Resid. Dens.  1.02Ang From U1           -3.76 eA-3
```

Author Response: Residual density is close to a heavy atom. It is present despite applying absorption corrections. It is not chemically significant

---

Alert level B

```
PLAT201_ALERT_2_B Isotropic non-H Atoms in Main Residue(s) .......          1 Report
              N3
```

Author Response: N3 was refined isotropically.

```
PLAT972_ALERT_2_B Check Calcd Resid. Dens.  0.80Ang From U1           -3.47 eA-3
```

Author Response: Residual density is close to a heavy atom. It is present despite applying absorption corrections. It is not chemically significant

```
PLAT972_ALERT_2_B Check Calcd Resid. Dens.  1.09Ang From U1           -3.23 eA-3
```

Author Response: Residual density is close to a heavy atom. It is present despite applying absorption corrections. It is not chemically significant

```
PLAT972_ALERT_2_B Check Calcd Resid. Dens.  0.90Ang From U1           -3.00 eA-3
```

Author Response: Residual density is close to a heavy atom. It is present despite applying absorption corrections. It is not chemically significant

---

Alert level C

```
PLAT042_ALERT_1_C Calc. and Reported MoietyFormula Strings  Differ     Please Check
PLAT342_ALERT_3_C Low Bond Precision on  C-C Bonds ...............    0.01247 Ang.
PLAT911_ALERT_3_C Missing FCF Refl Between Thmin & STh/L=    0.600         68 Report
PLAT971_ALERT_2_C Check Calcd Resid. Dens.  1.24Ang From U1             2.19 eA-3
PLAT971_ALERT_2_C Check Calcd Resid. Dens.  0.98Ang From U1             1.97 eA-3
PLAT971_ALERT_2_C Check Calcd Resid. Dens.  1.28Ang From U1             1.91 eA-3
PLAT971_ALERT_2_C Check Calcd Resid. Dens.  1.20Ang From U1             1.80 eA-3
```

```
PLAT971_ALERT_2_C Check Calcd Resid. Dens.  1.37Ang From U1            1.72 eA-3
PLAT971_ALERT_2_C Check Calcd Resid. Dens.  1.23Ang From U1            1.59 eA-3
PLAT975_ALERT_2_C Check Calcd Resid. Dens.  0.87Ang From N2      .     1.04 eA-3
PLAT975_ALERT_2_C Check Calcd Resid. Dens.  0.76Ang From N3      .     1.01 eA-3
PLAT977_ALERT_2_C Check Negative Difference Density on H19A      .    -0.36 eA-3
```

Alert level G

```
PLAT002_ALERT_2_G Number of Distance or Angle Restraints on AtSite          6 Note
PLAT045_ALERT_1_G Calculated and Reported Z Differ by a Factor ...      0.500 Check
PLAT172_ALERT_4_G The CIF-Embedded .res File Contains DFIX Records          1 Report
PLAT300_ALERT_4_G Atom Site Occupancy of C61       Constrained at         0.5 Check
PLAT300_ALERT_4_G Atom Site Occupancy of C62       Constrained at         0.5 Check
PLAT300_ALERT_4_G Atom Site Occupancy of C63       Constrained at         0.5 Check
PLAT300_ALERT_4_G Atom Site Occupancy of C64       Constrained at         0.5 Check
PLAT300_ALERT_4_G Atom Site Occupancy of C65       Constrained at         0.5 Check
PLAT300_ALERT_4_G Atom Site Occupancy of C66       Constrained at         0.5 Check
PLAT300_ALERT_4_G Atom Site Occupancy of C67       Constrained at         0.5 Check
PLAT300_ALERT_4_G Atom Site Occupancy of H61A      Constrained at         0.5 Check
PLAT300_ALERT_4_G Atom Site Occupancy of H61B      Constrained at         0.5 Check
PLAT300_ALERT_4_G Atom Site Occupancy of H61C      Constrained at         0.5 Check
PLAT300_ALERT_4_G Atom Site Occupancy of H63       Constrained at         0.5 Check
PLAT300_ALERT_4_G Atom Site Occupancy of H64       Constrained at         0.5 Check
PLAT300_ALERT_4_G Atom Site Occupancy of H65       Constrained at         0.5 Check
PLAT300_ALERT_4_G Atom Site Occupancy of H66       Constrained at         0.5 Check
PLAT300_ALERT_4_G Atom Site Occupancy of H67       Constrained at         0.5 Check
PLAT302_ALERT_4_G Anion/Solvent/Minor-Residue Disorder (Resd  2  )       100% Note
PLAT304_ALERT_4_G Non-Integer Number of Atoms in ..... (Resd  2  )       7.50 Check
PLAT789_ALERT_4_G Atoms with Negative _atom_site_disorder_group  #         15 Check
PLAT793_ALERT_4_G Model has Chirality at C1          (Centro SPGR)          R Verify
PLAT793_ALERT_4_G Model has Chirality at C6          (Centro SPGR)          R Verify
PLAT802_ALERT_4_G CIF Input Record(s) with more than 80 Characters          2 Info
PLAT860_ALERT_3_G Number of Least-Squares Restraints ............           6 Note
PLAT912_ALERT_4_G Missing # of FCF Reflections Above STh/L=  0.600        523 Note
PLAT933_ALERT_2_G Number of HKL-OMIT Records in Embedded .res File          1 Note
PLAT941_ALERT_3_G Average HKL Measurement Multiplicity ...........        2.8 Low
PLAT978_ALERT_2_G Number C-C Bonds with Positive Residual Density.          0 Info
```

---

   3 **ALERT level A** = Most likely a serious problem - resolve or explain
   4 **ALERT level B** = A potentially serious problem, consider carefully
  12 **ALERT level C** = Check. Ensure it is not caused by an omission or oversight
  29 **ALERT level G** = General information/check it is not something unexpected

```
   2 ALERT type 1 CIF construction/syntax error, inconsistent or missing data
  19 ALERT type 2 Indicator that the structure model may be wrong or deficient
   4 ALERT type 3 Indicator that the structure quality may be low
  23 ALERT type 4 Improvement, methodology, query or suggestion
   0 ALERT type 5 Informative message, check
```

Datablock: 5

```
Bond precision:   C-C = 0.0059 A          Wavelength=1.54184
```

Cell:	a=15.2420(1)	b=23.7285(2)	c=26.1857(2)
	alpha=90	beta=98.970(1)	gamma=90
Temperature:	100 K		

	Calculated	Reported
Volume	9354.75(12)	9354.75(13)
Space group	P 21/c	P 1 21/c 1
Hall group	-P 2ybc	-P 2ybc
Moiety formula	C60 H112 N4 P Si3 Th, C18 H36 N2 Na O6, 2(C4 H10 O)	C60 H112 N4 P Si3 Th, C18 H36 N2 Na O6, 2(C4 H10 O)
Sum formula	C86 H168 N6 Na O8 P Si3 Th	C86 H168 N6 Na O8 P Si3 Th
Mr	1784.54	1784.52
Dx,g cm-3	1.267	1.267
Z	4	4
Mu (mm-1)	6.125	6.125
F000	3792.0	3792.0
F000'	3788.57	
h,k,lmax	19,29,33	18,29,32
Nref	19754	19050
Tmin,Tmax	0.121,0.378	0.377,1.000
Tmin'	0.048	

Correction method= # Reported T Limits: Tmin=0.377 Tmax=1.000
AbsCorr = GAUSSIAN

Data completeness= 0.964 Theta(max)= 76.879

R(reflections)= 0.0382(18131) wR2(reflections)= 0.1054(19050)

S = 1.079 Npar= 960

The following ALERTS were generated. Each ALERT has the format
test-name_ALERT_alert-type_alert-level.
Click on the hyperlinks for more details of the test.

Alert level A

PLAT972_ALERT_2_A Check Calcd Resid. Dens. 0.83Ang From Th1 -3.53 eA-3

Author Response: Residual density is close to a heavy atom, and is present despite applying the correct absorption correction. It is not chemically significant.

Alert level B

```
PLAT972_ALERT_2_B Check Calcd Resid. Dens.  0.81Ang From Th1           -2.61 eA-3
```

Author Response: Residual density is close to a heavy atom, and is present despite applying the correct absorption correction. It is not chemically significant.

Alert level C

```
PLAT202_ALERT_3_C Isotropic non-H Atoms in Anion/Solvent .........          5 Check
              O8      C83     C84     C85     C86
PLAT241_ALERT_2_C High   'MainMol' Ueq as Compared to Neighbors of        C16 Check
PLAT242_ALERT_2_C Low    'MainMol' Ueq as Compared to Neighbors of        C15 Check
PLAT411_ALERT_2_C Short Inter H...H Contact  H17B     ..H48A     .       2.06 Ang.
                                          -1+x,y,z  =      1_455 Check
PLAT911_ALERT_3_C Missing FCF Refl Between Thmin & STh/L=    0.600        119 Report
PLAT971_ALERT_2_C Check Calcd Resid. Dens.  0.86Ang From Th1             2.39 eA-3
PLAT971_ALERT_2_C Check Calcd Resid. Dens.  0.84Ang From Th1             2.36 eA-3
PLAT971_ALERT_2_C Check Calcd Resid. Dens.  0.93Ang From Th1             2.12 eA-3
PLAT971_ALERT_2_C Check Calcd Resid. Dens.  0.87Ang From Th1             2.02 eA-3
PLAT971_ALERT_2_C Check Calcd Resid. Dens.  1.28Ang From Th1             1.67 eA-3
PLAT972_ALERT_2_C Check Calcd Resid. Dens.  0.74Ang From Th1            -1.71 eA-3
```

Author Response: Residual density is close to a heavy atom, and is present despite applying the correct absorption correction. It is not chemically significant.

```
PLAT972_ALERT_2_C Check Calcd Resid. Dens.  0.90Ang From Th1            -1.51 eA-3
```

Author Response: Residual density is close to a heavy atom, and is present despite applying the correct absorption correction. It is not chemically significant.

```
PLAT975_ALERT_2_C Check Calcd Resid. Dens.  1.04Ang From O7      .       0.61 eA-3
PLAT975_ALERT_2_C Check Calcd Resid. Dens.  0.92Ang From N2      .       0.53 eA-3
PLAT977_ALERT_2_C Check Negative Difference Density on H14B      .      -0.32 eA-3
PLAT977_ALERT_2_C Check Negative Difference Density on H17A      .      -0.31 eA-3
```

Alert level G

```
PLAT002_ALERT_2_G Number of Distance or Angle Restraints on AtSite         18 Note
PLAT003_ALERT_2_G Number of Uiso or Uij Restrained non-H Atoms ...         10 Report
PLAT083_ALERT_2_G SHELXL Second Parameter in WGHT  Unusually Large      14.97 Why ?
PLAT142_ALERT_4_G s.u. on b - Axis Small or Missing .............     0.00020 Ang.
PLAT143_ALERT_4_G s.u. on c - Axis Small or Missing .............     0.00020 Ang.
PLAT172_ALERT_4_G The CIF-Embedded .res File Contains DFIX Records          4 Report
PLAT176_ALERT_4_G The CIF-Embedded .res File Contains SADI Records          2 Report
PLAT178_ALERT_4_G The CIF-Embedded .res File Contains SIMU Records          1 Report
```

```
PLAT187_ALERT_4_G The CIF-Embedded .res File Contains RIGU Records          1 Report
PLAT302_ALERT_4_G Anion/Solvent/Minor-Residue Disorder (Resd  4  )       100% Note
PLAT302_ALERT_4_G Anion/Solvent/Minor-Residue Disorder (Resd  5  )       100% Note
PLAT303_ALERT_2_G Full Occupancy Atom H1        with # Connections       2.00 Check
PLAT304_ALERT_4_G Non-Integer Number of Atoms in ..... (Resd  4  )      11.20 Check
PLAT304_ALERT_4_G Non-Integer Number of Atoms in ..... (Resd  5  )       3.80 Check
PLAT398_ALERT_2_G Deviating  C-O-C   Angle From 120 for O9       .      109.2 Degree
PLAT411_ALERT_2_G Short Inter H...H Contact  H13B     ..H1BB     .       2.05 Ang.
                                         -1+x,1/2-y,-1/2+z  =      4_465 Check
PLAT411_ALERT_2_G Short Inter H...H Contact  H33A     ..H1BA     .       1.92 Ang.
                                            x,1/2-y,-1/2+z  =      4_565 Check
PLAT413_ALERT_2_G Short Inter XH3 .. XHn     H62A     ..H1DB     .       2.03 Ang.
                                               1-x,1-y,2-z  =      3_667 Check
PLAT720_ALERT_4_G Number of Unusual/Non-Standard Labels .........         10 Note
PLAT794_ALERT_5_G Tentative Bond Valency for Th1       (IV)      .       4.43 Info
PLAT802_ALERT_4_G CIF Input Record(s) with more than 80 Characters          3 Info
PLAT860_ALERT_3_G Number of Least-Squares Restraints ............         40 Note
PLAT912_ALERT_4_G Missing # of FCF Reflections Above STh/L=  0.600        540 Note
PLAT941_ALERT_3_G Average HKL Measurement Multiplicity ..........         3.0 Low
PLAT978_ALERT_2_G Number C-C Bonds with Positive Residual Density.          1 Info
```

---

1 **ALERT level A** = Most likely a serious problem - resolve or explain
1 **ALERT level B** = A potentially serious problem, consider carefully
16 **ALERT level C** = Check. Ensure it is not caused by an omission or oversight
25 **ALERT level G** = General information/check it is not something unexpected

0 ALERT type 1 CIF construction/syntax error, inconsistent or missing data
25 ALERT type 2 Indicator that the structure model may be wrong or deficient
4 ALERT type 3 Indicator that the structure quality may be low
13 ALERT type 4 Improvement, methodology, query or suggestion
1 ALERT type 5 Informative message, check

Datablock: 6

Bond precision:	C-C = 0.0138 A	Wavelength=0.71073	
Cell:	a=15.5451(4)	b=15.8372(4)	c=20.8962(7)
	alpha=84.767(2)	beta=79.885(2)	gamma=80.383(2)
Temperature:	150 K		

	Calculated	Reported
Volume	4983.2(2)	4983.2(2)
Space group	P -1	P -1
Hall group	-P 1	-P 1
Moiety formula	2(C60 H112 As N4 Si3 Th), 2(C18 H36 K N2 O6), 5(C4 H8 O)	C60 H112 As N4 Si3 Th, C18 H36 K N2 O6, 2.5(C4 H8 O)
Sum formula	C176 H336 As2 K2 N12 O17 Si6 Th2	C88 H168 As K N6 O8.50 Si3 Th
Mr	3753.24	1876.60
Dx,g cm-3	1.251	1.251
Z	1	2
Mu (mm-1)	1.955	1.955
F000	1980.0	1980.0
F000'	1967.14	
h,k,lmax	18,19,25	18,19,25
Nref	18943	18893
Tmin,Tmax	0.594,0.739	0.567,1.000
Tmin'	0.541	

Correction method= # Reported T Limits: Tmin=0.567 Tmax=1.000
AbsCorr = GAUSSIAN

Data completeness= 0.997 Theta(max)= 25.681

R(reflections)= 0.0838(14428) wR2(reflections)= 0.2113(18893)

S = 1.027 Npar= 928

The following ALERTS were generated. Each ALERT has the format
test-name_ALERT_alert-type_alert-level.
Click on the hyperlinks for more details of the test.

Alert level A

PLAT971_ALERT_2_A Check Calcd Resid. Dens. 1.24Ang From Th1 4.35 eA-3

Author Response: Residual density is close to a heavy atom, and is present despite applying the correct absorption correction. It is not chemically significant.

PLAT971_ALERT_2_A Check Calcd Resid. Dens. 1.08Ang From Th1 4.25 eA-3

Author Response: Residual density is close to a heavy atom, and is present despite applying the correct absorption correction. It is not chemically significant.

```
PLAT971_ALERT_2_A Check Calcd Resid. Dens.  0.91Ang From Th1            3.73 eA-3
```

Author Response: Residual density is close to a heavy atom, and is present despite applying the correct absorption correction. It is not chemically significant.

```
PLAT972_ALERT_2_A Check Calcd Resid. Dens.  1.05Ang From Th1           -4.41 eA-3
```

Author Response: Residual density is close to a heavy atom, and is present despite applying the correct absorption correction. It is not chemically significant.

```
PLAT972_ALERT_2_A Check Calcd Resid. Dens.  0.84Ang From Th1           -4.04 eA-3
```

Author Response: Residual density is close to a heavy atom, and is present despite applying the correct absorption correction. It is not chemically significant.

```
PLAT973_ALERT_2_A Check Calcd Positive Resid. Density on       Th1       4.23 eA-3
```

Author Response: Residual density is close to a heavy atom, and is present despite applying the correct absorption correction. It is not chemically significant.

Alert level B

```
PLAT910_ALERT_3_B Missing # of FCF Reflection(s) Below Theta(Min).        48 Note
```

Author Response: Missing reflections at low angle are most likely obscured by the beam stop.

```
PLAT971_ALERT_2_B Check Calcd Resid. Dens.  0.77Ang From Th1            3.49 eA-3
```

Author Response: Residual density is close to a heavy atom, and is present despite applying the correct absorption correction. It is not chemically significant.

```
PLAT971_ALERT_2_B Check Calcd Resid. Dens.  1.16Ang From Th1            3.03 eA-3
```

Author Response: Residual density is close to a heavy atom, and is present despite applying the correct absorption correction. It is not chemically significant.

```
PLAT971_ALERT_2_B Check Calcd Resid. Dens.  1.13Ang From Th1            2.65 eA-3
```

Author Response: Residual density is close to a heavy atom, and is present despite applying the correct absorption correction. It is not chemically significant.

```
PLAT972_ALERT_2_B Check Calcd Resid. Dens.  0.92Ang From Th1           -2.69 eA-3
```

Author Response: Residual density is close to a heavy atom, and is present despite applying the correct absorption correction. It is not chemically significant.

Alert level C

```
RINTA01_ALERT_3_C  The value of Rint is greater than 0.12
            Rint given   0.147
PLAT020_ALERT_3_C The Value of Rint is Greater Than 0.12 .........      0.147 Report
PLAT042_ALERT_1_C Calc. and Reported MoietyFormula Strings  Differ     Please Check
PLAT202_ALERT_3_C Isotropic non-H Atoms in Anion/Solvent .........         10 Check
              O7      C79     C80     C81     C82     O8                 etc.
PLAT213_ALERT_2_C Atom C44             has ADP max/min Ratio .....        3.5 prolat
PLAT220_ALERT_2_C NonSolvent   Resd 1  C   Ueq(max)/Ueq(min) Range        3.3 Ratio
PLAT230_ALERT_2_C Hirshfeld Test Diff for    C69      --C70      .        6.0 s.u.
PLAT234_ALERT_4_C Large Hirshfeld Difference O2       --C71      .       0.21 Ang.
PLAT234_ALERT_4_C Large Hirshfeld Difference C63      --C64      .       0.18 Ang.
PLAT241_ALERT_2_C High   'MainMol' Ueq as Compared to Neighbors of        C44 Check
PLAT241_ALERT_2_C High   'MainMol' Ueq as Compared to Neighbors of        C57 Check
PLAT241_ALERT_2_C High   'MainMol' Ueq as Compared to Neighbors of        C65 Check
PLAT241_ALERT_2_C High   'MainMol' Ueq as Compared to Neighbors of        C70 Check
PLAT241_ALERT_2_C High   'MainMol' Ueq as Compared to Neighbors of        C72 Check
PLAT241_ALERT_2_C High   'MainMol' Ueq as Compared to Neighbors of        C78 Check
PLAT242_ALERT_2_C Low    'MainMol' Ueq as Compared to Neighbors of         K1 Check
PLAT242_ALERT_2_C Low    'MainMol' Ueq as Compared to Neighbors of         O4 Check
PLAT242_ALERT_2_C Low    'MainMol' Ueq as Compared to Neighbors of         N6 Check
PLAT243_ALERT_4_C High   'Solvent' Ueq as Compared to Neighbors of         O8 Check
PLAT244_ALERT_4_C Low    'Solvent' Ueq as Compared to Neighbors of        C86 Check
PLAT342_ALERT_3_C Low Bond Precision on  C-C Bonds ..............    0.01382 Ang.
PLAT410_ALERT_2_C Short Intra H...H Contact  H1       ..H29      .       1.99 Ang.
                                                     x,y,z  =      1_555 Check
PLAT911_ALERT_3_C Missing FCF Refl Between Thmin & STh/L=    0.600          4 Report
PLAT971_ALERT_2_C Check Calcd Resid. Dens.  1.40Ang From N1             2.26 eA-3
```

Author Response: Residual density is close to a heavy atom, and is present despite applying the correct absorption correction. It is not chemically significant.

PLAT971_ALERT_2_C Check Calcd Resid. Dens. 1.04Ang From As1 2.19 eA-3

Author Response: Residual density is close to a heavy atom, and is present despite applying the correct absorption correction. It is not chemically significant.

PLAT971_ALERT_2_C Check Calcd Resid. Dens. 1.27Ang From Th1 2.04 eA-3

Author Response: Residual density is close to a heavy atom, and is present despite applying the correct absorption correction. It is not chemically significant.

PLAT971_ALERT_2_C Check Calcd Resid. Dens. 1.31Ang From Th1 1.86 eA-3

Author Response: Residual density is close to a heavy atom, and is present despite applying the correct absorption correction. It is not chemically significant.

PLAT971_ALERT_2_C Check Calcd Resid. Dens. 1.17Ang From N1 1.77 eA-3

Author Response: Residual density is close to a heavy atom, and is present despite applying the correct absorption correction. It is not chemically significant.

PLAT971_ALERT_2_C Check Calcd Resid. Dens. 0.99Ang From N4 1.76 eA-3

Author Response: Residual density is close to a heavy atom, and is present despite applying the correct absorption correction. It is not chemically significant.

PLAT971_ALERT_2_C Check Calcd Resid. Dens. 1.02Ang From As1 1.75 eA-3

Author Response: Residual density is close to a heavy atom, and is present despite applying the correct absorption correction. It is not chemically significant.

PLAT971_ALERT_2_C Check Calcd Resid. Dens. 1.80Ang From Th1 1.72 eA-3

Author Response: Residual density is close to a heavy atom, and is present despite applying the correct absorption correction. It is not chemically significant.

PLAT971_ALERT_2_C Check Calcd Resid. Dens. 1.32Ang From Th1 1.71 eA-3

Author Response: Residual density is close to a heavy atom, and is present despite applying the correct absorption correction. It is not chemically significant.

PLAT971_ALERT_2_C Check Calcd Resid. Dens. 1.61Ang From N2 1.57 eA-3

Author Response: Residual density is close to a heavy atom, and is present despite applying the correct absorption correction. It is not chemically significant.

PLAT971_ALERT_2_C Check Calcd Resid. Dens. 0.95Ang From As1 1.54 eA-3

Author Response: Residual density is close to a heavy atom, and is present despite applying the correct absorption correction. It is not chemically significant.

PLAT972_ALERT_2_C Check Calcd Resid. Dens. 0.87Ang From Th1 -2.47 eA-3

Author Response: Residual density is close to a heavy atom, and is present despite applying the correct absorption correction. It is not chemically significant.

PLAT972_ALERT_2_C Check Calcd Resid. Dens. 1.15Ang From Th1 -2.32 eA-3

Author Response: Residual density is close to a heavy atom, and is present despite applying the correct absorption correction. It is not chemically significant.

PLAT972_ALERT_2_C Check Calcd Resid. Dens. 1.32Ang From Th1 -2.29 eA-3

Author Response: Residual density is close to a heavy atom, and is present despite applying the correct absorption correction. It is not chemically significant.

PLAT972_ALERT_2_C Check Calcd Resid. Dens. 1.01Ang From Th1 -2.23 eA-3

Author Response: Residual density is close to a heavy atom, and is present despite applying the correct absorption correction. It is not chemically significant.

```
PLAT972_ALERT_2_C Check Calcd Resid. Dens.  1.38Ang From Th1           -2.17 eA-3
```

Author Response: Residual density is close to a heavy atom, and is present despite applying the correct absorption correction. It is not chemically significant.

```
PLAT972_ALERT_2_C Check Calcd Resid. Dens.  1.61Ang From Th1           -2.11 eA-3
```

Author Response: Residual density is close to a heavy atom, and is present despite applying the correct absorption correction. It is not chemically significant.

```
PLAT972_ALERT_2_C Check Calcd Resid. Dens.  1.53Ang From As1           -1.87 eA-3
```

Author Response: Residual density is close to a heavy atom, and is present despite applying the correct absorption correction. It is not chemically significant.

```
PLAT972_ALERT_2_C Check Calcd Resid. Dens.  1.35Ang From Th1           -1.84 eA-3
```

Author Response: Residual density is close to a heavy atom, and is present despite applying the correct absorption correction. It is not chemically significant.

```
PLAT972_ALERT_2_C Check Calcd Resid. Dens.  1.03Ang From N2            -1.70 eA-3
```

Author Response: Residual density is close to a heavy atom, and is present despite applying the correct absorption correction. It is not chemically significant.

```
PLAT972_ALERT_2_C Check Calcd Resid. Dens.  1.64Ang From Th1           -1.68 eA-3
```

Author Response: Residual density is close to a heavy atom, and is present despite applying the correct absorption correction. It is not chemically significant.

```
PLAT972_ALERT_2_C Check Calcd Resid. Dens.  1.12Ang From As1           -1.53 eA-3
```

Author Response: Residual density is close to a heavy atom, and is present despite applying the correct absorption correction. It is not chemically significant.

```
PLAT977_ALERT_2_C Check Negative Difference Density on H1         .      -1.05 eA-3
PLAT977_ALERT_2_C Check Negative Difference Density on H7A        .      -0.31 eA-3
PLAT977_ALERT_2_C Check Negative Difference Density on H19B       .      -0.37 eA-3
PLAT977_ALERT_2_C Check Negative Difference Density on H88A       .      -0.38 eA-3
PLAT977_ALERT_2_C Check Negative Difference Density on H90A       .      -0.34 eA-3
```

Alert level G

```
PLAT002_ALERT_2_G Number of Distance or Angle Restraints on AtSite         18 Note
PLAT003_ALERT_2_G Number of Uiso or Uij Restrained non-H Atoms ...         15 Report
PLAT045_ALERT_1_G Calculated and Reported Z Differ by a Factor ...      0.500 Check
PLAT154_ALERT_1_G The s.u.'s on the Cell Angles are Equal ..(Note)      0.002 Degree
PLAT172_ALERT_4_G The CIF-Embedded .res File Contains DFIX Records          4 Report
PLAT176_ALERT_4_G The CIF-Embedded .res File Contains SADI Records          2 Report
PLAT178_ALERT_4_G The CIF-Embedded .res File Contains SIMU Records          1 Report
PLAT187_ALERT_4_G The CIF-Embedded .res File Contains RIGU Records          1 Report
PLAT300_ALERT_4_G Atom Site Occupancy of O12        Constrained at        0.5 Check
PLAT300_ALERT_4_G Atom Site Occupancy of C87        Constrained at        0.5 Check
PLAT300_ALERT_4_G Atom Site Occupancy of C88        Constrained at        0.5 Check
PLAT300_ALERT_4_G Atom Site Occupancy of C89        Constrained at        0.5 Check
PLAT300_ALERT_4_G Atom Site Occupancy of C90        Constrained at        0.5 Check
PLAT300_ALERT_4_G Atom Site Occupancy of H87A       Constrained at        0.5 Check
PLAT300_ALERT_4_G Atom Site Occupancy of H87B       Constrained at        0.5 Check
PLAT300_ALERT_4_G Atom Site Occupancy of H88A       Constrained at        0.5 Check
PLAT300_ALERT_4_G Atom Site Occupancy of H88B       Constrained at        0.5 Check
PLAT300_ALERT_4_G Atom Site Occupancy of H89A       Constrained at        0.5 Check
PLAT300_ALERT_4_G Atom Site Occupancy of H89B       Constrained at        0.5 Check
PLAT300_ALERT_4_G Atom Site Occupancy of H90A       Constrained at        0.5 Check
PLAT300_ALERT_4_G Atom Site Occupancy of H90B       Constrained at        0.5 Check
PLAT302_ALERT_4_G Anion/Solvent/Minor-Residue Disorder (Resd  5  )       100% Note
PLAT303_ALERT_2_G Full Occupancy Atom H1        with # Connections       2.00 Check
PLAT304_ALERT_4_G Non-Integer Number of Atoms in ..... (Resd  5  )       6.50 Check
PLAT398_ALERT_2_G Deviating  C-O-C   Angle From 120 for O8       .      107.4 Degree
PLAT398_ALERT_2_G Deviating  C-O-C   Angle From 120 for O12      .       94.8 Degree
PLAT411_ALERT_2_G Short Inter H...H Contact  H57A     ..H88A     .       2.08 Ang.
                                                   -x,-y,-z  =      2_555 Check
PLAT767_ALERT_4_G INS Embedded LIST 6 Instruction Should be LIST 4     Please Check
PLAT790_ALERT_4_G Centre of Gravity not Within Unit Cell: Resd.  #          2 Note
              C18 H36 K N2 O6
PLAT790_ALERT_4_G Centre of Gravity not Within Unit Cell: Resd.  #          3 Note
              C4 H8 O
PLAT794_ALERT_5_G Tentative Bond Valency for Th1       (IV)      .       4.35 Info
PLAT802_ALERT_4_G CIF Input Record(s) with more than 80 Characters          5 Info
PLAT860_ALERT_3_G Number of Least-Squares Restraints ............          69 Note
PLAT933_ALERT_2_G Number of HKL-OMIT Records in Embedded .res File          2 Note
PLAT941_ALERT_3_G Average HKL Measurement Multiplicity ..........         2.5 Low
PLAT978_ALERT_2_G Number C-C Bonds with Positive Residual Density.          0 Info
```

---

6 **ALERT level A** = Most likely a serious problem - resolve or explain
5 **ALERT level B** = A potentially serious problem, consider carefully
50 **ALERT level C** = Check. Ensure it is not caused by an omission or oversight
36 **ALERT level G** = General information/check it is not something unexpected

3 ALERT type 1 CIF construction/syntax error, inconsistent or missing data

58 ALERT type 2 Indicator that the structure model may be wrong or deficient
8 ALERT type 3 Indicator that the structure quality may be low
27 ALERT type 4 Improvement, methodology, query or suggestion
1 ALERT type 5 Informative message, check

Datablock: 7

Bond precision: C-C = 0.0080 A Wavelength=1.54184

Cell:	a=15.2873(3)	b=23.5758(4)	c=25.9927(4)
	alpha=90	beta=98.536(2)	gamma=90
Temperature:	100 K		

	Calculated	Reported
Volume	9264.3(3)	9264.3(3)
Space group	P 21/c	P 1 21/c 1
Hall group	-P 2ybc	-P 2ybc
Moiety formula	C60 H112 N4 P Si3 U, C18 H36 N2 Na O6, 2(C4 H10 O)	C60 H112 N4 P Si3 U, C18 H36 N2 Na O6, 2(C4 H10 O)
Sum formula	C86 H168 N6 Na O8 P Si3 U	C86 H168 N6 Na O8 P Si3 U
Mr	1790.53	1790.51
Dx,g cm-3	1.284	1.284
Z	4	4
Mu (mm-1)	5.926	5.926
F000	3800.0	3800.0
F000'	3795.77	
h,k,lmax	19,29,32	19,29,32
Nref	19641	18293
Tmin,Tmax	0.428,0.556	0.427,1.000
Tmin'	0.274	

Correction method= # Reported T Limits: Tmin=0.427 Tmax=1.000
AbsCorr = GAUSSIAN

Data completeness= 0.931 Theta(max)= 77.158

R(reflections)= 0.0427(15269) wR2(reflections)= 0.1187(18293)

S = 1.073 Npar= 954

The following ALERTS were generated. Each ALERT has the format
test-name_ALERT_alert-type_alert-level.
Click on the hyperlinks for more details of the test.

Alert level C

```
PLAT202_ALERT_3_C Isotropic non-H Atoms in Anion/Solvent .........         10 Check
             O7      C79     C80     C81     C82     O8                  etc.
PLAT220_ALERT_2_C NonSolvent   Resd 1  C   Ueq(max)/Ueq(min) Range        3.3 Ratio
PLAT241_ALERT_2_C High   'MainMol' Ueq as Compared to Neighbors of         C6 Check
PLAT241_ALERT_2_C High   'MainMol' Ueq as Compared to Neighbors of        C61 Check
PLAT241_ALERT_2_C High   'MainMol' Ueq as Compared to Neighbors of        C63 Check
PLAT241_ALERT_2_C High   'MainMol' Ueq as Compared to Neighbors of        C71 Check
PLAT244_ALERT_4_C Low    'Solvent' Ueq as Compared to Neighbors of        C80 Check
PLAT342_ALERT_3_C Low Bond Precision on  C-C Bonds ...............    0.00804 Ang.
PLAT411_ALERT_2_C Short Inter H...H Contact  H27B      ..H51B     .      2.09 Ang.
                                                  1+x,y,z  =      1_655 Check
PLAT911_ALERT_3_C Missing FCF Refl Between Thmin & STh/L=    0.600        327 Report
PLAT972_ALERT_2_C Check Calcd Resid. Dens.  0.79Ang From U1             -2.33 eA-3
PLAT972_ALERT_2_C Check Calcd Resid. Dens.  0.76Ang From U1             -2.19 eA-3
PLAT972_ALERT_2_C Check Calcd Resid. Dens.  0.77Ang From U1             -2.05 eA-3
PLAT972_ALERT_2_C Check Calcd Resid. Dens.  0.79Ang From U1             -2.02 eA-3
PLAT972_ALERT_2_C Check Calcd Resid. Dens.  0.89Ang From U1             -1.90 eA-3
PLAT972_ALERT_2_C Check Calcd Resid. Dens.  0.81Ang From U1             -1.70 eA-3
PLAT972_ALERT_2_C Check Calcd Resid. Dens.  0.67Ang From U1             -1.56 eA-3
PLAT975_ALERT_2_C Check Calcd Resid. Dens.  0.99Ang From O7       .      1.27 eA-3
PLAT977_ALERT_2_C Check Negative Difference Density on H83C       .     -0.42 eA-3
```

Alert level G

```
PLAT002_ALERT_2_G Number of Distance or Angle Restraints on AtSite         28 Note
PLAT003_ALERT_2_G Number of Uiso or Uij Restrained non-H Atoms ...         15 Report
PLAT083_ALERT_2_G SHELXL Second Parameter in WGHT  Unusually Large       6.29 Why ?
PLAT172_ALERT_4_G The CIF-Embedded .res File Contains DFIX Records          4 Report
PLAT176_ALERT_4_G The CIF-Embedded .res File Contains SADI Records          1 Report
PLAT178_ALERT_4_G The CIF-Embedded .res File Contains SIMU Records          1 Report
PLAT187_ALERT_4_G The CIF-Embedded .res File Contains RIGU Records          1 Report
PLAT302_ALERT_4_G Anion/Solvent/Minor-Residue Disorder (Resd  2  )         7% Note
PLAT302_ALERT_4_G Anion/Solvent/Minor-Residue Disorder (Resd  4  )       100% Note
PLAT302_ALERT_4_G Anion/Solvent/Minor-Residue Disorder (Resd  5  )       100% Note
PLAT303_ALERT_2_G Full Occupancy Atom H1        with # Connections       2.00 Check
PLAT304_ALERT_4_G Non-Integer Number of Atoms in ..... (Resd  4  )      10.17 Check
PLAT304_ALERT_4_G Non-Integer Number of Atoms in ..... (Resd  5  )       4.83 Check
PLAT398_ALERT_2_G Deviating  C-O-C   Angle From 120 for O7        .     105.8 Degree
PLAT411_ALERT_2_G Short Inter H...H Contact  H13A      ..H1BA     .      2.09 Ang.
                                      x,3/2-y,-1/2+z  =      4_575 Check
PLAT413_ALERT_2_G Short Inter XH3 .. XHn     H6B       ..H1DB     .      2.08 Ang.
                                    1-x,-1/2+y,3/2-z  =      2_646 Check
PLAT413_ALERT_2_G Short Inter XH3 .. XHn     H82B      ..H1CB     .      2.14 Ang.
                                             x,y,z  =      1_555 Check
PLAT413_ALERT_2_G Short Inter XH3 .. XHn     H82B      ..H1DB     .      2.10 Ang.
                                             x,y,z  =      1_555 Check
PLAT432_ALERT_2_G Short Inter X...Y Contact  O7        ..C1D      .      2.91 Ang.
                                             x,y,z  =      1_555 Check
PLAT720_ALERT_4_G Number of Unusual/Non-Standard Labels ..........         10 Note
PLAT802_ALERT_4_G CIF Input Record(s) with more than 80 Characters          6 Info
PLAT860_ALERT_3_G Number of Least-Squares Restraints .............         61 Note
PLAT883_ALERT_1_G No Info/Value for _atom_sites_solution_primary .     Please Do !
PLAT912_ALERT_4_G Missing # of FCF Reflections Above STh/L=  0.600       1022 Note
PLAT933_ALERT_2_G Number of HKL-OMIT Records in Embedded .res File          7 Note
PLAT941_ALERT_3_G Average HKL Measurement Multiplicity ..........         2.8 Low
```

```
PLAT978_ALERT_2_G Number C-C Bonds with Positive Residual Density.         0 Info
```

```
   0 ALERT level A = Most likely a serious problem - resolve or explain
   0 ALERT level B = A potentially serious problem, consider carefully
  19 ALERT level C = Check. Ensure it is not caused by an omission or oversight
  27 ALERT level G = General information/check it is not something unexpected

   1 ALERT type 1 CIF construction/syntax error, inconsistent or missing data
  27 ALERT type 2 Indicator that the structure model may be wrong or deficient
   5 ALERT type 3 Indicator that the structure quality may be low
  13 ALERT type 4 Improvement, methodology, query or suggestion
   0 ALERT type 5 Informative message, check
```

Datablock: 8

Bond precision: C-C = 0.0093 A   Wavelength=1.54184

Cell:	a=13.5124(2)	b=16.2513(3)	c=22.1264(3)
	alpha=94.338(1)	beta=95.265(1)	gamma=102.228(1)
Temperature:	150 K		

	Calculated	Reported
Volume	4705.94(13)	4705.94(13)
Space group	P -1	P -1
Hall group	-P 1	-P 1
Moiety formula	2(C60 H112 As N4 Si3 U), 2(C18 H36 K N2 O6), C7 H8, 2(C4 H10 O)	C60 H112 As N4 Si3 U, C18 H36 K N2 O6, 0.5(C7 H8), C4 H10 O
Sum formula	C171 H324 As2 K2 N12 O14 Si6 U2	C85.50 H162 As K N6 O7 Si3 U
Mr	3645.08	1822.52
Dx,g cm-3	1.286	1.286
Z	1	2
Mu (mm-1)	6.399	6.399
F000	1918.0	1918.0
F000'	1913.85	
h,k,lmax	16,19,26	16,19,26
Nref	17254	16873
Tmin,Tmax	0.267,0.371	0.335,0.797
Tmin'	0.126	

Correction method= # Reported T Limits: Tmin=0.335 Tmax=0.797
AbsCorr = GAUSSIAN

Data completeness= 0.978 Theta(max)= 68.245

R(reflections)= 0.0529(15806) wR2(reflections)= 0.1482(16873)

S = 1.036 Npar= 1182

The following ALERTS were generated. Each ALERT has the format
test-name_ALERT_alert-type_alert-level.
Click on the hyperlinks for more details of the test.

Alert level A

PLAT972_ALERT_2_A Check Calcd Resid. Dens. 0.90Ang From U1 -3.98 eA-3

Author Response: Residual density is close to a heavy atom, and is present despite applying the correct absorption correction. It is not chemically significant.

Alert level B

PLAT971_ALERT_2_B Check Calcd Resid. Dens. 1.03Ang From U1 3.44 eA-3

Author Response: Residual density is close to a heavy atom, and is present despite applying the correct absorption correction. It is not chemically significant.

PLAT971_ALERT_2_B Check Calcd Resid. Dens. 1.04Ang From U1 3.38 eA-3

Author Response: Residual density is close to a heavy atom, and is present despite applying the correct absorption correction. It is not chemically significant.

PLAT972_ALERT_2_B Check Calcd Resid. Dens. 0.70Ang From U1 -3.03 eA-3

Author Response: Residual density is close to a heavy atom, and is present despite applying the correct absorption correction. It is not chemically significant.

Alert level C

```
PLAT029_ALERT_3_C _diffrn_measured_fraction_theta_full value Low .      0.979 Why?
PLAT042_ALERT_1_C Calc. and Reported MoietyFormula Strings  Differ     Please Check
PLAT202_ALERT_3_C Isotropic non-H Atoms in Anion/Solvent ........          6 Check
              C74A    O7      C79     C80     C81     C82
PLAT250_ALERT_2_C Large U3/U1 Ratio for Average U(i,j) Tensor ....        2.5 Note
PLAT331_ALERT_2_C Small Aver Phenyl C-C Dist C84      --C89      .       1.37 Ang.
PLAT342_ALERT_3_C Low Bond Precision on  C-C Bonds ..............    0.00933 Ang.
PLAT911_ALERT_3_C Missing FCF Refl Between Thmin & STh/L=    0.600        365 Report
PLAT913_ALERT_3_C Missing # of Very Strong Reflections in FCF ....          4 Note
PLAT971_ALERT_2_C Check Calcd Resid. Dens.  1.03Ang From C84             2.32 eA-3
```

Author Response: Residual density is close to a heavy atom, and is present despite applying the correct absorption correction. It is not chemically significant.

```
PLAT971_ALERT_2_C Check Calcd Resid. Dens.  0.63Ang From C87             1.81 eA-3
```

Author Response: Residual density is close to a heavy atom, and is present despite applying the correct absorption correction. It is not chemically significant.

```
PLAT971_ALERT_2_C Check Calcd Resid. Dens.  0.84Ang From As1             1.79 eA-3
```

Author Response: Residual density is close to a heavy atom, and is present despite applying the correct absorption correction. It is not chemically significant.

```
PLAT971_ALERT_2_C Check Calcd Resid. Dens.  0.74Ang From C88             1.66 eA-3
```

Author Response: Residual density is close to a heavy atom, and is present despite applying the correct absorption correction. It is not chemically significant.

```
PLAT971_ALERT_2_C Check Calcd Resid. Dens.  0.82Ang From C84             1.54 eA-3
```

Author Response: Residual density is close to a heavy atom, and is present despite applying the correct absorption correction. It is not chemically significant.

```
PLAT971_ALERT_2_C Check Calcd Resid. Dens.  1.68Ang From U1              1.52 eA-3
```

Author Response: Residual density is close to a heavy atom, and is present despite applying the correct absorption correction. It is not chemically significant.

```
PLAT971_ALERT_2_C Check Calcd Resid. Dens.  1.31Ang From U1             1.52 eA-3
```

Author Response: Residual density is close to a heavy atom, and is present despite applying the correct absorption correction. It is not chemically significant.

```
PLAT971_ALERT_2_C Check Calcd Resid. Dens.  0.91Ang From U1             1.52 eA-3
```

Author Response: Residual density is close to a heavy atom, and is present despite applying the correct absorption correction. It is not chemically significant.

```
PLAT971_ALERT_2_C Check Calcd Resid. Dens.  1.06Ang From N1             1.51 eA-3
```

Author Response: Residual density is close to a heavy atom, and is present despite applying the correct absorption correction. It is not chemically significant.

```
PLAT972_ALERT_2_C Check Calcd Resid. Dens.  0.75Ang From U1            -1.60 eA-3
```

Author Response: Residual density is close to a heavy atom, and is present despite applying the correct absorption correction. It is not chemically significant.

```
PLAT975_ALERT_2_C Check Calcd Resid. Dens.  0.88Ang From N2      .       0.90 eA-3
PLAT975_ALERT_2_C Check Calcd Resid. Dens.  1.02Ang From N4      .       0.84 eA-3
PLAT977_ALERT_2_C Check Negative Difference Density on H1        .      -0.35 eA-3
PLAT977_ALERT_2_C Check Negative Difference Density on H51C      .      -0.35 eA-3
```

Alert level G

```
PLAT002_ALERT_2_G Number of Distance or Angle Restraints on AtSite         85 Note
PLAT003_ALERT_2_G Number of Uiso or Uij Restrained non-H Atoms ...        140 Report
PLAT045_ALERT_1_G Calculated and Reported Z Differ by a Factor ...      0.500 Check
PLAT072_ALERT_2_G SHELXL First  Parameter in WGHT  Unusually Large       0.11 Report
PLAT154_ALERT_1_G The s.u.'s on the Cell Angles are Equal ..(Note)      0.001 Degree
PLAT172_ALERT_4_G The CIF-Embedded .res File Contains DFIX Records          5 Report
PLAT174_ALERT_4_G The CIF-Embedded .res File Contains FLAT Records          1 Report
PLAT176_ALERT_4_G The CIF-Embedded .res File Contains SADI Records          8 Report
PLAT178_ALERT_4_G The CIF-Embedded .res File Contains SIMU Records          1 Report
PLAT187_ALERT_4_G The CIF-Embedded .res File Contains RIGU Records          1 Report
PLAT232_ALERT_2_G Hirshfeld Test Diff (M-X)  U1       --As1      .       11.8 s.u.
PLAT300_ALERT_4_G Atom Site Occupancy of C83        Constrained at        0.5 Check
PLAT300_ALERT_4_G Atom Site Occupancy of C84        Constrained at        0.5 Check
```

```
PLAT300_ALERT_4_G Atom Site Occupancy of C85       Constrained at        0.5 Check
PLAT300_ALERT_4_G Atom Site Occupancy of C86       Constrained at        0.5 Check
PLAT300_ALERT_4_G Atom Site Occupancy of C87       Constrained at        0.5 Check
PLAT300_ALERT_4_G Atom Site Occupancy of C88       Constrained at        0.5 Check
PLAT300_ALERT_4_G Atom Site Occupancy of C89       Constrained at        0.5 Check
PLAT300_ALERT_4_G Atom Site Occupancy of H83A      Constrained at        0.5 Check
PLAT300_ALERT_4_G Atom Site Occupancy of H83B      Constrained at        0.5 Check
PLAT300_ALERT_4_G Atom Site Occupancy of H83C      Constrained at        0.5 Check
PLAT300_ALERT_4_G Atom Site Occupancy of H85       Constrained at        0.5 Check
PLAT300_ALERT_4_G Atom Site Occupancy of H86       Constrained at        0.5 Check
PLAT300_ALERT_4_G Atom Site Occupancy of H87       Constrained at        0.5 Check
PLAT300_ALERT_4_G Atom Site Occupancy of H88       Constrained at        0.5 Check
PLAT300_ALERT_4_G Atom Site Occupancy of H89       Constrained at        0.5 Check
PLAT301_ALERT_3_G Main Residue  Disorder .............(Resd  1  )        13% Note
PLAT302_ALERT_4_G Anion/Solvent/Minor-Residue Disorder (Resd  2  )       67% Note
PLAT302_ALERT_4_G Anion/Solvent/Minor-Residue Disorder (Resd  3  )      100% Note
PLAT302_ALERT_4_G Anion/Solvent/Minor-Residue Disorder (Resd  4  )      100% Note
PLAT302_ALERT_4_G Anion/Solvent/Minor-Residue Disorder (Resd  5  )      100% Note
PLAT303_ALERT_2_G Full Occupancy Atom H1        with # Connections      2.00 Check
PLAT304_ALERT_4_G Non-Integer Number of Atoms in ..... (Resd  3  )      7.50 Check
PLAT304_ALERT_4_G Non-Integer Number of Atoms in ..... (Resd  4  )      8.76 Check
PLAT304_ALERT_4_G Non-Integer Number of Atoms in ..... (Resd  5  )      6.24 Check
PLAT410_ALERT_2_G Short Intra H...H Contact  H48A     ..H55      .      2.05 Ang.
                                                     x,y,z  =     1_555 Check
PLAT410_ALERT_2_G Short Intra H...H Contact  H56A     ..H44C     .      2.13 Ang.
                                                     x,y,z  =     1_555 Check
PLAT410_ALERT_2_G Short Intra H...H Contact  H60B     ..H54C     .      2.09 Ang.
                                                     x,y,z  =     1_555 Check
PLAT410_ALERT_2_G Short Intra H...H Contact  H75B     ..H74A     .      2.07 Ang.
                                                     x,y,z  =     1_555 Check
PLAT410_ALERT_2_G Short Intra H...H Contact  H76B     ..H77C     .      2.05 Ang.
                                                     x,y,z  =     1_555 Check
PLAT432_ALERT_2_G Short Inter X...Y Contact  C50      ..C83      .      2.88 Ang.
                                                1-x,-y,2-z  =     2_657 Check
PLAT720_ALERT_4_G Number of Unusual/Non-Standard Labels ..........        10 Note
PLAT802_ALERT_4_G CIF Input Record(s) with more than 80 Characters         9 Info
PLAT811_ALERT_5_G No ADDSYM Analysis: Too Many Excluded Atoms ....         ! Info
PLAT860_ALERT_3_G Number of Least-Squares Restraints .............      2867 Note
PLAT912_ALERT_4_G Missing # of FCF Reflections Above STh/L=  0.600        16 Note
PLAT933_ALERT_2_G Number of HKL-OMIT Records in Embedded .res File         2 Note
PLAT941_ALERT_3_G Average HKL Measurement Multiplicity ...........       3.4 Low
PLAT978_ALERT_2_G Number C-C Bonds with Positive Residual Density.         0 Info
```

```
   1 ALERT level A = Most likely a serious problem - resolve or explain
   3 ALERT level B = A potentially serious problem, consider carefully
  22 ALERT level C = Check. Ensure it is not caused by an omission or oversight
  49 ALERT level G = General information/check it is not something unexpected

   3 ALERT type 1 CIF construction/syntax error, inconsistent or missing data
  33 ALERT type 2 Indicator that the structure model may be wrong or deficient
   8 ALERT type 3 Indicator that the structure quality may be low
  30 ALERT type 4 Improvement, methodology, query or suggestion
   1 ALERT type 5 Informative message, check
```

Datablock: 9

Bond precision:	C-C = 0.0063 A		Wavelength=0.71073
Cell:	a=10.8541(3)	b=33.5203(10)	c=18.6864(4)
	alpha=90	beta=95.295(2)	gamma=90
Temperature:	150 K		

	Calculated	Reported
Volume	6769.7(3)	6769.7(3)
Space group	P 21/n	P 1 21/n 1
Hall group	-P 2yn	-P 2yn
Moiety formula	C60 H113 N4 P Si3 Th, C6 H6	C60 H113 N4 P Si3 Th, C6 H6
Sum formula	C66 H119 N4 P Si3 Th	C66 H119 N4 P Si3 Th
Mr	1315.94	1315.92
Dx,g cm-3	1.291	1.291
Z	4	4
Mu (mm-1)	2.319	2.319
F000	2760.0	2760.0
F000'	2733.19	
h,k,lmax	14,45,25	14,45,25
Nref	18275	16298
Tmin,Tmax	0.846,0.911	0.934,1.000
Tmin'	0.846	

Correction method= # Reported T Limits: Tmin=0.934 Tmax=1.000
AbsCorr = GAUSSIAN

Data completeness= 0.892 Theta(max)= 29.157

R(reflections)= 0.0433(12372) wR2(reflections)= 0.1007(16298)

S = 1.038 Npar= 829

The following ALERTS were generated. Each ALERT has the format
test-name_ALERT_alert-type_alert-level.
Click on the hyperlinks for more details of the test.

Alert level C

```
PLAT220_ALERT_2_C NonSolvent   Resd 1  C   Ueq(max)/Ueq(min) Range        3.1 Ratio
PLAT244_ALERT_4_C Low    'Solvent' Ueq as Compared to Neighbors of        C65 Check
PLAT414_ALERT_2_C Short Intra D-H..H-X       H1B      ..H40B     .       1.92 Ang.
                                                    x,y,z  =      1_555 Check
PLAT906_ALERT_3_C Large K Value in the Analysis of Variance ......      4.337 Check
```

```
PLAT911_ALERT_3_C Missing FCF Refl Between Thmin & STh/L=    0.600          3 Report
PLAT971_ALERT_2_C Check Calcd Resid. Dens.  0.83Ang From Th1             1.84 eA-3
PLAT971_ALERT_2_C Check Calcd Resid. Dens.  0.86Ang From Th1             1.60 eA-3
PLAT972_ALERT_2_C Check Calcd Resid. Dens.  0.67Ang From Th1            -1.75 eA-3
PLAT973_ALERT_2_C Check Calcd Positive Resid. Density on       Th1       1.40 eA-3
PLAT977_ALERT_2_C Check Negative Difference Density on H1A       .      -0.34 eA-3
PLAT977_ALERT_2_C Check Negative Difference Density on H1B       .      -0.51 eA-3
```

Alert level G

```
PLAT002_ALERT_2_G Number of Distance or Angle Restraints on AtSite         44 Note
PLAT003_ALERT_2_G Number of Uiso or Uij Restrained non-H Atoms ...         40 Report
PLAT172_ALERT_4_G The CIF-Embedded .res File Contains DFIX Records          5 Report
PLAT187_ALERT_4_G The CIF-Embedded .res File Contains RIGU Records          1 Report
PLAT232_ALERT_2_G Hirshfeld Test Diff (M-X)  Th1      --P1       .       18.5 s.u.
PLAT301_ALERT_3_G Main Residue  Disorder ..............(Resd  1  )        23% Note
PLAT328_ALERT_4_G Possible Missing H on sp3? Phosphorus ..........         P1 Check
PLAT410_ALERT_2_G Short Intra H...H Contact  H42A     ..H56A     .       2.05 Ang.
                                                  x,y,z  =      1_555 Check
PLAT410_ALERT_2_G Short Intra H...H Contact  H44D     ..H54A     .       1.83 Ang.
                                                  x,y,z  =      1_555 Check
PLAT410_ALERT_2_G Short Intra H...H Contact  H50B     ..H56D     .       2.06 Ang.
                                                  x,y,z  =      1_555 Check
PLAT794_ALERT_5_G Tentative Bond Valency for Th1       (IV)      .       4.06 Info
PLAT802_ALERT_4_G CIF Input Record(s) with more than 80 Characters          6 Info
PLAT860_ALERT_3_G Number of Least-Squares Restraints ............         298 Note
PLAT910_ALERT_3_G Missing # of FCF Reflection(s) Below Theta(Min).          3 Note
PLAT912_ALERT_4_G Missing # of FCF Reflections Above STh/L=  0.600       1971 Note
PLAT933_ALERT_2_G Number of HKL-OMIT Records in Embedded .res File          3 Note
PLAT941_ALERT_3_G Average HKL Measurement Multiplicity ..........         4.4 Low
PLAT978_ALERT_2_G Number C-C Bonds with Positive Residual Density.          0 Info
```

---

0 **ALERT level A** = Most likely a serious problem - resolve or explain
0 **ALERT level B** = A potentially serious problem, consider carefully
11 **ALERT level C** = Check. Ensure it is not caused by an omission or oversight
18 **ALERT level G** = General information/check it is not something unexpected

0 ALERT type 1 CIF construction/syntax error, inconsistent or missing data
16 ALERT type 2 Indicator that the structure model may be wrong or deficient
6 ALERT type 3 Indicator that the structure quality may be low
6 ALERT type 4 Improvement, methodology, query or suggestion
1 ALERT type 5 Informative message, check

Datablock: 10

Bond precision: C-C = 0.0076 A   Wavelength=0.71073

Cell:	a=9.8582(3)	b=12.8737(4)	c=26.2610(7)
	alpha=97.089(2)	beta=100.523(2)	gamma=92.476(2)
Temperature:	150 K		

	Calculated	Reported
Volume	3244.33(17)	3244.33(17)
Space group	P -1	P -1
Hall group	-P 1	-P 1
Moiety formula	2(C60 H113 As N4 Si3 Th), C7 H8	C60 H113 As N4 Si3 Th, 0.5(C7 H8)
Sum formula	C127 H234 As2 N8 Si6 Th2	C63.50 H117 As N4 Si3 Th
Mr	2655.70	1327.83
Dx,g cm-3	1.359	1.359
Z	1	2
Mu (mm-1)	2.899	2.899
F000	1382.0	1382.0
F000'	1368.50	
h,k,lmax	13,17,35	13,16,34
Nref	17410	14866
Tmin,Tmax	0.814,0.896	0.870,0.982
Tmin'	0.814	

Correction method= # Reported T Limits: Tmin=0.870 Tmax=0.982
AbsCorr = GAUSSIAN

Data completeness= 0.854 Theta(max)= 29.090

R(reflections)= 0.0490(12721) wR2(reflections)= 0.1158(14866)

S = 1.061 Npar= 658

The following ALERTS were generated. Each ALERT has the format
test-name_ALERT_alert-type_alert-level.
Click on the hyperlinks for more details of the test.

Alert level A

PLAT971_ALERT_2_A Check Calcd Resid. Dens. 0.79Ang From As1 5.70 eA-3

Author Response: Residual density is close to a heavy atom, and is present despite applying the correct absorption correction. It is not chemically signifi

PLAT972_ALERT_2_A Check Calcd Resid. Dens. 0.74Ang From As1 -5.02 eA-3

Author Response: Residual density is close to a heavy atom, and is present despite applying the correct absorption correction. It is not chemically signifi

```
PLAT972_ALERT_2_A Check Calcd Resid. Dens.  0.31Ang From As1           -3.94 eA-3
```

Author Response: Residual density is close to a heavy atom, and is present despite applying the correct absorption correction. It is not chemically signifi

---

Alert level B

```
PLAT971_ALERT_2_B Check Calcd Resid. Dens.  0.27Ang From As1            3.05 eA-3
```

Author Response: Residual density is close to a heavy atom, and is present despite applying the correct absorption correction. It is not chemically signifi

```
PLAT971_ALERT_2_B Check Calcd Resid. Dens.  1.07Ang From Th1            2.54 eA-3
```

Author Response: Residual density is close to a heavy atom, and is present despite applying the correct absorption correction. It is not chemically signifi

---

Alert level C

```
PLAT042_ALERT_1_C Calc. and Reported MoietyFormula Strings  Differ     Please Check
PLAT222_ALERT_3_C NonSolvent Resd 1  H   Uiso(max)/Uiso(min) Range        8.1 Ratio
PLAT410_ALERT_2_C Short Intra H...H Contact  H1B      ..H37B     .       1.98 Ang.
                                                      x,y,z  =      1_555 Check
PLAT420_ALERT_2_C D-H Bond Without Acceptor  As1      --H1A      .     Please Check
PLAT420_ALERT_2_C D-H Bond Without Acceptor  As1      --H1B      .     Please Check
PLAT911_ALERT_3_C Missing FCF Refl Between Thmin & STh/L=    0.600          5 Report
PLAT971_ALERT_2_C Check Calcd Resid. Dens.  1.08Ang From Th1            2.15 eA-3
```

Author Response: Residual density is close to a heavy atom, and is present despite applying the correct absorption correction. It is not chemically signifi

```
PLAT971_ALERT_2_C Check Calcd Resid. Dens.  0.81Ang From Th1            2.03 eA-3
```

Author Response: Residual density is close to a heavy atom, and is present despite applying the correct absorption correction. It is not chemically signifi

```
PLAT971_ALERT_2_C Check Calcd Resid. Dens.  0.87Ang From Th1            1.88 eA-3
```

Author Response: Residual density is close to a heavy atom, and is present despite applying the correct absorption correction. It is not chemically signifi

```
PLAT972_ALERT_2_C Check Calcd Resid. Dens.  0.69Ang From Th1           -1.86 eA-3
```

Author Response: Residual density is close to a heavy atom, and is present despite applying the correct absorption correction. It is not chemically signifi

```
PLAT972_ALERT_2_C Check Calcd Resid. Dens.  1.35Ang From Th1           -1.83 eA-3
```

Author Response: Residual density is close to a heavy atom, and is present despite applying the correct absorption correction. It is not chemically signifi

```
PLAT972_ALERT_2_C Check Calcd Resid. Dens.  1.37Ang From Th1           -1.73 eA-3
```

Author Response: Residual density is close to a heavy atom, and is present despite applying the correct absorption correction. It is not chemically signifi

```
PLAT972_ALERT_2_C Check Calcd Resid. Dens.  0.69Ang From Th1           -1.72 eA-3
```

Author Response: Residual density is close to a heavy atom, and is present despite applying the correct absorption correction. It is not chemically signifi

```
PLAT973_ALERT_2_C Check Calcd Positive Resid. Density on       Th1       1.06 eA-3
PLAT977_ALERT_2_C Check Negative Difference Density on H16B      .      -0.31 eA-3
PLAT977_ALERT_2_C Check Negative Difference Density on H61C      .      -0.31 eA-3
```

Alert level G

```
PLAT002_ALERT_2_G Number of Distance or Angle Restraints on AtSite         11 Note
PLAT003_ALERT_2_G Number of Uiso or Uij Restrained non-H Atoms ...          7 Report
PLAT045_ALERT_1_G Calculated and Reported Z Differ by a Factor ...      0.500 Check
PLAT083_ALERT_2_G SHELXL Second Parameter in WGHT  Unusually Large       5.85 Why ?
PLAT154_ALERT_1_G The s.u.'s on the Cell Angles are Equal ..(Note)      0.002 Degree
PLAT172_ALERT_4_G The CIF-Embedded .res File Contains DFIX Records          5 Report
PLAT174_ALERT_4_G The CIF-Embedded .res File Contains FLAT Records          1 Report
PLAT176_ALERT_4_G The CIF-Embedded .res File Contains SADI Records          2 Report
PLAT178_ALERT_4_G The CIF-Embedded .res File Contains SIMU Records          1 Report
PLAT232_ALERT_2_G Hirshfeld Test Diff (M-X)  Th1      --As1      .        9.8 s.u.
PLAT300_ALERT_4_G Atom Site Occupancy of C61        Constrained at        0.5 Check
PLAT300_ALERT_4_G Atom Site Occupancy of C62        Constrained at        0.5 Check
PLAT300_ALERT_4_G Atom Site Occupancy of C63        Constrained at        0.5 Check
PLAT300_ALERT_4_G Atom Site Occupancy of C64        Constrained at        0.5 Check
PLAT300_ALERT_4_G Atom Site Occupancy of C65        Constrained at        0.5 Check
PLAT300_ALERT_4_G Atom Site Occupancy of C66        Constrained at        0.5 Check
PLAT300_ALERT_4_G Atom Site Occupancy of C67        Constrained at        0.5 Check
PLAT300_ALERT_4_G Atom Site Occupancy of H61A       Constrained at        0.5 Check
PLAT300_ALERT_4_G Atom Site Occupancy of H61B       Constrained at        0.5 Check
PLAT300_ALERT_4_G Atom Site Occupancy of H61C       Constrained at        0.5 Check
PLAT300_ALERT_4_G Atom Site Occupancy of H63        Constrained at        0.5 Check
PLAT300_ALERT_4_G Atom Site Occupancy of H64        Constrained at        0.5 Check
PLAT300_ALERT_4_G Atom Site Occupancy of H65        Constrained at        0.5 Check
PLAT300_ALERT_4_G Atom Site Occupancy of H66        Constrained at        0.5 Check
PLAT300_ALERT_4_G Atom Site Occupancy of H67        Constrained at        0.5 Check
PLAT302_ALERT_4_G Anion/Solvent/Minor-Residue Disorder (Resd  2  )       100% Note
PLAT304_ALERT_4_G Non-Integer Number of Atoms in ..... (Resd  2  )       7.50 Check
```

```
PLAT380_ALERT_4_G Incorrectly? Oriented X(sp2)-Methyl Moiety .....        C61 Check
PLAT413_ALERT_2_G Short Inter XH3 .. XHn     H40B     ..H61C     .       2.10 Ang.
                                                      x,y,z  =      1_555 Check
PLAT789_ALERT_4_G Atoms with Negative _atom_site_disorder_group  #         15 Check
PLAT794_ALERT_5_G Tentative Bond Valency for Th1       (IV)      .       4.10 Info
PLAT802_ALERT_4_G CIF Input Record(s) with more than 80 Characters          7 Info
PLAT860_ALERT_3_G Number of Least-Squares Restraints ............          39 Note
PLAT910_ALERT_3_G Missing # of FCF Reflection(s) Below Theta(Min).          4 Note
PLAT912_ALERT_4_G Missing # of FCF Reflections Above STh/L=  0.600       2401 Note
PLAT941_ALERT_3_G Average HKL Measurement Multiplicity ..........         2.4 Low
PLAT978_ALERT_2_G Number C-C Bonds with Positive Residual Density.          2 Info
```

```
   3 ALERT level A = Most likely a serious problem - resolve or explain
   2 ALERT level B = A potentially serious problem, consider carefully
  16 ALERT level C = Check. Ensure it is not caused by an omission or oversight
  37 ALERT level G = General information/check it is not something unexpected

   3 ALERT type 1 CIF construction/syntax error, inconsistent or missing data
  24 ALERT type 2 Indicator that the structure model may be wrong or deficient
   5 ALERT type 3 Indicator that the structure quality may be low
  25 ALERT type 4 Improvement, methodology, query or suggestion
   1 ALERT type 5 Informative message, check
```

Datablock: 11

Bond precision: C-C = 0.0084 A Wavelength=1.54184

Cell:	a=9.5639(3)	b=13.3160(4)	c=25.4526(7)
	alpha=78.871(2)	beta=82.668(2)	gamma=80.659(2)
Temperature:	150 K		

	Calculated	Reported
Volume	3122.77(16)	3122.77(16)
Space group	P -1	P -1
Hall group	-P 1	-P 1
Moiety formula	C60 H113 N4 P Si3 U	C60 H113 N4 P Si3 U
Sum formula	C60 H113 N4 P Si3 U	C60 H113 N4 P Si3 U
Mr	1243.82	1243.81
Dx,g cm-3	1.323	1.323
Z	2	2
Mu (mm-1)	8.387	8.387
F000	1300.0	1300.0
F000’	1295.95	
h,k,lmax	12,16,32	11,16,31
Nref	13037	12517
Tmin,Tmax	0.489,0.630	0.534,1.000
Tmin’	0.100	

```
Correction method= # Reported T Limits: Tmin=0.534 Tmax=1.000
AbsCorr = GAUSSIAN

Data completeness= 0.960          Theta(max)= 76.108

R(reflections)= 0.0396( 11638)    wR2(reflections)= 0.0930( 12517)

S = 1.057                 Npar= 628
```

The following ALERTS were generated. Each ALERT has the format
test-name_ALERT_alert-type_alert-level.
Click on the hyperlinks for more details of the test.

Alert level B

```
PLAT972_ALERT_2_B Check Calcd Resid. Dens.  0.75Ang From U1          -2.51 eA-3
```

Author Response: Residual density is close to a heavy atom, and is present despite applying the correct absorption correction. It is not chemically significant.

Alert level C

```
PLAT220_ALERT_2_C NonSolvent   Resd 1   C   Ueq(max)/Ueq(min) Range        3.4 Ratio
PLAT230_ALERT_2_C Hirshfeld Test Diff for    C46      --C47      .         5.6 s.u.
PLAT230_ALERT_2_C Hirshfeld Test Diff for    C57      --C58      .         5.4 s.u.
PLAT241_ALERT_2_C High   'MainMol' Ueq as Compared to Neighbors of        C47 Check
PLAT342_ALERT_3_C Low Bond Precision on  C-C Bonds ...............     0.00835 Ang.
PLAT410_ALERT_2_C Short Intra H...H Contact  H42B      ..H43      .        1.91 Ang.
                                                       x,y,z  =      1_555 Check
PLAT601_ALERT_2_C Unit Cell Contains Solvent Accessible VOIDS of .          33 Ang**3
PLAT906_ALERT_3_C Large K Value in the Analysis of Variance ......       2.166 Check
PLAT911_ALERT_3_C Missing FCF Refl Between Thmin & STh/L=    0.600          67 Report
PLAT972_ALERT_2_C Check Calcd Resid. Dens.  0.76Ang From U1          -2.46 eA-3
```

Author Response: Residual density is close to a heavy atom, and is present despite applying the correct absorption correction. It is not chemically significant.

```
PLAT972_ALERT_2_C Check Calcd Resid. Dens.  0.80Ang From U1          -1.71 eA-3
```

Author Response: Residual density is close to a heavy atom, and is present despite applying the correct absorption correction. It is not chemically significant.

```
PLAT972_ALERT_2_C Check Calcd Resid. Dens.  0.73Ang From U1           -1.58 eA-3
```

Author Response: Residual density is close to a heavy atom, and is present despite applying the correct absorption correction. It is not chemically significant.

```
PLAT972_ALERT_2_C Check Calcd Resid. Dens.  0.76Ang From U1           -1.52 eA-3
```

Author Response: Residual density is close to a heavy atom, and is present despite applying the correct absorption correction. It is not chemically significant.

```
PLAT977_ALERT_2_C Check Negative Difference Density on H1B       .      -0.53 eA-3
```

Alert level G

```
PLAT002_ALERT_2_G Number of Distance or Angle Restraints on AtSite          4 Note
PLAT083_ALERT_2_G SHELXL Second Parameter in WGHT  Unusually Large      11.93 Why ?
PLAT154_ALERT_1_G The s.u.'s on the Cell Angles are Equal ..(Note)      0.002 Degree
PLAT172_ALERT_4_G The CIF-Embedded .res File Contains DFIX Records          3 Report
PLAT232_ALERT_2_G Hirshfeld Test Diff (M-X)  U1       --P1       .       10.4 s.u.
PLAT232_ALERT_2_G Hirshfeld Test Diff (M-X)  U1       --N1       .        6.1 s.u.
PLAT232_ALERT_2_G Hirshfeld Test Diff (M-X)  U1       --N2       .        5.4 s.u.
PLAT328_ALERT_4_G Possible Missing H on sp3? Phosphorus ..........         P1 Check
PLAT802_ALERT_4_G CIF Input Record(s) with more than 80 Characters          2 Info
PLAT860_ALERT_3_G Number of Least-Squares Restraints ............           5 Note
PLAT912_ALERT_4_G Missing # of FCF Reflections Above STh/L=  0.600        439 Note
PLAT941_ALERT_3_G Average HKL Measurement Multiplicity ..........         2.8 Low
PLAT978_ALERT_2_G Number C-C Bonds with Positive Residual Density.          0 Info
```

```
   0 ALERT level A = Most likely a serious problem - resolve or explain
   1 ALERT level B = A potentially serious problem, consider carefully
  14 ALERT level C = Check. Ensure it is not caused by an omission or oversight
  13 ALERT level G = General information/check it is not something unexpected

   1 ALERT type 1 CIF construction/syntax error, inconsistent or missing data
  18 ALERT type 2 Indicator that the structure model may be wrong or deficient
   5 ALERT type 3 Indicator that the structure quality may be low
   4 ALERT type 4 Improvement, methodology, query or suggestion
   0 ALERT type 5 Informative message, check
```

Datablock: 12

```
Bond precision:   C-C = 0.0120 A          Wavelength=1.54184

Cell:             a=9.6385(1)        b=13.2463(3)       c=25.4050(4)
                  alpha=78.882(2)    beta=82.799(1)     gamma=80.727(1)
Temperature:      150 K
```

	Calculated	Reported
Volume	3126.18(9)	3126.18(10)
Space group	P -1	P -1
Hall group	-P 1	-P 1
Moiety formula	C60 H113 As N4 Si3 U	C60 H113 As N4 Si3 U
Sum formula	C60 H113 As N4 Si3 U	C60 H113 As N4 Si3 U
Mr	1287.77	1287.76
Dx,g cm-3	1.368	1.368
Z	2	2
Mu (mm-1)	8.724	8.724
F000	1336.0	1336.0
F000'	1329.48	
h,k,lmax	12,16,32	12,16,32
Nref	13346	13044
Tmin,Tmax	0.390,0.511	0.427,0.950
Tmin'	0.295	

Correction method= # Reported T Limits: Tmin=0.427 Tmax=0.950
AbsCorr = GAUSSIAN

Data completeness= 0.977 Theta(max)= 77.859

R(reflections)= 0.0589(12078) wR2(reflections)= 0.1509(13044)

S = 1.086 Npar= 685

The following ALERTS were generated. Each ALERT has the format
test-name_ALERT_alert-type_alert-level.
Click on the hyperlinks for more details of the test.

Alert level A

PLAT972_ALERT_2_A Check Calcd Resid. Dens. 0.75Ang From U1 -4.09 eA-3

Author Response: Residual density is close to a heavy atom, and is present despite applying the correct absorption correction. It is not chemically significant.

PLAT972_ALERT_2_A Check Calcd Resid. Dens. 0.75Ang From U1 -3.81 eA-3

Author Response: Residual density is close to a heavy atom, and is present despite applying the correct absorption correction. It is not chemically significant.

Alert level B

```
PLAT971_ALERT_2_B Check Calcd Resid. Dens.  1.31Ang From U1             3.03 eA-3
```

Author Response: Residual density is close to a heavy atom, and is present despite applying the correct absorption correction. It is not chemically significant.

```
PLAT971_ALERT_2_B Check Calcd Resid. Dens.  0.91Ang From U1             2.81 eA-3
```

Author Response: Residual density is close to a heavy atom, and is present despite applying the correct absorption correction. It is not chemically significant.

```
PLAT971_ALERT_2_B Check Calcd Resid. Dens.  0.92Ang From U1             2.71 eA-3
```

Author Response: Residual density is close to a heavy atom, and is present despite applying the correct absorption correction. It is not chemically significant.

```
PLAT976_ALERT_2_B Check Calcd Resid. Dens.  0.97Ang From N3      .     -1.72 eA-3
```

Author Response: Residual density is close to a heavy atom, and is present despite applying the correct absorption correction. It is not chemically significant.

Alert level C

```
PLAT213_ALERT_2_C Atom C15             has ADP max/min Ratio .....        3.5 prolat
PLAT220_ALERT_2_C NonSolvent   Resd 1  C   Ueq(max)/Ueq(min) Range        3.3 Ratio
PLAT234_ALERT_4_C Large Hirshfeld Difference C17      --C18      .       0.19 Ang.
PLAT241_ALERT_2_C High   'MainMol' Ueq as Compared to Neighbors of        C17 Check
PLAT342_ALERT_3_C Low Bond Precision on  C-C Bonds ..............     0.01196 Ang.
PLAT420_ALERT_2_C D-H Bond Without Acceptor  As1      --H1A      .     Please Check
PLAT420_ALERT_2_C D-H Bond Without Acceptor  As1      --H1B      .     Please Check
PLAT911_ALERT_3_C Missing FCF Refl Between Thmin & STh/L=    0.600         15 Report
PLAT971_ALERT_2_C Check Calcd Resid. Dens.  1.18Ang From N3             2.46 eA-3
```

Author Response: Residual density is close to a heavy atom, and is present despite applying the correct absorption correction. It is not chemically significant.

```
PLAT971_ALERT_2_C Check Calcd Resid. Dens.  0.97Ang From U1             1.98 eA-3
```

Author Response: Residual density is close to a heavy atom, and is present despite applying the correct absorption correction. It is not chemically significant.

```
PLAT971_ALERT_2_C Check Calcd Resid. Dens.  1.08Ang From U1             1.85 eA-3
```

Author Response: Residual density is close to a heavy atom, and is present despite applying the correct absorption correction. It is not chemically significant.

```
PLAT971_ALERT_2_C Check Calcd Resid. Dens.  1.08Ang From U1             1.73 eA-3
```

Author Response: Residual density is close to a heavy atom, and is present despite applying the correct absorption correction. It is not chemically significant.

```
PLAT971_ALERT_2_C Check Calcd Resid. Dens.  0.95Ang From U1             1.69 eA-3
```

Author Response: Residual density is close to a heavy atom, and is present despite applying the correct absorption correction. It is not chemically significant.

```
PLAT972_ALERT_2_C Check Calcd Resid. Dens.  0.76Ang From U1            -2.47 eA-3
```

Author Response: Residual density is close to a heavy atom, and is present despite applying the correct absorption correction. It is not chemically significant.

```
PLAT972_ALERT_2_C Check Calcd Resid. Dens.  0.72Ang From U1            -2.40 eA-3
```

Author Response: Residual density is close to a heavy atom, and is present despite applying the correct absorption correction. It is not chemically significant.

```
PLAT972_ALERT_2_C Check Calcd Resid. Dens.  0.83Ang From U1            -2.11 eA-3
```

Author Response: Residual density is close to a heavy atom, and is present despite applying the correct absorption correction. It is not chemically significant.

```
PLAT972_ALERT_2_C Check Calcd Resid. Dens.  0.79Ang From As1           -1.92 eA-3
```

Author Response: Residual density is close to a heavy atom, and is present despite applying the correct absorption correction. It is not chemically significant.

```
PLAT972_ALERT_2_C Check Calcd Resid. Dens.  1.47Ang From U1           -1.75 eA-3
```

Author Response: Residual density is close to a heavy atom, and is present despite applying the correct absorption correction. It is not chemically significant.

```
PLAT972_ALERT_2_C Check Calcd Resid. Dens.  0.97Ang From N3           -1.72 eA-3
```

Author Response: Residual density is close to a heavy atom, and is present despite applying the correct absorption correction. It is not chemically significant.

```
PLAT972_ALERT_2_C Check Calcd Resid. Dens.  0.76Ang From U1           -1.67 eA-3
```

Author Response: Residual density is close to a heavy atom, and is present despite applying the correct absorption correction. It is not chemically significant.

```
PLAT977_ALERT_2_C Check Negative Difference Density on H19A      .      -0.41 eA-3
```

Alert level G

```
PLAT002_ALERT_2_G Number of Distance or Angle Restraints on AtSite         19 Note
PLAT003_ALERT_2_G Number of Uiso or Uij Restrained non-H Atoms ...         12 Report
PLAT083_ALERT_2_G SHELXL Second Parameter in WGHT  Unusually Large      22.56 Why ?
PLAT172_ALERT_4_G The CIF-Embedded .res File Contains DFIX Records          3 Report
PLAT176_ALERT_4_G The CIF-Embedded .res File Contains SADI Records          2 Report
PLAT178_ALERT_4_G The CIF-Embedded .res File Contains SIMU Records          1 Report
PLAT187_ALERT_4_G The CIF-Embedded .res File Contains RIGU Records          1 Report
PLAT232_ALERT_2_G Hirshfeld Test Diff (M-X)  U1       --As1      .        5.2 s.u.
PLAT232_ALERT_2_G Hirshfeld Test Diff (M-X)  U1       --N2       .        9.0 s.u.
PLAT301_ALERT_3_G Main Residue  Disorder ..............(Resd  1  )         9% Note
PLAT410_ALERT_2_G Short Intra H...H Contact  H2A      ..H3       .       1.97 Ang.
                                                      x,y,z  =      1_555 Check
PLAT410_ALERT_2_G Short Intra H...H Contact  H2A      ..H3A      .       1.99 Ang.
                                                      x,y,z  =      1_555 Check
PLAT410_ALERT_2_G Short Intra H...H Contact  H14A     ..H4AA     .       2.10 Ang.
                                                      x,y,z  =      1_555 Check
PLAT720_ALERT_4_G Number of Unusual/Non-Standard Labels ..........         10 Note
PLAT722_ALERT_1_G Angle   Calc      109.00, Rep      110.10 Dev...       1.10 Degree
              C6A     -C5A     -H5AA       1_555  1_555  1_555     #  411 Check
PLAT802_ALERT_4_G CIF Input Record(s) with more than 80 Characters          3 Info
PLAT860_ALERT_3_G Number of Least-Squares Restraints .............        327 Note
PLAT912_ALERT_4_G Missing # of FCF Reflections Above STh/L=  0.600        287 Note
PLAT941_ALERT_3_G Average HKL Measurement Multiplicity ...........        4.0 Low
PLAT978_ALERT_2_G Number C-C Bonds with Positive Residual Density.          0 Info
```

```
 2 ALERT level A = Most likely a serious problem - resolve or explain
 4 ALERT level B = A potentially serious problem, consider carefully
21 ALERT level C = Check. Ensure it is not caused by an omission or oversight
20 ALERT level G = General information/check it is not something unexpected

 1 ALERT type 1 CIF construction/syntax error, inconsistent or missing data
33 ALERT type 2 Indicator that the structure model may be wrong or deficient
 5 ALERT type 3 Indicator that the structure quality may be low
 8 ALERT type 4 Improvement, methodology, query or suggestion
 0 ALERT type 5 Informative message, check
```

It is advisable to attempt to resolve as many as possible of the alerts in all categories. Often the minor alerts point to easily fixed oversights, errors and omissions in your CIF or refinement strategy, so attention to these fine details can be worthwhile. In order to resolve some of the more serious problems it may be necessary to carry out additional measurements or structure refinements. However, the purpose of your study may justify the reported deviations and the more serious of these should normally be commented upon in the discussion or experimental section of a paper or in the "special_details" fields of the CIF. checkCIF was carefully designed to identify outliers and unusual parameters, but every test has its limitations and alerts that are not important in a particular case may appear. Conversely, the absence of alerts does not guarantee there are no aspects of the results needing attention. It is up to the individual to critically assess their own results and, if necessary, seek expert advice.

Publication of your CIF in IUCr journals

A basic structural check has been run on your CIF. These basic checks will be run on all CIFs submitted for publication in IUCr journals (*Acta Crystallographica*, *Journal of Applied Crystallography*, *Journal of Synchrotron Radiation*); however, if you intend to submit to *Acta Crystallographica Section C* or *E* or *IUCrData*, you should make sure that full publication checks are run on the final version of your CIF prior to submission.

Publication of your CIF in other journals

Please refer to the *Notes for Authors* of the relevant journal for any special instructions relating to CIF submission.

PLATON version of 12/09/2022; check.def file version of 09/08/2022

Datablock TRENTCHSLi3 - ellipsoid plot

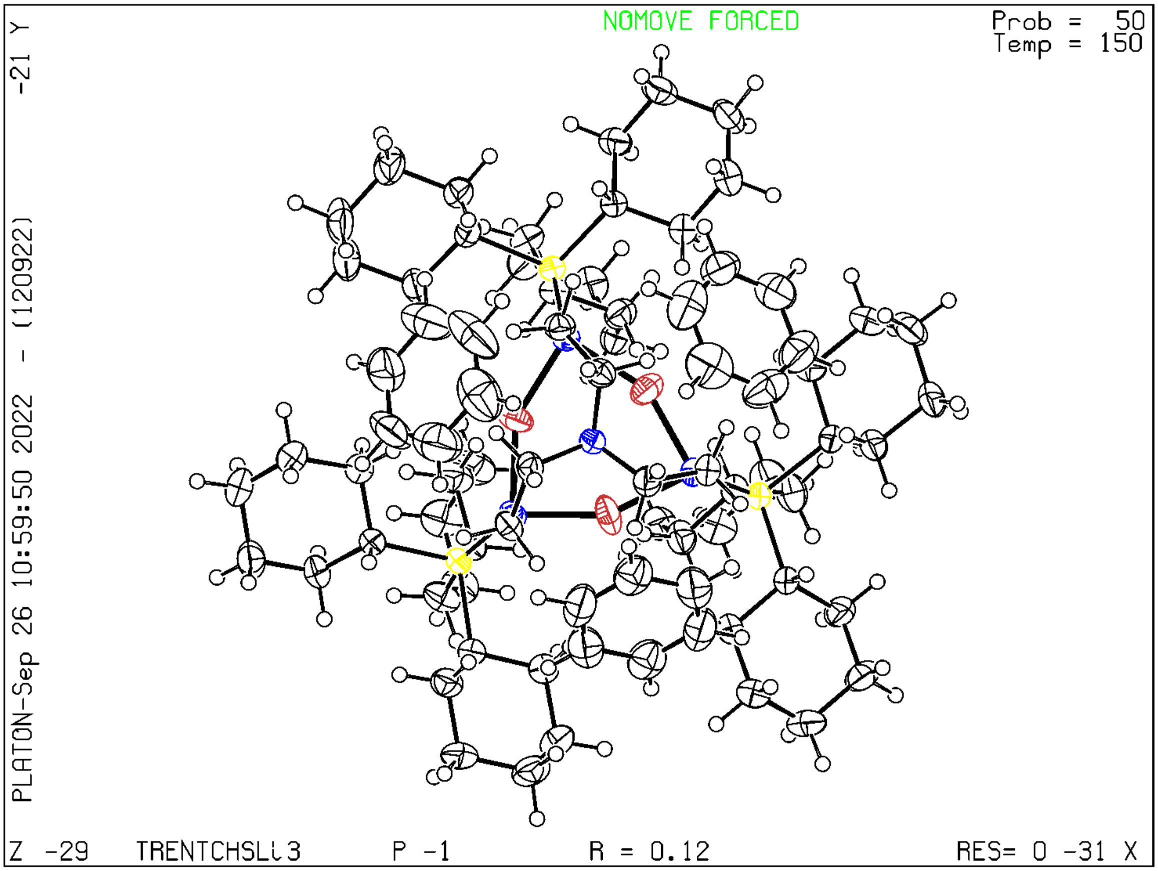

Datablock 1 - ellipsoid plot

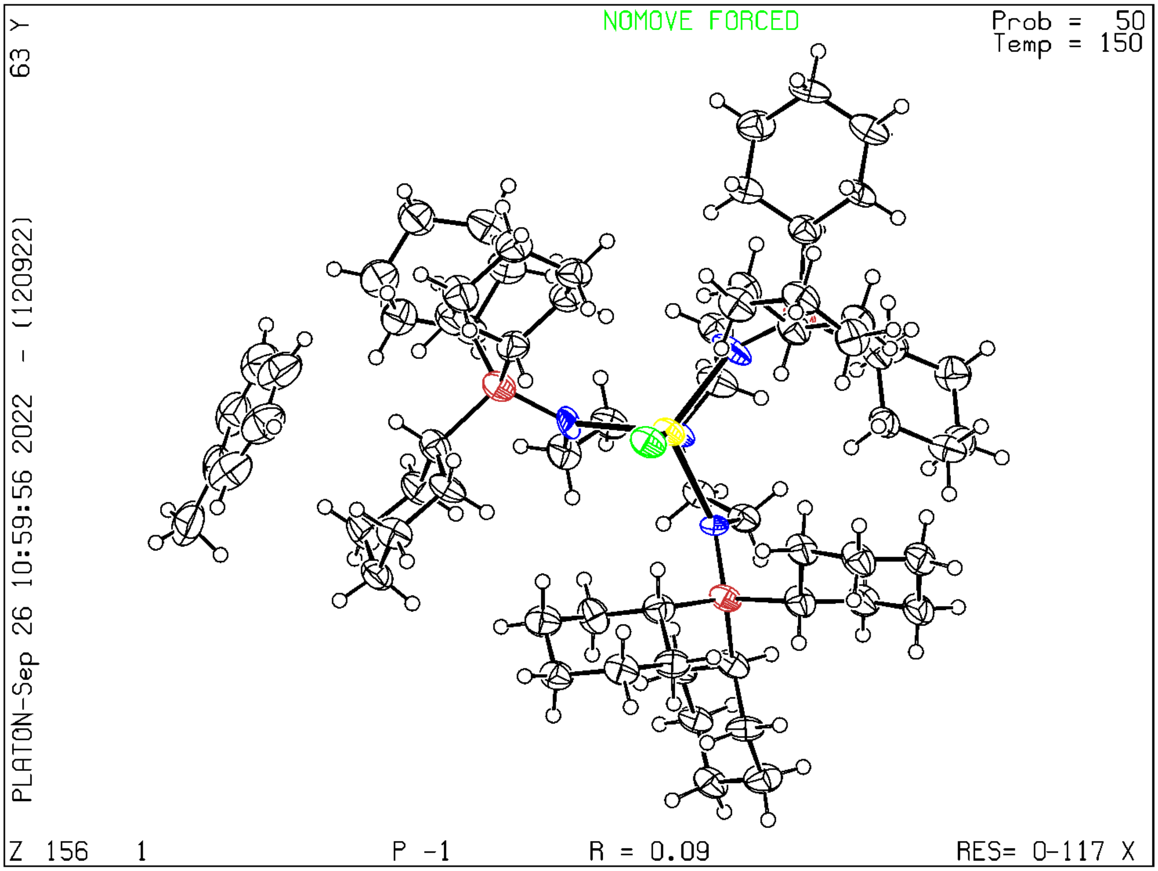

Datablock 2 - ellipsoid plot

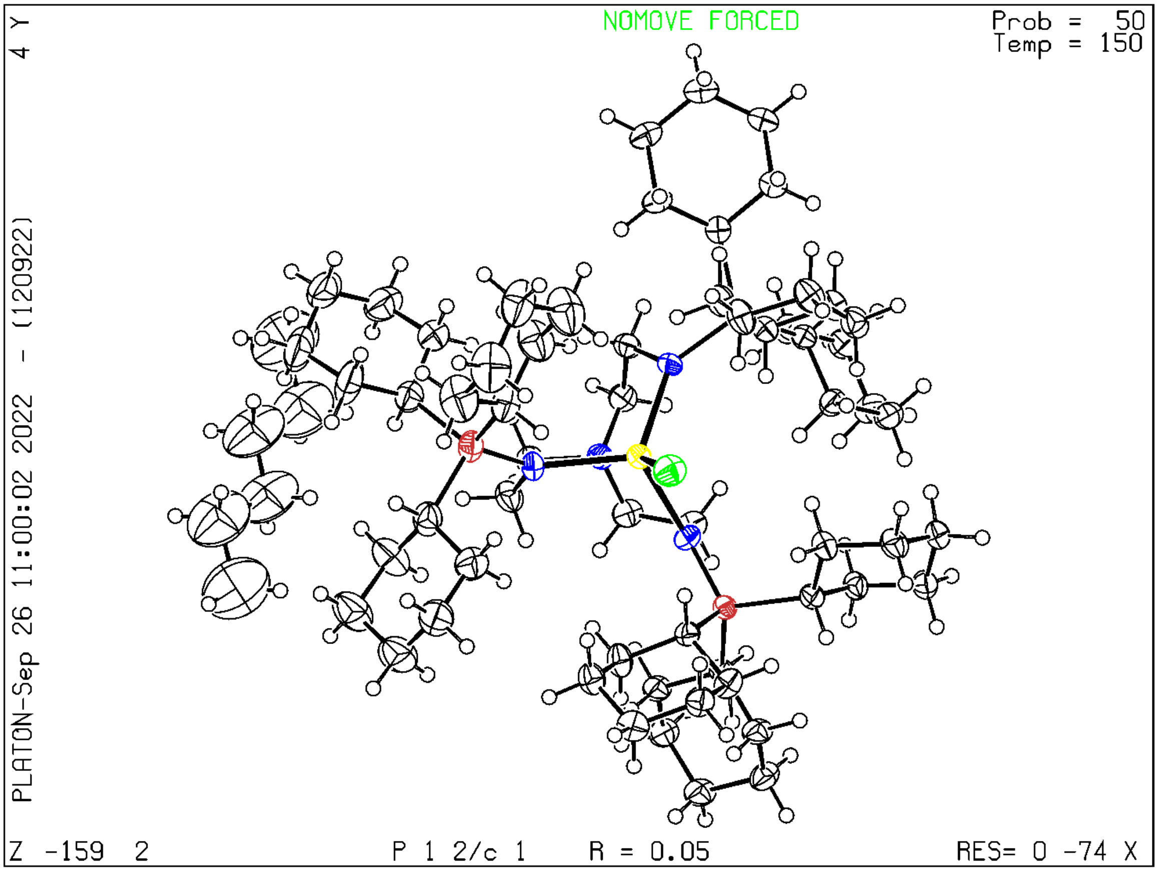

Datablock 3 - ellipsoid plot

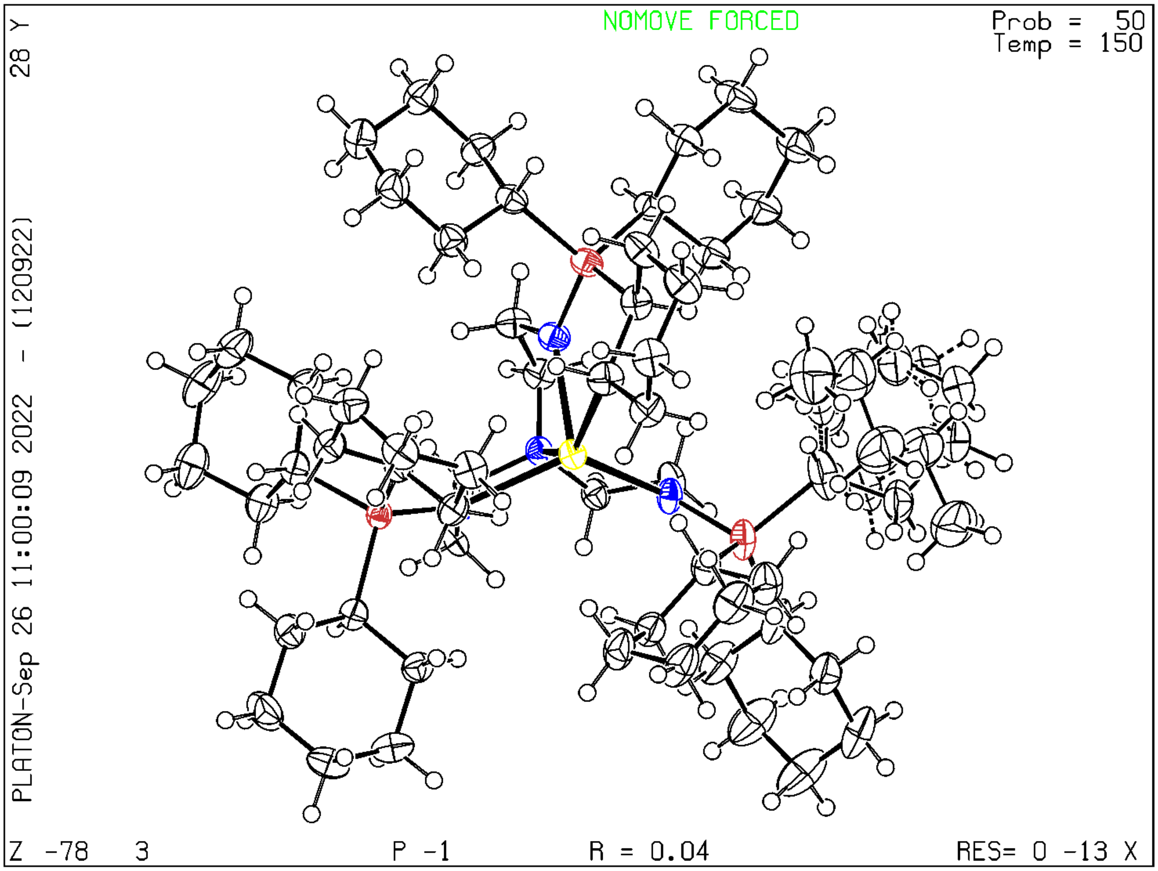

Datablock 4 - ellipsoid plot

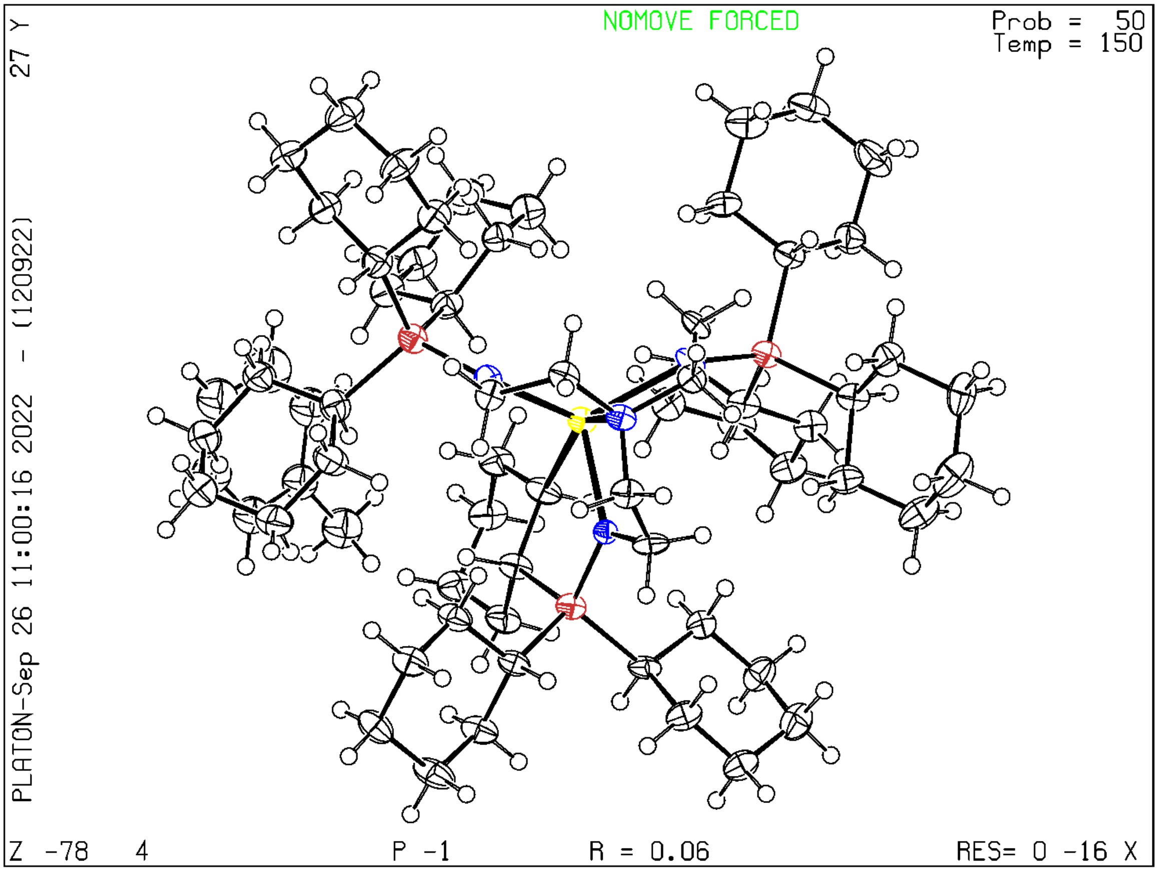

Datablock 5 - ellipsoid plot

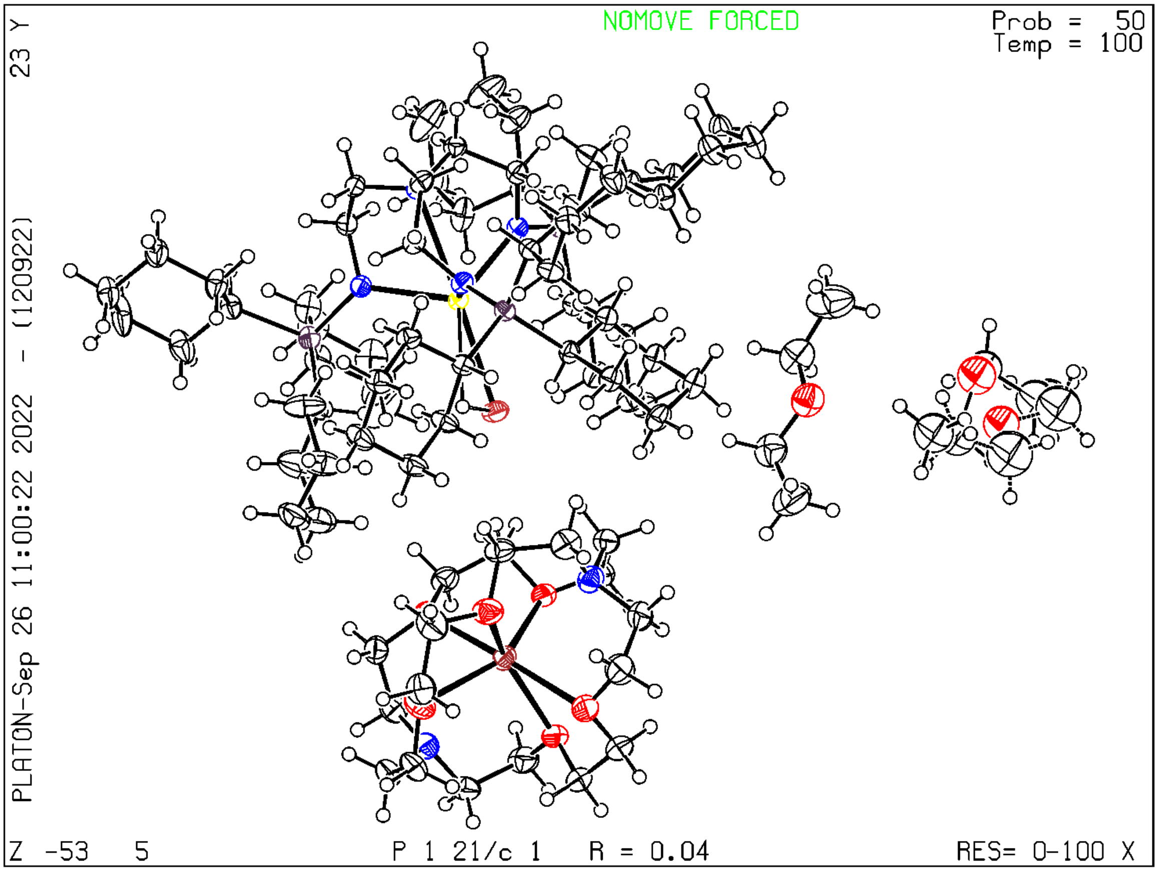

Datablock 6 - ellipsoid plot

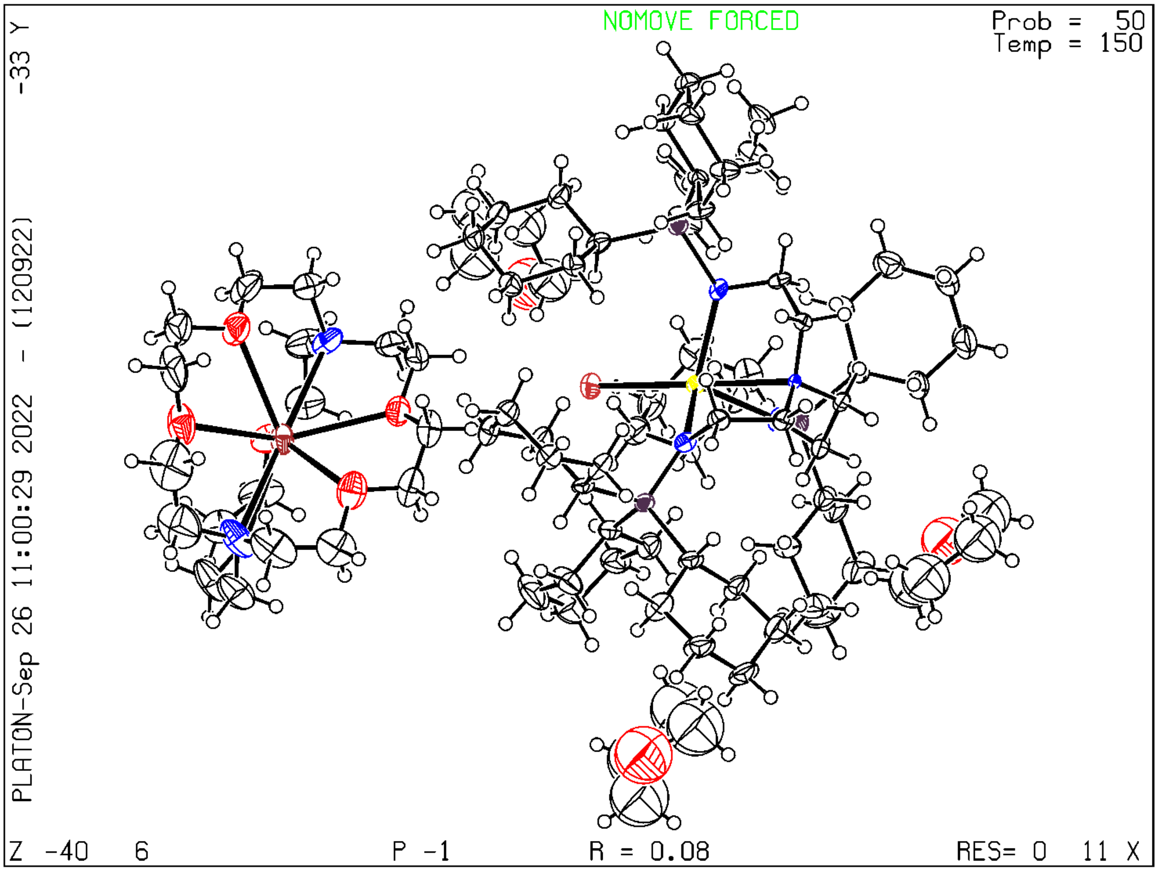

Datablock 7 - ellipsoid plot

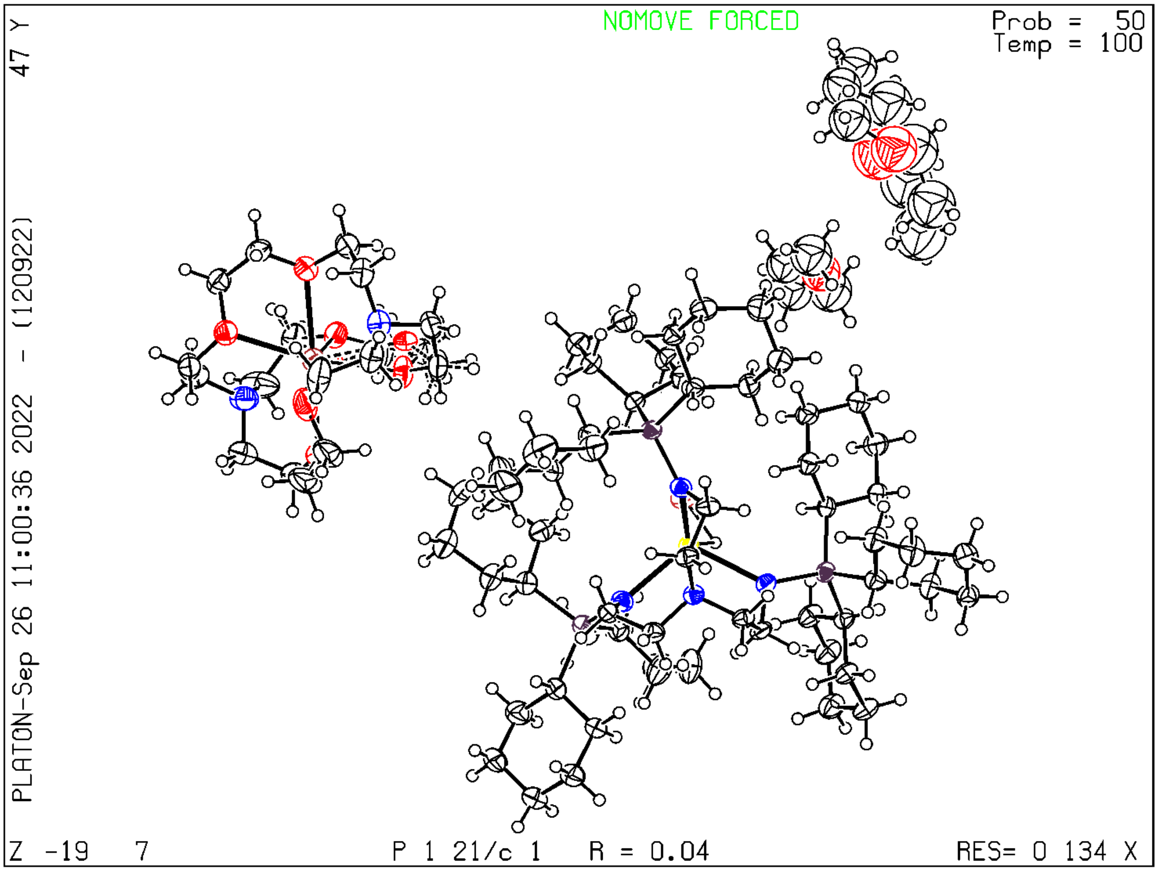

Datablock 8 - ellipsoid plot

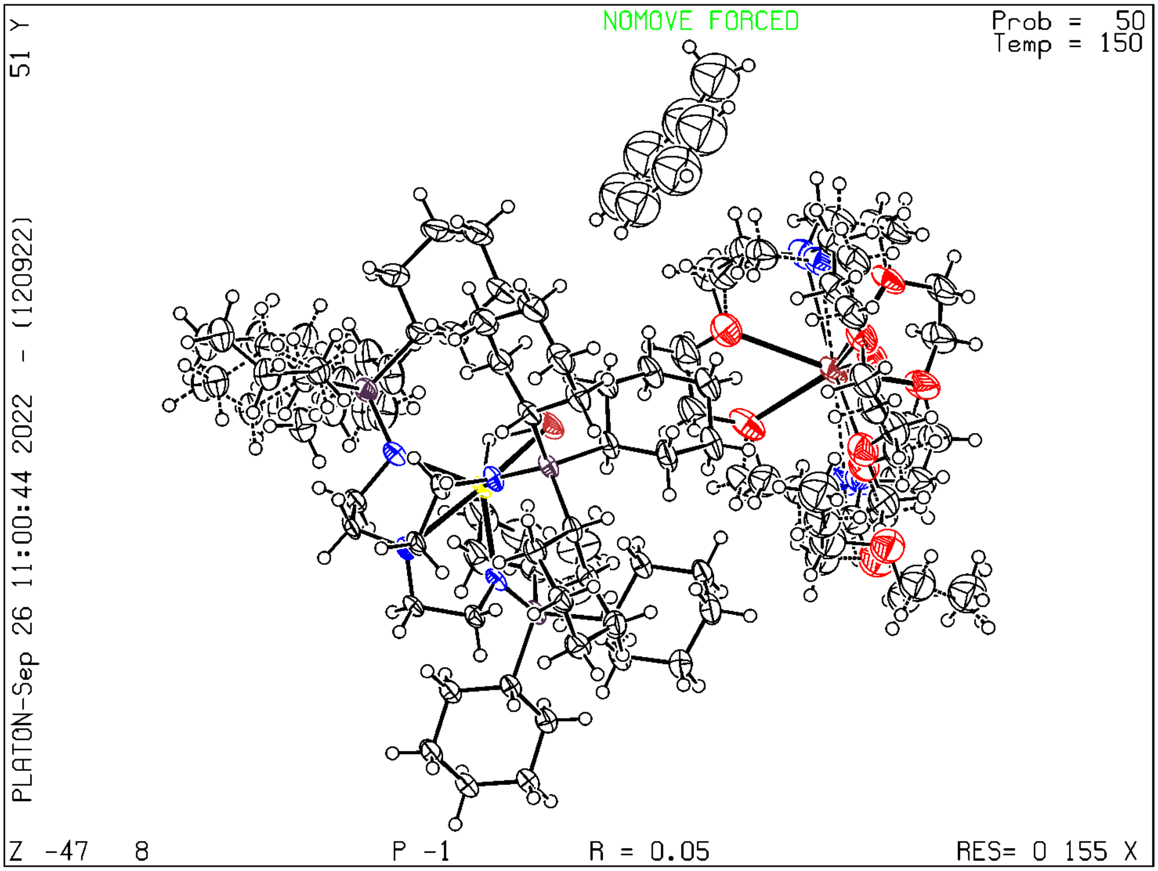

Datablock 9 - ellipsoid plot

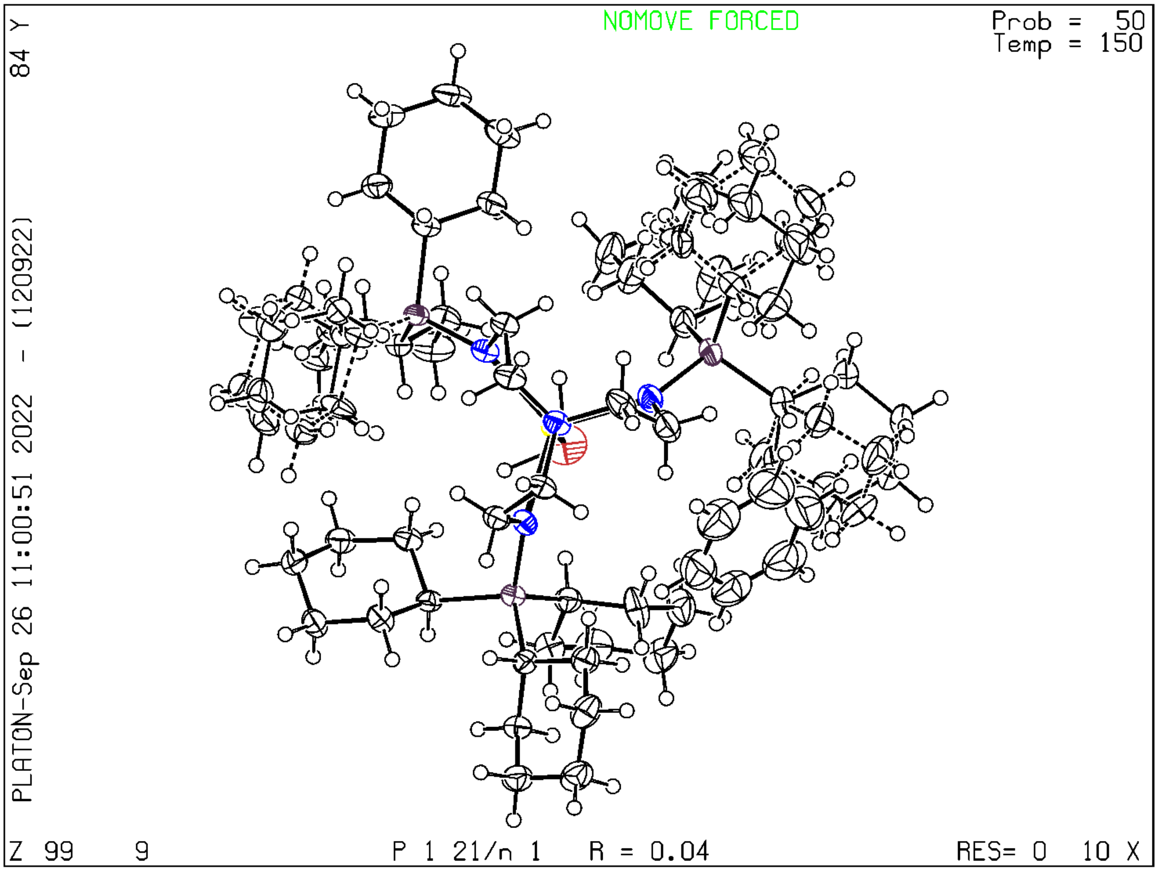

Datablock 10 - ellipsoid plot

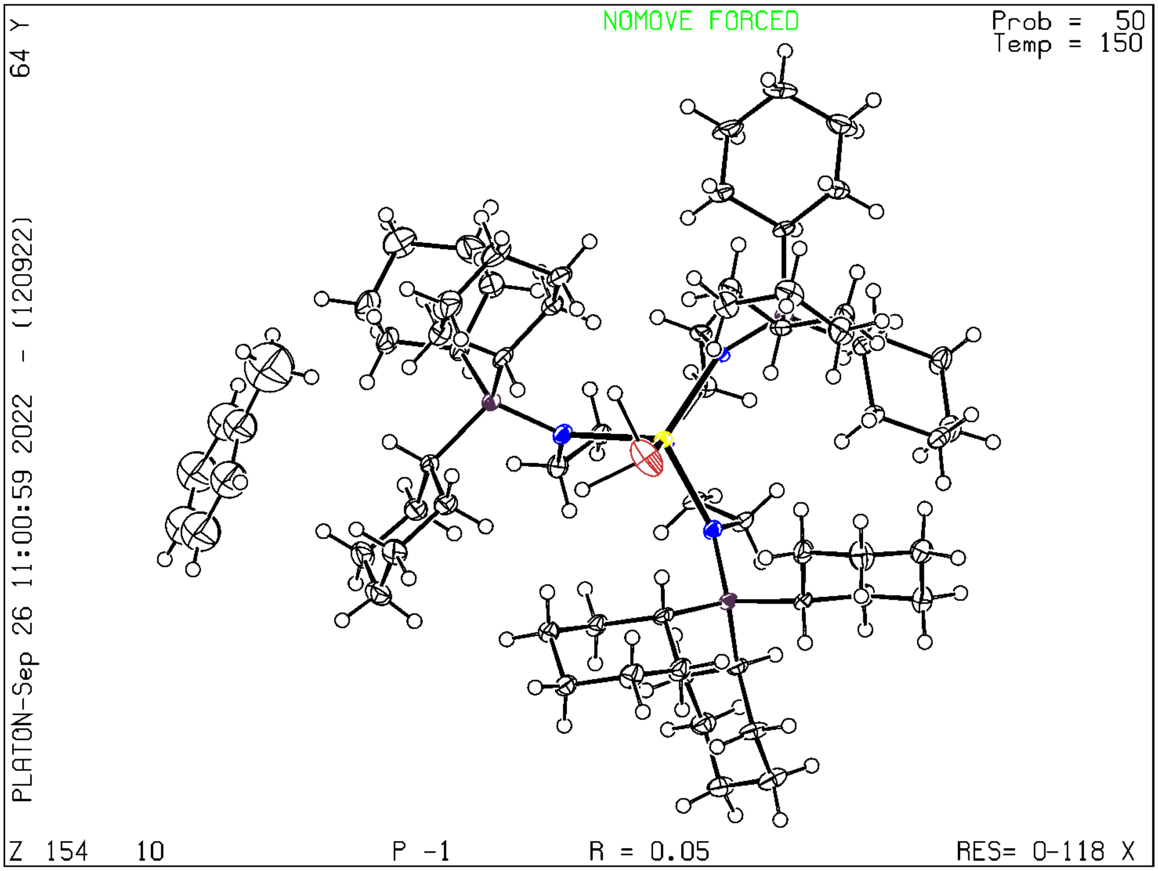

Datablock 11 - ellipsoid plot

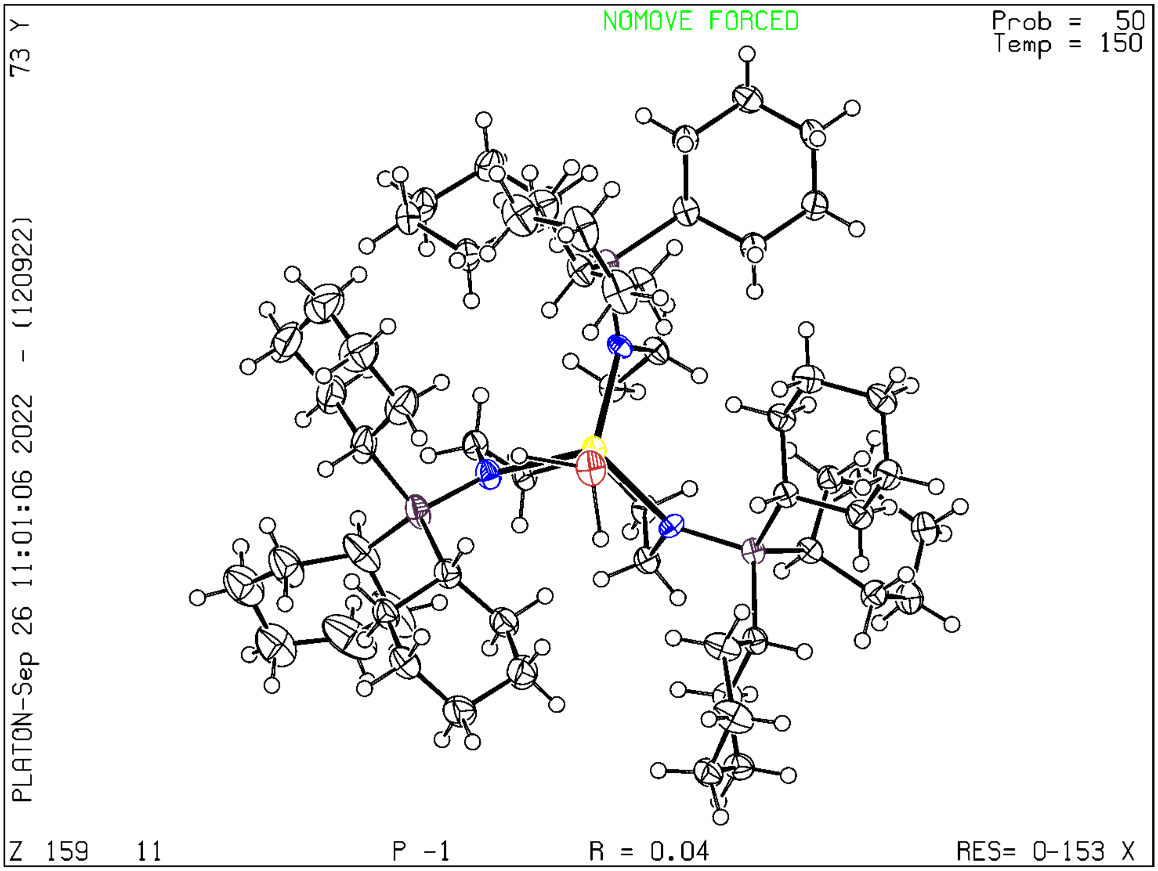

Datablock 12 - ellipsoid plot

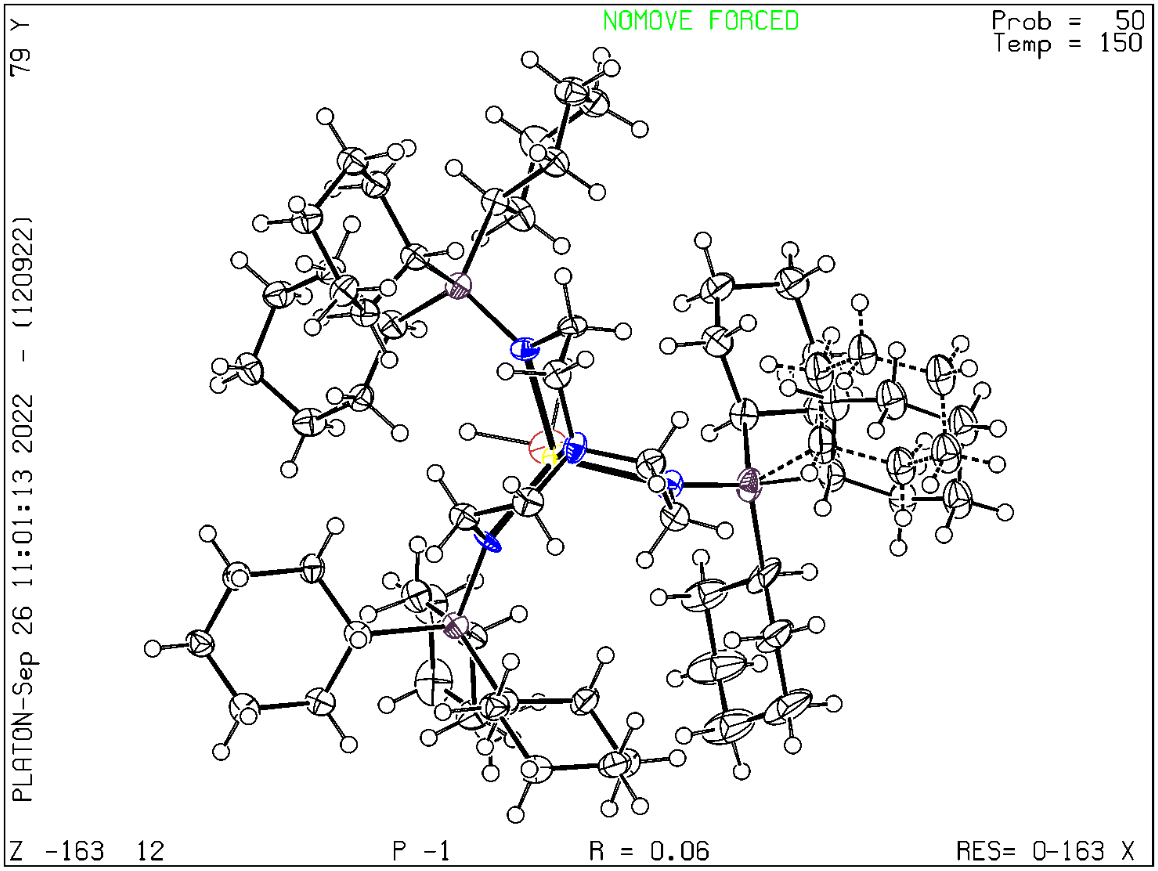

Supplement: Supplementary file 1 — Supporting Information [file ANIE-61-0-s001.pdf]
